# Recognition and revision of the *Phelister blairi* group (Histeridae, Histerinae, Exosternini)

**DOI:** 10.3897/zookeys.1001.58447

**Published:** 2020-12-09

**Authors:** Michael S. Caterino, Alexey K. Tishechkin

**Affiliations:** 1 Department of Plant & Environmental Sciences, Clemson University, Clemson, SC 29634, USA Clemson University Clemson United States of America; 2 California Dept. of Food and Agriculture Plant Pest Diagnostics Center, Sacramento, CA 95832, USA California Dept. of Food and Agriculture Plant Pest Diagnostics Center Sacramento United States of America

**Keywords:** Biodiversity, Histeroidea, myrmecophily, taxonomy

## Abstract

Forty-nine new species of Neotropical Exosternini are described in this work, representing the newly recognized *Phelister blairi* species group, within the large, heterogeneous taxon *Phelister*. Eight previously described species are also assigned to this group. Relationships within are indicated with several informal subgroups: *P. blairi* subgroup: (*P. blairi* Hinton, 1935, *P. erwini***sp. nov.**, *P. fimbriatus***sp. nov.**, *P. stellans***sp. nov.**, *P. sparsus***sp. nov.**, *P. pretiosus***sp. nov.**, *P. trigonisternus* Marseul, 1889, *P. globosus***sp. nov.**, *P. serratus***sp. nov.**, *P. geminus***sp. nov.**, *P. parana***sp. nov.**, *P. asperatus***sp. nov.**, *P. uniformis***sp. nov.**, *P. miscellus***sp. nov.**, *P. inbio***sp. nov.**, *P. sculpturatus* Schmidt, 1893, *P. tunki***sp. nov.**, *P. praedatoris* Reichensperger, 1939, *P. ifficus***sp. nov.**, *P. genieri***sp. nov.**, *P. marginatus***sp. nov.**, *P. vazdemelloi***sp. nov.**, *P. dilatatus***sp. nov.**, *P. spectabilis***sp. nov.**, *P. pervagatus***sp. nov.**); *P. amazoniae* subgroup: (*P. morbidus***sp. nov.**, *P. annulatus***sp. nov.**, *P. sphaericus***sp. nov.**, *P. geijskesi* Kanaar, 1997, *P. fraternus***sp. nov.**, *P. conjunctus***sp. nov.**, *P. chabooae***sp. nov.**, *P. striatinotum* Wenzel & Dybas, 1941, *P. notandus* Schmidt, 1893, *P. amazoniae* (Lewis, 1898) **comb. nov.**, *P. arcuatus***sp. nov.**); *P. gregarius* subgroup: (*P. gregarius***sp. nov.**, *P. praecisus***sp. nov.**, *P. rudis***sp. nov.**, *P. incongruens***sp. nov.**, *P. congruens***sp. nov.**, *P. praesignis***sp. nov.**); *P. umens* subgroup: (*P. umens***sp. nov.**, *P. almeidae***sp. nov.**, *P. chicomendesi***sp. nov.**, *P. microdens***sp. nov.**, *P. matatlantica***sp. nov.**); *P. curvipes* subgroup: (*P. curvipes***sp. nov.**, *P. vilavelha***sp. nov.**); *P. rio* subgroup: (*P. rio***sp. nov.**, *P. semotus***sp. nov.**, *P. uncinatus***sp. nov.**, *P. inscriptus***sp. nov.**); *incertae sedis* – unplaced to subgroup: (*P. incertus***sp. nov.**, *P. okeefei***sp. nov.**, *P. blairoides***sp. nov.**, *P. pirana***sp. nov.**). Lectotypes are designated for the following species: *P. trigonisternus* Marseul, *P. sculpturatus* Schmidt, *P. praedatoris* Reichensperger, *P. notandus* Schmidt, and *Discoscelis amazoniae* Lewis. Preliminary phylogenetic analyses of the broader Neotropical Exosternini do not support the monophyly of the *P. blairi* group, nor of all of these subgroups, but the majority do fall within one large clade (which is potentially paraphyletic with respect to some other Neotropical exosternine genera). More work on the phylogeny and taxonomy of this diverse fauna is needed.

## Introduction

The Neotropical Exosternini represent one of the largest radiations of Histeridae, with more than 650 species (Caterino and Tishechkin, unpub. data). To date fewer than half of these have been described (298 spp.), but even this represents a three-fold increase over the past decade. As relationships and taxonomic limits have slowly improved, we have carved off and revised a number of subgroups/genera (Caterino et al. 2012, 2013; Caterino and Tishechkin 2013a, b, 2014, 2016, 2019). However, the fauna remains a complicated one, containing many small, similar-looking species with relatively few distinguishing features. Attempts to resolve these relationships in order to delineate more meaningful taxa have met with mixed success (Caterino and Tishechkin 2015), and we have largely taken a pragmatic approach, under the presumption that increasing knowledge of the species level diversity across the tribe will lead to further improvements in our understanding of the higher level relationships. We continue that approach here, expanding the scope of the already large taxon *Phelister*.

In the present paper, we circumscribe a new subgroup of *Phelister*, the *P. blairi* group, redescribing several previously described species that we assign here, describe a large number of species, and perform a phylogenetic assessment of where they fit in among the other neotropical Exosternini. The *P. blairi* group contains 57 species, 49 of them being described here as new. We allocate most of these to six informal subgroups, leaving four unplaced to subgroup. Informally, the authors have long referred to these as ‘the scutellar impression group’, and most (not all) of the included species exhibit a distinct prescutellar impression. More formally, we have previously referred to these as ‘*Phelister blairi* group’, though without explicitly assigning species to it (Caterino and Tishechkin 2013b). Most (7 out of 8) of the previously described species here have been assigned to *Phelister*. However, the sole exception points out the primary challenge with this group: this is *Reninus amazoniae* (Lewis), clearly a relative of *Phelister blairi* and other more generalized species, but until now assigned to a genus of Haeteriinae. In fact, many Neotropical Exosternini exhibit highly modified morphologies, attributable in most to inquilinism with social insects, mainly ants. The *Phelister blairi* group encompasses one or more such transitions into inquilinous habits, and offers an interesting array of morphological characters accompanying the transition.

In our previous phylogenetic analysis of Neotropical Exosternini (Caterino and Tishechkin 2015), most of the species included here fell out within a large clade sister to much of *Phelister*. However, there were a number of exceptions to their exclusivity. Several species that we contend should be closely related fell elsewhere among the diverse *Phelister* species. In other cases, some falling outside the ‘*blairi* group’ would support preliminary hypotheses that the similarities shared (a prescutellar impression, mainly) were homoplasious. On the other hand, the core *blairi* group clade also appears to be paraphyletic with respect to several other taxa, especially if we include *Phelister striatinotum* in our *blairi* group concept, which we have. The few ‘typical *Phelister*’ inclusions are all species that have large amounts of missing data (male genitalia and DNA, especially), and for now cannot be adequately evaluated. However, the inclusion of most of the genera that we recently described (*Pyxister*, *Crenulister*, *Conocassis*, etc.; Caterino and Tishechkin 2014) as well as the very large genus *Operclipygus* poses significant difficulties. To address these, we present some new analyses, based on a reduced taxon set (allowing more thorough analysis of more contentious relationships), and discuss some slightly refined perspectives on relationships below.

## Materials and methods

The morphological terminology used is that defined by Wenzel and Dybas (1941), supplemented by Helava et al. (1985), Ôhara (1994), and Caterino and Tishechkin (2013b). Following histerid conventions, total body length is measured from the anterior margin of the pronotum to the posterior margin of the elytra (to exclude preservation variability in head and pygidial extension), while width is taken at the widest point, generally near the elytral humeri. Conventional imaging was done using Leica’s Photomontage imaging system and Visionary Digital’s ‘Passport’ portable imaging system, which incorporates a Canon 7D with MP-E 65 mm 1–5 × macro zoom lens. Images obtained with the Passport system were stacked using Helicon Focus software. SEM imaging was done on a Zeiss EVO 40 scope, and the specimens were sputter-coated with gold. We have studied and imaged primary types of all species; images of these type specimens beyond those presented in this paper are available from the authors. Data quoted verbatim from labels are surrounded by double quotes (“_”) with distinct labels separated by slashes (/). Non-verbatim data (paratypes and ‘other material’) are summarized in a standard format. GPS coordinates have been inferred for most localities and/or standardized to decimal degrees. Dates outside of verbatim records are presented as MM/DD/YY.

**Specimens from following institutions were examined**:

**AKTC** Alexey Tishechkin Collection, Sacramento, USA;

**AVSC** Alexander Sokolov Collection, Moscow, Russia;

**CASC**California Academy of Sciences Collection, San Francisco, USA;

**CESP**Coleção Entomológica do Setor Palotina, Universidade Federal do Paraná, Palotina, Brazil;

**CEMT**Setor de Entomologia da Coleção de Zoológica da Universidade Federal de Mato Grosso, Cuiabá, Brazil;

**CERPE**Coleção Entomológica da Universidade Federal Rural de Pernambuco, Recife, Brazil;

**CHND** Nicolas Dégallier Collection, Paris;

**CHSM** Slawomir Mazur Collection, Genova, Italy;

**CMNC**Canadian Museum of Nature, Ottawa, Canada;

**CSCA**California State Collection of Arthropods – CDFA, Sacramento, USA;

**CUAC**Clemson University Arthropod Collection, Clemson, USA;

**DZUP**Coleção Entomológica Padre Jesus Santiago Moure, Departamento de Zoologia, Universidade Federal do Paraná, Curitiba, Brazil;

**FMNH**Field Museum, Chicago, USA;

**FSCA**Florida State Collection of Arthropods, Gainesville, USA;

**INBIO**Instituto Nacional de Biodiversidad, San Jose, Costa Rica;

**LSAM**Louisiana State Arthropod Museum, Baton Rouge, USA;

**MNCR**Museo Nacional de Costa Rica, San Jose, Costa Rica;

**MNHN**Museum National d’Histoire Naturelle, Paris, France;

**MSCC** Michael Caterino Collection, Clemson, USA;

**MUSM**Colección Entomológica del Museo de Historia Natural de la Universidad Nacional Mayor de San Marcos, Lima, Peru;

**NHMUK**Natural History Museum, London, UK;

**OUMNH** Hope Museum of Natural History, Oxford, UK;

**RMNH**Naturalis Biodiversity Center, Leiden, Netherlands;

**SEMC**Snow Entomology Museum, University of Kansas, Lawrence, USA;

**TAMU**Texas A&M University Collection, College Station, USA;

**UCONN**Rettenmeyer Army Ant Guest Collection, Biodiversity Research Collections, University of Connecticut, Storrs, USA;

**UNESP**Universidade Estadual Paulista, Faculdade de Engenharia de Ilha Solteira, Ilha Solteira, Brazil;

**USFQ**Universidad San Francisco de Quito, Ecuador;

**USNM**National Museum of Natural History, Washington, USA;

**WBWC** Bill Warner Collection, Chandler, USA;

**ZMHB** Zoological Museum of Humboldt University, Berlin, Germany.

### Phylogeny

We analyzed a subset of taxa from the 750+ taxon data set of Caterino and Tishechkin (2015) to attempt to better resolve species hypothesized to belong to the *Phelister blairi* group. This pruned data set included only small numbers of exemplars for those groups previously strongly supported as monophyletic. Specifically, it includes only three species of *Baconia* Lewis, two species of *Hypobletus* Schmidt, and one of *Megalocraerus* Lewis. We included twelve species of *Operclipygus* Marseul, given potentially close relationships to the *blairi* group, and most species of recently described genera (Caterino and Tishechkin 2014) with potential relationships to the *blairi* group. We reduced the number of outgroups to 12 (from 61), representing non-neotropical Exosternini, other tribes of Histerinae, and Haeteriinae. Finally, we added five newly discovered or recognized species within our concept of the *blairi* group (and removed a few others now considered synonyms of other species). This revised data set included a total of 235 taxa, including most described and undescribed *Phelister* and *Pseudister* spp., as well as many other undescribed species of uncertain placement. The included data for all partitions are identical to those included in our 2014 analysis. This included 259 morphological characters (141 external morphology, 87 of male genitalic morphology, and 31 of female genitalic morphology), and approximately 1/4 were represented by some molecular data, including some combination of 18S, 28S, and cytochrome oxidase I. Molecular data were sparse for *blairi* group taxa, as most of these have been collected only through flight interception trapping. Suppl. material 1: Table S1 details what species were included and what partitions were available for them. We did not realign the length variable portions for this reduced dataset, maintaining homology assessments from the preceding analysis. For original alignment parameters see Caterino and Tishechkin (2015). This reduced data set is available as an online supplement (Suppl. material 2). Tree searching was performed in PAUP* (v. 4.0a164; Swofford 2002) under the maximum parsimony criterion, running 1000 random sequence addition replicates, saving no more than 2000 trees for each replicate.

## Results

### Phylogeny

**Figure 1.**
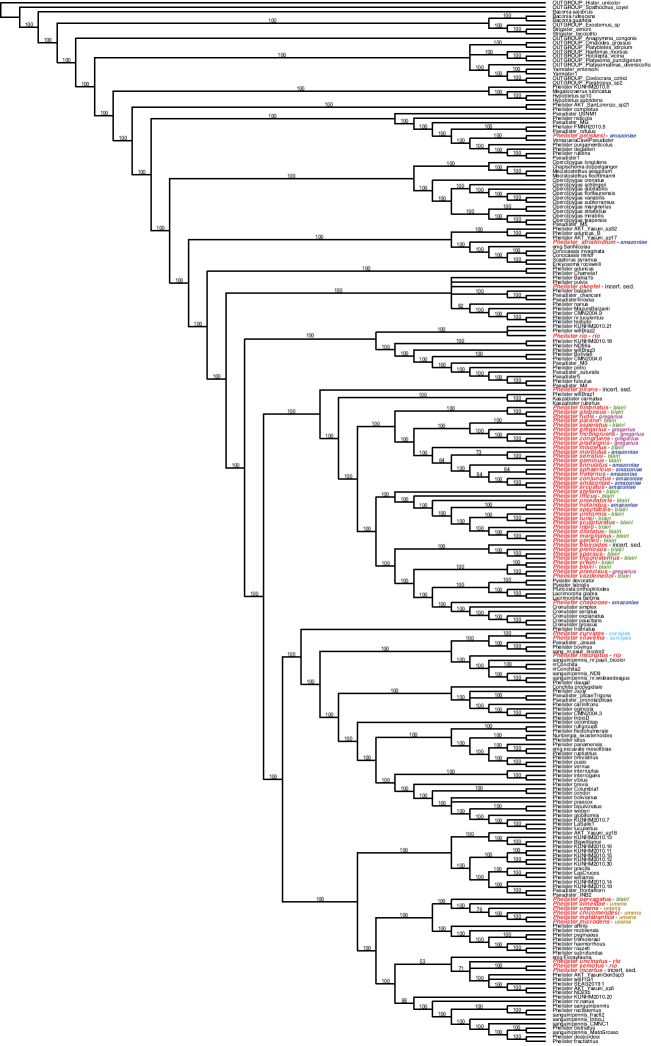
Majority rule consensus of 131648 trees of 13048 steps (CI: 0.1752; RI: 0.4006). Selected members of all Neotropical Exosternini higher taxa are included. Those we assign to the *Phelister blairi* group have their names in bold red font. The subgroups that we assign them to are indicated by colored names at the right (red = *rio* subgroup, green = *blairi* subgroup, dark blue = *amazoniae* subgroup, light blue = *curvipes* subgroup, gold = *umens* subgroup, violet = *gregarius* subgroup). Numbers above branches represent majority rule consensus indices.

This analysis yielded 131648 trees of 13048 steps (CI: 0.1752; RI: 0.4006). The majority rule consensus of these (Fig. 1) is remarkably well resolved, with high consistency of the vast majority of branches. However, the consistency of the topology with the current taxonomy of Neotropical Exosternini is very low – few genera represented by multiple species are monophyletic. With respect to the possible monophyly of the *P. blairi* group, which we establish here, monophyly is also not supported. The species that we treat here as the *blairi* group (names in red in Fig. 1) fall out in 10 places in the tree. Still, the majority (39 spp.) do fall out together, together constituting most of a clade, the only exceptions being a few small genera which have been named as distinct (including *Crenulister* Caterino & Tishechkin, [2014], to which one of the species we describe here might possibly be related). This large lineage includes most members of the *blairi*, *gregarius*, and *amazoniae* subgroups with the *blairi* subgroup appearing potentially paraphyletic with respect to the others. The *amazoniae* subgroup species that are not resolved within this lineage (*P. geijskesi* and *P. striatinotum*) are indeed species that exhibit some inconsistent characters. Three subgroups fall entirely outside of this group. Two of these, the *curvipes* and *umens* subgroups do appear monophyletic, though far from each other and the rest of the *blairi* group. The *umens* subgroup is resolved as the sister of the *P. haemorrhous* group, which we recently revised (Caterino & Tishechkin, 2019). The *rio* subgroup falls out in three separate places on the tree, suggesting that the characters those species share are mostly plesiomorphies or convergences. Overall, despite the obvious problems this phylogenetic hypothesis poses for an ideally cladistic nomenclature, this tree does offer improved perspective on possible relationships across *Phelister* and helps point out areas in which further characters are needed. As we continue to accumulate DNA-grade specimens of these taxa, we look forward to continued improvement in understanding their relationships.

### Taxonomy

#### Diagnosis of the *Phelister blairi* group

**Figure 2.**
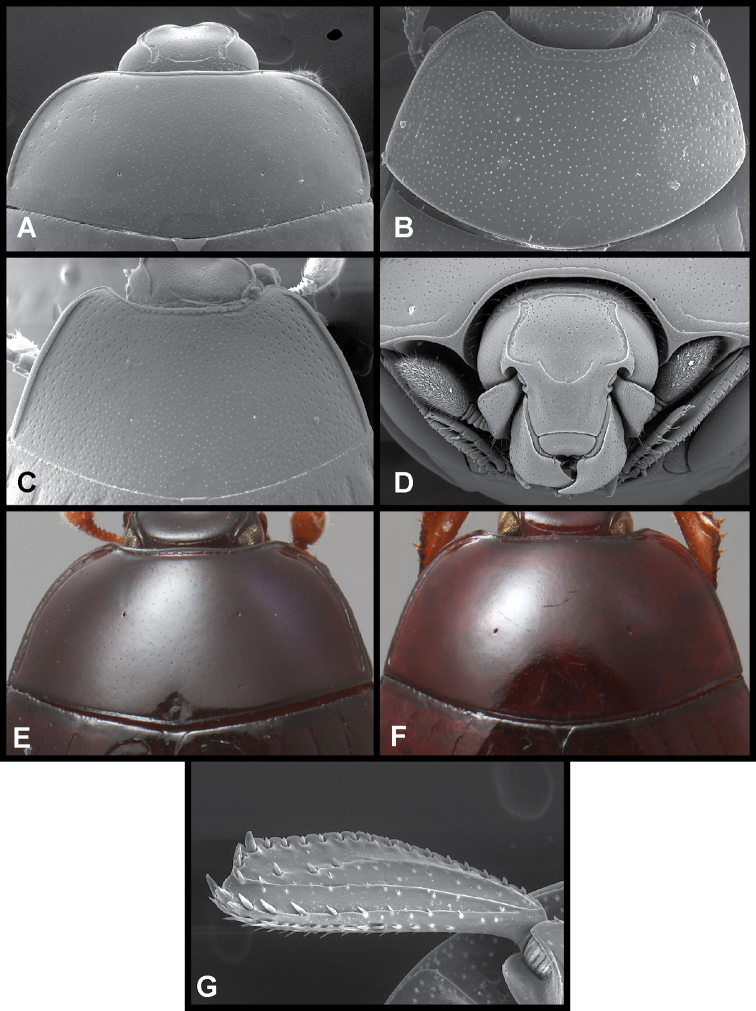
Images of selected characters **A** SEM of pronotum of *Phelister geminus*, showing prescutellar impression and annulate median pronotal glands **B** SEM of pronotum of *P. erwini*, showing prescutellar impression and annulate median pronotal glands **C** SEM of pronotum of *Phelister sculpturatus*, showing prescutellar impression and annulate median pronotal glands **D** head of *P. geminus*, showing depressed frons and epistoma, and emarginate labrum **E** pronotum of *P. uncinatus*, showing non-annulate median pronotal gland openings **F** pronotum of *P. semotus*, showing non-annulate median pronotal gland openings **G** protibia of *P. geminus*, showing ‘scalloped’ protibial margin.

Prescutellar impression (Fig. 2A–C, E, F) as large or larger than the scutellum, posteriorly confluent with the pronotal margin; frons and epistoma (Fig. 2D) fairly strongly depressed, epistoma sometimes ridged around the margin of this depression; frontal stria almost always present, often interrupted medially or at sides or both; distal margin of labrum emarginate, weakly transversely ridged at middle, sometimes subcarinate; anterior arch of mesometaventral stria reaching middle of mesoventrite or beyond, usually angulate at middle, sometimes arched; lateral submarginal pronotal stria, if present, close to margin, the marginal bead frequently raised between the stria and the edge; median pronotal gland openings (Fig. 2A–C, E, F; sensu Caterino and Tishechkin 2013b) conspicuous, usually annulate (with a fine stria encircling opening), located on posterior 1/2 of disk in most species; anterior marginal pronotal stria crenulate, usually entire and slightly separated from margin behind head, occasionally interrupted behind eyes and recurved posterad (as in *P. blairi* and a few others).

#### Checklist of the species treated in this paper

*Phelister blairi* subgroup

1. *P. blairi* Hinton, 1935
2. *P. erwini* sp. nov.
3. *P. fimbriatus* sp. nov.
4. *P. stellans* sp. nov.
5. *P. sparsus* sp. nov.
6. *P. pretiosus* sp. nov.
7. *P. trigonisternus* Marseul, 1889
8. *P. globosus* sp. nov.
9. *P. serratus* sp. nov.
10. *P. geminus* sp. nov.
11. *P. parana* sp. nov.
12. *P. asperatus* sp. nov.
13. *P. uniformis* sp. nov.
14. *P. miscellus* sp. nov.
15. *P. inbio* sp. nov.
16. *P. sculpturatus* Schmidt, 1893a
17. *P. tunki* sp. nov.
18. *P. praedatoris* Reichensperger, 1939
19. *P. ifficus* sp. nov.
20. *P. genieri* sp. nov.
21. *P. marginatus* sp. nov.
22. *P. vazdemelloi* sp. nov.
23. *P. dilatatus* sp. nov.
24. *P. spectabilis* sp. nov.
25. *P. pervagatus* sp. nov.

*P. amazoniae* subgroup

26. *P. morbidus* sp. nov.
27. *P. annulatus* sp. nov.
28. *P. sphaericus* sp. nov.
29. *P. geijskesi* Kanaar, 1997
30. *P. fraternus* sp. nov.
31. *P. conjunctus* sp. nov.
32. *P. chabooae* sp. nov.
33. *P. striatinotum* Wenzel & Dybas, 1941
34. *P. notandus* Schmidt, 1893b
35. *P. amazoniae* (Lewis, 1898)
36. *P. arcuatus* sp. nov.

*P. gregarius* subgroup

37. *P. gregarius* sp. nov.
38. *P. praecisus* sp. nov.
39. *P. rudis* sp. nov.
40. *P. incongruens* sp. nov.
41. *P. congruens* sp. nov.
42. *P. praesignis* sp. nov.

*P. umens* subgroup

43. *P. umens* sp. nov.
44. *P. almeidae* sp. nov.
45. *P. chicomendesi* sp. nov.
46. *P. microdens* sp. nov.
47. *P. matatlantica* sp. nov.

*P. curvipes* subgroup

48. *P. curvipes* sp. nov.
49. *P. vilavelha* sp. nov.

*P. rio* subgroup

50. *P. rio* sp. nov.
51. *P. semotus* sp. nov.
52. *P. uncinatus* sp. nov.
53. *P. inscriptus* sp. nov.

*incertae sedis* – unplaced to subgroup

54. *P. incertus* sp. nov.
55. *P. okeefei* sp. nov.
56. *P. blairoides* sp. nov.
57. *P. pirana* sp. nov.

#### Key to the species of the *Phelister blairi* group

This key will diagnose members of the *P. blairi* group from any other *Phelister*, and will also separate them from members of similar genera, particularly *Operclipygus. Phelister blairi* group species are numbered according to their order of appearance in the text below.

**Table:** 

1	Pronotum with prescutellar impression distinct (a flat depressed area delimited by a distinct stria immediately anterior to the scutellum; e.g., Fig. 2A–C)	**2**
–	Pronotal prescutellar impression poorly defined or absent	**7**
2	Anterior margin of pronotum behind head produced; prescutellar impression usually little larger than scutellum; prosternal keel striae usually weak to obsolete; base of prosternal keel weakly emarginate	[***Phelister sanguinipennis* group**]
–	Anterior margin of pronotum behind head straight or posteriorly concave, rarely very weakly produced; prescutellar impression usually substantially larger than scutellum; prosternal keel striae usually well developed, base of prosternal keel usually distinctly emarginate	**3**
3	Median pronotal gland openings no more than three diameters from anterior margin of pronotum, never annulate	[**various *Phelister* & *Operclipygus***]
–	Median pronotal gland openings more than three gland diameters from anterior margin (if pronotal gland openings are unclear because of dense pronotal ground punctation, go to *Phelister blairi* group key, starting at couplet 14), usually distinctly annulate (if non-annulate, then inner margins of protibiae curved)	**4**
4	Pronotal gland openings not annulate, a simple hole with no ring around it (Fig. 2E–F)	**5**
–	Pronotal gland openings distinctly annulate (e.g., Fig. 2A–C)	**6**
5	Prescutellar impression as wide or wider than scutellum, usually much wider; frons and epistoma typically longitudinally depressed (e.g., Fig. 2D)	[***Phelister blairi* group] 14**
–	Prescutellar impression not as wide as scutellum, or thin and elongate; frons and epistoma rarely strongly impressed	[**various *Phelister* & *Operclipygus***]
6	Body size very small, ~ 1 mm or less; lateral submarginal pronotal stria absent; frons and epistoma only weakly or not at all impressed; pronotal gland openings from 4–6 diameters from the anterior pronotal margin; antennal club barely elongate	[**various *Phelister***]
–	Body size generally larger; lateral submarginal stria usually present (may be variously abbreviated); frons and epistoma moderately to strongly depressed along midline (except in *P. geijskesi*); pronotal gland openings nearly always more than six diameters from anterior pronotal margin (exception: *P. blairi*); antennal club usually markedly elongate	[***Phelister blairi* group] 14**
7	Inner subhumeral stria raised to form a marginal elytral carina aligned with lateral pronotal margin; pronotal disk explanate along inner edge of submarginal pronotal stria (e.g., Fig. 21C, E); entire body (dorsum and venter) densely punctate; body rounded, subdepressed; tibiae moderately widened	[***Phelister blairi* group] 14**
–	Inner subhumeral stria only very rarely forming marginal elytral carina; if it does, pronotum not strongly explanate	**8**
8	Elytral striae 3–5 and sutural stria absent	**9**
–	At least sutural and 3^rd^ elytral striae present	**11**
9	Body depressed, sides rounded; pygidium prolonged and subacute; frons convex; protibiae rounded, strongly spinose; meso- and metatibiae very narrow, with few marginal spines	[***Lacrimorpha***]
–	Not fitting the above description	**10**
10	Mesoventrite with deep anterolateral depressions (Fig. 15B)	***P. spectabilis* [24**]
–	Mesoventrite lacking deep anterolateral depressions	***P. notandus* [34**]
11	Pattern of pronotal punctation unique, with most conspicuous punctures across middle of basal portion, grading to smooth anterad (Fig. 19E); most elytral striae complete (somewhat varied), strongly crenulate; body subdepressed, rather strongly tapered at pygidium; protarsi of both sexes with spatulate setae	***P. chabooae* [32**]
–	Not fitting the above description	**12**
12	Pronotal gland openings located ~ 1/6 of pronotal length from anterior margin, at apices of broken anterior marginal stria (Fig. 36G); outer subhumeral stria complete; prosternal keel striae absent (Fig. 36H); dorsal elytral striae 2–5 becoming disconnected series of punctures apically (Fig. 36G)	***P. pirana* [57**]
–	Not fitting the above description, usually with prosternal keel striae and with elytral striae well defined to apices; other characters varied	**13**
13	Dorsum conspicuously punctate; body large (> 4 mm), rarely with marginal pygidial stria	[***Phelister blairi* group] 14**
–	Dorsum typically with only fine ground punctation (only a few exceptions, which have a marginal pygidial stria); body size variable, but usually < 4 mm	[**various *Phelister*, *Operclipygus***]

#### *Phelister blairi* group

**Table:** 

14	Protibial margin appearing scalloped, marginal teeth and spines minute, set deep in marginal incisions (Fig. 2G)	**15**
–	Protibial margin normally toothed; marginal spines conspicuous	**21**
15	Pronotal glands non-annulate (e.g., Figs 2E–F, 27C, 27E); 4^th^ dorsal elytral stria abbreviated from base	**17**
–	Pronotal gland openings usually annulate; 4^th^ dorsal elytral stria arched at base toward sutural stria	**16**
16	Frontal stria usually complete (central portion not detached from lateral portion); pronotum with more conspicuous, enlarged punctures in lateral 1/3 of disk, punctures appearing rather abruptly laterad of the pronotal gland openings; body more elongate (Fig. 27C); pronotal disk not deeply impressed along inner edge of submarginal stria	***P. serratus* [9**]
–	Central portion of frontal stria detached from lateral portion; pronotum with fewer, smaller lateral pronotal punctures; body broader (Fig. 27E); pronotal disk deeply impressed along inner edge of submarginal stria, sub-explanate	***P. geminus* [10**]
17	Lateral submarginal pronotal stria present in less than apical 1/2	**18**
–	Lateral submarginal pronotal stria longer, complete or nearly so	**20**
18	Striae of prosternal keel well-impressed, meeting anteriorly (Fig. 27D)	***P. almeidae* [44**]
–	Striae of prosternal keel week to obsolete, not meeting anteriorly (e.g., Fig. 27F)	**19**
19	Median pronotal gland openings very close to pronotal midpoint (~ 1/2 pronotal length behind anterior pronotal margin) (Fig. 29A)	***P. microdens* [46**]
–	Median pronotal gland openings behind pronotal midpoint, nearly 2/3 pronotal length behind anterior pronotal margin (Fig. 27E)	***P. chicomendesi* [45**]
20	Body smaller, more rufescent; outer subhumeral elytral stria absent; lateral submarginal pronotal stria usually slightly abbreviated from apex	***P. umens* [43**]
–	Body larger, faintly bicolored (Fig. 29C), with the posterior 3/4 darker than most of the rest of the rufescent dorsum; outer subhumeral stria present in apical 1/3; lateral submarginal pronotal stria complete or nearly so	***P. matatlantica* [47**]
21	Pronotal gland openings non-annulate (Fig. 2E, F)	**22**
–	Pronotal gland openings usually annulate (Fig. 2A–C), or gland openings are obscured by dense pronotal punctation, position varied	**30**
22	Fourth dorsal elytral stria not arched to meet sutural stria	**24**
–	Fourth dorsal elytral stria arched to suture	**23**
23	Pronotal ground punctation very fine, sparse, and inconspicuous (Fig. 7A)	***P. globosus* [8**]
–	Pronotal ground punctation very conspicuous (Fig. 9E)	***P. miscellus* [14**]
24	Sutural stria complete, basally hooked (Fig. 36A)	***P. incertus* [54**]
–	Sutural stria not complete	**25**
25	Protibia with inner margin straight, mandibles without strong teeth, at most small blunt teeth at base of incisor edge	**26**
–	Protibia with inner margin inwardly curved (Fig. 31E); both mandibles with strong tooth, that of left mandible weakly bifid	**29**
26	Fourth or 5^th^ elytral stria represented at base by arch or fragments, may be connected to other striae or not	**27**
–	No arches or fragments of striae four or five present near elytral base	**28**
27	Fourth elytral stria reaching base of elytron as a short straight stria or disconnected basal appendix; 5^th^ stria represented by short, disconnected basal arch (Fig. 33C)	***P. semotus* [51**]
–	Base of elytron with longer disconnected arch from base of 4^th^ stria to base of sutural stria (Fig. 33E) (neither of which otherwise reaches the elytral base)	***P. uncinatus* [52**]
28	Third elytral stria complete; 4^th^ and 5^th^ elytral striae present at least in apical 1/3 of elytron	***P. rio* [50**]
–	Third elytral stria interrupted or basally abbreviated, continued to elytral base only by very fine scratch (Fig. 33G); 4^th^ and 5^th^ striae extremely short and apical	***P. inscriptus* [53**]
29	Fourth elytral stria reaching elytral base, sutural stria normally impressed	***P. curvipes* [48**]
–	Fourth elytral stria obsolete in basal 1/3; sutural stria present only as a series of disconnected punctures	***P. vilavelha* [49**]
30	Lateral pronotal margins rather broadly explanate; all tibiae widened; dorsum coarsely punctate	**31**
–	Lateral pronotal margins not explanate, at most narrowly depressed along inner edge of lateral submarginal stria; some tibiae may be somewhat broad, but never with tibiae distinctly expanded; dorsum sculpturing variable; elytral striae only very rarely (and then weakly) carinate	**34**
31	Body convex, elongate; tibiae and femora both broadly expanded (Fig. 21G); several elytral striae carinate, but none forming distinct lateral elytral margin	**32**
–	Body subdepressed, rounded; tibiae but not femora broadly expanded; only inner subhumeral stria carinate, forming lateral elytral margin (Fig. 13A, C)	**33**
32	Pygidium with complete apical marginal stria	***P. amazoniae* [35**]
–	Pygidium lacking marginal stria	***P. arcuatus* [36**]
33	Most dorsal elytral striae very weakly impressed, effaced (Fig. 13C); known only from Costa Rica	***P. marginatus* [21**]
–	All dorsal elytral striae distinct; known only from Bolivia (Fig. 13A)	***P. genieri* [20**]
34	Some part of lateral submarginal pronotal stria present (may be close to margin and carinate, or substantially abbreviated and/or represented by series of punctures)	**42**
–	Lateral submarginal pronotal stria absent	**35**
35	Prosternal keel broad, striae strongly reduced or absent (Fig. 15D); 4^th^ elytral stria present as a basal transverse arch (disconnected from apical portion of stria; Fig. 15C)	***P. pervagatus* [25**]
–	Prosternal striae well developed	**36**
36	Frons and epistoma at least weakly depressed along midline; elytral striae not carinate; frontal stria present at middle (may be detached from sides)	**37**
–	Frons and epistoma not at all depressed; elytral striae weakly carinate (Fig. 17G); frontal stria broadly interrupted across middle	***P. geijskesi* [29**]
37	Pronotum uniformly and conspicuously punctate (Fig. 25C, E)	**38**
–	Pronotum may be densely punctate at sides, but not uniformly across entire disk	**39**
38	Outer subhumeral elytral stria present at base and apex (may be interrupted at middle); prescutellar impression wider, ~ 2 × as wide as scutellum (Fig. 25C)	***P. congruens* [41**]
–	Outer subhumeral elytral stria present in apical 1/2 only; prescutellar impression rather narrow, little wider than scutellum (Fig. 25E)	***P. praesignis* [42**]
39	Prescutellar impression very large, > 6 × scutellum width, anterior margin sinuate (Fig. 19G)	***P. striatinotum* [33**]
–	Prescutellar impression 2–3 × scutellum width, anterior margin broadly or narrowly rounded	**40**
40	Prescutellar impression subtriangular (Fig. 36C); median pronotal gland openings ~ 1/3 behind the anterior margin; left mandible with strong basal tooth	***P. okeefei* [55**]
–	Prescutellar impression broadly oval; median pronotal gland openings ~ 3/4 from anterior margin; left mandible without basal tooth	**41**
41	Labral disk with a large fovea (Fig. 5G); apex of aedeagus broad and flat (Fig. 6J, K)	***P. trigonisternus* [7**]
–	Labrum without a fovea on dorsal surface; apex of aedeagus very narrow and curved ventrad (Fig. 18A, B)	***P. morbidus* [26**]
42	Outer subhumeral elytral stria ‘split’, with two branches in basal 1/2	***P. dilatatus* [23**]
–	Outer subhumeral stria, if present, never split, only present as a single stria throughout its length (which may range from complete to absent)	**43**
43	4^th^ elytral stria with inwardly directed basal arch, reaching or nearly reaching the sutural stria (may or may not be connected to either the 5^th^ or the sutural striae)	**44**
–	4^th^ dorsal without basal arch, either more or less straight at base, or present only at apex of elytra (there may be an arch at the base of the 5^th^ stria)	**62**
44	Median pronotal gland openings 1/5 from the anterior margin; anterior marginal pronotal stria broken slightly, recurved at ends (Figs 3A, 36E)	**45**
–	Median pronotal gland openings 1/3 or more from anterior pronotal margin (or obscured by dense pronotal punctation)	**46**
45	Lateral submarginal pronotal stria abbreviated, present only in apical 1/2 (Fig. 3A); outer subhumeral stria present in apical 1/2 only	***P. blairi* [1**]
–	Lateral submarginal pronotal stria complete (Fig. 36E); outer subhumeral elytral stria present at base and apex, interrupted at middle	***P. blairoides* [56**]
46	Significant portion of dorsal surface conspicuously punctate, pronotum with at least some secondary punctures in middle third (e.g., Figs 11A, 13G)	**47**
–	Dorsal surface with only scattered, minute (typical) surface punctures, larger secondary punctures, if present, confined to lateral thirds of the pronotum	**58**
47	Some portion of inner subhumeral elytral stria present (rarely as series of punctures)	**51**
–	Inner subhumeral elytral stria absent	**48**
48	Prescutellar impression distinct; punctation of elytra finer and sparser than that of pronotum	**49**
–	Prescutellar impression obscured by punctures, indistinct; pronotum and elytra similarly and densely punctate (Fig. 11G)	***P. ifficus* [19**]
49	Sutural stria complete to base; outer subhumeral stria complete	**50**
–	Sutural stria present only in apical 3/4, not reaching base (Fig. 7G) outer subhumeral stria present in apical 1/2 only	***P. parana* [11**]
50	Outer subhumeral stria simple; mandibles untoothed; larger, rounder (Fig. 17E); widespread in South America	***P. sphaericus* [28**]
–	Outer subhumeral stria with a short extension mediad along basal elytral margin; each mandible with strong basal tooth; more elongate (Fig. 9G); only known from Costa Rica	***P. inbio* [15**]
51	Punctures of pronotum single, comprising ground punctation only (which may be dense and conspicuous; Fig. 9C); outer subhumeral stria interrupted at middle, inner present in apical 1/2	***P. uniformis* [13**]
–	Punctures of pronotum ‘double’, comprising fine ground punctation and distinctly larger secondary punctation intermingled (e.g., Figs 9A, 19A); subhumeral striae varied	**52**
52	Pronotal secondary punctures denser in basal 1/2 of pronotum, much sparser anterad (Fig. 19A); all elytral striae complete, connected along apical margin; body large (~ 5 mm), subdepressed	***P. conjunctus* [31**]
–	Pronotal secondary punctures, if conspicuous, not denser basally – may be uniform in density, or be denser in lateral third of disk; elytral striae not connected along apical margin; body size varied, but mostly < 4 mm	**53**
53	Pronotum and elytra similar in density of punctation	**56**
–	Pronotum much more coarsely punctate than elytra	**54**
54	Lateral submarginal pronotal stria present only as a short arc of punctures around anterior margin; body smaller; pronotum very coarsely punctate (Fig. 9A)	***P. asperatus* [12**]
–	Lateral submarginal pronotal stria complete, close to margin; body larger; pronotum only moderately coarsely punctate	**55**
55	Prescutellar impression distinct, broadly oval, ~ 2 × width of scutellum; body more elongate, with sides weakly subparallel (Fig. 17C)	***P. annulatus* [27**]
–	Prescutellar impression indistinct, at most a small vague depression immediately in front of scutellum; body rounded (Fig. 13E)	***P. vazdemelloi* [22**]
56	Elytral striae three and four obliterated apically, stria five absent, and sutural stria barely visible at apex (Fig. 11E)	***P. praedatoris* [18**]
–	Elytral stria all more or less complete; sutural stria reaching elytral base and connected to 4^th^ stria by basal arch	**57**
57	Median pronotal gland openings ~ 2/3 behind pronotal margin (Fig. 11A); frontal stria complete through median depression; frons without microsculpture; antennal club without basal setose patches	***P. sculpturatus* [16**]
–	Median pronotal gland openings nearer halfway back from anterior margin (Fig. 11C); frontal stria obsolete in median depression, frons with distinct microsculpture; antennal club with two distinct dorsal and one ventral setal sensory patch basal to elongate median patch (Fig. 11C)	***P. tunki* [17**]
58	Pronotal glands more than halfway back on pronotal disk; lateral pronotal stria complete	**59**
–	Pronotal glands ~ 1/3 from anterior margin; lateral pronotal stria usually abbreviated posteriorly (Figs 2B, 3C)	***P. erwini* [2**]
59	Apices of inner elytral striae (3^rd^-5^th^ and sutural striae) little more than series of disconnected punctures (Fig. 5A); first abdominal ventrite impunctate along anterior margin (Fig. 5B)	***P. sparsus* [5**]
–	At least sutural stria and usually 5^th^ elytral stria normally impressed; first abdominal ventrite with row of punctures along anterior margin (e.g., Fig. 5D, F)	**60**
60	Outer subhumeral stria interrupted	**61**
–	Outer subhumeral stria complete	***P. stellans* [4**]
61	Eighth sternite of male genitalia with dense apical setal fringe (Fig. 6A); prosternal keel striae enclosing longer, more distinctly triangular space	***P. fimbriatus* [3**]
–	Eighth sternite of male genitalia with only ~ three setae at outer apical corners; prosternal keel striae enclosing rather short, narrow (especially basally) space	***P. pretiosus* [6**]
62	Pronotum with secondary punctures mainly at sides	**64**
–	Pronotum with secondary punctures more uniformly distributed across disk	**63**
63	Pronotum with secondary punctures denser in basal 1/2, smoother anterad (Fig. 19A); complete 5^th^ elytral stria connected to complete sutural stria at base; body large (~ 4 mm), convex	***P. fraternus* [30**]
–	Pronotum uniformly doubly punctate (Fig. 25A)	***P. incongruens* [40**]
64	Anterior marginal pronotal stria entire (Fig. 23A)	***P. gregarius* [37**]
–	Anterior marginal pronotal stria ‘broken’ behind eyes and briefly recurved posterad (Fig. 23C)	**65**
65	Lateral submarginal pronotal stria present only in anterior third; ground punctation of dorsum more conspicuous (Fig. 23E); frons with microsculpture within frontal depression; metaventrite impunctate	***P. rudis* [39**]
–	Lateral submarginal pronotal stria complete; ground punctation of dorsum less conspicuous (Fig. 23C); frons lacking microsculpture; metaventrite usually with secondary punctures anteromediad the hind coxae (Fig. 23D)	***P. praecisus* [42**]

#### *Phelister blairi* subgroup

This diverse subgroup, comprising 25 species, is defined primarily by genitalic characters, which we list below. There are also some external characters that are frequent if not ubiquitous across the group.

Genitalic characters:

- Aedeagus rather broad, moderately flattened, rounded apically, narrowed to base
- Medioventral process of tegmen usually present, non-acute, relatively basally situated (weak or lost in a few species)
- Basal piece with apico-ventral margin acutely produced
- Median lobe elongate (> 1/2 tegmen length), with short, differentiated basal apodemes
- T10 partially to entirely fused (but never apically-only as in amazoniae subgroup)
- Accessory sclerites nearly always present
- S8 apically with conspicuous setae at apical corners
- S9 (spiculum gastrale) generally with strong apical emargination and median keel

External characters

- Antennal club elongate
- Antennal club with basally expanded subapical annulus
- Antennal club with median antennal annulus displaced apicad
- Prosternal keel shelf-like
- Prosternal lobe short
- Prescutellar impression present, round to oval
- Ventral tarsal setae numerous
- Lateral submarginal pronotal stria usually present, often incomplete
- Pronotal bead convex (if lateral submarginal stria present)

##### 1. Phelister blairi

Taxon classificationAnimaliaColeopteraHisteridae

Hinton, 1935

FC0AA521-7281-5E06-816D-647FC9A7179E

**Figure 3.**
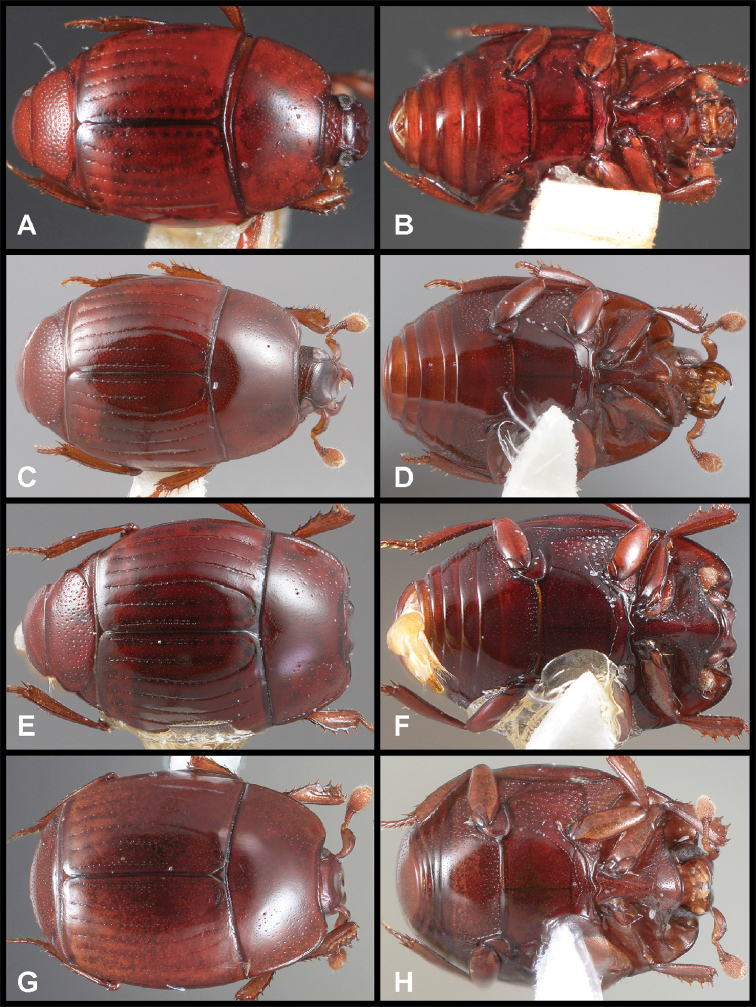
**A, B***Phelister blairi* Lewis: **A** dorsal habitus **B** ventral habitus. **C, D***P. erwini*: **C** dorsal habitus **D** ventral habitus **E, F***P. fimbriatus*: **E** dorsal habitus **F** ventral habitus **G, H***P. stellans*: **G** dorsal habitus **H** ventral habitus.

**Map 1.**
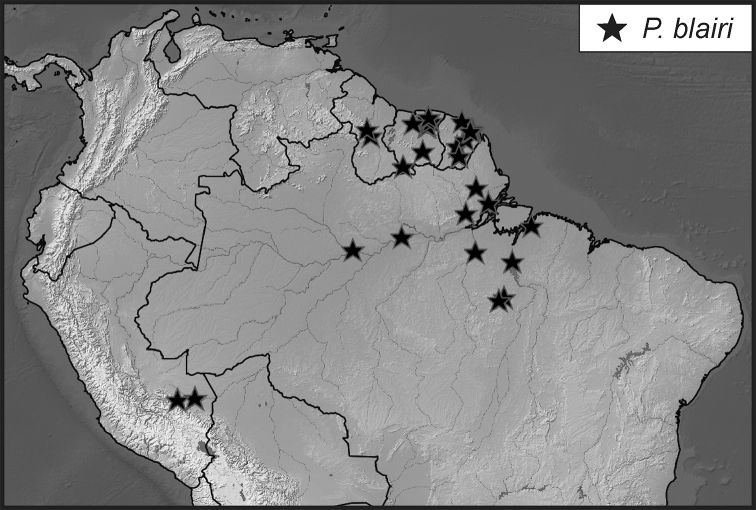
Collecting records for *Phelister blairi*.

**Figure 4.**
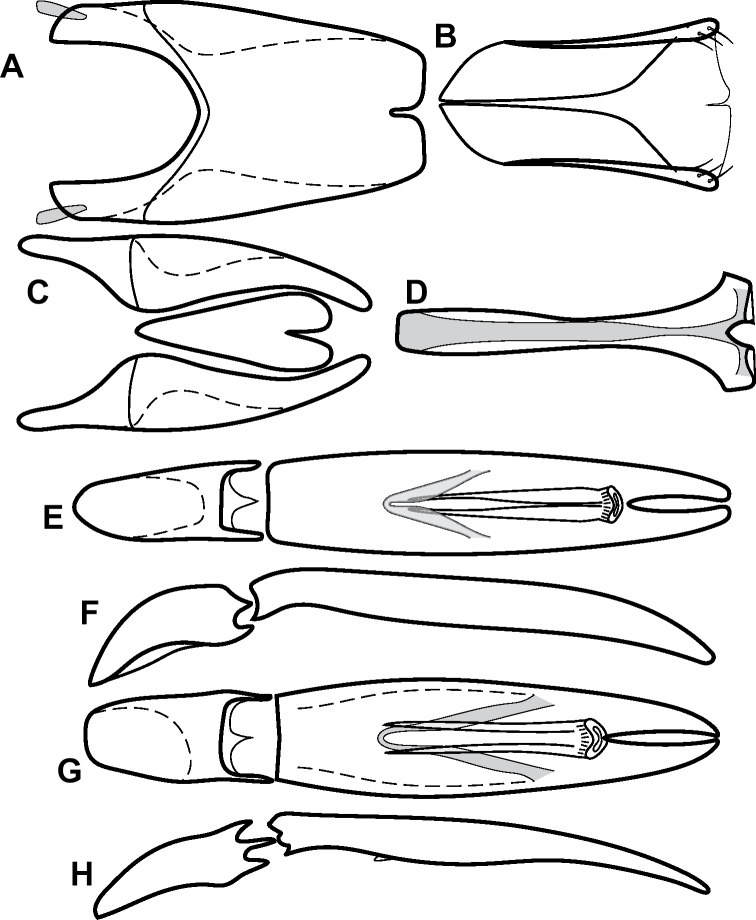
Male genitalia **A–D***Phelister trigonisternus* Hinton: **A** tergite 8 **B** sternite 8 **C** tergites 9 & 10 **D** sternite 9 **E, F***Phelister blairi* Lewis: **E** aedeagus, dorsal **F** aedeagus, lateral **G, H***P. erwini*: **G** aedeagus, dorsal **H** aedeagus, lateral.

Figs 3A, B , 4E, F , Map 1

Phelister blairi Hinton, 1935: 61.

###### Type material.

***Holotype* female**: “Pará, Brazil.” / “June” / “Phelister blairi Type Hntn.” / “G.Lewis Coll. B.M.1926-369”, NHMUK.

###### Other material.

**Brazil**: Amapá, Serra do Navio (0.9833, -52), 1/28/90–2/2/90, FIT (CHND, 3ex.); Amapá, Serra Lombard, Limão (0, -51.2), 8/20/61, EXO-03427 (CHND, 1ex.); Amazonas, Reserva Ducke, 26 km NE Manaus, Plot B (-3, -59.94), February 1995, FIT, M.G.V. Barbosa, EXO-03426 (NHMUK, 1ex.); Pará, Altamira – Marabá: km 18 (-3.15, -52.05), May 1985, FIT, EXO-03372 (CHND, 1ex.); Pará, Belém, Utinga (IPEAN) (-1.45, -48.4333), September 1984, FIT (CHND, 2ex.); Pará, Carajás (Serra Norte) (-6.0667, -50.2), 9/26/86–10/6/86, FIT, EXO-03376 (CHND, 1ex.); Pará, Carajás (Serra Norte) (-6.0667, -50.2), 11/13/87–12/2/87, FIT, EXO-03421 (CHND, 1ex.); Pará, Carajás (Serra Norte) (-6.0667, -50.2), 3/29/89–4/6/89, FIT (CHND, 2ex.); Pará, Carajás (Serra Norte) (-6.0667, -50.2), October 1986, FIT, EXO-03375 (CHND, 1ex.); Pará, Marajó-Breves (-6.26, -50.5333), 11/18/87–12/5/87, FIT (CHND, 5ex.); Pará, Mcpio. Jari, Pacanari (-0.65, -52.6667), 220 m, 3/21/11–4/2/11, Baited pitfalls, C.J. Marsh, EXO-03424 (CHND, 1ex.); Pará, Tucuruí (-3.75, -49.667), 7/10/85–7/29/85, FIT (CHND, 2ex.); Pará, Tucuruí (-3.75, -49.667), June 1985, FIT (CHND, 3ex.); **French Guiana**: Cayenne, 33.5 km S and 8.4 km NW of Hwy N2 on Hwy D5 (4.805, -52.4781), 30 m, 5/29/97–6/9/97, FIT, J. Ashe & R. Brooks, SM0131158 (SEMC, 1ex.); Régina, Rés. Natur. des Nouragues, Camp Inselberg (4.0833, -52.6833), 8/20/10, FIT, SEAG, EXO-03365 (CHND, 1ex.); Régina, Rés. Natur. des Nouragues, Camp Inselberg (4.0833, -52.6833), 10/22/10 and 1/25/11, FIT, SEAG (CHND, 2ex.); Rés. Natur. de la Trinité, (4.6883, -53.2833), 7/20/10, FIT, SEAG (CHND, 1ex.); Roura, 27.4 km SSE (4.7389, -52.2236), 280 m, 5/25/97–5/29/97, FIT, J. Ashe & R. Brooks, SM0096365 (SEMC, 1ex.); Saül, 7 km N, 1 km NW Les Eaux Claires, along Route de Bélizon trail (3.6628, -53.2219), 280 m, 6/4/97–6/8/97, FIT, J. Ashe & R. Brooks, SM0133772 (SEMC, 1ex.); Belvédère de Saül, point de vue (3.6228, -53.2094), 2/14/11, FIT, SEAG (CHND, 1ex.); Rés. Trésor (route de Kaw Pk18) (4.6105, -52.279), 225 m, 11/21/09, FIT, SEAG, EXO-03360 (CHND, 1ex.); Mont Tabulaire, Itoupé (3.022, -53.0842), 800 m, 3/17/10, FIT, SEAG (CHND, 5ex.); Mont Tabulaire, Itoupé (3.023, -53.0955), 570 m, 3/24/10, FIT, SEAG (CHND, 2ex.); Mont Tabulaire Itoupé (3.0303, -53.1067), 400 m, 3/17/10, FIT, SEAG, EXO-03362 (CHND, 1ex.); Mont Tabulaire Itoupé (3.023, -53.0955), 570 m, 3/17/10, FIT, SEAG, EXO-03361 (CHND, 1ex.); Montagne des Chevaux (4.7167, -52.4), 12/6/08–12/16/08, 1/10/09–1/18/09, 3/15/09, 4/11/09 and 1/31/11, FIT, SEAG (AKTC & CHND, 10ex.); Paracou (5.27, -52.92), November 1996, FIT, P.M. Hammond, EXO-03358 (NHMUK, 1ex.); Route Nac. 2, P.k. 47 (4.6333, -52.3667), 7/11/78, Piège + cad. de serpent (CHND, 2ex.); **Guyana**: Region 8, Iwokrama Forest, 26 km SW Kurupukari, Iwokrama Mt. (4.3339, -58.7883), 400 m, 5/23/01–5/25/01, FIT, R. Brooks, Z. Falin (SEMC, 2ex.); Region 8, Iwokrama Forest, 26 km SW Kurupukari, Iwokrama Mt. (4.3339, -58.7883), 600 m, 5/23/01–5/25/01, FIT, R. Brooks, Z. Falin, SM0565566 (SEMC, 1ex.); Region 8, Iwokrama Forest, Pakatau hills (4.7483, -59.0267), 70 m, 5/25/01–5/29/01, FIT, R. Brooks, Z. Falin, SM0569456 (SEMC, 1ex.); **Peru**: Cusco, Villa Carmen Fld Stn., 12.89250°S, 71.41917°W, 555 m, 28–30.V.2011, DJ Bennett, FIT (SEMC, 1ex.); Madre de Dios, Los Amigos Field Station, Manu, CICRA (-12.5624, -70.093), 288 m, 7/31/06–8/9/06, pitfall trap, terra firma forest, J. Jacobs, CASENT8123338 (CASC, 1ex.); Madre de Dios, Los Amigos Field Station, Manu, Maderero bamboo forest (-12.5458, -70.1149), 283 m, 1/2/07–1/11/07, pitfall trap, bamboo forest, J. Jacobs, CASENT 8123423 (CASC, 1ex.); Madre de Dios, Pantiacolla Lodge, Alto Madre de Dios R. (-12.655, -71.2317), 420 m, 11/14/07–11/19/07, FIT, D. Brzoska (SEMC, 9ex.); Madre de Dios, Pantiacolla Lodge, Alto Madre de Dios River (-12.6561, -71.2319), 400 m, 10/23/00–10/26/00, FIT, R. Brooks (SEMC, 3ex.); Madre de Dios, Rio Alto Madre de Dios, Pantiacolla Lodge (-12.655, -71.2317), 11/14/07–11/19/07, FIT, D. Brzoska (SEMC, 4ex.); Madre de Dios, CICRA Field Stn., ~ 2 km NW cafeteria res. plot, 12.55236°S, 70.10989°W, 295 m, Flight intrcept. 7–9.vi.2011, Chaboo Team (SEMC 2ex.); **Suriname**: Brokopondo, Brownsberg Nature Preserve, Witi Creek Trail (4.9486, -55.1814), 340 m, 6/23/99–6/25/99, FIT, Z. Falin, A. Gangadin, H. Hiwat, SM0166833 (SEMC, 1ex.); Brokopondo, Ston Eiland Eco Resort nr. Brownsberg (4.9833, -55.1333), 2/10/10–1/13/10, FIT, C. Gillet, P. Skelley, W. Warner (FSCA, 3ex.); Commewijne, Akintosoela, 32 km SE Suriname River Bridge, road to Redi Doti (5.2714, -54.9208), 40 m, 6/29/99–7/3/99, FIT, Z. Falin, B. DeDijn, A. Gangadin, SM0176672 (SEMC, 1ex.); Marowijne, Palumeu (3.3489, -55.4383), 160 m, 7/9/99, FIT, Z. Falin (CMNC, 2ex.); Marowijne, Palumeu, (3.3489, -55.4383), 160 m, 7/7/99–7/8/99, FIT, Z. Falin, SM0176368 (SEMC, 1ex.); Pará, Carolina Creek, 11 km SE Zanderij Airport (5.3933, -55.1581), 30 m, 6/19/99–6/20/99, FIT, Z. Falin, A. Gangadin, SM0165187 (SEMC, 1ex.); Pará, nr. Overbridge River Resort (5.53, -55.058), 2/15/10–2/18/99, FIT, C. Gillet, P. Skelley, W. Warner (FSCA, 2ex.); Pará, nr. Overbridge at 5.5195, -55.0695, 2/10/10–2/14/10, FIT, W.B. Warner (AKTC & WBWC, 15ex.); Saramacca, West Suriname Road, 139 km WSW Zanderij Airport (5.15, -56.0667), 40–50 m, 6/10/99–6/14/99, FIT, Z. Falin, B. DeDijn, SM0178423 (SEMC, 1ex.); Sipaliwini, CI-RAP Surv. Camp 1: on Kutari River (-2.1753, -56.7874), 228 m, 8/19/10–8/24/10, FIT, Larsen & Short (SEMC, 6ex.); Sipaliwini, CI-RAP Surv. Camp 2: Sipaliwini River (-2.1753, -56.7874), 210 m, 8/27/10–9/1/10, FIT, Larsen & Short, EXO-03369 (SEMC, 4ex.); Sipaliwini, CI-RAP Surv. Camp 3: Wehepai SE Kwamala (2.3629, -56.6977), 237 m, 9/3/10–9/7/10, FIT, Larsen & Short (SEMC, 7ex.); Sipaliwini, CI-RAP Surv. Camp 1: upper Palumeu River (2.4770°N, 55.6294°W), 275 m, FIT, 3/10/12–3/16/12, A.E.Z. Short (AKTC & SEMC, 13ex.); Sipaliwini, CI-RAP Surv. Camp 4: on lower Kasikasima River (2.9773, -55.3850), 200 m, 3/20/12–3/25/12, FIT, T. Larsen (AKTC & SEMC, 6ex.).

###### Diagnostic description.

Length: 1.50–1.65 mm (avg. 1.62 mm); width: 1.22–1.42 mm (avg. 1.36 mm). Body small, elongate oval, convex, rufescent, with ground punctation moderately conspicuous; frons and epistoma somewhat narrow, deeply depressed along midline, sparsely but conspicuously punctate; supraorbital stria present, narrowly separated from sides of frontal stria, which is complete across depression; epistoma raised at sides; labrum emarginate and subcarinate apically; mandibles lacking conspicuous basal teeth; antennal club elongate with conspicuous setose patch on dorsal surface; prescutellar impression of pronotum ovoid to weakly acuminate at front, approximately equal in width to scutellum; pronotal disk with conspicuous secondary punctures in lateral thirds, more densely to front; median pronotal gland openings distinctly annulate, ~ 1/5 behind anterior margin; marginal pronotal stria complete laterally, broken with inner ends recurved briefly behind eyes; submarginal pronotal stria in apical 1/2 only, weakly impressed, crenulate, just turning anterior corner; elytron with single, complete epipleural stria; outer subhumeral stria present in apical one-third only, inner absent; dorsal striae 1–4 complete, 4^th^ arched to sutural, 5^th^ present in apical 1/2, sutural stria present in apical 2/3; propygidium with dense secondary punctures, those of pygidium smaller and sparser; prosternal keel emarginate at base, striae united along base, enclosing a triangle ~ 3/4 length of keel, with secondary striae along basal 1/2; prosternal lobe short, rounded, with complete marginal stria; mesoventrite produced, with complete marginal stria continued at sides by postmesocoxal stria, which ends freely at middle of lateral portion of metaventrite; mesometaventral stria angulate at middle, reaching middle of mesoventrite, continued at sides by lateral metaventral stria to middle of metacoxa; middle portion of metaventrite impunctate; 1^st^ abdominal ventrite with secondary punctures along anterior margin, with incomplete lateral stria along inner margin of metacoxa; protibia with outer margin rounded, weakly dentate, with five or six marginal spines; meso- and metatibiae slender, mesotibia with rather robust marginal spines, those of metatibia fine and restricted to apical one-third. Male: basal piece ~ 1/3 length of tegmen; tegmen with sides evenly rounded, apices slightly separated, weak medioventral process present in basal 1/3; median lobe ~ 1/2 tegmen length, basal apodemes thin.

###### Distribution.

This species is most commonly found in northeastern South America, including Pará, Brazil, French Guiana, and Suriname. We have also seen specimens from Peru, but surprisingly not Ecuador.

###### Remarks.

Among the species treated here, *P. blairi* is best recognized by its small body size, median pronotal gland openings located close to anterior pronotal margin (Fig. 3A), broken anterior marginal pronotal stria, and posteriorly obsolete lateral submarginal pronotal stria. It also has a relatively small area (shortened) enclosed by its prosternal keel striae. *Phelister blairoides* (#56, below) has similarly anterior pronotal gland openings, but has the lateral submarginal pronotal stria complete, and has the outer subhumeral elytral stria present basally as well as apically (though interrupted at middle). *Phelister blairoides* is also ~ 2 × as large. Although both these species are widespread and somewhat variable across their ranges, these differences are consistent. This species is also very similar to the following, *P. erwini*, differing principally by the more anteriorly located pronotal gland openings.

Most specimens have been collected by flight interception traps, with a few taken in carrion and dung baited pitfalls.

##### 2. Phelister erwini sp. nov.

Taxon classificationAnimaliaColeopteraHisteridae

EBC5EBA9-1834-5385-AA67-9D8DBD534E4C

http://zoobank.org/E1D62B16-0AED-4761-A124-F709E8B17D77

**Map 2.**
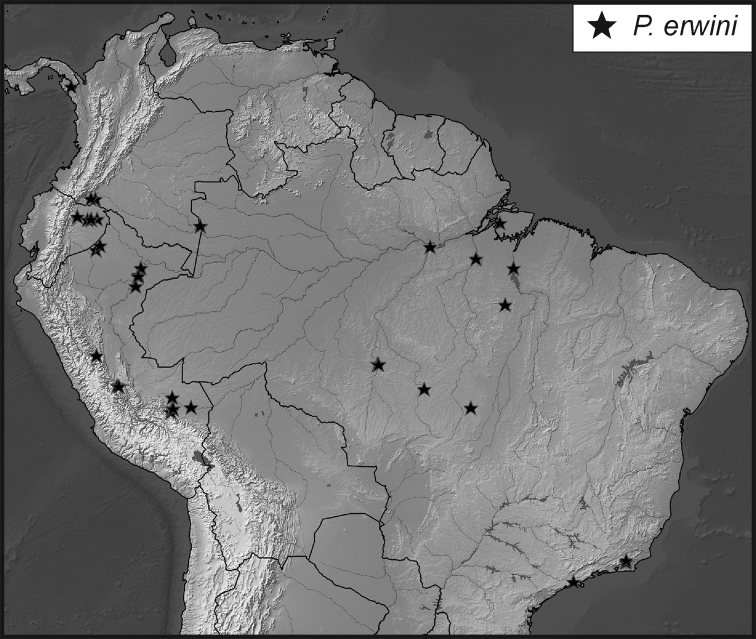
Collecting records for *Phelister erwini*.

Figs 2B , 3C, D , 4G, H , Map 2

###### Type material.

***Holotype* male**: “**Ecuador**: Napo, mid.Rio Tiputini, Yasuní Res. Stn. 0°40.5'S, 76°24'W, FIT#3. 18–23 June 1999 AKT#016 A.Tishechkin” / “LSAM 0045157” / “Phelister sp. #7. Hist 148 Yasuní NP Inventory A.K. Tishechkin det. 2010” (FMNH). ***Paratypes* (191): Ecuador**: Orellana, Est. Biodiv. Tiputini (-0.6333, -76.0383), 220 m, 9/5/00–9/25/00, D. Inward & K. Jackson (NHMUK, 8ex.); Orellana, Est. Biodiv. Tiputini (-0.6376, -76.1499), 6/2/11–6/9/11, FIT, M. Caterino & A. Tishechkin (MSCC, 28ex.); Orellana, Est. Biodiv. Tiputini (-0.6376, -76.1499), 6/9/11, Dead *Ec. hamatum* bait, M. Caterino & A. Tishechkin, EXO-00608 (MSCC, 1ex.); Orellana, Est. Cientifica Yasuní, mid. Rio Tiputini (-0.675, -76.4), 6/17/99–8/4/99, FIT, C. Carlton & A. Tishechkin (LSAM, 95ex.); Orellana, Est. Cientifica Yasuní, mid. Rio Tiputini (-0.675, -76.4), 7/18/99, *Eciton burchelli* colony EC#21, Statary bivouac site just after emigration, A. Tishechkin (LSAM, 3ex.); Orellana, Est. Cientifica Yasuní, mid. Rio Tiputini (-0.675, -76.4), 7/22/99, *Eciton burchelli* colony EC#23, nomadic bivouac site just after emigration, A. Tishechkin, LSAM 0045288 (LSAM, 1ex.); Orellana, Est. Cientifica Yasuní, mid. Rio Tiputini (-0.675, -76.4), 7/29/99, *Eciton burchelli* colony EC#27, refuse deposit statary phase, A. Tishechkin, LSAM 0045289 (LSAM, 1ex.); Orellana, Est. Cientifica Yasuní (-0.6744, -76.6472), 215 m, 9/5/99–9/10/99, FIT, primary forest, E. Riley (LSAM & TAMU, 49ex.); Orellana, Payamino Research Station (-0.4933, -77.2914), 300 m, 7/30/07–8/12/07, FIT, Tropical Rainforest, CPDT Gillett (NHMUK, 2ex.); Orellana, Res. Ethnica Waorani, 1 km S Onkone Gare Camp, Trans. Ent. (0.6528, -76.4333), 220 m, 1/24/94, Insecticidal fogging, mostly bare green leaves, some with covering of lichenous or bryophytic plants in terra firme forest, T.L. Erwin, EXO-03445 (USNM, 1ex.); Orellana, Res. Ethnica Waorani, 1 km S Onkone Gare Camp, Trans. Ent. (-0.6528, -76.4333), 220 m, 10/7/94, Insecticidal fogging, mostly bare green leaves, some with covering of lichenous or bryophytic plants in terra firme forest, T.L. Erwin, EXO-03446 (USNM, 1ex.); Orellana, nr. Tributary R. Tiputini, 6/25/96–6/30/96, FIT, A. Cognato, EXO-03443 (MSCC, 1ex.).

###### Other material.

**Brazil**: Mato Grosso, Mpio. Claudia (-11.4083, -55.325), 10/17/10–10/27/10, FIT, A.F. Oliveira (CEMT, 3ex.); Mato Grosso, Mpio. Cotriguaçu, Fazenda São Nicolau (-9.815, -58.2858), 12/15/10–12/18/10, FIT, F.Z. Vaz-de-Mello & A.F. Oliveira (CEMT, 4ex.); Mato Grosso, Mpio. Cotriguaçu, Fazenda São Nicolau, Mata Norte (-9.8192, -58.26), 12/8/10–12/14/10, FIT, F.Z. Vaz-de-Mello (CEMT, 6ex.); Mato Grosso, Mpio. Cotriguaçu, Fazenda São Nicolau, Matinha (-9.8383, -58.2508), 4/3/09, FIT, F.Z. Vaz-de-Mello (CEMT, 10ex.); Mato Grosso, Mpio. Cotriguaçu, Fazenda São Nicolau, Matinha (-9.8383, -58.2508), October 2009, FIT, F.Z. Vaz-de-Mello (CEMT, 8ex.); Mato Grosso, Mpio. Cotriguaçu, Fazenda São Nicolau, Prainha (-9.86, -58.215), September 2009, FIT, R.V. Nunes, EXO-03456 (CEMT, 1ex.); Mato Grosso, Mpio. Querencia, Fazenda São Luiz (-12.597, -52.3749), February 2009, FIT, R. Andrade (CEMT, 4ex.); Pará, Altamira – Marabá: km 18 (-3.15, -52.05), May 1985, FIT (CHND, 3ex.); Pará, Carajás (Serra Norte) (-6.0667, -50.2), 3/29/89–4/6/89, FIT (CHND, 2ex.); Pará, Ilha Arapiuns (-2.4, -54.95), 12/30/08–12/31/08, FIT (CHND, 3ex.); Pará, Marajó-Breves (-0.8833, -50.5333), 11/18/87–12/5/87, FIT, EXO-03463 (CHND, 1ex.); Pará, Tucuruí (-3.75, -49.667), June 1985, FIT, EXO-03462 (CHND, 1ex.); Rio de Janeiro, 17 km E Nova Friburgo (-22.3844, -42.5583), 750 m, 1/21/00, FIT, secondary mount. Atlantic for., F. Génier & S. Ide (CMNC, 3ex.); Rio de Janeiro, 17 km E Nova Friburgo (-22.3844, -42.5583), 750 m, 1/23/00, FIT, secondary mount. Atlantic for., F. Génier & S. Ide, EXO-03451 (CMNC, 1ex.); Rio de Janeiro, Nova Friburgo (-22.2667, -42.5333), 10/26/09–10/31/09, FIT (CHND, 13ex.); São Paulo, Boracéia Biol. Station, nr. Salesópolis (-23.6333, -45.8667), 11/23/08–11/30/08, FIT, P.C. Grossi, EXO-03453 (CHND, 1ex.); **Colombia**: Vaupes, Est. Biol. Caparu, Rio Apoporis (-1.1, -69.5), 9/27/95–12/1/95, FIT, Black-water terrace forest on sandy soils, B. Gill (AKTC, 56ex.); Vaupes, Parco Nac. Mosiro-Itajura (Caparu) Centro Ambiental (-1.0667, -69.5167), 60 m, 1/20/03–1/30/03, FIT, D. Arias & M. Sharkey (AKTC, 55ex.); **Ecuador**: Orellana, Est. Biodiv. Tiputini (-0.64, -76.15), 7/27/08–8/3/08, FIT, A.K.Tishechkin (AKTC & LSAM, 114ex.); Orellana, Est. Biodiv. Tiputini (-0.64, -76.15), 8/3/08–8/6/08, FIT, LSAM Team (LSAM, 21ex.); Orellana, Est. Cientifica Yasuní (-0.675, -76.4), 7/10/08–7/26/08, FIT, A. Tishechkin (AKTC & LSAM, 228ex.); **Panama**: Darien, Cana Biological Station, Serrania de Pirre (7.755, -77.685), 1200 m, 6/4/96–6/9/96, FIT, J. Ashe & R. Brooks (SEMC, 10ex.); **Peru**: Cusco, Villa Carmen Fld Stn., 12.89221°S, 71.41946°W, 555 m, 20–30.V.2011, DJ Bennett, FIT (SEMC, 22 ex.); Cusco, Villa Carmen Fld Stn., 12.87753°S, 71.40153°W, 555–1000 m, 20–30.V.2011, DJ Bennett, misc. hand collecting (SEMC, 8ex.); Cusco, Villa Carmen Fld Stn., 12.89250°S, 71.41917°W, 555 m, 26–28.V.2011, DJ Bennett, FIT (SEMC, 1ex.); Huanuco, Tingo Maria-Monzon Rd. S of Agua Blanca (-9.2917, -76.08), 1000 m, 10/12/99–10/13/99, FIT, R. Brooks, SM0144307 (SEMC, 1ex.); Junín, ~ 16 km NW Satipo, Rio Venado (-11.1989, -74.7705), 1110 m, 2/20/10–2/22/10, A.V. Petrov, EXO-03486 (AKTC, 1ex.); Junín, Sector San Isidro (-11.2418, -74.6663), 730 m, 4/12/09–4/13/09, FIT, treefall, A.V. Petrov, EXO-03485 (AKTC, 1ex.); Junín, 11 km NE Puerto Ocopa, Los Olivos (-11.05, -74.2587), 1200 m, 3/25/09–4/2/09, FIT, A. Tishechkin (AKTC, 36ex.); Junín, ~ 15 km NW Satipo, Rio Venado at 11°11.71'S, 74°46.12'W, 1150 m, window trap, 20–24.v.2012, A. V. Petrov (AKTC, 2ex.); Junín, ~ 15 km NW Satipo, Rio Venado (-11.1979, -74.77), 1100 m, 2/20/13, window trap, A.V. Petrov (AKTC, 4ex.); Junín, Rio Venado at 11°11'40"S, 74°46'8"W, 1195 m, window trap, 26.i.2019, A. V. Petrov (AKTC, 2ex.); Loreto, 1.5 km N Teniente Lopez (-2.5943, -76.1153), 210–240 m, 7/18/93–7/26/93, FIT, R. Leschen (SEMC, 34ex.); Loreto, 1.5 km N Teniente Lopez (-2.5943, -76.1153), 210–240 m, 7/18/93, FIT, R. Leschen (SEMC, 4ex.); Loreto, 68 km SW Iquitos to Nauta, Rio Itaya (-4.1833, -73.4167), 110 m, 2/26/08–2/30/2008, A.V. Petrov, EXO-03482 (AKTC, 1ex.); Loreto, Campamento San Jacinto (-2.3125, -75.8628), 175–215 m, 7/11/93, FIT, R. Leschen, EXO-03467 (SEMC, 1ex.); Loreto, Iquitos (-3.74, -73.27), 90 m, 5/5/92, FIT, J. Danoff-Berg (SEMC, 3ex.); Loreto, Iquitos – Nauta rd., km 58, Rio Itaya (-4.2563, -73.4675), 120 m, 5/5/09–5/9/09, FIT, entrance into *Eciton burchelli* statary bivouac in a hollow treee, A.V. Petrov (AKTC, 8ex.); Loreto, Iquitos, Jenero Herbera (-4.9, -73.6), October-November 1988, FIT, G. Couturier, EXO-03479 (CHND, 1ex.); Loreto, km 63, rd. Iquitos – Nauta, Rio Itaya (-4.2534, -73.4346), 140 m, 1/9/11–1/13/11, A.V. Petrov, EXO-03483 (AKTC, 1ex.); Madre de Dios, Manu, Los Amigos Field Station (-12.5624, -70.093), 288 m, 7/31/06–8/9/06, pitfall trap, CICRA terra firma forest, J. Jacobs, CASENT 8123337 (CASC, 1ex.); Madre de Dios, Pakitza Bio. Stn., Reserved Zone, Manu National Park, Castanal Trail (-11.9447, -71.2833), 317 m, 10/15/00–10/16/00, FIT, R. Brooks, SM0271786 (SEMC, 1ex.); Madre de Dios, Amazonas Lodge, N Atalaya (-12.87, -71.3767), 480 m, 11/10/07–11/13/07, FIT, D. Brzoska, SEMC0870245 (SEMC, 1ex.); Madre de Dios, Pantiacolla Lodge, 2–7 km NW El Mirador Trail, Alto Madre de Dios River (-12.6528, -71.2578), 450–700 m, 10/23/00–10/26/00, FIT, R. Brooks (SEMC, 8ex.); Madre de Dios, Pantiacolla Lodge, 8 km NW El Mirador Trail, Alto Madre de Dios River (-12.6417, -71.2781), 800 m, 10/23/00–10/26/00, FIT, R. Brooks (SEMC, 9ex.); Madre de Dios, Pantiacolla Lodge, Alto Madre de Dios R. (-12.655, -71.2317), 420 m, 11/14/07–11/19/07, FIT, D. Brzoska (SEMC, 36ex.); Madre de Dios, Pantiacolla Lodge, Alto Madre de Dios River (-12.6561, -71.2319), 400 m, 10/23/00–10/26/00, FIT, R. Brooks (SEMC, 3ex.).

###### Diagnostic description.

Length: 1.54–1.81 mm (avg. 1.63 mm); width: 1.30–1.58 mm (avg. 1.40 mm). This species is extremely similar to *P. blairi*, but differs consistently in a few characters: pronotal gland openings at least 1/4 removed from the anterior pronotal margin, more typically ~ 1/3 removed; anterior portion of marginal pronotal stria rarely broken, generally subangulate behind eye, but without recurved ends; lateral submarginal pronotal stria slightly more deeply depressed. Male: basal piece ~ 1/3 length of tegmen; tegmen rather flat, with sides rounded, widest in basal 1/3, apices slightly separated, medioventral process long, projecting in basal 1/3; median lobe ~ 1/2 tegmen length, basal apodemes evenly narrowed to tips.

###### Etymology.

We dedicate this species to the renowned coleopterist, Dr. Terry Erwin, who unfortunately passed away early in 2020. His groundbreaking surveys of neotropical beetles, outstanding contributions to ground beetle systematics, and important contributions to the cause of insect biodiversity conservation have been a great inspiration to us, and many, many others.

###### Remarks.

This species shows strong similarity to *P. blairi*, above; their almost completely non-overlapping distributions suggest that the two are sister taxa (and they are resolved as such in our analyses). The pronotal gland openings in *P. erwini* are nearly always ~ 1/3 removed from the anterior pronotal margin, whereas those of *P. blairi* are much closer to the anterior margin (see Fig. 3C and 3A, respectively). The two consistently differ near *P. blairi*’s type locality (Pará, Brazil), as well at another point of sympatry (southeastern Peru), but some variation in gland opening location and sculpturing (pronotal and pygidial) make the two difficult to distinguish throughout their ranges and in all specimens.

A number of specimens of *P. erwini* have been collected in loose association with neotropical army ants (*Eciton* spp.), suggesting some degree of myrmecophily. However, the vast majority of specimens of this abundant species have been collected with flight interception traps, so other possible associations cannot be assessed.

###### Distribution.

This is an abundant and widely distributed species, known from Panama, Amazonian regions of Colombia, Ecuador, Peru, and Brazil, as well as southeastern coastal Brazil.

**Figure 5.**
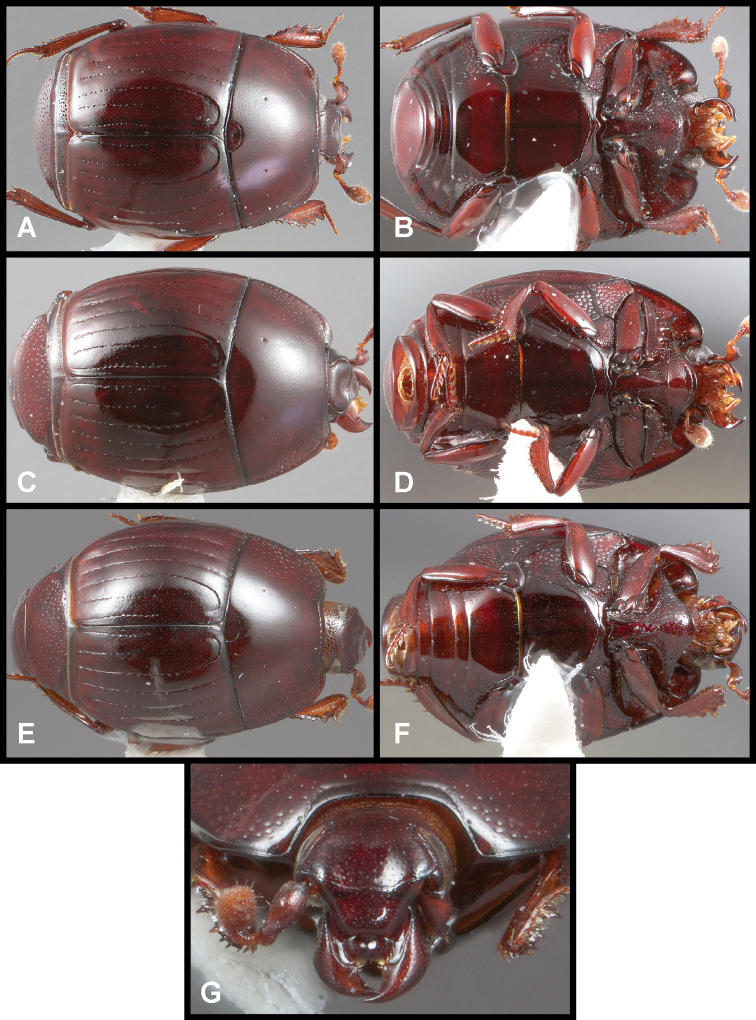
**A, B***Phelister sparsus*: **A** dorsal habitus **B** ventral habitus **C, D***P. pretiosus*: **C** dorsal habitus **D** ventral habitus **E–G***P. trigonisternus*: **E** dorsal habitus **F** ventral habitus **G** frons and labrum showing labral fovea.

##### 3. Phelister fimbriatus sp. nov.

Taxon classificationAnimaliaColeopteraHisteridae

43D17C39-9F94-5742-A07C-D2EF26EF5A67

http://zoobank.org/2A6CB680-62C2-403C-B923-A1D7972C47AC

**Figure 6.**
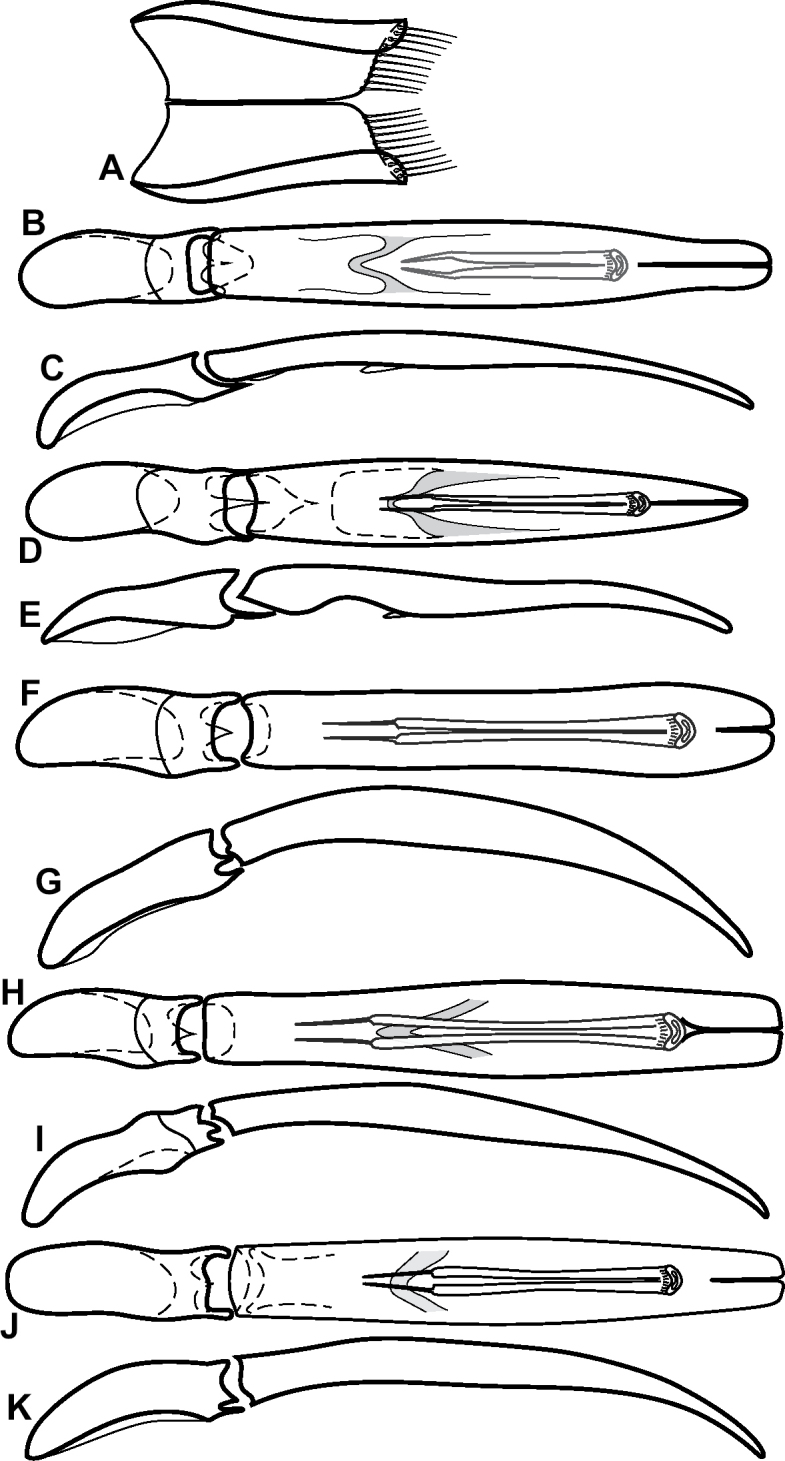
Male genitalia **A–C***Phelister fimbriatus*: **A** sternite 8 **B** aedeagus, dorsal **C** aedeagus, lateral **D, E***P. stellans*: **D** aedeagus, dorsal **E** aedeagus, lateral **F, G***P. sparsus*: **F** aedeagus, dorsal **G** aedeagus, lateral **H, I***P. pretiosus*: **H** Aedeagus, dorsal **I** Aedeagus, lateral **J, K***P. trigonisternus*: **J** aedeagus, dorsal **K** aedeagus, lateral.

**Map 3.**
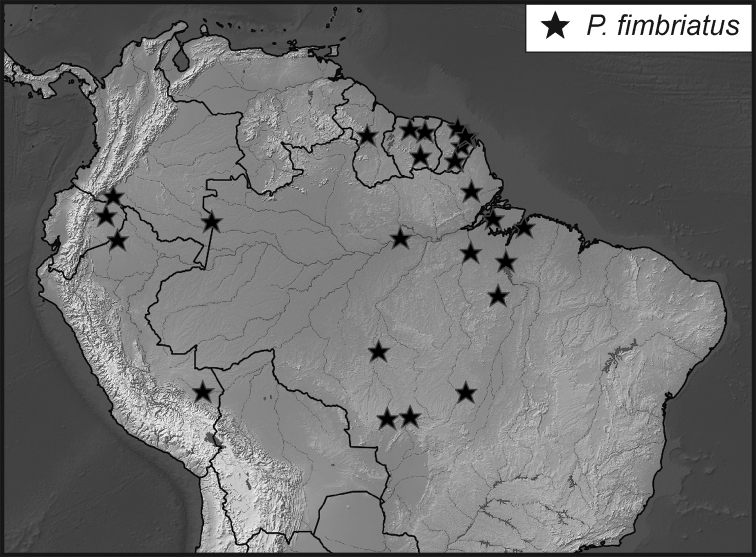
Collecting records for *Phelister fimbriatus*.

Figs 3E, F , 6A–C , Map 3

###### Type material.

***Holotype* male**: “**French Guiana** Roura (8.4km SSE), 200 m 4°40'41"N, 52°13'25"W, 29.v–10.vi.1997, J.Ashe & R.Brooks, FG1AB97 #182, ex: flight int. trap” / “Caterino/Tishechkin Exosternini Voucher EXO-02927” (CMNC). ***Paratypes* (17 – confirmed males only): French Guiana**: Belvédère de Saül, point de vue (3.6228, -53.2094), 1/31/11 FIT, SEAG (CHND, 1ex.); Belvédère de Saül, point de vue (3.6228, -53.2094), 2/7/11, FIT, SEAG (CHND, 1ex.); Régina, Rés. Natur. des Nouragues (4.0378, -52.6725), 11/3/09, FIT, SEAG (CHND, 1ex.); Régina, Rés. Natur. des Nouragues, Camp Inselberg (4.0833, -52.6833), 9/9/10, 9/22/10, FIT, SEAG (AKTC & CHND, 3ex.); Régina, Rés. Natur. des Nouragues, Camp Inselberg (4.0833, -52.6833), 1/25/11, FIT, SEAG (AKTC & CHND, 2ex.); Montagne des Chevaux (4.7167, -52.4), 12/16/08, FIT, SEAG (AKTC, 1ex.); Montagne des Chevaux (4.7167, -52.4), 12/6/08–12/16/08, 4/11/09, FIT, SEAG (AKTC & CHND, 2ex.); Montagne des Chevaux (4.7167, -52.4), 2/23/09, FIT, SEAG (CHND, 1ex.); **Suriname**: Brokopondo, Ston Eiland Eco Resort nr. Brownsberg (4.9833, -55.1333), 2/10/10–2/13/10, FIT, C. Gillet, P. Skelley, W. Warner, EXO-02929 (FSCA, 1ex.); Sipaliwini, CI-RAP Surv. Camp 2: Sipaliwini River (-2.1753, -56.7874), 210 m, 8/27/10–9/1/10, FIT, Larsen & Short, EXO-02928 (SEMC, 1ex.); **Brazil**: Amapá, Serra do Navio (0.9833, -52), 5/1/91–5/14/91, FIT, EXO-02935 (CHND, 1ex.); Pará, Altamira – Marabá: km 18 (-3.15, -52.05), May 1985, FIT (CHND, 1ex.); Pará, Marajó-Breves (-0.8833, -50.5333), 11/18/87–12/5/87, FIT (CHND, 1ex.).

###### Other material.

**French Guiana**: Belvédère de Saül, point de vue (3.6228, -53.2094), 1/17/11, 1/31/11 and 2/7/11, FIT, SEAG (CHND, 2ex.); Paracou (5.27, -52.92), November 1996, FIT, P.M. Hammond, EXO-02926 (NHMUK, 1ex.); Régina, Rés. Natur. des Nouragues (4.0378, -52.6725), 11/3/09, FIT, SEAG (CHND, 1ex.); Régina, Rés. Natur. des Nouragues (4.0378, -52.6725), 1/28/10, FIT, SEAG (CHND, 3ex.); Régina, Rés. Natur. des Nouragues (4.0378, -52.6725), 2/19/10, FIT, SEAG, EXO-02920 (CHND, 1ex.); Rés. Trésor (route de Kaw Pk18) (4.6105, -52.279), 225 m, 11/6/09, FIT, SEAG (CHND, 2ex.); Mont Tabulaire, Itoupé (3.023, -53.0955), 570 m, 3/24/10, FIT, SEAG, EXO-02923 (CHND, 1ex.); Mont Tabulaire Itoupé (3.0303, -53.1067), 400 m, 3/17/10, FIT, SEAG (CHND, 5ex.); Montagne des Chevaux (4.7167, -52.4), 8/9/09, FIT, SEAG, EXO-02925 (CHND, 1ex.); Montagne des Chevaux (4.7167, -52.4), 5/22/10, FIT, SEAG, EXO-02945 (CHND, 1ex.); **Brazil**: Mato Grosso, Mpio. Cotriguaçu, Fazenda São Nicolau, Mata Norte (-9.8192, -58.26), 12/8/10–12/14/10, FIT, F.Z. Vaz-de-Mello (CEMT, 2ex.); Mato Grosso, Mpio. Querencia, Fazenda São Luiz (-12.597, -52.3749), February 2009, FIT, R. Andrade, EXO-02932 (CEMT, 1ex.); Mato Grosso, Mpio. Tangara da Serra (-14.3617, -57.6717), 580 m, 1/17/10–2/3/10, FIT, R.S. Silva, EXO-02933 (CEMT, 1ex.); Mato Grosso, Mun. Diamantino. Faz. São João (-14.2361, -56.1364), 400 m, 1/11/01, FIT, primary gallery for., Génier & Vaz-de-Mello, EXO-00364 (CMNC, 1ex.); Pará, Altamira – Marabá: km 18 (-3.15, -52.05), May 1985, FIT (CHND, 3ex.); Pará, Belém, Utinga (IPEAN) (-1.45, -48.4333), October, 1984, FIT, EXO-02936 (CHND, 1ex.); Pará, Carajás (Serra Norte) (-6.0667, -50.2), November 1984, FIT, EXO-02937 (CHND, 1ex.); Pará, Marajó-Breves (-0.8833, -50.5333), 11/18/87–12/5/87, FIT (CHND, 2ex.); Pará, Tucuruí (-3.75, -49.667), 12/9/85–12/17/85, FIT (CHND, 4ex.); Pará, Tucuruí (-3.75, -49.667), 11/28/89–12/11/89, FIT (CHND, 2ex.); **Colombia**: Vaupes, Parco Nac. Mosiro-Itajura (Caparu) Centro Ambiental (-1.0667, -69.5167), 60 m, 1/20/03–1/20/03, FIT, D. Arias & M. Sharkey (MSCC, 2ex.); **Ecuador**: Orellana, Est. Biodiv. Tiputini (-0.6376, -76.1499), 6/2/11–6/9/11, FIT, M. Caterino & A. Tishechkin (MSCC, 2ex.); Orellana, Est. Cientifica Yasuní (-0.6744, -76.6472), 215 m, 9/5/99–9/10/99, FIT, primary forest, E. Riley (TAMU, 9ex.); **Peru**: Loreto, Campamento San Jacinto (-2.3125, -75.8628), 175–215 m, 7/11/93, FIT, R. Leschen, EXO-02930 (SEMC, 1ex.); Madre de Dios, CICRA Field Stn., ~ 2 km NW cafeteria res. plot. 12.55236°S, 70.10989°W, 295 m, Flight intrcept. 7–9.vi.2011, Chaboo Team (SEMC); Marowijne, Palumeu, (3.3489, -55.4383), 160 m, 7/7/99–7/8/99, FIT, Z. Falin, SM0176323 (SEMC, 1ex.); Region 8, Iwokrama Forest, Pakatau hills (4.7483, -59.0267), 70 m, 5/25/01–5/29/01, FIT, R. Brooks, Z. Falin (SEMC, 2ex.); Saramacca, West Suriname Road, 145 km WSW Zanderij Airport (5.1517, -56.1456), 50 m, 6/10/99–6/14/99, FIT, Z. Falin, B. DeDijn, SM0179990 (SEMC, 1ex.); Sipaliwini, CI-RAP Surv. Camp 4: on lower Kasikasima River (-2.9773, -55.3850), 200 m, 3/20/12–3/25/12, FIT, T. Larsen (SEMC, 1ex.).

###### Diagnostic description.

Length: 1.54–1.81 mm (avg. 1.74 mm); width: 1.38–1.58 mm (avg. 1.50 mm). Body elongate oval, widest behind elytral humeri, rufescent, with ground punctation fine; frons depressed along midline; frontal stria fine but complete within median depression; epistoma with raised edges along sides and front; labrum rather narrow, emarginate & weakly subcarinate along apical margin; mandibles lacking basal teeth; prescutellar impression large, semicircular, ~ 3 × width of scutellum; median pronotal gland openings conspicuous, annulate, ~ 3/4 behind anterior margin; lateral thirds of pronotal disk (laterad a longitudinal line drawn through the gland opening) abruptly more coarsely punctate, secondary punctures varied in size and density; marginal pronotal stria complete along lateral and anterior margins; submarginal stria complete, well-impressed along sides, just turning anterior corner; elytron with single, complete epipleural stria; outer subhumeral stria present at base and apex but usually interrupted at middle; inner subhumeral stria absent; dorsal striae 1–4 complete, 4^th^ arched to sutural, 5^th^ present in apical 1/2, sutural stria slightly longer; propygidium with moderately coarse secondary punctation, that of pygidium sparser; prosternal keel emarginate at base, striae separate at base, converging, united anteriorly; prosternal lobe slightly narrowed, with marginal stria complete, slightly removed from the margin; mesoventrite produced, with complete marginal stria continued at sides by postmesocoxal stria ending at middle of lateral portion of metaventrite; mesometaventral stria angulate at middle, crenulate, reaching middle of mesoventrite, continued at sides by lateral metaventral stria, reaching inner 1/3 of metacoxa; 1^st^ abdominal ventrite with incomplete lateral stria along inner margin of metacoxa; metaventrite lacking secondary punctation; 1^st^ abdominal ventrite with secondary punctures along anterior margin; protibia with outer margin weakly dentate, with five or six robust marginal spines; meso- and metatibiae slender, the mesotibia with ~ five spines along outer margin, metatibia with fewer marginal spines restricted to apical 1/2. Male: S8 with conspicuous apical fringe; aedeagus with basal piece ~ 1/4 length of tegmen; tegmen with sides subparallel to near apex, apex abruptly narrowed, weakly curved in lateral aspect; medioventral process present in basal 1/3; median lobe ~ ½ tegmen length, basal apodemes short, thin.

###### Etymology.

We name this species for the distinctive marginal fringe on the eighth sternite of the male genitalia.

###### Remarks.

While this species shares a lot of its characters with various others in this group, the combination of having its outer subhumeral stria usually interrupted at middle, having ‘normal’ (not scalloped) protibiae, and having the lateral thirds of the pronotal disk quite distinctly more strongly punctate than the middle third (Fig. 3E) is unique. It is particularly similar to the following species (*P. stellans*), but it differs from that one in having the arch of 4^th^ dorsal stria a little narrower, and the 5^th^ and sutural striae a little shorter. These seemingly insubstantial differences are backed up by a remarkable difference in the male 8^th^ sternite (Fig. 6A), which has a long apical fringe; the 8^th^ sternite of *P. stellans* has at most a few setae at the outer corners. We have restricted the type series of both species to males where identity could be confirmed by genitalia.

Specimens of this species have only been collected using flight interception traps.

###### Distribution.

This species is known from the Guianas, as well as Amazonian Brazil, Peru, and Ecuador.

##### 4. Phelister stellans sp. nov.

Taxon classificationAnimaliaColeopteraHisteridae

E5F4A987-6734-53E1-9F40-69B0DA63EF57

http://zoobank.org/F379FB75-09EC-4082-B38C-B18C41ACCAA9

**Map 4.**
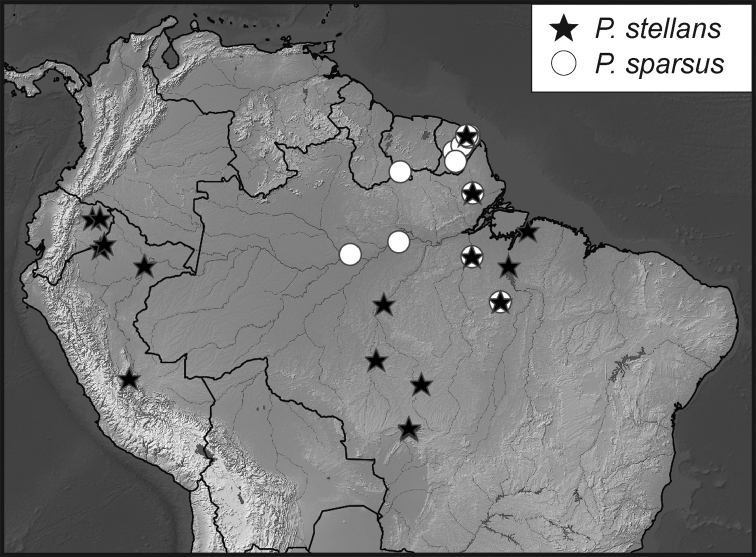
Collecting records for *Phelister stellans* (stars), *P. sparsus* (white circles).

Figs 3G, H , 6D, E , Map 4

###### Type material.

***Holotype* male**: “**Peru**: Loreto Prov. Iquitos, 90 m, 5 May 1992, J. Danoff-Berg(sic) ex. Flight intercept trap” / “SEMC0903641” (SEMC). ***Paratypes* (23 – confirmed males only): Peru**: Cusco, Villa Carmen Fld Stn., 12.89250°S, 71.41917°W, 555 m, 28–30.V.2011, DJ Bennett, FIT (SEMC, 1ex.); Cusco, Villa Carmen Fld Stn., 12.89221°S, 71.41946°W, 555 m, 20–30.V.2011, DJ Bennett, FIT (SEMC, 5ex); Loreto, Campamento San Jacinto (-2.3125, -75.8628), 175–215 m, 7/10/93, FIT, R. Leschen, EXO-02885 (SEMC, 1ex.); Loreto, Iquitos (-3.74, -73.27), 90 m, 5/7/92, FIT, J. Danoff-Berg (SEMC, 1ex.); Junín, 11 km NE Puerto Ocopa, Los Olivos (-11.05, -74.2587), 1200 m, 3/23/09–3/24/09, FIT, A. Tishechkin, EXO-00626 (AKTC, 1ex.); Madre de Dios, CICRA Field Stn., ~ 2 km NW cafeteria res. plot. 12.55236°S, 70.10989°W, 295 m, Flight intrcept. 9–13.vi.2010, Chaboo Team (SEMC, 5ex.); Madre de Dios, Pantiacolla Lodge, Alto Madre de Dios R. (-12.655, -71.2317), 420 m, 11/14/07–11/19/07, FIT, D. Brzoska (SEMC, 1ex.); **Ecuador**: Orellana, Est. Biodiv. Tiputini (-0.64, -76.15), 7/28/08–7/31/08, FIT, A.K.Tishechkin, EXO-02906 (AKTC, 1ex.); Orellana, Est. Biodiv. Tiputini (-0.64, -76.15), 7/28/08–8/3/08, FIT, A.K.Tishechkin (AKTC & LSAM, 3ex.); Orellana, Est. Biodiv. Tiputini (-0.64, -76.15), 8/3/08–8/6/08, FIT, LSAM Team (AKTC & LSAM, 3ex.); Orellana, Est. Cientifica Yasuní (-0.6744, -76.6472), 215 m, 9/5/99–9/10/99, FIT, primary forest, E. Riley (TAMU, 1ex.).

###### Other material.

**Peru**: Cusco, Villa Carmen Fld Stn., 12.89250°S, 71.41917°W, 555 m, 28–30.V.2011, DJ Bennett, FIT (SEMC, 3ex.); Cusco, Villa Carmen Fld Stn.,12.89221°S, 71.41946°W, 555 m, 20–30.V.2011, DJ Bennett, FIT (SEMC, 2ex); Junín, 11 km NE Puerto Ocopa, Los Olivos (-11.05, -74.2587), 1200 m, 3/28/09–3/30/09, FIT, A. Tishechkin (AKTC, 2ex.); Loreto, 1.5 km N Teniente Lopez (-2.5943, -76.1153), 210–240 m, 7/23/93, FIT, R. Leschen (SEMC, 3ex.); Loreto, Iquitos (-3.74, -73.27), 90 m, 5/5/92, FIT, J. Danoff-Berg (SEMC, 7ex.); Madre de Dios, CICRA Field Stn., ~ 2 km NW cafeteria res. plot. 12.55236°S, 70.10989°W, 295 m, Flight intrcept. 9–13.vi.2010, Chaboo Team (SEMC, 2ex.); Madre de Dios, CICRA Field Stn., ~ 2 km NW cafeteria res. plot. 12.55201°S, 71.10991°W, 295 m, Flight intrcept. 9–11.vi.2010, Chaboo Team (SEMC, 1ex.); Madre de Dios, CICRA Field Stn., ~ 2 km NW cafeteria res. plot. 12.55212°S, 70.10921°W, 295 m, Flight intrcept. 9–11.vi.2010, Chaboo Team (SEMC, 1ex.); **Brazil**: Amapá, Serra do Navio (0.9833, -52), 5/1/91–5/14/91, FIT, EXO-02899 (CHND, 1ex.); Mato Grosso, Mpio. Claudia (-11.4083, -55.325), 10/17/10–10/27/10, FIT, A.F. Oliveira, EXO-02903 (CEMT, 1ex.); Mato Grosso, Mpio. Cotriguaçu, Fazenda São Nicolau, Matinha (-9.8383, -58.2508), December 2010, FIT, F.Z. Vaz-de-Mello (CEMT, 3ex.); Mato Grosso, Mpio. Cotriguaçu, Fazenda São Nicolau, Matinha (-9.8383, -58.2508), October 2009, FIT, F.Z. Vaz-de-Mello, EXO-02902 (CEMT, 1ex.); Mato Grosso, Mpio. Diamantino, Vale da Solidão (-14.3638, -56.123), 640 m, February 2009, FIT, D.C.T. Oliveira (CEMT, 2ex.); Mato Grosso, Mun. Diamantino, Faz. São João (-14.2361, -56.1364), 400 m, 1/13/01, FIT, primary gallery for., Génier & Vaz-de-Mello, EXO-02900 (CMNC, 1ex.); Pará, Altamira – Marabá: km 18 (-3.15, -52.05), May 1984, FIT, EXO-02890 (CHND, 1ex.); Pará, Altamira – Marabá: km 18 (-3.15, -52.05), May 1985, FIT (CHND, 3ex.); Pará, Belém, Utinga (IPEAN) (-1.45, -48.4333), September 1984, FIT (CHND, 2ex.); Pará, Belém, Utinga (IPEAN) (-1.45, -48.4333), October, 1984, FIT (CHND, 6ex.); Pará, Belém, Utinga (IPEAN) (-1.45, -48.4333), November 1984, FIT (CHND, 2ex.); Pará, Belém, Utinga (IPEAN) (-1.45, -48.4333), October, 1985, FIT, EXO-02896 (CHND, 1ex.); Pará, Belém, Utinga (IPEAN) (-1.45, -48.4333), October, 1986, FIT, EXO-02898 (CHND, 1ex.); Pará, Carajás (Serra Norte) (-6.0667, -50.2), March 1985, FIT (CHND, 2ex.); Pará, Jacareacanga (-6.22, -57.76), December 1968, M. Alvarenga, EXO-02889 (DZUP, 1ex.); Pará, Tucuruí (-3.75, -49.667), 12/9/85–12/17/85, FIT, EXO-02897 (CHND, 1ex.); **Colombia**: Vaupes, Parco Nac. Mosiro-Itajura (Caparu), Centro Ambiental (-1.0667, -69.5167), 60 m, 1/20/03–1/30/03, FIT, D. Arias & M. Sharkey (AKTC, 10ex.); **Ecuador**: Orellana, Est. Biodiv. Tiputini (-0.64, -76.15), 7/28/08–7/31/08, FIT, EXO-02906 (AKTC, 1ex.); Orellana, Est. Biodiv. Tiputini (-0.64, -76.15), 7/28/08–7/31/08, FIT, A.K.Tishechkin (AKTC, 1ex.); Orellana, Est. Biodiv. Tiputini (-0.64, -76.15), 8/3/08–8/6/08, FIT, LSAM Team (AKTC & LSAM, 3ex.); Orellana, Est. Cientifica Yasuní (-0.675, -76.4), 7/11/08–7/24/08, FIT, A. Tishechkin (AKTC & LSAM, 6ex.); Orellana, Est. Cientifica Yasuní (-0.6744, -76.6472), 215 m, 9/5/99–9/10/99, FIT, primary forest, E. Riley (TAMU, 4ex.); **French Guiana**: Montagne des Chevaux (4.7167, -52.4), 9/21/08, FIT, SEAG, EXO-02907 (CHND, 1ex.); Montagne des Chevaux (4.7167, -52.4), 12/6/08–12/22/08, FIT, SEAG (CHND, 2ex.); Régina, Rés. Natur. des Nouragues, Camp Inselberg (4.0833, -52.6833), 1/25/11, FIT, SEAG (AKTC & CHND, 7ex.); **Suriname**: Sipaliwini, upper Palumeu River, CI-RAP Survey Camp 1, 2.4770°N, 55.6294°W, 275 m, flight intercept trap, 10–16.iii.2012, A. E. Z. Short (SEMC, 1ex.).

###### Diagnostic description.

Length: 1.85–2.09 mm (avg. 1.95 mm); width: 1.65–1.93 mm (avg. 1.73 mm). This species is very similar and closely related to the preceding, differing principally in the following characters: ground punctation of dorsum slightly coarser; secondary punctures of lateral portion of pronotum gradually increasing in size and density laterad; lateral submarginal pronotal stria usually not reaching anterior corner, abbreviated at front; outer subhumeral stria complete, not interrupted at middle. Male: S8 with only a couple setae at apical corner; basal piece ~ 1/3 length of tegmen; tegmen with sides weakly rounded, slightly wider toward base, more or less flat, with basal ‘notch’ in lateral view, apices subacute, weak medioventral process present in basal 1/3; median lobe ~ 1/2 tegmen length, basal apodemes thin.

###### Etymology.

The name of this species means starry, referring to the array of pronotal punctures.

###### Remarks.

This species, like the preceding one, has only been collected through the use of flight interception traps.

###### Distribution.

This species is known from Amazonian Ecuador, Peru, and Brazil, as well as French Guiana.

##### 5. Phelister sparsus sp. nov.

Taxon classificationAnimaliaColeopteraHisteridae

9B97D4AB-A109-50A0-AAB0-5F430A139627

http://zoobank.org/4B7940C1-E3BE-4A00-89B9-D9653A5B615B

Figs 5A, B , 6F, G , Map 4

###### Type material.

***Holotype* male**: “**French Guiana**: Roura, 18.4 km SSE, 4°36'38"N, 52°13'25"W, 29 MAY–10 JUN 1997, J. Ashe, R. Brooks FG1AB97 180 ex. Flight intercept trap” / “SM0131741 KUNHM-ENT” (SEMC). ***Paratypes* (87): French Guiana**: Belvédère de Saül, point de vue (3.6228, -53.2094), 12/10/10, 1/7/11, 2/7/11 and 2/14/11, FIT, SEAG (AKTC & CHND, 7ex.); Régina, Rés. Natur. des Nouragues, Camp Inselberg (4.0833, -52.6833), 8/20/10, FIT, SEAG, EXO-02951 (CHND, 1ex.); Régina, Rés. Natur. des Nouragues, Camp Inselberg (4.0833, -52.6833), 7/20/09, 9/9/10 and 9/22/09, FIT, SEAG (CHND, 3ex.); Régina, Rés. Natur. des Nouragues (4.0378, -52.6725), 1/28/10, FIT, SEAG (CHND, 13ex.); Régina, Rés. Natur. des Nouragues (4.0378, -52.6725), 2/19/10, FIT, SEAG (CHND, 6ex.); Roura, 18.4 km SSE (4.6106, -52.2236), 240 m, 5/29/97–6/10/97, FIT, J. Ashe & R. Brooks, (SEMC, 2ex.); Roura, 27.4 km SSE (4.7389, -52.2236), 280 m, 6/10/97, FIT, J. Ashe & R. Brooks, SM0132188 (SEMC, 1ex.); Saül, 7 km N, Les Eaux Claires (3.6628, -53.2219), 220 m, 5/31/97–6/3/97, FIT, J. Ashe & R. Brooks, SM0094399 (SEMC, 1ex.); Rés. Trésor (route de Kaw Pk18) (4.6105, -52.279), 225 m, 11/21/09, FIT, SEAG, EXO-02962 (CHND, 1ex.); Mont Tabulaire Itoupé (3.0303, -53.1067), 400 m, 3/17/10, FIT, SEAG (CHND, 5ex.); Mont Tabulaire Itoupé (3.0303, -53.1067), 570 m, 3/24/10, FIT, SEAG (CHND, 2ex.); Montagne des Chevaux (4.7167, -52.4), 7/11/09, FIT, SEAG, EXO-02961 (CHND, 1ex.); Montagne des Chevaux (4.7167, -52.4), 5/22/10, FIT, SEAG, EXO-02950 (CHND, 1ex.); Montagne des Chevaux (4.7167, -52.4), 12/6/08–12/22/08, 1/4/09–1/25/09, 2/23/09, 3/8/09–3/15/09 and 4/11/09, FIT, SEAG (AKTC & CHND, 18ex.); Route de Bélizon, p. km 4.5, flight intercept, 8/1/16–8/31/16, J.-L. Guiglaris (AVSC, 2ex.). **Suriname**: Sipaliwini, CI-RAP Survey Camp 1, upper Palumeu River (-2.4770, -55.6294), 275 m, 3/10/12–3/16/12, flight intercept, A.E.Z. Short (AKTC, 5ex.); Sipaliwini, CI-RAP Surv. Camp 1: on Kutari River (-2.1753, -56.7874), 228 m, 8/19/10–8/24/10, FIT, Larsen & Short (SEMC, 2ex.); Sipaliwini, CI-RAP Surv. Camp 2: Sipaliwini River (-2.1753, -56.7874), 210 m, 8/27/10–9/1/10, FIT, Larsen & Short (SEMC, 2ex.); Sipaliwini, CI-RAP Surv. Camp 3: Wehepai SE Kwamala (2.3629, -56.6977), 237 m, 9/3/10–9/7/10, FIT, Larsen & Short (SEMC, 12ex.); Sipaliwini, CI-RAP Surv. Camp 4: on lower Kasikasima River (2.9773, -55.3850), 200 m, 3/20/12–3/25/12, FIT, T. Larsen (SEMC, 1ex.).

###### Other material.

**Brazil**: Amapá, Serra do Navio (0.9833, -52), 1/28/90–2/2/90, FIT, EXO-02954 (CHND, 1ex.); Amapá, Serra do Navio (0.9833, -52), 1/28/90–2/2/90, FIT, EXO-02952 (CHND, 1ex.); Amapá, Serra do Navio (0.9833, -52), 5/1/91–5/14/91, FIT (CHND, 3ex.); Amazonas, Reserva Ducke, 26 km NE. Manaus Barbosa (-3, -59.94), February 1995, FIT, M.G.V., EXO-02953 (NHMUK, 1ex.);Pará, Altamira – Marabá: km 18 (-3.15, -52.05), 11/22/83, Ss. cad. de rat, EXO-02956 (CHND, 1ex.); Pará, Carajás (Serra Norte) (-6.0667, -50.2), 3/29/89–4/6/89, FIT, EXO-02955 (CHND, 1ex.).

###### Diagnostic description.

Length: 1.85–2.01 mm (avg. 1.95 mm); width: 1.65–1.93 mm (avg. 1.73 mm). Body somewhat narrowly elongate oval, convex, dark rufescent, with ground punctation very fine and inconspicuous; frons and epistoma narrow, depressed along midline, sparsely punctate; supraorbital stria present, narrowly separated from sides of frontal; frontal stria complete, sinuate through frontal depression; epistoma weakly raised along sides and front; labrum narrow, apically emarginate; mandibles lacking basal teeth; antennal club elongate with elongate median setose patch and two smaller basal setose patches on dorsal surface; prescutellar impression broadly oval, ~ twice as wide as scutellum; pronotal disk with sparse secondary punctures close to sides; median pronotal gland openings distinctly annulate, 3/4 behind anterior margin; marginal pronotal stria complete along sides and front; submarginal pronotal stria complete along sides, just turning anterior corner, the marginal bead markedly convex; elytron with single, complete epipleural stria, with a rather uniform row of punctures along its inner edge; outer subhumeral stria variably present in basal and apical halves, typically interrupted and basally fragmented (nearing absent), inner absent, dorsal stria one slightly abbreviated from apex, striae 2–4 complete, 4^th^ arched to suture, 5^th^ present in apical 1/2, sutural stria in apical 2/3, all striae formed by punctures connected by a thin stria; propygidium with large, elongate secondary punctures separated by ~ their widths, those of pygidium smaller and sparser, densest in basal corners; prosternal keel emarginate at base, striae separate basally, evenly convergent to meet ~ 1/4 from presternal suture; prosternal lobe short, bluntly triangular, lacking marginal stria; mesoventrite produced, with complete marginal stria continued at sides by postmesocoxal stria, which ends posterolaterad mesocoxa; mesometaventral stria bluntly angulate at middle, reaching middle of mesoventrite, continued at sides by lateral metaventral stria toward, but ending short of, inner 1/3 of metacoxa; metaventrite and 1^st^ abdominal ventrite impunctate; 1^st^ abdominal ventrite with incomplete lateral stria along inner margin of metacoxa; protibia with outer margin rounded, weakly dentate, with five or six marginal spines; male protarsus with flattened ventral setae; meso- and metatibiae slender, with marginal spines largely restricted to apical one-third. Male: basal piece ~ 1/4 length of tegmen; tegmen with sides subparallel to near apex, then apex roundly expanded, strongly curved in lateral view, medioventral process absent; median lobe ~ 3/4 tegmen length, basal apodemes abruptly thinner near tips.

###### Etymology.

The name *sparsus* is meant to contrast with the earlier *fimbriatus*, referring to the setae of the apex of the male 8^th^ sternite.

###### Remarks.

Like several members of this group, this species is relatively elongate, with weakly rounded sides, strongly convex, and with relatively inconspicuous ground punctation. It is somewhat generalized within the group, lacking the oddly modified, ‘scalloped’ tibiae seen in a few species. This species, *P. trigonisternus*, *P. pretiosus*, and *P. globosus* also have two small sensory patches in place of the basal antennal annulus (at least on the ventral/anterior face), have the elytral striae serially punctiform (variably, but always to a great degree), and the males have flattened protarsal setae. Among these, *P. sparsus* is distinguished by the combination of a broadly oval prescutellar impression, a complete lateral submarginal pronotal stria, the lack of a prosternal lobe stria, the presence of ~ 20 larger secondary lateral pronotal punctures, and an outer subhumeral stria that is present basally and apically, but interrupted in the middle.

###### Distribution.

This species is mainly found in lower Amazonian portions of Brazil, as well as in French Guiana and Suriname.

##### 6. Phelister pretiosus sp. nov.

Taxon classificationAnimaliaColeopteraHisteridae

30A932DE-E1B1-554C-B714-1B39CBFB298D

http://zoobank.org/52B124ED-1466-4771-A774-2DE7C09DDE0B

**Map 5.**
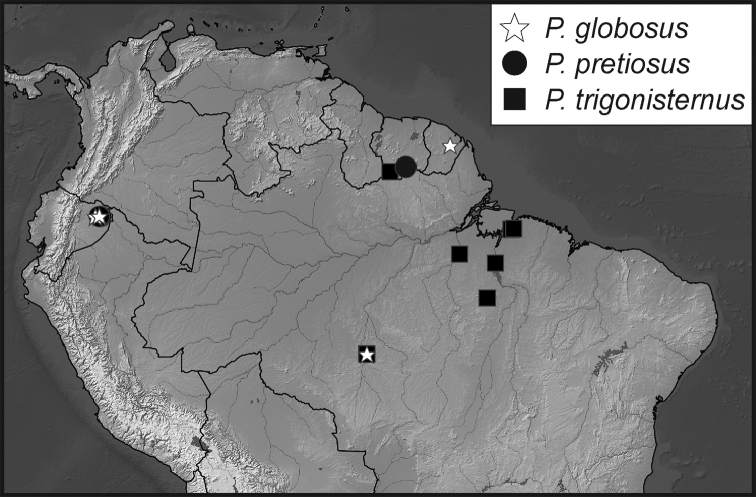
Collecting records for *Phelister pretiosus* (black circles), *P. trigonisternus* (black squares) and *P. globosus* (stars).

Figs 5C, D , 6H, I , Map 5

###### Type material.

***Holotype* male**: “**Ecuador** Napo Region 5–25.ix.00” / “Tiputini Research Station 220m 0°38'0"S, 76°9'0"W” / “BM2000:194 D.J.Inward K.A.Jackson” / “Caterino/Tishechkin Exosternini Voucher EXO-00359” (NHMUK). ***Paratypes* (3): Ecuador**: same data as type (NHMUK, 1ex.); Orellana, Est. Biodiv. Tiputini (-0.64, -76.15), 8/3/08–8/6/08, FIT, LSAM Team (AKTC, 1ex.); Orellana: Tiputini Biodiversity Station, 0.6376°S, 76.1499°W, 4–9.vi.2011, FIT, M.S. Caterino & A.K. Tishechkin (MSCC, 1ex.).

###### Other material.

**Ecuador**: Orellana, Est. Cientifica Yasuní (-0.675, -76.4), 7/19/08–7/22/08, FIT, A. Tishechkin (AKTC, 1ex.); **Suriname**: Sipaliwini, upper Palumeu River, CI-RAP Survey Camp 1, 2.4770°N, 55.6294°W, 275 m, flight intercept trap, 10–16.iii.2012, A. E. Z. Short (SEMC, 2ex.).

###### Diagnostic description.

Length: 1.97–2.17 mm (avg. 2.10 mm); width: 1.65–1.93 mm (avg. 1.84 mm). This species is also very closely related to *P. sparsus*, differing principally in the following characters: sides more broadly rounded; prosternal keel striae united by a transverse stria basally, enclosing a rather small triangular space ~ 3/4 as long as keel; prosternal lobe with marginal stria present; middle angle of mesometaventral stria only reaching basal fourth of mesoventrite; abdominal ventrite one with row of secondary punctures along anterior margin; male with aedeagus narrower, less curved in lateral view, with medioventral process that barely protrudes beneath; tergite 10 partially divided. Male: basal piece ~ 1/4 length of tegmen; tegmen widest near middle, evenly narrowed to base and apex, apex subtruncate; tegmen weakly curved, rather flat in lateral view, weak medioventral process present in basal 1/3; median lobe ~ 2/3 tegmen length, basal apodemes thin.

###### Etymology.

This species name means ‘precious’, in reference to its jewel-like appearance.

###### Distribution.

This species is known from only a few specimens from Amazonian Ecuador and Suriname.

###### Remarks.

The external characters listed in the description to distinguish this species are all relatively minor, but consistent. It lacks the labral fovea of *P. trigonisternus* and exhibits secondary punctures along the anterior margin of the 1^st^ abdominal ventrite that *P. sparsus* lacks. The male genitalia are also quite distinctive, with the tegmen sides being straighter than others (Fig. 6H vs. 6B, D, F, and 6J).

##### 7. Phelister trigonisternus

Taxon classificationAnimaliaColeopteraHisteridae

Marseul, 1889

D463AA46-6D2E-54BA-BED5-DA6B311D847B

Figs 4A–D , 5E–G , 6J, K , Map 5

Phelister trigonisternus Marseul, 1889: cxxxix.

###### Type material.

***Lectotype***, of undetermined sex, hereby designated: “Amer Sahlberg” / “2772” / “Marseul 14.12.86” / “trigonisternus” / “Type” / “Phelister trigonisternum [sic] Mars Type” / “Lectotype Phelister trigonisternus Marseul, 1889, M.S.Caterino & A.K.Tishechkin des. 2010”; NHMUK.

###### Other material.

**Brazil**: Mato Grosso, Mpio. Cotriguaçu, Fazenda São Nicolau (-9.815, -58.2858), 12/15/10–12/18/10, FIT, F.Z. Vaz-de-Mello & A.F. Oliveira (CEMT, 2ex.); Mato Grosso, Mpio. Cotriguaçu, Fazenda São Nicolau, Matinha (-9.8383, -58.2508), October, 2009, FIT, F.Z. Vaz-de-Mello (CEMT, 3ex.); Pará, Altamira – Marabá: km 18 (-3.15, -52.05), May 1984, FIT (CHND, 8ex.); Pará, Barcarena (-1.5, -48.6167), 6/13/91–6/25/91, FIT (CHND, 4ex.); Pará, Barcarena (-1.5, -48.6167), 12/7/91–12/20/91, FIT, EXO-02915 (CHND, 1ex.); Pará, Belém, Utinga (IPEAN) (-1.45, -48.4333), October, 1984, FIT (CHND, 4ex.); Pará, Belém, Utinga (IPEAN) (-1.45, -48.4333), November 1984, FIT (CHND, 3ex.); Pará, Belém, Utinga (IPEAN) (-1.45, -48.4333), August 1985, FIT (CHND, 3ex.); Pará, Carajás (Serra Norte) (-6.0667, -50.2), May 1985, FIT, EXO-02913 (CHND, 1ex.); Pará, Carajás, Serra Norte (-6.0667, -50.2), 1/22/84–1/27/84, Ss. cad. de Sarigue (CHND, 2ex.); Pará, Tucuruí (-3.75, -49.667), 11/28/85–12/11/85, FIT (CHND, 4ex.); Pará, Tucuruí (-3.75, -49.667), 2/16/89–3/2/89, FIT (CHND, 5ex.); **Suriname**: Sipaliwini, CI-RAP Surv. Camp 3: Wehepai SE Kwamala (2.3629, -56.6977), 237 m, 9/3/10–9/7/10, FIT, Larsen & Short, EXO-02918 (SEMC, 1ex.)

###### Diagnostic description.

Length: 2.09–2.36 mm (avg. 2.19 mm); width: 1.81–2.01 mm (avg. 1.89 mm). *Phelister trigonisternus* is closely related and very similar to the two described above (*P. sparsus* & *P. pretiosus*), differing principally in the following characters: head (frons) slightly broader, distinctly widened above antennal bases; labrum depressed on anterior surface, appearing foveate; lateral submarginal pronotal stria weak to obliterated by larger, more conspicuous lateral pronotal punctures (submarginal stria basically obsolete except for a few connected punctures in some); 5^th^ dorsal stria present in apical 1/3, sutural stria in apical 1/2; striae of prosternal keel weakly united along basal margin; mesometaventral stria reaching basal 1/3 of mesoventrite, lateral metaventral stria nearly reaching middle of metacoxa (longer and displaced laterad from its course in *P. sparsus*). Male: basal piece ~ 1/3 length of tegmen; tegmen weakly widened at middle, evenly narrowed to base and apex, apex subtruncate; tegmen weakly curved in lateral view, slightly humped near base, weak medioventral process present in basal 1/3; median lobe ~ 2/3 tegmen length, basal apodemes thin.

###### Remarks.

The impressed labrum of this species is unique among the species in this paper. This species was described only from ‘Amer.[ica]’, so the type locality is very imprecisely known. The closest match we were able to make was with a specimen from Pará, Brazil. It matches the lectotype very well in most characters but differs slightly in that the type lacks a basal fragment of the outer subhumeral stria, has a weaker frontal stria, and has the lateral pronotal punctation slightly sparser.

###### Distribution.

This species is known from central to northeastern Brazil and Suriname.

##### 8. Phelister globosus sp. nov.

Taxon classificationAnimaliaColeopteraHisteridae

82037AA5-D873-529A-B715-D2605C2E30FA

http://zoobank.org/2A57D901-5DCE-49B1-9850-610EC0340932

**Figure 7.**
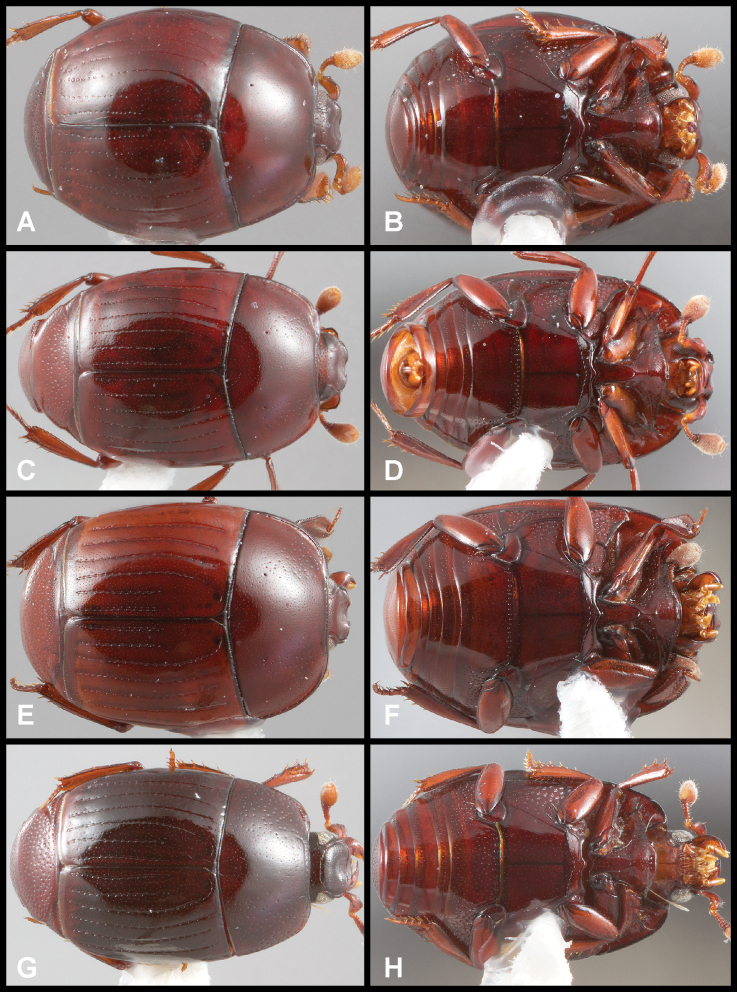
**A, B***Phelister globosus*: **A** dorsal habitus **B** ventral habitus **C, D***P. serratus*: **C** dorsal habitus **D** ventral habitus **E, F***P. geminus*: **E** dorsal habitus **F** ventral habitus **G, H***P. parana*: **G** dorsal habitus **H** ventral habitus.

**Figure 8.**
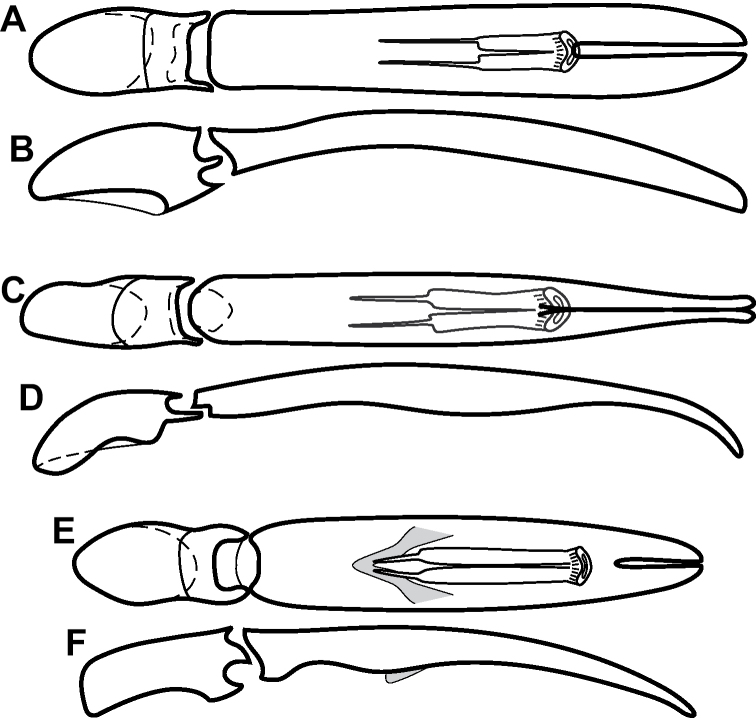
Male genitalia **A, B***Phelister globosus*: **A** aedeagus, dorsal **B** aedeagus, lateral **C, D***P. serratus*: **C** aedeagus, dorsal **D** aedeagus, lateral **E, F***P. parana*: **E** aedeagus, dorsal **F** aedeagus, lateral.

Figs 7A, B , 8A, B , Map 5

###### Type material.

***Holotype* male**: “**Ecuador**: Orellana, Tiputini Biodiversity Station 0°38.2'S, 76°8.9'W. Flight intercept FIT7-1. 27–31 July 2008. A.K.Tishechkin” / “Caterino/Tishechkin Exosternini Voucher EXO-00365” (FMNH). ***Paratypes* (45): Ecuador**: Orellana, Est. Biodiv. Tiputini (-0.6367, -76.1483), 7/27/08–7/31/08, FIT, A. Tishechkin, EXO-00365 (AKTC, CHND, LSAM & MSCC, 11ex.); Orellana, Est. Biodiv. Tiputini (-0.6367, -76.1483), 7/29/08–8/3/08, FIT, A. Tishechkin, EXO-00365 (AKTC & LSAM, 3ex.); Orellana, Est. Biodiv. Tiputini (-0.6367, -76.1483), 7/29/08–8/3/08, FIT, A. Tishechkin, EXO-00365 (AKTC, 1ex.); Orellana, Est. Biodiv. Tiputini (-0.6367, -76.1483), 8/3/08–8/6/08, FIT, LSAM Team, EXO-00365 (AKTC, CHND, LSAM & MSCC, 15ex.); Orellana, Est. Cientifica Yasuní, mid. Rio Tiputini (-0.675, -76.4), 6/18/99–7/20/99, FIT, A.K. Tishechkin (LSAM, 5ex.); Orellana, Est. Cientifica Yasuní, mid. Rio Tiputini (-0.675, -76.4), 7/11/08–7/24/11, FIT, A.K. Tishechkin, (AKTC, CHND, LSAM & MSCC, 9ex.); Orellana, Yasuni NP, Via Maxus at Puente Piraña, 0°39.5'S, 76°26'W, 245 m, flight intercept trap, 20–24.vii.2008, A. K. Tishechkin (AKTC, 1ex.).

###### Other material.

**Brazil**: Mato Grosso, Mpio. Cotriguaçu, Fazenda São Nicolau, Matinha (-9.8383, -58.2508), December 2010, FIT, F.Z. Vaz-de-Mello, EXO-00854 (CEMT, 1ex.); **French Guiana**: Montagne des Chevaux (4.7167, -52.4), 2/23/09–3/8/09, FIT, SEAG, (CHND, 3ex.); Régina, Rés. Natur. des Nouragues, Petit Plateau (4.09, -52.68), 9/16/10, FIT, S. Brûlé, EXO-02946 (DZUP, 1ex.); Régina, Rés. Natur. des Nouragues, Camp Inselberg (4.0833, -52.6833), 1/25/11, FIT (CHND, 1ex.); **Suriname**: Sipaliwini, CI-RAP Surv. Camp 1: on Kutari River (-2.1753, -56.7874), 228 m, 8/19/10–8/24/10, FIT, T. Larsen & A.E.Z. Short (SEMC, 1ex.); Sipaliwini, CI-RAP Surv. Camp 4: on lower Kasikasima River (-2.9773, -55.3850), 200 m, 3/20/12–3/25/12, FIT, T. Larsen (AKTC & SEMC, 4ex.).

###### Diagnostic description.

Length: 1.58–1.59 mm (avg. 1.58 mm); width: 1.26–1.38 mm (avg. 1.32 mm). Body small, elongate oval, convex, rufescent, with ground punctation very fine and inconspicuous; supraorbital stria absent; frons narrow, smooth, depressed at middle, depression barely extending onto epistoma, the latter mostly convex; frontal stria impressed at middle with ends curved dorsad, separate from sides; labrum narrow, produced, subangulate at middle; mandibles lacking basal teeth; prescutellar area depressed, but with impression weakly defined, little larger than scutellum; pronotal disk lacking secondary punctures; median pronotal gland openings small, not distinctly annulate, 3/4 behind anterior margin; marginal pronotal stria complete along sides and front; submarginal pronotal stria complete along the sides, just turning anterior corner, the marginal bead markedly convex; elytron with single, complete epipleural stria; outer subhumeral stria present in apical 1/2 only; inner absent; dorsal stria one abbreviated from apex, obsolete in apical one-third, striae 2–4 complete, but comprising series of weakly connected punctures apically, 4^th^ stria arched to near suture, 5^th^ stria present in apical one-third as series of punctures only, sutural stria a series of punctures in apical 1/2; propygidium only very sparsely punctate; prosternal keel broad, emarginate at base, striae widely separate basally, evenly convergent to meet in anterior arc; prosternal lobe short, rounded, slightly reflexed, with marginal stria rather deeply impressed and slightly removed from edge, especially at sides; mesoventrite produced, with very fine, complete marginal stria, continued at sides by postmesocoxal stria, which ends posterolaterad mesocoxa; mesometaventral stria fine, bluntly angulate at middle, nearly reaching middle of mesoventrite, continued at sides by lateral metaventral stria to middle of metacoxa; metaventrite and 1^st^ abdominal ventrite impunctate; 1^st^ abdominal slender, weakly dentate, with five or six prominent marginal spines; protarsus of both sexes with flattened ventral setae; meso- and metatibiae slender, with few fine marginal spines. Male: basal piece ~ 1/4 length of tegmen; tegmen narrowest near base, widening slightly toward rounded apex, weakly curved and thick in lateral view, medioventral process absent; median lobe ~ 1/3 tegmen length, basal apodemes abruptly narrowed at their midpoints.

###### Etymology.

This species name refers to its broadly rounded body.

###### Distribution.

This species is known from three widely scattered localities, Amazonian Ecuador, Mato Grosso, Brazil, and French Guiana.

###### Remarks.

This species is also quite similar to the above three, but is the most broadly rounded and convex of them. It is also distinguished by its smaller prescutellar impression, non-annulate median pronotal gland openings; lack of lateral pronotal punctures, and frontal stria with the median portion detached from the lateral portions. Despite the disjunct localities occupied by this species, no geographic variation is apparent.

##### 9. Phelister serratus sp. nov.

Taxon classificationAnimaliaColeopteraHisteridae

03697C5C-F522-54EC-8203-932FC760C1D5

http://zoobank.org/8CAABFE5-09F8-4963-9A7D-3946C03333E4

**Map 6.**
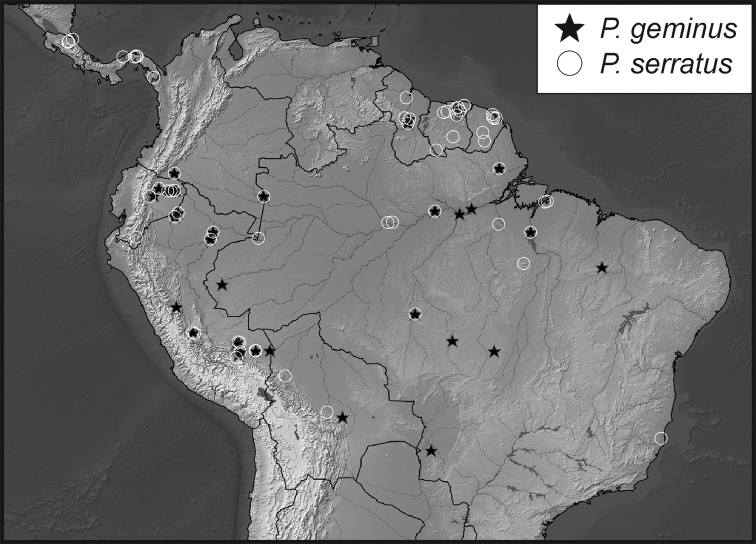
Collecting records for *Phelister serratus* (white circles), *P. geminus* (stars).

Figs 2F , 7C, D , 8C, D , Map 6

###### Type material.

***Holotype***: “**Ecuador**: Orellana: Est. Biodiv. Tiputini, 0.6376°S, 76.1499°W, flight intercepts, 4–9.vi.2011, M.S.Caterino & A.K.Tishechkin, AT1342” / “Caterino DNA Voucher, Extraction: MSC-2188, Species: ExoScutimp13.22, Extraction Date: viii.17.2011” / “Caterino/Tishechkin Exosternini Voucher EXO-00710” (FMNH). ***Paratypes* (202): Ecuador**: same data as type (MSCC, AKTC, CHND, 10ex.); Napo (20 km S. Tena) (-1.1, -78), 600 m, 7/11/76, Broken termite nests, S. Peck (FMNH, 5ex.); Orellana, Est. Biodiv. Tiputini (-0.6367, -76.1483), 7/27/08–8/3/08, FIT, A. Tishechkin, EXO-03056 (AKTC, 63ex.); Orellana, Est. Biodiv. Tiputini (-0.64, -76.15), 8/1/08, FIT, A. Tishechkin, EXO-00080 (1ex.); Orellana, Est. Biodiv. Tiputini (-0.64, -76.15), 8/3/08–8/6/08, FIT, LSAM Team (LSAM, 19ex.); Orellana, Est. Cientifica Yasuní, mid. Rio Tiputini (-0.675, -76.4), 6/18/99–7/23/99, FIT, A. Tishechkin, (LSAM, 3ex.); Orellana, Est. Cientifica Yasuní (-0.6744, -76.6472), 215 m, 9/5/99–9/10/99, FIT, primary forest, E. Riley (TAMU, 9ex.); Orellana, Est. Cientifica Yasuní (-0.675, -76.4), 7/10/08–7/24/08, FIT, A. Tishechkin (AKTC & LSAM, 61ex.); Orellana, Payamino Research Station (-0.4933, -77.2914), 300 m, 7/30/07–8/12/07, FIT, Tropical Rainforest, CPDT Gillett (NHMUK, 3ex.); Zamora, Chinchipe, Rio Bombuscaro (-4.1167, -78.9833), 6/26/96–7/4/96, FIT, P. Hibbs, SM0091227 (SEMC, 1ex.); **Colombia**: Amazonas, Leticia (-4.2, -69.9), 700ft, 2/24/74–2/28/74, S. Peck, EXO-03055 (CMNC, 1ex.); Vaupes, Est. Biol. Caparu, Rio Apoporis (-1.1, -69.5), 9/27/95–12/1/95, FIT, Black-water terrace forest on sandy soils, B. Gill (AKTC, 2ex.); Vaupes, Parco Nac. Mosiro-Itajura (Caparu), Centro Ambiental (-1.0667, -69.5167), 60 m, 1/20/03–1/30/03, FIT, D. Arias & M. Sharkey (AKTC, 10ex.); **Peru**: Loreto, Explornapo Lodge, 160 km NNE Iquitos (-3.2713, -72.8883) xii.29.1997, FIT, M.V.L. Barclay (NHMUK, 2ex.); Loreto, 1.5 km N Teniente Lopez (-2.5943, -76.1153), 210–240 m, 7/18–24/93, FIT, R. Leschen (SEMC, 5ex.); Loreto, Campamento San Jacinto (-2.3125, -75.8628), 175–215 m, 7/5–7/93, FIT, R. Leschen (SEMC, 5ex.); Loreto, Iquitos (-3.74, -73.27), 90 m, 5/5/92, FIT, J. Danoff-Berg (SEMC, 11ex.); Loreto, Iquitos – Nauta rd., km 58, Rio Itaya (-4.2563, -73.4675), 120 m, 5/5/09–5/9/09, FIT, next to entrance into *Eciton burchelli* statary bivouac in a hollow treee, A.V. Petrov, EXO-03118 (AKTC, 1ex.).

###### Other material.

**Bolivia**: Cochabamba, Univ. de San Simon, Cochabamba, 67.5 km NE Est. Biol. Valle del Sajita (-17.1092, -64.7978), 300 m, 2/7/99–2/9/99, FIT, R. Hanley (SEMC, 5ex.); La Paz., Rio Tuich, Chalan (-14.4267, -67.92), 320 m, 8/10/95–8/20/95, S. Spector, USNM 2026428 (USNM, 1ex.); **Brazil**: Amapá, Serra do Navio (0.9833, -52), 5/1/91–5/14/91, FIT (CHND, 2ex.); Amazonas, Presidente Figueiredo, 2.017°S, 59.717°W, 17.ix.2009, FIT, FWT Leivas (DZUP 2ex.); Amazonas, Reserva Ducke, 26 km NE. Manaus Barbosa (-3, -59.94), February 1995, FIT, M.G.V., EXO-03062 (NHMUK, 1ex.); BRAZIL: Amazonas, Reserva Ducke (-2.9, -59.9667), 11/29/77, emergence trap, J. Arias (USNM, 1ex.); Amazonas, Reserva Ducke, AM-010, km 26 (-3, -59.94), 12/2/77, J. Arias (USNM, 3ex.); Amazonas, Manaus (-3, -59.94), 7/9/08–7/10/08, FIT, P. Grossi, EXO-03063 (CHND, 1ex.); Amazonas, Rio Taruma Mirim 2 km from Rio Negro – Igapó (-3.0333, -60.2833), 7/27/79, fogged with Pyrethrum, Black-water innundation forest canopy, Adis, Erwin, Montgomery, et.al., EXO-03061 (USNM, 1ex.); Espírito Santo, Mun. Linhares, Faz. Lagoa do Macuco (-19.0639, -39.9786), 10 m, 1/27/00, FIT, primary lowland Atlantic forest, sandy soil, F. Génier & S. Ide, SM0809973 (SEMC, 1ex.); Mato Grosso, Mpio. Cotriguaçu, Fazenda São Nicolau, Mata Norte (-9.8192, -58.26), 12/8/10–12/14/10, FIT, F.Z. Vaz-de-Mello (CEMT, 6ex.); BRAZIL: Minas Gerais, Parque Estadual do Rio Doce, Centro de Pesquisas, 19.7637°S, 42.6303°W, 305 m, flight intercept (AKTC, 8ex.); Pará, Altamira – Marabá: km 18 (-3.15, -52.05), May 1985, FIT (CHND, 4ex.); Pará, Barcarena (-1.5, -48.6167), 12/7/91–12/20/91, FIT, EXO-03066 (CHND, 1ex.); Pará, Belém, Utinga (IPEAN) (-1.45, -48.4333), September 1984, FIT, EXO-03068 (CHND, 1ex.); Pará, Belém, Utinga (IPEAN) (-1.45, -48.4333), August 1985, FIT (CHND, 2ex.); Pará, Belém, Utinga (IPEAN) (-1.45, -48.4333), September 1985, FIT (CHND, 2ex.); Pará, Carajás (Serra Norte) (-6.0667, -50.2), May 1985, FIT, EXO-03075 (CHND, 1ex.); Pará, Carajás (Serra Norte) (-6.0667, -50.2), March 1985, FIT, EXO-03074 (CHND, 1ex.); Pará, Carajás (Serra Norte) (-6.0667, -50.2), October, 1986, FIT (CHND, 5ex.); Pará, Tucuruí (-3.75, -49.667), 11/28/89–12/11/89, FIT, EXO-03073 (CHND, 1ex.); Pará, Tucuruí (-3.75, -49.667), May 1986, FIT, EXO-03071 (CHND, 1ex.); Pará, Tucuruí (-3.75, -49.667), June 1985, FIT, EXO-03072 (CHND, 1ex.); Pará, July 1898, H.H. Smith, EXO-03065 (NHMUK, 1ex.); **Costa Rica**: Heredia, Est. Biol. La Selva (10.4333, -84.0167), 6/29/98, FIT, C. Carlton & A. Tishechkin, LSAM 0046301 (LSAM, 1ex.); Heredia, Est. Magsasay, P.N. Braulio Carrillo (10.4, -84.05), 200 m, January 1991, M. Barrelier, INBIO CRI000 701997 (INBIO, 1ex.); Limon, Sector Cerro Cocori, Fca de E. Rojas (10.6, -83.72), 150 m, August 1991, E. Rojas, INBIO CRI000 582416 (INBIO, 1ex.); Limon, Sector Cerro Cocori, Fca de E. Rojas (10.6, -83.72), 150 m, April 1992, E. Rojas, INBIO CRI000 861199 (INBIO, 1ex.); Limon, Sector Cerro Cocori, Fca de E. Rojas (10.6, -83.72), 150 m, May 1993, E. Rojas, INBIO CRI001 322121 (INBIO, 1ex.); **French Guiana**: Cayenne, 33.5 km S and 8.4 km NW of Hwy N2 on Hwy D5 (4.805, -52.4781), 30 m, 5/29/97–6/9/97, FIT, J. Ashe & R. Brooks, SM0102385 (SEMC, 1ex.); Roura, 27.4 km SSE (4.7389, -52.2236), 280 m, 6/10/97, FIT, J. Ashe & R. Brooks (SEMC, 2ex.); Saül, 7 km N, 3 km NW Les Eaux Claires, Mt. La Fumée (3.6628, -53.2219), 490 m, 6/1/97–6/8/97, FIT, J. Ashe & R. Brooks, SM0096866 (SEMC, 1ex.); Cayenne-Kourou, lieu-dit Tibourou (5, -52.5), 9/20/08, FIT, J. Touroult, EXO-03091 (CHND, 1ex.); Mont Tabulaire Itoupé (3.0303, -53.1067), 400 m, 3/17/10, FIT, SEAG (CHND, 2ex.); Mont Tabulaire Itoupé (3.0303, -53.1067), 400 m, 3/23/10, FIT, SEAG, EXO-03095 (CHND, 1ex.); Montagne des Chevaux (4.7167, -52.4), 1/18/09, FIT, SEAG, EXO-03093 (CHND, 2ex.); Montagne des Chevaux (4.7167, -52.4), 11/16/09, FIT, SEAG, EXO-03092 (CHND, 1ex.); Montagne des Chevaux (4.7167, -52.4), 1/4/09, & 4/11/09, FIT, SEAG (CHND, 2ex.); **Guyana**: Mazaruni Potaro, Takutu Mountains (6.25, -58.9167), 12/8/83, FIT, montane rainforest near logging area, W.E. Steiner & P.D. Perkins (USNM, 2ex.); Region 8, Iwokrama Forest, 26 km SW Kurupukari, Iwokrama Mt. (4.3339, -58.7883), 300 m, 5/23/01–5/25/01, FIT, R. Brooks, Z. Falin, SM0567082 (SEMC, 1ex.); Region 8, Iwokrama Forest, Pakatau hills (4.7483, -59.0267), 70 m, 5/25/01–5/29/01, FIT, R. Brooks, Z. Falin, SM0569306 (SEMC, 1ex.); Kurupukari (4.6667, -58.6667), September-November 1992, Malaise/FIT, EXO-03096 (NHMUK, 1ex.); Kurupukari, Mora Plot (4.6667, -58.6667), 10/26/92, FIT, EXO-03097 (NHMUK, 1ex.); **Panama**: Colon, San Lorenzo Forest (9.2833, -79.9667), 9/28/03–9/30/03, FIT, A. Tishechkin, EXO-03085 (AKTC, 1ex.); Colon, San Lorenzo Forest, STRI crane site (9.2833, -79.9667), 5/13/04–5/25/04, FIT, A. Tishechkin (AKTC, 7ex.); Colon, San Lorenzo Forest, STRI crane site (9.2833, -79.9667), 5/17/04–5/18/04, Crushed *Nasutitermes* nest, site, A. Tishechkin, EXO-03088 (AKTC, 1ex.); Darien, Cana Biological Station, Serrania de Pirre (7.755, -77.685), 1200 m, 6/4/96–6/9/96, FIT, J. Ashe & R. Brooks (SEMC, 9ex.); Darien, Cana Biological Station, Serrania de Pirre (7.755, -77.685), 1380 m, 6/4/96–6/9/96, FIT, J. Ashe & R. Brooks (SEMC, 3ex.); Darien, Cana Biological Station (7.755, -77.685), 550 m, 6/3/96–6/7/96, FIT, J. Ashe & R. Brooks (SEMC, 7ex.); Panama, Chepo-Carti Rd. (9.28, -79.1), 400 m, June 1982, FIT, B. Gill (CNCI, 2ex.); San Blas, Nusagandi Res. (9.35, -78.9833), 350 m, 5/16/95–5/17/95, FIT, J.&A. Ashe (SEMC, 2ex.); San Blas, Nusagandi Res. (9.3167, -78.9167), 320 m, 5/27/95–5/28/95, FIT, Gillogly – Stockwell, EXO-03084 (TAMU, 1ex.); **Peru**: Cusco, Villa Carmen Fld Stn. ~ 1.7 km west cafeteria, 12.89250°S, 71.41917°W, 555 m, 28–30.V.2011, DJ Bennett & E. Razuri, FIT (SEMC, 1ex.); Cusco, Villa Carmen Fld Stn., 12.87753°S, 71.40153°W, 555–1000 m, 26–28.V.2011, DJ Bennett, misc. hand collecting (SEMC, 1ex.); Cusco, Cock of the Rock Lodge NE Paucartambo (-13.055, -71.545), 1120 m, 11/4/07–11/9/07, FIT, D. Brzoska (SEMC, 4ex.); Junín, ~ 16 km NW Satipo, Rio Venado (-11.1989, -74.7705), 1110 m, 2/20/10–2/22/10, A.V. Petrov, EXO-03078 (AKTC, 3ex.); Junín, ~ 15 km NW Satipo, nr. Rio Venado at 11°11.18'S, 74°46.02'W, 1100–1400 m, 4/20/19–5/18/19, A. V. Sokolov (AVSC, 2ex.); Junín, ~ 3 km S Satipo, Ricardo Palma (-11.276, -74.642), 780 m, 3/19/09–3/21/09, FIT, J. Tapia & A. Tishechkin, EXO-00383 (AKTC, 1ex.); Junín, 11 km NE Puerto Ocopa, Los Olivos (-11.05, -74.2587), 1200 m, 3/25/09–4/1/09, FIT, A. Tishechkin (AKTC, 15ex.); Madre de Dios, Cocha Cashu Bio. Stn., Manu Nat. Park (-11.8958, -71.4067), 350 m, 10/17/00–10/19/00, FIT, R. Brooks, SM0257955 (SEMC, 1ex.); Madre de Dios, Los Amigos Field Station, Manu (-12.5424, -70.1343), 284 m, 7/20/06–7/29/06, pitfall trap, Pacal bamboo forest, J. Jacobs, CASENT 8123334 (CASC, 1ex.); Madre de Dios, Los Amigos Field Station, Manu (-12.5421, -70.1435), 292 m, 7/31/06–8/9/06, pitfall trap, Huangana terra firma forest, J. Jacobs, CASENT 8123335 (CASC, 1ex.); Madre de Dios, Los Amigos Field Station, Manu (-12.5657, -70.0988), 272 m, 7/31/06–8/9/06, pitfall trap, Sobrevuelo terra firma forest, J. Jacobs, CASENT 8123336 (CASC, 1ex.); Madre de Dios, Pakitza Bio. Stn., Reserved Zone, Manu National Park, Castanal Trail (-11.9447, -71.2833), 317 m, 10/15/00–10/16/00, FIT, R. Brooks (SEMC, 4ex.); Madre de Dios, Pantiacolla Lodge, 5.5 km NW El Mirador Trail, Alto Madre de Dios River (-12.6528, -71.2578), 500 m, 10/23/00–10/26/00, FIT, R. Brooks (SEMC, 3ex.); Madre de Dios, Pantiacolla Lodge, Alto Madre de Dios R. (-12.655, -71.2317), 420 m, 11/14/07–11/19/07, FIT, D. Brzoska (SEMC, 16ex.); Madre de Dios, CICRA Field Stn., ~ 2 km NW cafeteria res. plot. 12.55236°S, 70.10989°W, 295 m, Flight intrcept. 7–13.vi.2011, Chaboo Team (SEMC 35ex.); Madre de Dios, CICRA Field Stn., ~ 2 km NW cafeteria res. plot. 12.55212°S, 70.10921°W, 295 m, Flight intrcept. 9–11.vi.2010, Chaboo Team (SEMC, 4ex.); Cusco, Villa Carmen Fld Stn., 12.89221°S, 71.41946°W, 555 m, 20–22.V.2011, DJ Bennett, FIT (SEMC, 1ex.); **Suriname**: Brokopondo, Brownsberg Nature Preserve, Witi Creek Trail (4.9486, -55.1814), 340 m, 6/23/99–6/25/99, FIT, Z. Falin, A. Gangadin, H. Hiwat, SM0166958 (SEMC, 1ex.); Brokopondo, Brownsberg Nature Preserve, Witi Creek Trail (4.9486, -55.1814), 420 m, 6/23/99–6/25/99, FIT, Z. Falin, A. Gangadin, H. Hiwat, SM0178921 (SEMC, 1ex.); Commewijne, Akintosoela (5.2714, -54.9208), 40 m, 7/3/99, FIT, Z. Falin, EXO-03049 (CMNC, 1ex.); Commewijne, Akintosoela, 32 km SE Suriname River bridge, road to Redi Doti (5.2714, -54.9208), 40 m, 6/29/99–7/3/99, FIT, Z. Falin, B. DeDijn, A. Gangadin, SM0176718 (SEMC, 1ex.); Marowijne, Palumeu, (3.3489, -55.4383), 160 m, 7/5/99–7/9/99, FIT, Z. Falin, D. Konoe, SM0180204 (SEMC, 1ex.); Marowijne, Perica, 70 km E Paramaribo on East-West Road (5.6744, -54.6086), 5 m, 5/31/99–6/5/99, FIT, Z. Falin, B. DeDijn, SM0182867 (SEMC, 1ex.); Pará, nr. Overbridge at 5.5195, -55.0695, 2/10/10–2/14/10, FIT, W.B. Warner (WBWC, 4ex.); Pará, nr. Overbridge River Resort (5.53, -55.058), 2/15/10–2/18/10, FIT, C. Gillet, P. Skelley, W. Warner, EXO-03053 (FSCA, 1ex.); Pará, Zanderij, nr. Guesthouse (5.4583, -55.2167), 2/7/10–2/18/10, FIT, C. Gillet, P. Skelley, W. Warner (FSCA, 4ex.); Saramacca, West Suriname Road, 108 km WSW Zanderij Airport (5.2269, -55.8817), 30 m, 6/8/99–6/10/99, FIT, Z. Falin, B. DeDijn, SM0178833 (SEMC, 1ex.); Saramacca, West Suriname Road, 145 km WSW Zanderij Airport (5.1517, -56.1456), 50 m, 6/10/99–6/14/99, FIT, Z. Falin, B. DeDijn, SM0179968 (SEMC, 1ex.); Sipaliwini, CI-RAP Surv. Camp 1: on Kutari River (-2.1753, -56.7874), 228 m, 8/19/10–8/24/10, FIT, Larsen & Short, EXO-03050 (SEMC, 3ex.); Sipaliwini, CI-RAP Surv. Camp 3: Wehepai SE Kwamala (2.3629, -56.6977), 237 m, 9/3/10–9/7/10, FIT, Larsen & Short, EXO-03051 (SEMC, 1ex.); Sipaliwini, CI-RAP Surv. Camp 1: upper Palumeu River (2.4770°N, 55.6294°W), 275 m, FIT, 3/10/12–3/16/12, A.E.Z. Short (AKTC & SEMC, 5ex.); Sipaliwini, CI-RAP Surv. Camp 4: on lower Kasikasima River (-2.9773, -55.3850), 200 m, 3/20/12–3/25/12, FIT, T. Larsen (AKTC & SEMC, 3ex.).

###### Diagnostic description.

Length: 1.85–2.17 mm (avg. 2.01 mm); width: 1.50–1.77 mm (avg. 1.64 mm). Body subparallel-sided, weakly elongate, slightly flattened, rufescent, ground punctation of dorsum moderately fine; frons narrow, deeply impressed along midline; supraorbital stria fine, short, detached at sides; frontal stria deeply impressed along eyes, finely impressed at middle, complete; labrum weakly emarginate at apex; mandibles lacking basal teeth; antennal club elongate; prescutellar impression semicircular, ~ 2.5 × scutellar width; pronotum lacking secondary punctures in middle third, but with sparse (varied in density) secondary punctures laterad gland openings; median pronotal gland openings ~ 3/4 behind anterior margin, distinctly annulate; lateral submarginal pronotal stria complete, curved inward at front, weakly crenulate, disk depressed along its inner edge, marginal bead convex; elytron with single, complete epipleural stria; outer lateral subhumeral stria in apical 1/2 or less, inner absent; dorsal striae 1–4 complete, 4^th^ arched to suture, 5^th^ stria present in apical 1/2, sutural stria slightly longer; propygidium with small secondary punctures uniformly separated by about their diameters, those of pygidium slightly smaller and sparser; prosternal keel broad at base, striae widely separated at base, meeting 2/3 from base, keel abruptly narrowed anterad; prosternal lobe extremely short, with marginal stria; mesoventrite short, strongly projecting, marginal stria fine, continued at side by postmesocoxal stria to middle of lateral portion of metaventrite; mesometaventral stria bluntly angulate at middle, reaching middle of mesoventrite, lateral metaventral stria reaching middle of metacoxa; metaventrite impunctate; 1^st^ abdominal ventrite with single row of secondary punctures along anterior margin; protibiae narrow, outer margin ‘scalloped’, with a close set series of fine incisions, each with a minute spine, the apical-most spine larger; protarsal setae of male not modified; mesotibia with rather dense series of fine marginal spines, those of metatibia fewer, sparser, and principally restricted to apical 1/2. Male: basal piece sp. nov. 1/4 length of tegmen; tegmen with sides subparallel in basal 2/3, narrowed to thin, blunt, slightly divergent apices; weakly curved, thickened at middle in lateral view; medioventral process absent; median lobe ~ 1/3 tegmen length, basal apodemes abruptly narrowed at basal 1/3.

###### Etymology.

This species name refers to the somewhat ‘serrate’ protibiae, shared with the very similar following species.

###### Remarks.

This species and the next one (*P. geminus*) share one very distinctive character, having what we’ve previously referred to as ‘scalloped’ protibial margins (Fig. 2G), with very small, closely set marginal spines and no development of marginal teeth. While unusual, a similar form of protibia also occurs in the *P. umens* subgroup, not obviously related to this one. *Phelister serratus* and *P. geminus* are also rather elongate, parallel-sided, and relatively impunctate, with depressed pronotal margins, a quite vertical head, and a narrow frons. These two species are, however, extremely similar, and difficult to distinguish, even in male genitalia. However, we feel that the differences we have observed are sufficiently marked and consistent to recognize them as distinct (see Fig. 7C, D vs. 7E, F): *P. serratus* is narrower in body form, with the pronotal disk less strongly depressed along the inner edge of the lateral submarginal stria, has the median portion of the frontal stria attached to the lateral portions, and is generally more strongly punctate, both in ground punctation and in secondary pronotal punctures.

We have generated some DNA sequence data for individuals of each from the same (type) locality, and, while we have not successfully sequenced the barcoding gene for both, they are distinct in 18S sequence, which also supports the hypothesis that they are distinct. We have selected these respective DNA vouchers as the type specimens of each.

Central American specimens of *P. serratus* tend to show a few distinctive variants, but not consistently; often they have the prosternal keel less strongly narrowed toward the front, and the lateral epistomal ridges may appear striate. We considered separating these as distinct, but for now leave this as a widespread and variable species. In part for this reason, we have restricted the type series to a relatively small number of localities in eastern Ecuador and Colombia, and northern Peru.

###### Distribution.

This common and widespread species is known from Costa Rica southward to Bolivia, east to the Guianas and Amazonian Brazil, with one record from coastal Brazil (Espírito Santo).

##### 10. Phelister geminus sp. nov.

Taxon classificationAnimaliaColeopteraHisteridae

B44986AD-F4F7-54D4-A599-38B15896DA4C

http://zoobank.org/C39A5584-7FE2-4757-93D6-2A8188A6494B

Fig. 7E, F , Map 6

###### Type material.

***Holotype***: “**Ecuador**: Orellana: Est. Biodiv. Tiputini, 0.6376°S, 76.1499°W, flight intercepts, 4–9.vi.2011, M.S.Caterino & A.K.Tishechkin, AT1342” / “Caterino DNA Voucher, Extraction: MSC-2283, Species: ExoScutimpr. #4, Extraction Date: i.27.2012” / “Caterino/Tishechkin Exosternini Voucher EXO-00947” (FMNH). ***Paratypes* (217): Ecuador**: same data as type (MSCC, AKTC, 6ex.); Napo, Puerto Misahualli (-1.0333, -77.6667), 3/17/04–3/22/04, J. Jensen, EXO-03167 (CHND, 1ex.); Orellana, Est. Biodiv. Tiputini (-0.6333, -76.0383), 220 m, 9/5/00–9/25/00, FIT, D. Inward & K. Jackson (NHMUK, 11ex.); Orellana, Est. Biodiv. Tiputini (-0.6367, -76.1483), 7/27/08–8/3/08, FIT, A. Tishechkin, EXO-03162 (AKTC, 36ex.); Orellana, Est. Biodiv. Tiputini (-0.64, -76.15), 8/3/08–8/6/08, FIT, LSAM Team (LSAM, 33ex.); Orellana, Est. Cientifica Yasuní, mid. Rio Tiputini (-0.675, -76.4), 6/19/99, Berlese forest litter with *Eciton burchelli* refuse deposits, C. Carlton, LSAM 0045657 (LSAM, 1ex.); Orellana, Est. Cientifica Yasuní, mid. Rio Tiputini (-0.675, -76.4), 7/10/99–7/26/99, FIT, A. Tishechkin (LSAM, 67ex.); Orellana, Est. Cientifica Yasuní (-0.6744, -76.6472), 215 m, 9/5/99–9/10/99, FIT, primary forest, E. Riley, (TAMU, 31ex.); Orellana, Est. Cientifica Yasuní (-0.675, -76.4), 7/13/08, recently abandoned *E. burchelli* bivouac, A. Tishechkin, EXO-00079 (AKTC, 1ex.); Orellana, Payamino Research Station (-0.4933, -77.2914), 300 m, 7/30/07–8/12/07, FIT, Tropical Rainforest, CPDT Gillett (NHMUK, 4ex.). **Colombia**: Vaupes, Est. Biol. Caparu, Rio Apoporis (-1.1, -69.5), 9/27/95–12/1/95, FIT, Black-water terrace forest on sandy soils, B. Gill (AKTC, 2ex.); Vaupes, Parco Nac. Mosiro-Itajura (Caparu) Centro Ambiental (-1.0667, -69.5167), 60 m, 1/20/03–1/30/03, FIT, D. Arias & M. Sharkey, EXO-03170 (10ex.); **Peru**: Loreto, Explornapo Lodge, 160 km NNE Iquitos (-3.2713, -72.8883) xii.29.1997, FIT, M.V.L. Barclay (NHMUK, 2ex.); Loreto, 1.5 km N Teniente Lopez (-2.5943, -76.1153), 210–240 m, 7/22–24/93, FIT, R. Leschen (SEMC, 3ex.); Loreto, Campamento San Jacinto (-2.3125, -75.8628), 175–215 m, 7/7/93, FIT, R. Leschen (SEMC, 3ex.); Loreto, Iquitos (-3.74, -73.27), 90 m, 5/5–7/92, FIT, J. Danoff-Berg (SEMC, 3ex.); Loreto, Iquitos – Nauta rd., km 58, Rio Itaya (-4.2563, -73.4675), 120 m, 5/5/09–5/9/09, FIT, next to entrance into *Eciton burchelli* statary bivouac in a hollow treee, A.V. Petrov, EXO-03139 (AKTC, 1ex.).

###### Other material.

**Bolivia**: Santa Cruz, 5 km SSE Buena Vista, Flora y Fauna Hotel (-17.4987, -63.6521), 12/15/03–12/24/03, FIT, S. & J. Peck, EXO-03172 (AKTC, 1ex.); Santa Cruz, ~ 5 km SSE Buena Vista, Flora y Fauna Hotel, 17.498°S, 63.652°W, 440 m, flight intercept trap, 24–31.xii.2003, S. & J. Peck (AKTC, 2ex.); **Brazil**: Acre, Cruzeiro do Sul (-7.6333, -72.6), January-February 1988, FIT (CHND, 7ex.); Amapá, Serra do Navio (0.9833, -52), 5/1/91–5/14/91, FIT, EXO-03150 (CHND, 1ex.); Maranhão, Mirador, Caicarinha (-6.3667, -44.3667), 5/1/93, FIT, EXO-03152 (CHND, 1ex.); Mato Grosso, Mpio. Cotriguaçu, Fazenda São Nicolau (-9.8167, -58.2767), 12/15/10–12/18/10, FIT, F.Z. Vaz-de-Mello & A.F. Oliveira (CEMT, 4ex.); Mato Grosso, Mpio. Cotriguaçu, Fazenda São Nicolau, Mata Norte (-9.8192, -58.26), 12/8/10–12/14/10, FIT, F.Z. Vaz-de-Mello (CEMT, 7ex.); Mato Grosso, Mpio. Cotriguaçu, Fazenda São Nicolau, Matinha (-9.8383, -58.2508), December 2010, FIT, F.Z. Vaz-de-Mello, EXO-03157 (CEMT, 1ex.); Mato Grosso, Mpio. Cotriguaçu, Fazenda São Nicolau, Matinha (-9.8383, -58.2508), October 2009, FIT, F.Z. Vaz-de-Mello (CEMT, 5ex.); Mato Grosso, Mpio. Cotriguaçu, Fazenda São Nicolau, Matinha (-9.8383, -58.2508), December 2009, FIT, F.Z. Vaz-de-Mello, EXO-03156 (CEMT, 1ex.); Mato Grosso, Municip. Tangara da Serra, Fazenda Diamante (-14.313, -57.5317), 515 m, 12/14/13, gallery forest, FIT, F. Génier & L. Sawaris (CMNC, 1ex.); Mato Grosso, Mpio. Querencia, Fazenda São Luiz (-12.597, -52.3749), February 2009, FIT, R. Andrade (CEMT, 2ex.); Mato Grosso, Sinop (-11.8167, -55.4833), 6/12/85–6/24/85, FIT (CHND, 3ex.); Mato Grosso do Sul, Corumba, Passo da Lontra (-19.965, -57.07), 6/19/08, pitfall trap, no data, DZUP 272500 (DZUP, 1ex.); Pará, Ilha Arapiuns (-2.4, -54.95), 12/30/08–12/31/08, FIT (CHND, 6ex.); Pará, Monte Alegre (-2, -54.07), 6/17/92–7/3/92, FIT (CHND, 3ex.); Pará, Tucuruí (-3.75, -49.667), 11/28/89–12/11/89, FIT, EXO-03148 (CHND, 1ex.); Pará, Tucuruí (-3.75, -49.667), August 1984, FIT (CHND, 3ex.); **French Guiana**: Montagne des Chevaux (4.7167, -52.4), 1/18/09, 2/23/09 and 4/11/09, FIT, SEAG (CHND, 3ex.); **Guyana**: Region 8, Iwokrama Field Stn., Iwokrama Forest, 1 km W Kurupukari (4.6719, -58.6844), 60 m, 5/20/01–5/25/01, FIT, R. Brooks, Z. Falin, SM0567396 (SEMC, 1ex.); Region 8, Iwokrama Field Stn., Iwokrama Forest, 26 km SW Kurupukari, Iwokrama Mt. Base camp (4.3381, -58.8106), 100 m, 5/22/01–5/25/01, FIT, R. Brooks, Z. Falin, SM0572778 (SEMC, 1ex.); **Peru**: Cusco, Villa Carmen Fld Stn., 12.89221°S, 71.41946°W, 555 m, 24–26.V.2011, DJ Bennett, FIT (SEMC, 1ex.); Junín, ~ 16 km NW Satipo, Rio Venado (-11.1989, -74.7705), 1110 m, 2/19/10–2/22/10, A.V. Petrov (AKTC, 4ex.); Junín, ~ 15 km NW Satipo, nr. Rio Venado at 11°11.18'S, 74°46.02'W, 1100–1400 m, 4/20/19–5/18/19, A. V. Sokolov (AVSC, 1ex.); Madre de Dios, Cocha Cashu Bio. Stn., Manu Nat. Park (-11.8958, -71.4067), 350 m, 10/17/00–10/19/00, FIT, R. Brooks, EXO-03125 (CMNC, 5ex.); Madre de Dios, Cocha Cashu Bio. Stn., Manu Nat. Park (-11.8958, -71.4067), 350 m, 10/17/00–10/19/00, FIT, R. Brooks (SEMC, 18ex.); Madre de Dios, Cocha Salvador, Manu National Park (-12.0036, -71.5267), 310 m, 10/20/00–10/21/00, FIT, R. Brooks (SEMC & CMNC, 21ex.); Madre de Dios, Tambopata, Res. Cuzco Amazonico, 15 km NE Puerto Maldonado (-12.55, -69.05), 200 m, 6/20/89, FIT, J. Ashe, R. Leschen (CHSM & SEMC, 6ex.); Madre de Dios, Amazonas Lodge, N Atalaya (-12.87, -71.3767), 480 m, 11/10/07–11/13/07, FIT, D. Brzoska (SEMC, 2ex.); Madre de Dios, Pantiacolla Lodge, 2–7 km NW El Mirador Trail, Alto Madre de Dios River (-12.6528, -71.2578), 450–700 m, 10/23/00–10/26/00, FIT, R. Brooks (SEMC, 4ex.); Madre de Dios, Pantiacolla Lodge, 8 km NW El Mirador Trail, Alto Madre de Dios River (-12.6417, -71.2781), 800 m, 10/23/00–10/26/00, FIT, R. Brooks (SEMC, 9ex.); Madre de Dios, Pantiacolla Lodge, Alto Madre de Dios R. (-12.655, -71.2317), 420 m, 11/14/07–11/19/07, FIT, D. Brzoska (SEMC, 21ex.); Madre de Dios, Pantiacolla Lodge, Alto Madre de Dios River (-12.6561, -71.2319), 400 m, 10/23/00–10/26/00, FIT, R. Brooks (SEMC, 2ex.); Madre de Dios, Rio Los Amigos, CICRA(Puerto) (-12.5709, -70.1018), 230 msm, 8/5/06, basurero de *Eciton*, A. Asenjo (MUSM Lima, 2ex.); Huánuco Monson Valley, Tingo Maria (-9.3, -76), 11/2/54, E.I. Schlinger & E.S. Ross, EXO-03140 (CASC, 1ex.); **Suriname**: Commewijne, Akintosoela, 32 km SE Suriname River bridge, road to Redi Doti (5.2714, -54.9208), 40 m, 6/29/99–7/3/99, FIT, Z. Falin, B. DeDijn, A. Gangadin, SM0176662 (SEMC, 1ex.); Pará, Zanderij, nr. Guesthouse (5.4583, -55.2167), 2/9/10–2/18/10, FIT, C. Gillet, P. Skelley, W. Warner, EXO-03168 (FSCA, 1ex.); Sipaliwini, CI-RAP Surv. Camp 1: on Kutari River (-2.1753, -56.7874), 228 m, 8/19/10–8/24/10, FIT, Larsen & Short (SEMC, 4ex.); Sipaliwini, CI-RAP Surv. Camp 1: upper Palumeu River (2.4770°N, 55.6294°W), 275 m, FIT, 3/10/12–3/16/12, A.E.Z. Short (SEMC, 1ex.); Sipaliwini, CI-RAP Surv. Camp 4: on lower Kasikasima River (-2.9773, -55.3850), 200 m, 3/20/12–3/25/12, FIT, T. Larsen (AKTC & SEMC, 8ex.).

###### Diagnostic description.

Length: 1.65–2.05 mm (avg. 1.89 mm); width: 1.50–1.77 mm (avg. 1.62 mm). This species is extremely similar to the preceding (*P. serratus*), but differs consistently in being broader in body form (Fig. 7E), having the median portion of the frontal stria detached from the lateral portions rather than continuous across the frons, the pronotal disk more strongly depressed along the lateral margin, and in having sparser dorsal ground punctation, along with slightly lower density of secondary lateral pronotal punctures. The male genitalia exhibit no obvious differences from that of the *P. serratus* (Fig. 8C, D).

###### Etymology.

This species name refers to its being a near twin of the preceding.

###### Distribution.

This species’ distribution broadly overlaps the preceding, covering much of northern South America. It does not extend into Central America, however, and records are very few in the Guianas.

###### Remarks.

As discussed under the preceding species, these are two very similar species, easily distinguished from all others by their unusual protibiae, but minimally different from each other. The description covers all characters that we have been able to find to distinguish them. We have restricted the type series to a relatively small number of localities in eastern Ecuador and Colombia, and northern Peru.

##### 11. Phelister parana sp. nov.

Taxon classificationAnimaliaColeopteraHisteridae

EFB2A3E7-08CC-59F4-94F9-77E043E8EFEA

http://zoobank.org/B4130E05-1ED8-419A-A6EE-0BADF47DE8D3

**Map 7.**
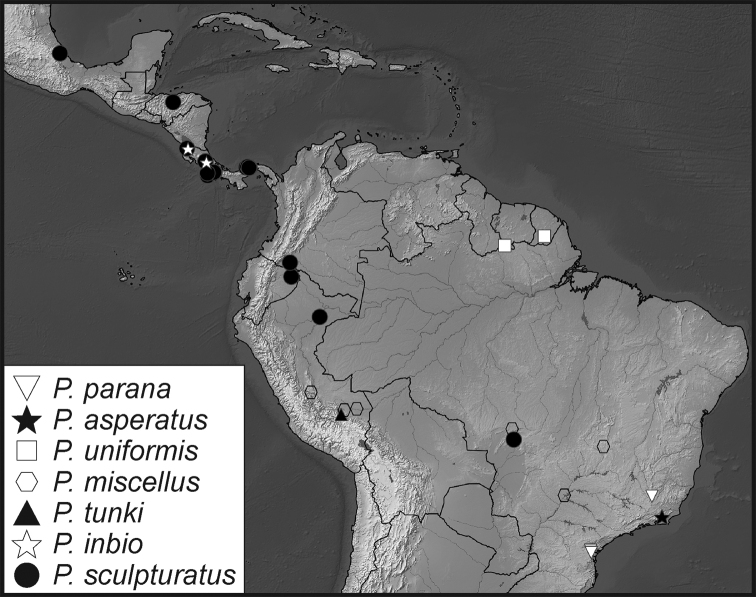
Collecting records for *Phelister parana* (inverted triangles), *P. asperatus* (black stars), *P. uniformis* (white squares), *P. miscellus* (white hexagons) *P. sculpturatus* (black circles), *P. inbio* (white stars) and *P. tunki* (black upright triangle).

Figs 7G, H , 8E, F , Map 7

###### Type material.

***Holotype* male**: “**Brazil**: Piraquara, Mananciais da Serra, 1,000 m, 25°29.77'S, 48°58.90'W, Arm. intercep. de vôo. 17–31.x.2007, FIT, P. Grossi & D. Parizotto” / “Caterino/Tishechkin Exosternini Voucher EXO-00424” (DZUP). ***Paratypes* (9): Brazil**: Paraná, Mananciais da Serra (-25.4934, -48.9786), 12/9/11–12/11/11, FIT, F.W.T. Leivas, M.S. Caterino & A.K. Tishechkin, EXO-00874 (MSCC, 1ex.); Paraná, Mpio. Curitiba, nr. Campina Grande do Sul (-25.2965, -49.0381), 12/7/11–12/10/11, FIT, F.W.T. Leivas, EXO-00875 (MSCC, 1ex.); Paraná, Piraquara (Sanepar) (-25.4961, -48.9817), 7/18/01, P.C. Grossi (DZUP, CESP, 2ex.); Paraná, Piraquara Mananciais da Serra (-25.4961, -48.9817), 1000 m, 11/23/07–12/1/07, FIT, P. Grossi & D. Parizotto (CHND, 3ex.); Paraná, Piraquara Mananciais da Serra (-25.4961, -48.9817), 1000 m, January 2007, FIT, P. Grossi & D. Parizotto (CHND, 2ex.).

###### Other material.

**Brazil**: Minas Gerais, Parque Estadual do Itacolomi, Trilha do Forno at 20.4290°S, 43.5075°W, 1350 m, flight intercept trap, 8–9.ii.2014, A. K. Tishechkin (AKTC, 1ex.)

###### Diagnostic description.

Length: 1.81–2.01 mm (avg. 1.89 mm); width: 1.42–1.81 mm (avg. 1.57 mm). This species is very similar to several others in this group, especially *P. blairi* and to *P. erwini*, but is unique in a few characters: body elongate (Fig. 7G), almost parallel-sided, with sides weakly rounded, dark rufescent, with conspicuous ground punctation; frons and epistoma deeply depressed along midline, sparsely but doubly punctate; pronotal gland openings just under halfway back on pronotal disk; pronotal disk with conspicuous secondary punctures throughout, sparser but distinct in middle third; marginal pronotal stria broken with inner ends recurved briefly behind eyes; submarginal pronotal stria short and weakly impressed, in anterior corners only; both propygidium and pygidium with dense secondary punctures; prosternal lobe very short, rounded. Male: basal piece ~ 1/3 length of tegmen; tegmen with sides weakly rounded, apices subacute, weakly separated; tegmen weakly curved, flatter toward apex, in lateral view; weak medioventral process present in basal 1/3; median lobe ~ 1/2 tegmen length, basal apodemes abruptly thinner near bases.

###### Etymology.

We name this species for the state of its primary occurrence, Paraná, Brazil.

###### Distribution.

This species is only known from a relatively small area in coastal southeastern Brazil.

###### Remarks.

The description presents all of this species’ distinguishing characters.

##### 12. Phelister asperatus sp. nov.

Taxon classificationAnimaliaColeopteraHisteridae

005CD6FA-5EAA-5F67-98F7-F56E1B91D334

http://zoobank.org/163C6211-C1F1-4563-84B6-407C7AF80F9C

**Figure 9.**
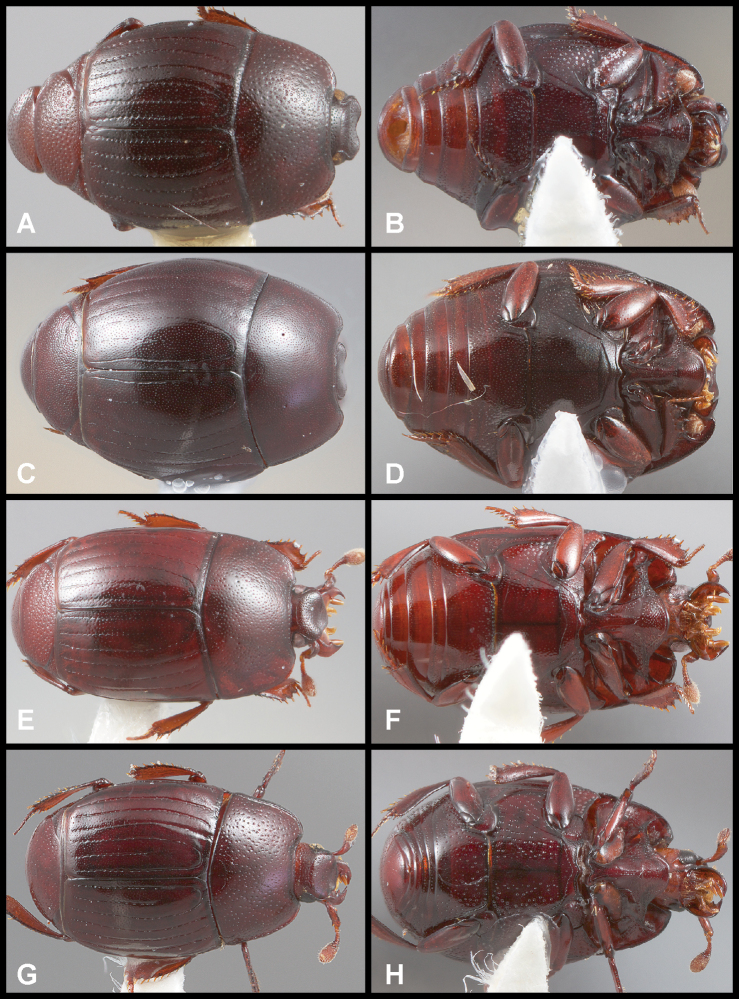
**A, B***Phelister asperatus*: **A** dorsal habitus **B** ventral habitus **C, D***P. uniformis*: **C** dorsal habitus **D** ventral habitus **E, F***P. miscellus*: **E** dorsal habitus **F** ventral habitus **G, H***P. inbio*: **G** dorsal habitus **H** ventral habitus.

**Figure 10.**
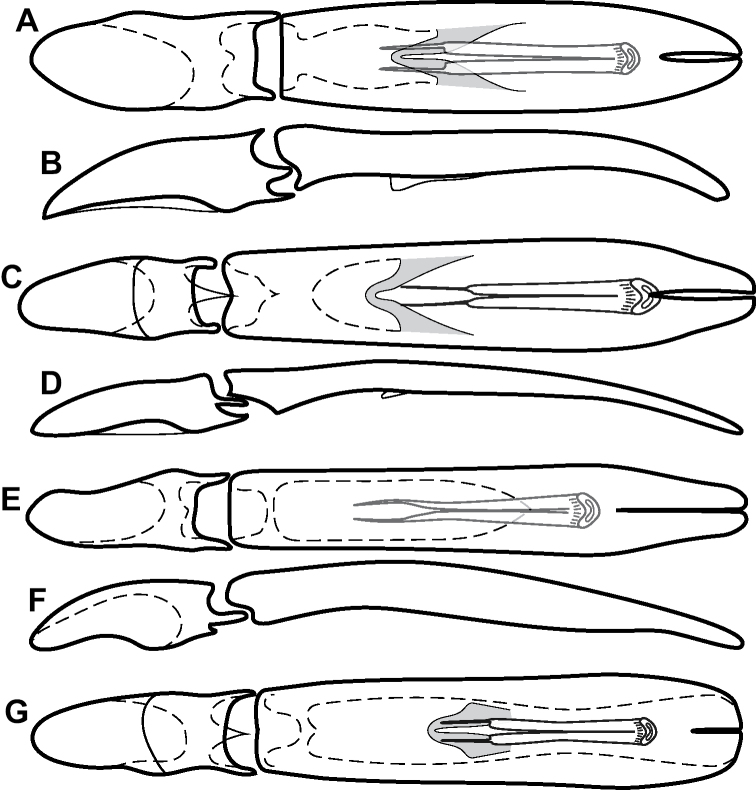
Male genitalia **A, B***Phelister asperatus*: **A** aedeagus, dorsal **B** aedeagus, lateral **C, D***P. uniformis*: **C** aedeagus, dorsal. **D** aedeagus, lateral **E, F***P. miscellus*: **E** aedeagus, dorsal **F** aedeagus, lateral **G***P. inbio*: Aedeagus, dorsal.

Figs 9A, B , 10A, B , Map 7

###### Type material.

***Holotype* male**: “**Brazil**, Rio de Janeiro: Nova Friburgo, Sans Souci, 9–15/XI/2009, E. Grossi (leg.)” / “Interceptação de vôo (FIT)” / “DZUP272524” (DZUP).

###### Diagnostic description.

Length: 1.50 mm; width: 1.22 mm. Body small, elongate oval, convex, rufescent, with conspicuous ground punctation and coarse secondary punctures uniformly intermixed on pronotum, more sparsely so on elytron; frons and epistoma somewhat narrow, deeply depressed along midline, doubly punctate; supraorbital stria present, separated from sides of frontal; frontal stria complete, fine across median depression; epistoma raised, subcarinate along sides; labrum emarginate and subcarinate apically; mandibles each with very small basal tooth; antennal club elongate with conspicuous setose patch on dorsal surface; prescutellar impression forming an elongate triangle with rounded bases, slightly wider than scutellum; pronotal disk densely, doubly punctate; median pronotal gland openings obscured by punctures; marginal pronotal stria complete along sides and front; submarginal pronotal stria obscure, vaguely present as disconnected series of punctures around anterior corner only; elytron with single, complete epipleural stria; outer subhumeral stria present in apical 1/2 only, inner subhumeral vaguely represented by series of punctures; dorsal striae 1–4 complete, 4^th^ arched to sutural stria, 5^th^ and sutural stria nearly complete, fragmented at bases; propygidium uniformly doubly punctate; prosternal keel emarginate at base, striae separate at base, sinuate, united anteriorly; prosternal lobe short, reflexed, with complete marginal stria; mesoventrite produced, with complete marginal stria continued at sides by postmesocoxal stria, which ends freely at middle of lateral portion of metaventrite; mesometaventral stria angulate at middle, crenulate, reaching middle of mesoventrite, continued at sides by lateral metaventral stria to middle of metacoxa; middle portion of metaventrite mainly with conspicuous ground punctation; 1^st^ abdominal ventrite with secondary punctures more densely intermingled, with incomplete lateral stria along inner margin of metacoxa; protibia with outer margin rounded, weakly dentate, with ~ five marginal spines; protarsal setae of male not flattened; meso- and metatibiae slightly broadened, with a few spines along outer margins. Male: basal piece nearly 1/2 length of tegmen; tegmen with sides weakly rounded, apices subacute, weakly separated; tegmen mostly straight in lateral view; medioventral process present in basal 1/3; median lobe ~ 2/3 tegmen length, basal apodemes abruptly thinner near bases.

###### Etymology.

This species name refers to its asperate, or rough appearance.

###### Distribution.

This species is only known from the type locality, Brazil: Rio de Janeiro.

###### Remarks.

This species is straightforward to recognize, particularly assuming it is restricted in distribution to southeastern Brazil. It has an unusual pattern of sculpture, with the pronotum densely doubly punctate, and the elytra less densely and mostly singly punctate. Among all the strongly punctate species in this group it is particularly small and elongate. Its elytral striae are also quite distinctive, appearing as double series of large punctures connected by a relatively fine stria.

##### 13. Phelister uniformis sp. nov.

Taxon classificationAnimaliaColeopteraHisteridae

70FEB5C7-FD26-52A1-8963-BCD5042F73AC

http://zoobank.org/DD4DE726-F0FE-484E-91F6-4CE5A3836701

Figs 9C, D , 10C, D , Map 7

###### Type material.

***Holotype* male**: “**Suriname**: Sipaliwini: CI-RAP Surv. Camp 1: on Kutari River, 228 M, 2°10.521'N, 56°47.244'W, 27.viii.2010, flight intercept trap, 19–24.viii.2010, Larsen & Short” / “Caterino/Tishechkin Exosternini Voucher EXO-00579” (SEMC). ***Paratypes* (10)**: “**Guyane Française**: Bélvédère de Saül, point de vue. 3°37'22"N, 53°12'34"W. Piège vitre, 7.ii.2011. SEAG leg.” (AKTC, MSCC, CHND & MNHN, 8 ex.); same locality, collector and method, but 2.ix.2010 (DZUP & CHND, 2 ex.).

###### Diagnostic description.

Length: 1.69–2.09 mm (avg. 1.93 mm); width: 1.46–1.81 mm (avg. 1.68 mm). Body ovoid, sides quite rounded, slightly flattened, dark rufescent, with conspicuous ground punctation (and little secondary punctation) on nearly all surfaces; frons and epistoma deeply depressed along midline; supraorbital stria in middle third only; frontal stria complete, very fine in median depression; sides of epistoma subcarinate; labrum short, emarginate apically; each mandible with very small basal tooth; antennal club elongate, with extended setose patch along dorsal surface; prescutellar impression weakly triangular, about as long as, and 1.5 × as wide as scutellum; pronotum without lateral secondary punctures; median pronotal gland openings ~ 3/4 behind anterior margin, distinct, annulate; marginal pronotal stria complete along front and sides, weakly crenulate in front; lateral submarginal stria complete, barely turned inward anteriorly, the marginal bead weakly convex; elytral epipleuron with single, complete marginal stria; outer subhumeral stria present basally and apically, narrowly interrupted at middle, inner subhumeral stria present in apical 1/2 to 2/3; dorsal striae 1–3 complete, stria four present but fragmented along most of its length, with distinct basal arch to suture, stria five very weakly impressed in apical 1/2, sutural stria complete, meeting basal arch; propygidium with few larger secondary punctures sparsely intermingled with dense ground punctation; pygidium with dense ground punctation only; prosternal keel emarginate at base, striae complete, separate at base, converging to middle, close and parallel anteriorly, united in narrow apical arch; keel with distinct, annulate gland openings; prosternal lobe rounded, with complete marginal stria; mesoventrite produced, with complete marginal stria continued at sides by postmesocoxal stria nearly to middle of lateral portion of metaventrite; mesometaventral stria bluntly angulate at middle, reaching middle of mesoventrite, continued by lateral metaventral stria to middle of metacoxa; mesoventrite, anterior and lateral portions of metaventrite, and sides of 1^st^ abdominal ventrite with conspicuous, transversely reticulate microsculputure; 1^st^ abdominal ventrite with single, incomplete lateral stria, also with distinct, annulate gland opening along inner edge of stria; protibia with outer margin distinctly dentate, with five marginal spines; male protarsal setae not expanded; meso- and metatibiae similar, weakly expanded to apex, with few marginal spines mostly confined to apical 1/2. Male: basal piece ~ 1/3 length of tegmen; tegmen with sides widening toward apical 1/4, then narrowed to blunt apices; tegmen rather flat, weakly curved in lateral view; medioventral process present in basal 1/3; median lobe ~ 2/3 tegmen length, basal apodemes abruptly thinner near bases.

###### Etymology.

This species name refers to its relatively ‘uniform,’ fine, even dorsal punctation.

###### Distribution.

This species is known only from French Guiana and Suriname.

###### Remarks.

Among several species with moderately conspicuous ground punctation, *P. uniformis* is distinctive in lacking larger secondary punctures, having a small, bluntly triangular prescutellar impression, the pronotal glands 2/3 back from anterior margin, the outer subhumeral stria interrupted at middle and the inner present in apical 1/2.

##### 14. Phelister miscellus sp. nov.

Taxon classificationAnimaliaColeopteraHisteridae

559FA418-A9CB-58F8-9A5E-0471C59A17D1

http://zoobank.org/3DCB1C33-21F0-4351-97F9-A4C345C73454

Figs 9E, F , 10E, F , Map 7

###### Type material.

***Holotype* male**: “**Brazil**: Distr. Federal Brasília, Reserva Ecol. de IBGE, Brasília, 15°56.5'S, 47°53'W, Cerrado, flight intercept trap. Oct.1986, I. Diniz” / “Caterino/Tishechkin Exosternini Voucher EXO-00446” (CEMT). ***Paratypes* (10): Brazil**: Mato Grosso, Mpio. Diamantino, Vale da Solidão (-14.3638, -56.123), 640 m, 26.i.2009, FIT, D.C.T. Oliveira (CEMT, 1ex.); Mato Grosso, Mpio. Diamantino, Vale da Solidão (-14.3638, -56.123), 640 m, February 2009, FIT, D.C.T. Oliveira (CEMT, 2ex.); Mato Grosso do Sul, cerradão fragment nr. Selvíria (-20.3354, -51.4095), 11/30/11–12/3/11, FIT, M. Caterino & A. Tishechkin, EXO-00857 (UNESP, 1ex.); Mato Grosso do Sul, cerradão fragment nr. Selvíria at 20°20'10"S, 51°24'36"W, 390 m, window traps, ground to 1.5 m above ground, 27.xi.2010–11.ii.2011, C. A. H. Flechtmann (UNESP, 6ex.)

###### Other material.

**Brazil**: Pará, Altamira – Marabá, km18. 3°09'S, 52°03'W. FIT, v.1984 (CHND, 1ex.); **Peru**: Junín, 11 km NE Puerto Ocopa, Los Olivos (-11.05, -74.2587), 1200 m, 3/29/09–3/30/09, FIT, A. Tishechkin, EXO-00670 (AKTC, 1ex.); Madre de Dios, Los Amigos Field Station, Manu (-12.5434, -70.1343), 284 m, 1/2/07–1/11/07, pitfall trap, Pacal terra firma forest, J. Jacobs, EXO-03042 (CASC, 1ex.); Madre de Dios, Pantiacolla Lodge, 2–7 km NW El Mirador Trail, Alto Madre de Dios River (-12.6528, -71.2578), 450–700 m, 10/23/00–10/26/00, FIT, R. Brooks, SM0261459 (SEMC, 1ex.); Cusco, Villa Carmen Fld Stn., 12.87753°S, 71.40153°W, 555–1000 m, 20–30.V.2011, DJ Bennett, misc. hand collecting (SEMC, 1ex.); Madre de Dios, CICRA Field Stn., ~ 2 km NW cafeteria res. plot. 12.55236°S, 70.10989°W, 295 m, Flight intrcept. 7–9.vi.2010, Chaboo Team (SEMC, 1ex.).

###### Diagnostic description.

Length: 1.81–2.01 mm (avg. 1.90 mm); width: 1.38–1.58 mm (avg. 1.47 mm). Body quite elongate, weakly rounded to almost parallel-sided, rufescent, with moderately conspicuous ground punctation throughout; frons broad, distinctly punctate, depressed along midline; supraorbital stria present for middle-3/4, abbreviated at sides; frontal stria interrupted at sides, present within median depression; epistoma weakly depressed; labrum smooth, flat emarginate; mandibles lacking basal teeth; prescutellar impression forming semi-circle ~ 2.5 × width of scutellum; median pronotal gland openings rather obscure among dense ground punctation, located within small impunctate areas ~ 2/3 behind anterior pronotal margin, not annulate; pronotal disk with secondary punctures only slightly larger than ground punctation, intermingled more densely from middle to lateral margin; marginal pronotal stria complete along lateral and anterior margins, crenulate at front; elytron with single, complete epipleural stria; outer subhumeral stria complete, inner absent; dorsal striae 1–4 complete, 4^th^ arched to suture, 5^th^ stria present in apical 2/3, sutural stria in apical 3/4; pygidia with moderately coarse secondary punctation, slightly sparser posterad; prosternal keel emarginate at base, striae separate at base, converging, united near apex; prosternal lobe weakly reflexed, rather narrow, with marginal stria complete; mesoventrite narrowly produced, with complete marginal stria continued by postmesocoxal stria nearly to base of metepisternum; mesometaventral stria bluntly angulate at middle, weakly crenulate, reaching middle of mesoventrite, continued at sides by lateral metaventral stria, reaching inner 1/3 of metacoxa; 1^st^ abdominal ventrite with single, complete lateral stria, bent posterad at inner posterior corner of metacoxa; middle of ventrites with only sparse ground punctation; protibia with outer margin somewhat rounded, weakly dentate, with six or seven marginal spines; mesotibia slightly broadened, with five or six long marginal spines; metatibia more slender, with two or three fine spines limited to apical 1/2. Male: basal piece ~ 1/3 length of tegmen; tegmen narrow, with sides widening toward apical fourth, then narrowed to bluntly rounded apices; tegmen rather thick, bent near base in lateral view; medioventral process absent; median lobe ~ 1/2 tegmen length, basal apodemes abruptly thinner near bases.

###### Etymology.

This species name refers to the mixed, or ‘miscellaneous,’ small and large punctures on the dorsum.

###### Distribution.

This species is known from southern Peru and south-central Brazil.

###### Remarks.

*Phelister* is distinctive in a several features, especially its elongate body form, doubled pronotal punctation, broad prescutellar impression, and complete lack of lateral submarginal pronotal stria. Its apical three protibial marginal spines also form a distinctly larger set than those closer to the base.

##### 15. Phelister inbio sp. nov.

Taxon classificationAnimaliaColeopteraHisteridae

4FB5B7CE-7F8A-5F68-95DE-6DF342B324A3

http://zoobank.org/F9476786-6F64-46E0-8D04-F813D7D60C3B

Figs 9G, H 10G, Map 7

###### Type material.

***Holotype* male**: “P.N. Tapantí, Prov Carta, **Costa Rica**. 1150 m. Mar 1994. G. Mora, L N 194000_559800 #2681” / “INBIO CRI001733668” (INBIO). ***Paratype* (1)**: “Est. Pitilla, 700 m, 9 km S. Sta. Cecilia, P. N. Guanacaste, Prov. Guan. COSTA RICA. P.Rios, 4–25 Nov 1991,L-N-330200_380200” / “INBIO CRI000497380” (MNCR).

###### Diagnostic description.

Length: 2.60–2.68 mm (avg. 2.64 mm); width: 2.05–2.13 mm (avg. 2.09 mm). Body elongate, rather narrow, widest behind elytral humeri, rufescent, with conspicuous ground punctation throughout, the pronotum with uniformly dense secondary punctation as well; frons moderately depressed along midline, supraorbital stria present, fine, disconnected at sides; frontal stria complete, epistoma raised, subcarinate along sides and front; labrum emarginate but not subcarinate along apical margin; both mandibles with conspicuous basal tooth; pronotum with large, semicircular prescutellar impression, ~ 5 × scutellar width; median pronotal gland openings obscured by secondary punctures but present just beyond midway behind anterior margin, distinctly annulate; marginal pronotal stria complete along lateral and anterior margins; lateral submarginal stria complete, clearly impressed, just turning anterior corner; elytron with single, complete epipleural stria; outer subhumeral stria complete, characteristically bent inward along basal elytral margin, inner absent; dorsal striae 1–4 complete, 4^th^ arched to sutural, 5^th^ obsolete in basal 1/3, sutural stria complete; pygidia with uniformly sparse secondary punctation; prosternal keel narrow, emarginate at base, striae separate at base, converging then subparallel to apex, united anteriorly; prosternal lobe narrowed, weakly reflexed, with marginal stria complete; mesoventrite produced, with complete marginal stria continued at sides by postmesocoxal stria ending laterad mesocoxa; mesometaventral stria angulate at middle, crenulate, reaching anterior 1/3 of mesoventrite, continued at sides by lateral metaventral stria, reaching middle of metacoxa; 1^st^ abdominal ventrite with incomplete lateral stria along inner margin of metacoxa; ventrites with rather uniformly sparse secondary punctation, the metaventrite largely impunctate along midline; protibia with outer margin strongly dentate, with five or six marginal spines; meso- and metatibiae slightly broadened, the mesotibia with rather robust spines along outer margin, those of metatibia fewer and finer. Male: basal piece ~ 1/3 length of tegmen; tegmen with sides unevenly widening toward apical fourth, then weakly narrowed to rounded apices; tegmen rather flat, weakly curved in lateral view; weak medioventral process present near middle; median lobe narrow, ~ 1/2 tegmen length, basal apodemes abruptly thinner near bases.

###### Etymology.

By this species name we honor the institution and effort that was the Instituto Nacional de Biodiversidad (INBio), now part of the MNCR. The parataxonomists and professional taxonomists engaged in the effort to document Costa Rica’s biodiversity provided a successful model to which the rest of the world could aspire.

###### Distribution.

This species is only known from Costa Rica.

###### Remarks.

This species is very distinctive, particularly among the Central American Exosternini fauna. Its narrow, uniformly doubly punctate pronotum, broadly oval prescutellar impression, complete outer subhumeral stria with basomedial extension, and basally connected 4^th^ and sutural elytral striae distinguish it from anything else in the region.

##### 16. Phelister sculpturatus

Taxon classificationAnimaliaColeopteraHisteridae

Schmidt, 1893

EAB15608-6F48-56F7-87C6-3039F556B2B7

**Figure 11.**
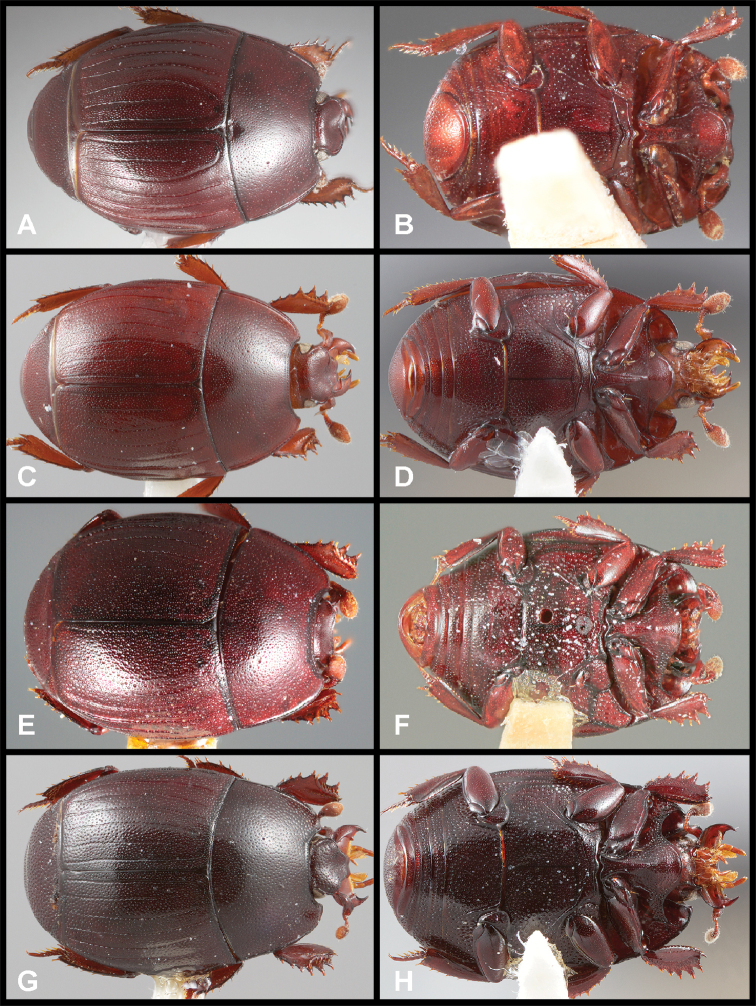
**A, b***Phelister sculpturatus*: **A** dorsal habitus **B** ventral habitus **C, D***P. tunki*: **C** dorsal habitus **D** ventral habitus **E, F***P. praedatoris*: **E** dorsal habitus **F** ventral habitus **G, H***P. ifficus*: **G** dorsal habitus **H** ventral habitus.

**Figure 12.**
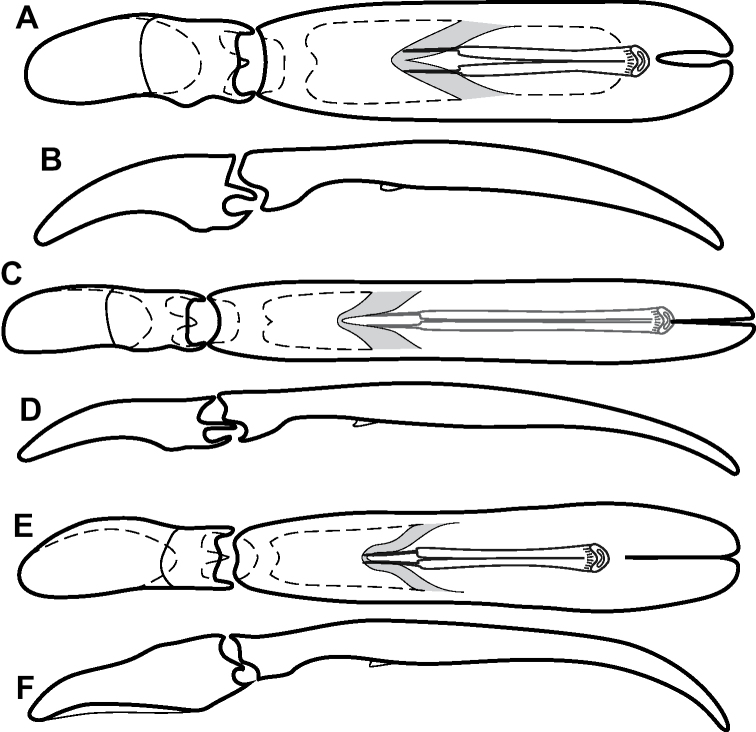
Male genitalia **A, B***Phelister sculpturatus*: **A** aedeagus, dorsal **B** aedeagus, lateral **C, D***P. praedatoris*: **C** aedeagus, dorsal **D** aedeagus, lateral **E, F***P. ifficus*: **E** aedeagus, dorsal **F** aedeagus, lateral.

Figs 11A, B , 12A, B , Map 7

Phelister sculpturatus Schmidt, 1893a: 12.

###### Type material.

***Lectotype***, of undetermined sex, hereby designated: “Mexique” / “coll. J. Schmidt” / “Type” / “sculpturatus Schm.” / “Lectotype Phelister sculpturatus Schmidt, 1893, M.S.Caterino & A.K.Tishechkin des. 2010”; ZMHB.

###### Other material.

**Brazil**: Mato Grosso, Mpio. Cuiaba, Fazenda Mutuca (-15.3145, -55.9703), 11/15/08, FIT, F.H. Gava & J. R. Rocha, EXO-02973 (CEMT, 1ex.); **Costa Rica**: Cartago, P.N. Tapanti, Quebrada Segunda (9.76, -83.79), 1250 m, February 1993, G. Mora, INBIO CRI001 359423 (INBIO, 1ex.); Guanacaste, Cacao Biological Station (10.9272, -85.4519), 1050 m, 7/10/00–7/11/00, FIT, J. Ashe, R. Brooks, Z. Falin, SM0245385 (SEMC, 1ex.); Puntarenas, Est. Biol. Las Cruces (8.7857, -82.9697), 1330 m, 5/28/04–5/31/04, FIT, J. Ashe, Z. Falin, I. Hinojosa, SM0611403 (SEMC, 1ex.); Puntarenas, Est. Sirena, P.N. Corcovado (8.48, -83.59), 0–100 m, October, 1990, C. Saborio, INBIO CRI000 293821 (INBIO, 1ex.); Puntarenas, 11 km S.W. Est. Biol. Las Cruces (8.7786, -83.0306), 1450 m, 7/9/99, wet cloud forest litter, R. Anderson, EXO-02969 (CMNC, 1ex.); Puntarenas, Rancho Quemado, Peninsula de Osa (8.7, -83.6), 200 m, September 1992, M. Segura, INBIO CRI000 907726 (INBIO, 1ex.); **Ecuador**: Orellana, Est. Biodiv. Tiputini (-0.6333, -76.0383), 220 m, 9/5/00–9/25/00, D. Inward & K. Jackson, EXO-02972 (NHMUK, 1ex.); Orellana, Est. Biodiv. Tiputini (-0.6376, -76.1499), 6/4/11–6/9/11, FIT, M. Caterino & A. Tishechkin, EXO-00707 (MSCC, 1ex., DNA voucher); Orellana, Est. Cientifica Yasuní, mid. Rio Tiputini (-0.675, -76.4), 7/18–7/24/08, FIT, A. K. Tishechkin (AKTC, 1ex.); **Guatemala**: Suchitepéquez: Volcán Atitlán, Ref. El Quetzal, 1670 m, 14.55067°N, 91.19235°W, 13–13.vi.2015, Z.Falin & F.Carrillo, FIT, cloud forest (SEMC, 2ex.); **Honduras**: Olancho, La Union, La Muralla (15.1, -86.7), 9/1/94, R. Cordero, EXO-02970 (AKTC, 1ex.); **Mexico**: Veracruz, 3 km S Jalapa (19.5, -96.9), 1370 m, 5/26/91, FIT, J. Ashe, EXO-02971 (SEMC, 1ex.); **Panama**: Canal Zone, Barro Colo. Is. (9.2, -79.8), 1/30/55, *Eciton burchelli* refuse deposit, Colony ‘55 B1, Satary Day, C.W. Rettenmeyer (UCONN, 2ex.); Canal Zone, Barro Colorado I (9.2, -79.8), 5/9/56, E-156RD Berlese, C.W. & M.E. Rettenmeyer, EXO-02976 (UCONN, 1ex.); Canal Zone, Barro Colorado I. (9.2, -79.8), 3/28/56, *Eciton burchelli* Bivouac Site Colony E-132RD, C.W. Rettenmeyer, EXO-02975 (UCONN, 1ex.); Canal Zone, Barro Colorado Island (9.2, -79.8), 3/30/63, refuse deposit, Host: *Eciton hamatum* colony E-321, EXO-02977, UCONN (1ex.); Colon, P.N. San Lorenzo, Achiote (9.2167, -80.1067), 10 m, 5/13/08–5/28/08, FIT, A. Mercado, EXO-02980 (AKTC, 1ex.); Colon, San Lorenzo Forest (9.2833, -79.9667), 10/3/03–10/4/03, FIT, A. Tishechkin, EXO-02979 (AKTC, 1ex.); Colon, San Lorenzo Forest (9.2833, -79.9667), 10/24/03–10/25/03, FIT, A. Tishechkin, EXO-00376 (AKTC, 1ex.); Colon, San Lorenzo Forest (9.2833, -79.9667), 5/12/04–5/13/04, FIT, A. Tishechkin, EXO-02981 (AKTC, 1ex.); Panama, Barro Colorado Isd. (9.1833, -79.85), 7/8/94, FIT, D. Banks (SEMC, 2ex.); Darien, Darien National Park, Camp Pirre, Rancho Frio, 8°1'11"N, 77°43'57"W, 100 m, 6–13.ii.2014, L. G. Bezark (AKTC, 1ex.); **Peru**: Junín, 11 km NE Puerto Ocopa, Los Olivos (-11.05, -74.2587), 1200 m, 3/26/09–3/31/09, FIT, A. Tishechkin (AKTC, 2ex.); Loreto, Iquitos – Nauta rd. km 58, Rio Itaya (-4.2563, -73.4675), 120 m, 5/5/09–5/9/09, FIT, next to entrance into *Eciton burchelli* statary bivouac in a hollow treee (AKTC, 3ex.).

###### Diagnostic description.

Length: 1.73–2.09 mm (avg. 1.85 mm); width: 1.54–1.77 mm (avg. 1.64 mm). Body elongate oval, convex, dark rufescent, with densely intermingled ground and secondary punctation throughout; frons rather wide, depressed along midline; supraorbital stria present at middle, obsolete at sides; frontal stria complete; epistoma obliquely subcarinate along sides; labrum emarginate and subcarinate apically; mandibles each with small basal tooth; antennal club with elongate setose patch on dorsal surface; prescutellar impression wide, forming a short rounded triangle 2–3 × as wide as scutellum; median pronotal gland openings present ~ 2/3 back from anterior margin, distinctly annulate; pronotal punctation doubled throughout, but more sparsely so in middle third; marginal pronotal stria complete along sides and front; submarginal pronotal stria complete, raised, close to margin, barely turning anterior corner; elytron with two complete epipleural striae; outer subhumeral stria complete, inner subhumeral nearly complete, obsolete at base; dorsal striae 1–4 complete, the 4^th^ arched to and usually connected to the sutural, 5^th^ slightly abbreviated from base, sutural stria complete or nearly so; prosternal keel narrow, emarginate at base, striae separate at base, evenly converging to near apex, united anteriorly; prosternal lobe shortly, weakly reflexed, with marginal stria complete; mesoventrite produced, with complete marginal stria continued at sides by postmesocoxal stria, ending behind mesocoxa; mesometaventral stria angulate at middle, crenulate, reaching middle of mesoventrite, continued at sides by lateral metaventral stria nearly reaching outer 1/3 of metacoxa; 1^st^ abdominal ventrite with incomplete lateral stria along inner margin of metacoxa; protibia with outer margin strongly dentate, with ~ five marginal spines; protarsal setae of male not flattened; meso- and metatibiae slightly broadened, with a few spines along outer margins. Male: basal piece nearly 1/2 length of tegmen; tegmen narrow at base, expanded then subparallel over most of length, apices rounded, slightly separated; tegmen flattened, slightly curved in lateral view; medioventral process present, projecting at basal fourth; median lobe ~ 1/2 tegmen length, basal apodemes abruptly narrowed at bases.

###### Distribution.

This species ranges from southeastern Mexico, through Central America to south-central Brazil.

###### Remarks.

This widespread and variable species has much in common with *P. praedatoris*, especially in the nearly complete coverage of ground and secondary punctation. However, *P. sculpturatus* always has its inner elytral striae distinct amongst the punctures, is slightly more elongate in overall body form, and has the protibia less strongly dentate. The distributions of the two species don’t appear to overlap, with *P. sculpturatus* known only from cerrado and western Amazonia in South America (beyond its more typical range in Central America). Like *P. praedatoris*, it appears to be an army ant associate, having been collected a few times (in both Peru and Panama) in *Eciton* bivouacs, though most records come only from flight interception traps.

##### 17. Phelister tunki sp. nov.

Taxon classificationAnimaliaColeopteraHisteridae

01490209-F7AD-5FB9-9AEB-B54AC028BB7D

http://zoobank.org/928A79D3-9205-4386-B061-30F14B0B8C0F

Fig. 11C, D , Map 7

###### Type material.

***Holotype* female**: “**Peru**: Dept. Cusco: Cock of the Rock Lodge, NE Paucartambo 13°03.3'S, 71°32.7'W 1120 m, 4–9-XI-2007, D.Brzoska, ex. Flight intercept trap, PER1B07 001” / “SEMC0870736 KUNHM-ENT” (SEMC).

###### Diagnostic description.

Length: 2.25 mm; width: 2.05 mm. This species is extremely similar to the preceding, and differs in only a few unusual characters: body larger; median pronotal gland openings nearer halfway back from anterior margin; frontal stria obsolete in median depression, frons with distinct microsculpture; antennal club with two distinct dorsal and one ventral setal sensory patch basal to elongate median patch (Fig. 11C); propygidium more coarsely and densely punctate; protibia slightly broader; lateral metaventral stria divergent toward mesepimeron. Male not known.

###### Etymology.

This species name, *tunki*, is the local name of the Andean cock-of-the-rock (*Rupicola peruvianus*), a beautiful and popular bird in the area, for which the lodge at the type locality was named.

###### Distribution.

This species is only known from the type locality, Cusco, Peru.

###### Remarks.

Although this species is known only from a singleton, from a locality on the margin of the range of its closest relative *P. sculpturatus*, there are enough clear morphological differences between the two to support its separate species status.

##### 18. Phelister praedatoris

Taxon classificationAnimaliaColeopteraHisteridae

Reichensperger, 1939

9248F2CC-D8FC-59A2-A6FF-6BE9FF2A667B

**Map 8.**
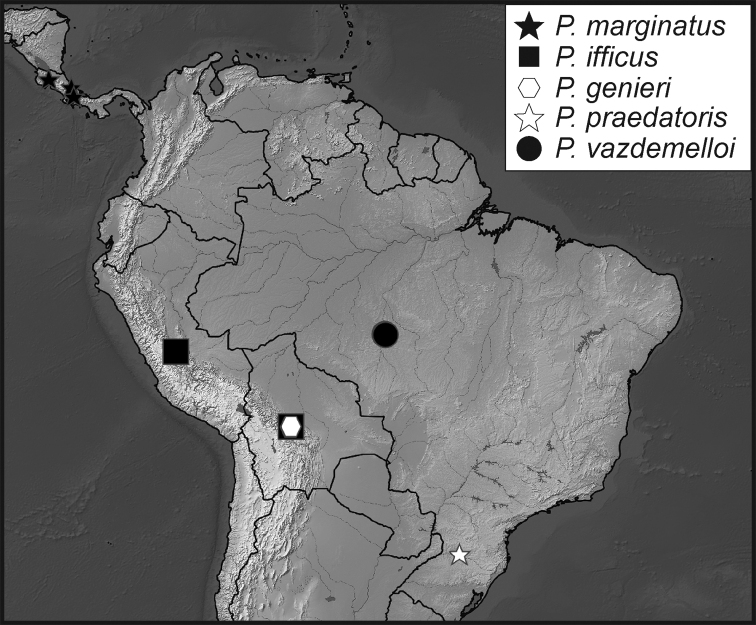
Collecting records for *Phelister vazdemelloi* (black circles), *P. praedatoris* (white star), *P. ifficus* (black squares), *P. genieri* (hexagon), and *P. marginatus* (black stars).

Figs 11E, F , 12C, D , Map 8

Phelister praedatoris Reichensperger, 1939: 281.
Phelister praedatorius Mazur, 1984: 284, 1997: 28, 2011: 28 (misspelling).

###### Type material.

***Lectotype***, hereby designated: “F. Plaumann Nova Teutonia Brasilien” / “Eciton praedator” / “Phelister praedatoris Reichensp.”; Bonn (not seen). ***Paralectotypes*** (3), hereby designated: same data as type (CHND, FMNH-INS0000069298 and FMNH-INS0000069356, examined by the authors).

###### Other material.

**Brazil**: Santa Catarina, Nova Teutonia (-27.1833, -52.3833), 22.vi.1939, F. Plaumann, *E. praed*. , EXO-00005 (NHMUK, 1ex.); Santa Catarina, Nova Teutonia (-27.1833, -52.3833), i[?].14.193[?], F. Plaumann, (FMNH-INS0000069239, 1ex.); Santa Catarina, Nova Teutonia (-27.1833, -52.3833), x.-.1939, F. Plaumann, (FMNH, 1ex.); Santa Catarina, Nova Teutonia (-27.1833, -52.3833), 1953, F. Plaumann, FMNH-INS 0000 069 305 (FMNH, 2ex.); Santa Catarina, Nova Teutonia (-27.1833, -52.3833), May 1953, F. Plaumann, EXO-02984 (FMNH, 1ex.); Santa Catarina, Nova Teutonia (-27.1833, -52.3833), April 1952, F. Plaumann, Host: *Labidus praedator* (FMNH, 3ex.); Santa Catarina, Nova Teutonia (-27.1833, -52.3833), 1951, F. Plaumann, Eciton (Lab.) praedator (FMNH, 2ex.); Santa Catarina, Nova Teutonia (-27.1833, -52.3833), ii.1959, F. Plaumann, with *Eciton* prey [maybe an error in transcription?] (FMNH, 9ex.); Santa Catarina, Nova Teutonia (-27.1833, -52.3833), no date, F. Plaumann (FMNH, 1ex.).

###### Diagnostic description.

Length: 1.97–2.52 mm (avg. 2.18 mm); width: 1.69–2.13 mm (avg. 1.92 mm). Body elongate oval, convex, dark rufescent, with densely intermingled ground and secondary punctation throughout; frons rather wide, moderately depressed along midline; supraorbital stria present at middle, obsolete at sides; frontal stria complete; epistoma subcarinate along sides; labrum emarginate and subcarinate apically; mandibles each with small basal tooth; antennal club with elongate setose patch on dorsal surface; prescutellar impression small, forming an oval ~ equal in size to scutellum; median pronotal gland openings present ~ 3/4 from anterior margin, distinctly annulate, vaguely obscured by punctures; marginal pronotal stria complete along sides and front; submarginal pronotal stria complete, close to margin, barely turning anterior corner; elytron with single, complete epipleural stria; outer subhumeral stria complete, but may be interrupted at middle, inner subhumeral nearly complete, obsolete at base; dorsal striae one and two usually complete, 3^rd^ present in basal 1/2 only, 4^th^ and 5^th^ largely or completely absent, though a basal arch of the 4^th^ may be traceable, sutural stria may be traceable in apical 1/2; prosternal keel narrowed, emarginate at base, striae separate at base, converging and subparallel to near apex, united anteriorly; prosternal lobe reflexed, with marginal stria complete or obsolete at middle; mesoventrite produced, with complete marginal stria continued at sides by postmesocoxal stria, which ends behind mesocoxa; mesometaventral stria angulate at middle crenulate, reaching middle of mesoventrite, continued at sides by lateral metaventral stria toward but not reaching middle of metacoxa; 1^st^ abdominal ventrite with faint, incomplete lateral stria along inner margin of metacoxa; protibia with outer margin strongly dentate, with ~ five marginal spines; protarsal setae of male not flattened; meso- and metatibiae slightly broadened, with a few spines along outer margins. Male: aedeagus very narrow, basal piece ~ 1/3 length of tegmen; tegmen with sides subparallel over most of length, weakly narrowed at middle, apices rounded; tegmen slightly curved in lateral view; medioventral process present, projecting at basal 1/3; median lobe ~ 2/3 tegmen length, basal apodemes abruptly narrowed at bases.

###### Distribution.

While the species is well represented in collections, it seems still only to be known from the type locality (in Santa Catarina, Brazil), and only in association with army ants (both *Labidus* and *Eciton* are recorded as hosts).

###### Remarks.

*Phelister praedatoris* was one of the first species in this group to be described, and it remains among the most distinctive, with its inner elytral striae largely obliterated by dense secondary elytral punctation. Few other species treated here are as thoroughly doubly punctate, including on the thoracic and abdominal ventrites, the principal exceptions being the species treated immediately below. Additional distinctive characters include its unusually strongly dentate protibiae, and a raised outer subhumeral stria that almost forms a lateral elytral margin (aligned with the strongly raised marginal pronotal bead).

##### 19. Phelister ifficus sp. nov.

Taxon classificationAnimaliaColeopteraHisteridae

9F261646-008A-5417-9EFD-78F56C987C6E

http://zoobank.org/51DDD357-0476-4F11-8B7B-5481761978EC

Figs 11G, H , 12E, F , Map 8

###### Type material.

***Holotype* male**: “**Bolivia**: Cochabamba, 117 km E Cochabamba, at Lagunitas, 1000 m, 17°06'22"S, 65°40'57"W, 1–6.II.1999, F. Génier, mountain evergreen forest, ex. f.i.t. 99-029” / “Caterino/Tishechkin Exosternini Voucher EXO-00001” (CMNC). ***Paratype* (2): Peru**: Junín: ~ 16 km NW Satipo, Rio Venado, 11°11.677'S, 74°46.137'W, 1150 m, 3–8. March 2010, A.V. Petrov, Caterino/Tishechkin Exosternini Voucher EXO-02993 (AKTC, 1ex.); Junín, ~ 15 km NW Satipo, nr. Rio Venado at 11°11.18'S, 74°46.02'W, 1100–1400 m, 4/20/19–5/18/19, A. V. Sokolov (AVSC, 1ex.).

###### Diagnostic description.

Length: 3.27–3.43 mm (avg. 3.35 mm); width: 2.80–2.84 mm (avg. 2.82 mm). Body elongate oval, slightly depressed, dark rufescent, with conspicuous double punctation throughout, the ground punctation coarse and uniformly intermingled with moderately large secondary punctures; frons rather broad, depressed along midline; supraorbital stria present at middle; frontal stria interrupted at middle; epistoma subcarinate along sides; labrum emarginate and subcarinate apically; mandibles each with small basal tooth; antennal club elongate, with small setose patch near apex of dorsal surface; prescutellar impression small and weak; median pronotal gland openings obscured by punctures, but distinctly annulate and present about midway from anterior margin; marginal pronotal stria complete around lateral and anterior margins; lateral submarginal pronotal stria complete, pronotal disk weakly impressed along its inner edge; elytron with single, complete epipleural stria; outer subhumeral stria interrupted at middle, inner absent; all dorsal striae finely impressed as a series of punctures, complete, with 4^th^ arched to sutural, 5^th^ not quite attaining basal arch; pygidia densely punctate; prosternal keel emarginate at base, striae separated at base, sinuate to united apex; prosternal lobe rounded, with marginal stria complete; mesoventrite produced, its marginal stria complete, continued by postmesocoxal stria toward anterior third of mesepimeron; mesometaventral stria weakly impressed, bluntly angulate at middle; lateral metaventral stria diverging to side, barely separated from postmesocoxal; 1^st^ abdominal ventrite with incomplete lateral stria along inner edge of metacoxa; all ventrites punctate; all tibia slightly broadened; protibia with outer margin strongly dentate, bearing four or five marginal spines; protarsal setae of male not flattened; meso- and metatibiae slightly broadened, mesotibia with several conspicuous spines along outer margins, those of metatibia fewer and finer. Male: basal piece 1/3 length of tegmen; tegmen narrow at base, weakly and unevenly expanded to near apex, apices rounded; tegmen flattened, mostly straight but apically curved in lateral view; medioventral process present, projecting at basal fourth; median lobe ~ 1/2 tegmen length, basal apodemes abruptly narrowed at bases.

###### Etymology.

This species name is a play on the full binomial, together forming -*ter ifficus*, in reference to its attractive morphology.

###### Distribution.

This species only known from two localities, in the Andean foothills of central Peru and Bolivia.

###### Remarks.

This species seems to be part of a monophyletic lineage, also comprising the following three species. *Phelister ifficus* is much more densely punctate than the most similar of them, *P. vazdemelloi*, as well as more elongate, yet not markedly flattened or laterally explanate like *P. genieri* and *P. marginatus* are. The two available specimens of this species differ slightly in the appearance of the prescutellar area, the Peruvian specimen having a very weak depression, and the Bolivian one having only a tiny smooth area with a puncture.

##### 20. Phelister genieri sp. nov.

Taxon classificationAnimaliaColeopteraHisteridae

5B3850CE-4688-521E-AF96-E55213259B07

http://zoobank.org/D5C97694-8DF0-41F5-AC4B-474C29AE092D

**Figure 13.**
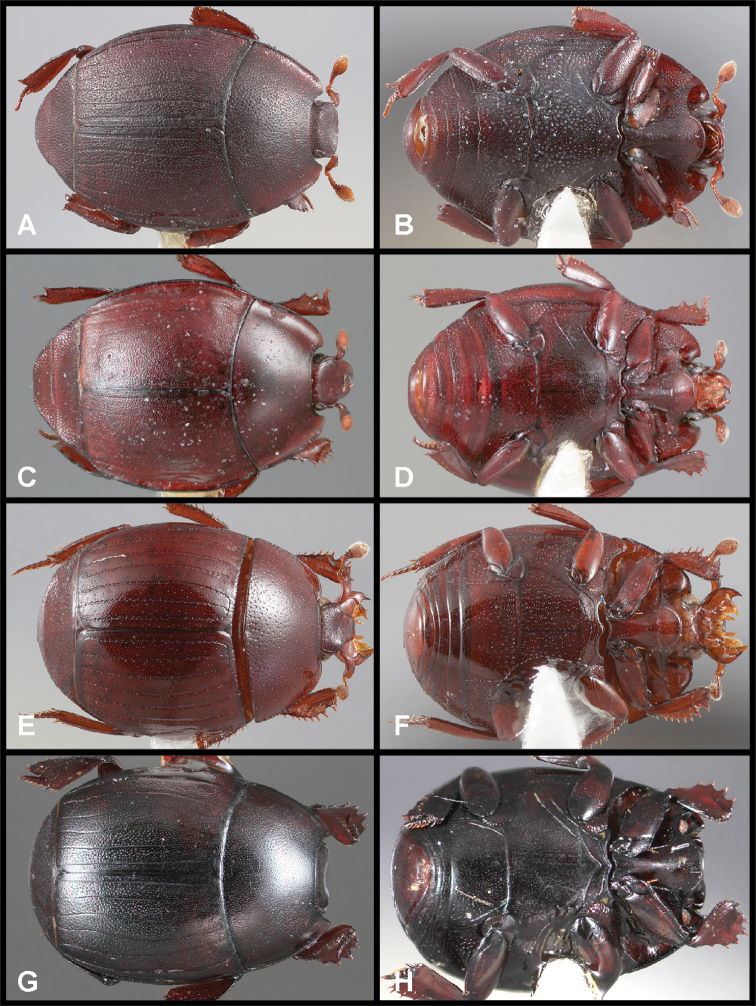
**A, B***Phelister genieri*: **A** dorsal habitus **B** ventral habitus **C, D***P. marginatus*: **C** dorsal habitus **D** ventral habitus **E, F***P. vazdemelloi*: **E** dorsal habitus **F** ventral habitus **G, H***P. dilatatus*: **G** dorsal habitus **H** ventral habitus.

**Figure 14.**
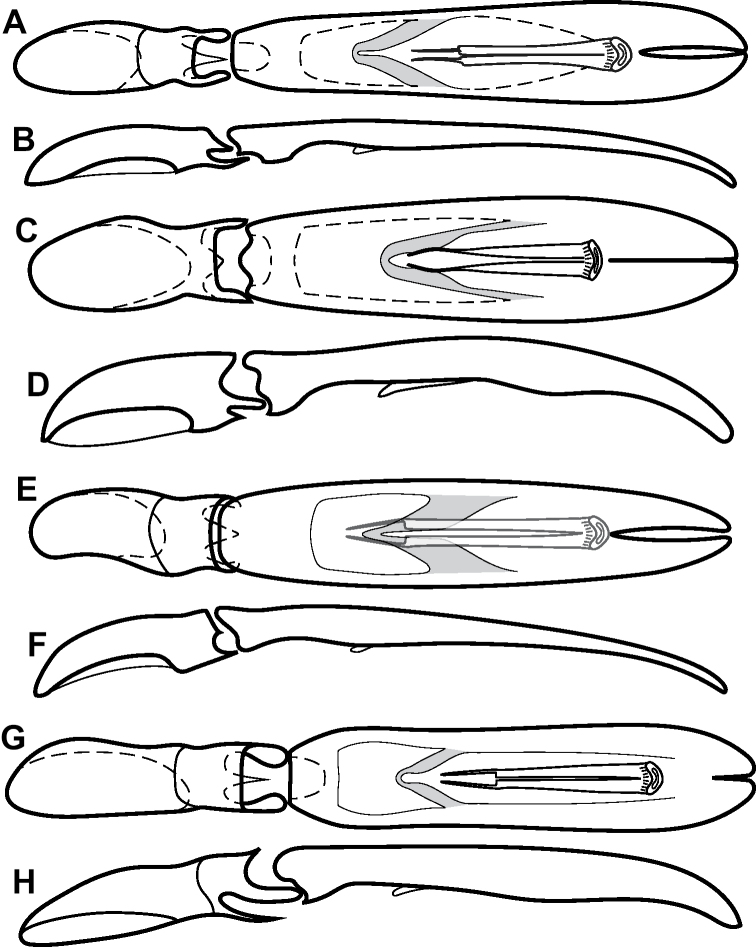
Male genitalia **A, B***Phelister genieri*: **A** aedeagus, dorsal **B** aedeagus, lateral **C, D***P. marginatus*: **C** aedeagus, dorsal **D** aedeagus, lateral **E**, **F***P. vazdemelloi*: **E** aedeagus, dorsal **F** aedeagus, lateral **G, H***P. dilatatus*: **G** aedeagus, dorsal **H** aedeagus, lateral.

Figs 13A, B , 14A, B , Map 8

###### Type material.

***Holotype* male**: “**Bolivia**: Cochabamba, 105 km E Cochabamba, at Rio Carmen Mayo, 1800 m, 17°08'47"S, 65°43'55"W [17.1464, -65.7319], 6–8.II.1999, F. Génier, low yungas forest, ex. f.i.t. 99-033” / “Caterino/Tishechkin Exosternini Voucher EXO-00379” (CMNC). ***Paratype* (1)**: Cochabamba, 117 km E Cochabamba at Lagunitas (-17.1061, -65.6825), 1000 m, 2/8/99–2/12/99, FIT, mountain evergreen forest, F. Génier, EXO-02994 (CMNC, 1ex.).

###### Diagnostic description.

Length: 2.52–2.76 mm (avg. 2.64 mm); width: 2.21–2.40 mm (avg. 2.30 mm). Body broadly rounded, depressed, sides explanate, rufescent, with very dense and conspicuous ground punctation throughout; frons rather broad, depressed along midline; supraorbital stria absent; frontal stria complete; epistoma subcarinate along sides; labrum emarginate and subcarinate apically; mandibles each with small basal tooth; antennal club elongate, with small setose patch near apex of dorsal surface; prescutellar impression absent; median pronotal gland openings small, obscured by punctures, but present ~ 2/3 from anterior margin; marginal pronotal stria complete, fine, continued around anterior margin; submarginal pronotal stria present close to marginal as a raised carina, disk deeply depressed along its inner edge; elytron with single, complete epipleural stria; outer subhumeral stria complete beneath strong lateral elytral carina, which may be the complete inner subhumeral stria; dorsal striae shallowly impressed but distinct, all complete, with 4^th^ arched to sutural; pygidia densely covered with elongate punctures, pygidium weakly differentiated, with flat anterodorsal surface and short, curved posteroventral surface, similarly punctate throughout; prosternal keel slightly depressed, emarginate at base, striae obsolete; prosternal lobe weakly reflexed, with marginal stria complete; mesoventrite produced, its marginal stria weakly impressed, continued by postmesocoxal stria toward but not reaching middle of mesepimeron; mesometaventral stria weakly impressed, angulate at middle; lateral metaventral stria diverging to side, subparallel to postmesocoxal; 1^st^ abdominal ventrite with incomplete lateral stria along inner edge of metacoxa; all ventrites punctate; all tibia slightly broadened; protibia with outer margin strongly dentate, bearing four or five marginal spines; protarsal setae of male not flattened; meso- and metatibiae slightly broadened, with a few spines along outer margins. Male: basal piece 1/3 length of tegmen; tegmen narrow at base, weakly and unevenly expanded to near apex, apices rounded; tegmen quite flattened, only weakly curved in lateral view; medioventral process present, projecting at basal fourth; median lobe almost 1/2 tegmen length, basal apodemes abruptly narrowed at bases.

###### Etymology.

We are pleased to name this species in honor of François Génier, collector of the type, and contributor of numerous valuable specimens to our histerid studies.

###### Distribution.

This species is only known from the type locality, Cochabamba, Bolivia.

###### Remarks.

As described above, this species is part of a related lineage comprising the preceding one and following two species. It is particularly close to *P. marginatus* but is easily distinguished by the much coarser dorsal punctation, and distinct elytral striae. Its body is also not so broad, and its sides less broadly explanate.

##### 21. Phelister marginatus sp. nov.

Taxon classificationAnimaliaColeopteraHisteridae

76CCE646-7C25-5915-A432-A5702476274D

http://zoobank.org/205FD7E7-C774-46CD-85A3-812371CA5D9A

Figs 13C, D , 14C, D , Map 8

###### Type material.

***Holotype* male**: “**Costa Rica**: Puntarenas, Monte Verde [10.3, -84.8], 1520 m, 4 May 1989, J. Ashe, R. Brooks, R. Leschen, ex. flight intercept trap” / “SEMC0903646 KUNHM-ENT (SEMC). ***Paratypes* (2): Costa Rica**: Limon, Valle de la Estrella, R.B. Hitoy Cerere, Camino a S. Toma de Agua (9.67, -83.02), 160 m, 9/30/99–10/9/99, W. Arana, INB0003142728 (INBIO, 1ex.); Puntarenas, Est. Biol. Las Alturas (8.9362, -82.8335), 1660 m, 5/31/04–6/3/04, FIT, J. Ashe, Z. Falin, I. Hinojosa, SM0606778 (SEMC, 1ex.).

###### Diagnostic description.

Length: 2.64–2.80 mm (avg. 2.71 mm); width: 2.48–2.56 mm (avg. 2.52 mm). Body broadly rounded, depressed, sides explanate, rufescent, with very dense and conspicuous ground punctation throughout; frons rather broad, moderately depressed along midline; supraorbital stria absent; frontal stria interrupted across median depression; epistoma subcarinate along sides; labrum emarginate and subcarinate apically; mandibles each with small basal tooth; antennal club elongate, with small setose patch near apex of dorsal surface; prescutellar impression absent, at most faintly depressed; median pronotal gland openings small, obscured by punctures, but present ~ 2/3 from anterior margin; marginal pronotal stria complete, fine, continued around anterior margin; submarginal pronotal stria present close to marginal as a raised carina, disk deeply depressed along its inner edge; elytron with single, complete epipleural stria; outer subhumeral stria complete beneath strong lateral elytral carina, which may be the complete inner subhumeral stria; dorsal striae faintly impressed, only a few vaguely traceable, primarily the complete sutural stria; propygidium flat, densely covered with elongate punctures, pygidium with flat anterodorsal surface punctate, and short, curved posteroventral surface impunctate but shagreened; prosternal keel narrowed, emarginate at base, striae narrowly separate at base, converging and subparallel to near apex, united anteriorly; prosternal lobe reflexed, with marginal stria complete; mesoventrite produced, its marginal stria only faintly impressed; postmesocoxal stria present, not reaching mesepimeron; mesometaventral stria absent across middle; lateral metaventral stria forming a short stria parallel to postmesocoxal; all ventrites punctate; all tibia slightly broadened; protibia with outer margin strongly dentate, bearing four or five marginal spines; protarsal setae of male not flattened; meso- and metatibiae slightly broadened, with a few spines along outer margins. Male: basal piece 1/3 length of tegmen; tegmen narrow at base, evenly widened, rounded to near apex, apices rounded; tegmen curved to apex in lateral view; medioventral process present, projecting at basal fourth; median lobe ~ 1/3 tegmen length, basal apodemes abruptly narrowed at bases.

###### Etymology.

The name of this species refers to the carinate margins of the pronotum and elytra.

###### Distribution.

This species is known only from Costa Rica, from Limón and Puntarenas provinces.

###### Remarks.

As described above, this species is part of a related lineage comprising the preceding two and the following species. *Phelister marginatus* is easily distinguished by its broad, flattened, laterally carinate and explanate body, along with its largely effaced elytral striae.

##### 22. Phelister vazdemelloi sp. nov.

Taxon classificationAnimaliaColeopteraHisteridae

1B829FA5-998A-581F-94C2-130E34DC6D03

http://zoobank.org/FC0FAC03-AEBC-490F-BB20-B6B7BE340218

Figs 13E, F , 14E, F , Map 8

###### Type material.

***Holotype* male**: “**Brazil**: Mato Grosso, Mpio. Cotriguaçu, Fazenda São Nicolau, Prainha. -9°51.6'S, -58°12.9'W [-9.86, -58.215], Flight intercept, Oct 2009, F.Z. Vaz-de-Mello” / “Caterino/Tishechkin Exosternini Voucher, EXO-00853” (CEMT). ***Paratypes* (6): Brazil**: Mato Grosso, Mpio. Cotriguaçu, Fazenda São Nicolau (-9.815, -58.2858), 12/15/10–12/18/10, FIT, F.Z. Vaz-de-Mello & A.F. Oliveira (CEMT, 2ex.); Mato Grosso, Mpio. Cotriguaçu, Fazenda São Nicolau, Mata Norte (-9.8192, -58.26), 12/8/10–12/14/10, FIT, F.Z. Vaz-de-Mello (FMNH & MSCC, 2ex.); Mato Grosso, Mpio. Cotriguaçu, Fazenda São Nicolau, Matinha (-9.8383, -58.2508), December 2010, FIT, F.Z. Vaz-de-Mello (AKTC & CHND, 2ex.).

###### Diagnostic description.

Length: 2.05–2.44 mm (avg. 2.25 mm); width: 1.85–2.29 mm (avg. 2.05 mm). Body rather broadly rounded, widest behind elytral humeri, rufescent, with conspicuous ground punctation throughout, the pronotum with uniformly dense secondary punctation as well; frons depressed along midline; frontal stria obsolete within median depression; epistoma broad, with raised edges along sides and front; labrum emarginate, weakly subcarinate along apical margin; mandibles lacking basal teeth; prescutellar impression not evident; median pronotal gland openings only slightly larger than secondary punctures, ~ 1/3 behind anterior margin, distinctly annulate; marginal pronotal stria complete along lateral and anterior margins; submarginal stria complete, well-impressed along lateral margin, just turning anterior corner; elytron with single, complete epipleural stria; outer subhumeral stria present at base and apex but interrupted at middle; inner subhumeral stria present at middle but obsolete at ends; all dorsal striae complete, 4^th^ arched to sutural, with 5^th^ nearly meeting basal arch; pygidia with moderately coarse secondary punctation; prosternal keel emarginate at base, striae separate at base, converging, meeting ¾ of the distance to apex, united anteriorly; prosternal lobe weakly reflexed, with marginal stria complete; mesoventrite weakly produced, with complete marginal stria continued at sides by postmesocoxal stria ending laterad mesocoxa; mesometaventral stria angulate at middle, crenulate, reaching middle of mesoventrite, continued at sides by lateral metaventral stria, reaching middle of metacoxa; 1^st^ abdominal ventrite with nearly complete lateral stria along inner margin of metacoxa; metaventrite with coarse secondary punctation; 1^st^ abdominal ventrite with secondary punctures largely limited to anterior third; protibia with outer margin weakly dentate, with five or six robust marginal spines; meso- and metatibiae slender, the mesotibia spinose along most of outer margin, metatibia with marginal spines finer and restricted to apical 1/2. Male: basal piece 1/3 length of tegmen; tegmen narrow at base, widest at basal 1/3, then rounded, narrowing to apex, apices rather thin, slightly separated; tegmen very flattened, straight with weak apical curve in lateral view; medioventral process present, projecting at basal fourth; median lobe ~ 1/2 tegmen length, basal apodemes abruptly narrowed at bases.

###### Etymology.

This species is named in honor of our friend and colleague, Fernando Vaz-de-Mello. Fernando’s lab and the collection he runs (CEMT) have provided many specimens to our studies, and he and his students graciously hosted the authors during a 2011 field trip to Mato Grosso.

###### Distribution.

This species is only known from Mato Grosso, Brazil.

###### Remarks.

This species and the preceding three (*P. marginatus*, *P. genieri*, and *P. ifficus*) form a closely related lineage within this group. All have lost a distinct prescutellar impression and are considerably larger in body size than average for the group. All also have more or less ‘complete’ elytral striation, uniformly double (ground and secondary) punctation on the dorsum, and have slightly expanded and strongly punctate protibiae. *Phelister vazdemelloi* is the least modified of these, with relatively sparse dorsal punctation, and a convex body.

##### 23. Phelister dilatatus sp. nov.

Taxon classificationAnimaliaColeopteraHisteridae

C209F862-5471-59E4-A9B1-07198749D5FB

http://zoobank.org/8D3A519B-0ED5-4FE1-BA9A-F7D2EF91318A

**Map 9.**
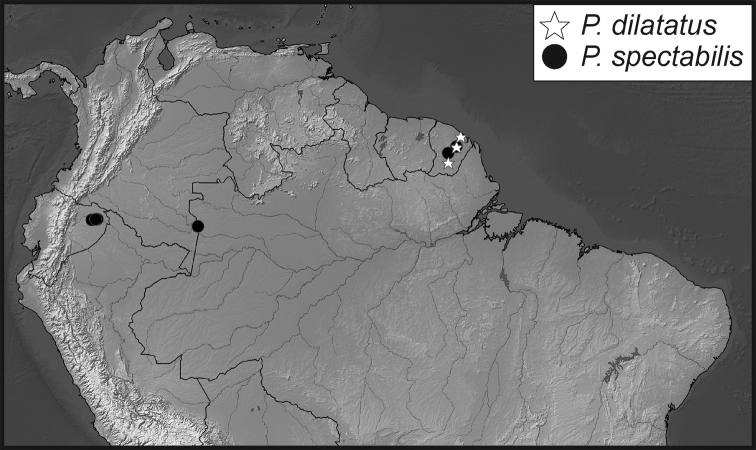
Collecting records for *Phelister dilatatus* (stars), and *P. spectabilis* (circles).

Figs 13G, H , 14G, H , Map 9

###### Type material.

***Holotype* male**: “**Guyane Française**: Montagne des Chevaux 4°43'N, -52°24'W [4.7167, -52.4] piège d’interception 27 Juin 2009. SEAG leg.” / “Caterino/Tishechkin Exosternini Voucher EXO-00370 (MNHN). ***Paratypes* (9): French Guiana**: Régina, Rés. Natur. des Nouragues, Camp Inselberg (4.0833, -52.6833), 9/22/10, FIT, SEAG, EXO-02991 (CHND, 1ex.); Régina, Rés. Natur. des Nouragues (4.0378, -52.6725), 8/4/09, FIT, SEAG, EXO-02990 (CHND, 1ex.); Belvédère de Saül, point de vue (3.6228, -53.2094), 12/10/10, FIT, SEAG, EXO-02992 (CHND, 1ex.); Montagne des Chevaux (4.7167, -52.4), 5/2/09, FIT, SEAG, EXO-02988 (CHND, 1ex.); Montagne des Chevaux (4.7167, -52.4), 5/16/09, FIT, SEAG, EXO-02989 (CHND, 1ex.); Montagne des Chevaux (4.7167, -52.4), 6/13/09, FIT, SEAG, EXO-03041 (CHND, 1ex.); Bélizon. 4.2769, -52.6435. 45m. Jan 2017. J.L..Guiglaris (CHND, 1ex.); Régina, Rés. Natur. des Nouragues (4.0378, -52.6725), 4.vii.2009, FIT, SEAG (CHND, 1ex.); Régina, Rés. Natur. des Nouragues (4.0378, -52.6725), 11.vii.2010, FIT, SEAG (CHND, 1ex.).

###### Diagnostic description.

Length: 2.96–3.51 mm (avg. 3.21 mm); width: 2.56–3.84 mm (avg. 2.70 mm). Body large, elongate oval, strongly convex, castaneous to piceous; most surfaces conspicuously punctate; head with frons depressed along midline, produced above antennal bases, ground punctures not reticulate; supraorbital stria absent; frontal stria complete, continued above epistoma, also apparently continued anterad by lateral marginal epistomal carina; epistoma not constricted at base, elevated along anterior margin, labrum short, apex weakly emarginate; mandibles with incisor edges rather short, each with weak basal marginal tooth; pronotum broad, uniformly densely punctate; distinct prescutellar impression absent, prescutellar area only faintly depressed; median pronotal gland openings simple, not annulate, located ~ 2/3 from anterior margin; lateral submarginal stria complete, deeply impressed; lateral marginal stria complete and continuous with anterior marginal; elytron with one complete epipleural stria and a secondary epipleural striole between it and the margin over the metafemur; outer subhumeral striae complete, with outer basal appendix (i.e., appearing y-shaped); inner subhumeral slightly abbreviated from base; all other dorsal striae complete, 5^th^ stria arched to sutural; punctures of elytral disk distinctly interconnected by fine reticulations; propygidium large, midline length equal to that of pygidium; prosternal keel emarginate at base, with primary striae anteriorly connected ~ 1/3 short of presternal suture, divergent at base; secondary striae present along basal 1/2 of keel; prosternal lobe rounded, with complete marginal stria; mesoventrite with strong median projection; marginal mesoventral stria may be weakly interrupted at middle, continued by postmesocoxal stria nearly to metepisternum; mesometaventral stria strongly angulate at middle, extending anteriad to anterior third of mesoventrite, extending to mesocoxa at sides, not directly connected by lateral metaventral stria, which extends posterolaterally to posterior third of metepisternum; 1^st^ abdominal ventrite with single, incomplete lateral stria along inner edge of metacoxa; all tibiae (but not femora) broadly expanded; protibia with inner edge straight, outer edge deeply toothed, with five marginal spines; tarsi compressed, the tarsomeres together little longer than the tibiae are broad. Male: basal piece long, > 1/2 length of tegmen; tegmen widest near base, narrowed at basal 1/3, then weakly widening to apex, apices rounded; tegmen rather thick, mostly straight with weak apical bend in lateral view; medioventral process present, projecting at basal fourth; median lobe ~ 1/2 tegmen length, basal apodemes abruptly narrowed at bases.

###### Etymology.

This species name refers to its broad, ‘dilated’ tibiae.

###### Distribution.

This species is only known from French Guiana.

###### Remarks.

This is a distinctive species, superficially quite similar to *P. amazoniae* and *P. arcuatus* in the large convex body size with expanded tibiae (which we assert to be convergences). But the non-explanate pronotum and ‘normal’, non-expanded femora of the present species will easily separate it from the others. Its anteriorly split (y-shaped) outer subhumeral stria is unique among species treated in this paper.

##### 24. Phelister spectabilis sp. nov.

Taxon classificationAnimaliaColeopteraHisteridae

0788235E-0AAE-5AD2-B847-8272076F6CF8

http://zoobank.org/87349FF2-C9E6-4381-AE82-35F75E313B2D

**Figure 15.**
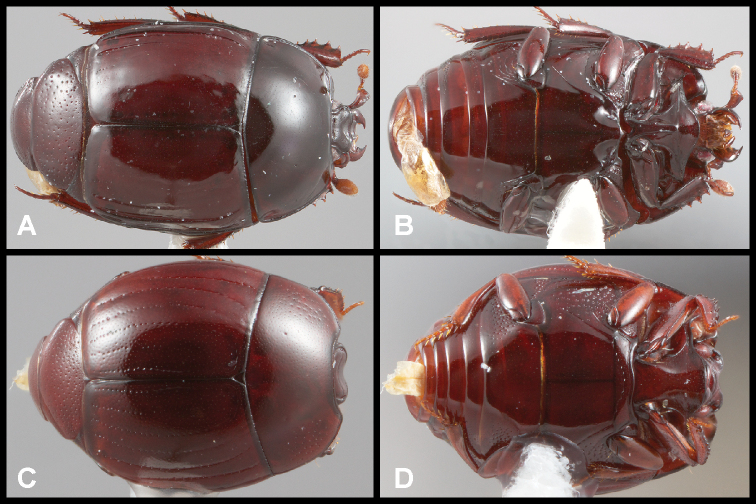
**A, B***Phelister spectabilis*: **A** dorsal habitus **B** ventral habitus **C, D***P. pervagatus*: **C** dorsal habitus **D** ventral habitus.

**Figure 16.**
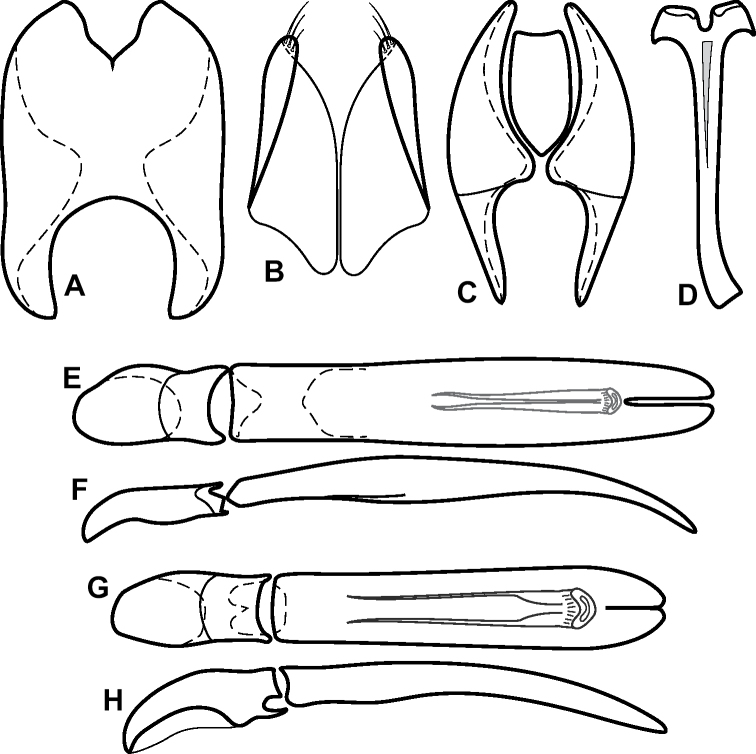
Male genitalia **A–F***Phelister spectabilis*: **A** tergite 8 **B** sternite 8 **C** tergites 9 & 10 **D** sternite 9 **E** aedeagus, dorsal **F** aedeagus, lateral **G, H***P. pervagatus*: **G** aedeagus, dorsal **H** aedeagus, lateral.

Figs 15A, B , 16A–F , Map 9

###### Type material.

***Holotype* male**: “**Ecuador**: Orellana, P.N. Yasuní, Est. Cienc. Yasuní 0°40.5'N, 76°24'W [-0.675, -76.4]. Flight intercept FIT#6-6. 17–19.vii.2008, A.K.Tishechkin AT924” / “Caterino DNA Voucher, Extraction: MSC-2187, Species: Enigm nr.dubEcu, Extraction Date: viii.16.2011” / “Caterino/Tishechkin Exosternini Voucher EXO-00697” (FMNH). ***Paratypes* (4): Ecuador**: Orellana, Est. Cientifica Yasuní (-0.675, -76.4), 7/19/08–7/24/08, FIT, A. Tishechkin, EXO-00697 (AKTC, 1ex.); Orellana, Est. Biodiv. Tiputini (-0.6333, -76.0383), 220 m, 9/5/00–9/25/00, D. Inward & K. Jackson (NHMUK, 2ex.); Orellana, Est. Biodiv. Tiputini (-0.6367, -76.1483), 7/27/08–7/31/08, FIT, A. Tishechkin, EXO-00808 (AKTC, 1ex.).

###### Other material.

**Colombia**: Vaupes, Est. Biol. Caparu, Rio Apoporis (-1.1, -69.5), 9/27/95–12/1/95, FIT, Black-water terrace forest on sandy soils, B. Gill, EXO-00377 (AKTC, 1ex.); **French Guiana**: Régina, Rés. Natur. des Nouragues, Camp Inselberg (4.0833, -52.6833), 1/25/11, FIT, SEAG, EXO-02983 (CHND, 1ex.); Saül, 7 km N, 3 km NW Les Eaux Claires, Mt. La Fumée (3.6628, -53.2219), 490 m, 6/1/97–6/8/97, FIT, J. Ashe & R. Brooks, SM0096747 (SEMC, 1ex.).

###### Diagnostic description.

Length: 2.68–2.96 mm (avg. 2.84 mm); width: 2.32–2.64 mm (avg. 2.43 mm). Body large, elongate, dark-rufescent, nearly parallel-sided, smooth, with fine inconspicuous ground punctation; supraorbital stria complete, forming a carina between upper margin of eyes; frons broad, widened anteriorly, deeply depressed at middle; frontal stria complete, raised as a sinuate transverse carina; epistoma deeply depressed at middle, sides raised, subcarinate; labrum wide, emarginate; mandibles lacking basal teeth; antennal club elongate; pronotum lacking prescutellar impression, disk without secondary lateral punctures; pronotal gland openings small, annulate, located just beyond middle of disk; lateral submarginal pronotal stria complete, deeply impressed, curving inward and joining anterior marginal stria, marginal bead raised laterad; anterior marginal stria separated from lateral portions of marginal stria; elytra with single, complete epipleural stria; outer subhumeral stria complete, forming a strong lateral marginal carina, other dorsal striae reduced, those present displaced laterad, carinate, inner subhumeral present as very short apical carina, stria one complete, 2^nd^ present in apical 2/3, 3^rd^ represented only by scratch-like basal vestige; other dorsal striae absent; propygidium with depressions on either side, disk with secondary punctures mainly at sides; pygidium with broken marginal stria, disk punctate along lateral and apical margins; prosternal keel elevated, narrow, emarginate at base, with complete striae united anteriorly; prosternal lobe short, marginal stria complete; mesoventrite deeply sulcate at sides, raised along midline, marginal stria obscured; postmesocoxal stria present, recurved to mesepimeral-metepisternal corner; mesometaventral stria absent at middle, lateral metaventral stria very short, divergent, ending freely behind mesocoxa; median portions of metaventrite and 1^st^ abdominal ventrite impunctate, the latter with incomplete lateral stria along metacoxa; protibia with outer margin strongly dentate, with five marginal spines; male protarsus with modified setae; meso- and metatibiae with fine marginal spines. Male: basal piece 1/4 length of tegmen; tegmen narrow at base, weakly expanded to just beyond middle, narrowed to rounded apices; tegmen flattened at base and apex, thicker in middle in lateral view; medioventral process absent; median lobe ~ 1/3 tegmen length, basal apodemes narrower toward bases.

###### Etymology.

The ‘spectacular,’ highly autapomorphic general morphology of this species inspires its name.

###### Distribution.

This species is known from two disjunct areas, Amazonian Ecuador and Colombia in the west, and French Guiana far to the east.

###### Remarks.

This very distinctive species is easily recognized by its elongate body form, carinate outer elytral striae, and obsolete inner elytral striae (including the sutural stria). The deep pits on the mesoventrite are also unique among Neotropical Exosternini. It lacks a distinct prescutellar impression, and its relationship to others in this group is not very clear. Its annulate pronotal gland openings near the middle of the pronotal disk do seem to support relationships somewhere in this group, however.

Specimens from the rather disjunct areas reported here differ somewhat. In Guianan specimens the frons is slightly broader, the supraorbital stria is connected to the frontal stria at the sides; the lateral submarginal pronotal stria is joined to the anterior marginal, and the lateral epistomal carinae is less prominent. Even the aedeagus is a bit more parallel-sided and apically quadrate. We considered separating these as distinct, but expect that additional specimens from intervening gaps will fill these minor morphological gaps.

##### 25. Phelister pervagatus sp. nov.

Taxon classificationAnimaliaColeopteraHisteridae

10250F01-CEC4-58EF-9A68-BD446B77F488

http://zoobank.org/B15A0C51-C7F0-4092-811A-F09901F15535

**Map 10.**
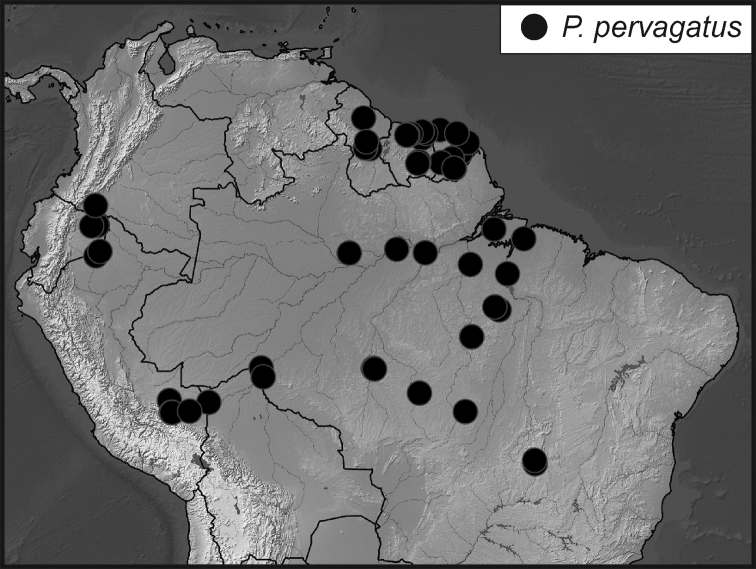
Collecting records for *Phelister pervagatus*.

Figs 15C, D , 16G, H , Map 10

###### Type material.

***Holotype* male**: “**Ecuador**: Orellana: Est. Biodiv. Tiputini, 0.6376°S, 76.1499°W, flight intercepts, 2–9.vi.2011, M.S.Caterino & A.K.Tishechkin, AT1342” / “Caterino/Tishechkin Exosternini Voucher EXO-03282” (FMNH). ***Paratypes* (53): Ecuador**: Orellana, Est. Biodiv. Tiputini (-0.6333, -76.0383), 220 m, 9/5/00–9/25/00, D. Inward & K. Jackson (NHMUK, 2ex.); Orellana, Est. Biodiv. Tiputini (-0.6376, -76.1499), 6/2/11–6/9/11, FIT, M. Caterino & A. Tishechkin (MSCC, 3ex., including DNA voucher EXO-00708); Orellana, Est. Biodiv. Tiputini (-0.64, -76.15), 7/27/08–8/3/08, FIT, A.K.Tishechkin (AKTC & LSAM, 8ex.); Orellana, Est. Biodiv. Tiputini (-0.64, -76.15), 8/3/08–8/6/08, FIT, LSAM Team (LSAM, 4ex.); Orellana, Est. Cientifica Yasuní (-0.675, -76.4), 7/11/08–7/26/08, FIT, A. Tishechkin (AKTC & LSAM, 16ex.); Orellana, Est. Cientifica Yasuní, mid. Rio Tiputini (-0.675, -76.4), 6/18/99–7/13/99, FIT, C. Carlton & A. Tishechkin (LSAM, 2ex.); **Peru**: Loreto, 1.5 km N Teniente Lopez (-2.5943, -76.1153), 210–240 m, 7/20–24/93, FIT, R. Leschen (SEMC, 4ex.); Loreto, Campamento San Jacinto (-2.3125, -75.8628), 175–215 m, 7/3/93, FIT, R. Leschen (SEMC, 14ex.).

###### Other material.

**Bolivia**: Pando, nr. Fortaleza, 2.5 km SW of Fortalea del Abuna (-9.7833, -65.5), 120 m, 2/16/96, pitfall human dung, primary floodplain forest, F. Guerra, EXO-03284 (USNM, 1ex.); Pando, nr. Villa Bella (-10.3667, -65.3667), 120 m, 2/25/96, pitfall human dung, slightly disturbed primary forest, F. Guerra (USNM, 2ex.); Pando, Santa Rosa in Manuripi (-12, -68.8667), 180–190 m, 10/24/04, human faeces baited PF trap, terra firme forest, A.C. Hamel & D. Aguirre, EXO-03283 (OUMNH, 1ex.); **Brazil**: Amazonas, INPA, Manaus (-2.4167, -59.8333), March 1994, Winkler method, Leaf litter, Terra firme fst., R. Didham, EXO-03281 (NHMUK, 1ex.); Distrito Federal, Res. Ecol. do IBGE, Brasília (-15.9416, -47.8833), October, 1986, Cerrado, FIT, I. Diniz, EXO-03271 (AKTC, 1ex.); Distrito Federal, Brasília (-15.73, -47.92), December 1987, F. Vaz de Mello (CHND, 3ex.); Mato Grosso, Mpio. Claudia (-11.4083, -55.325), 10/17/10–10/27/10, FIT, A.F. Oliveira (CEMT, 8ex.); Mato Grosso, Mpio. Cotriguaçu, Fazenda São Nicolau (-9.815, -58.2858), 12/15/10–12/18/10, FIT, F.Z. Vaz-de-Mello & A.F. Oliveira (CEMT, 8ex.); Mato Grosso, Mpio. Cotriguaçu, Fazenda São Nicolau, Mata Norte (-9.8192, -58.26), 12/8/10–12/14/10, FIT, F.Z. Vaz-de-Mello, EXO-03274 (CEMT, 1ex.); Mato Grosso, Mpio. Cotriguaçu, Fazenda São Nicolau, Matinha (-9.8383, -58.2508), 4/3/09, FIT, F.Z. Vaz-de-Mello (CEMT, 10ex.); Mato Grosso, Mpio. Cotriguaçu, Fazenda São Nicolau, Matinha (-9.8383, -58.2508), October 2009, FIT, F.Z. Vaz-de-Mello (CEMT, 5ex.); Mato Grosso, Mpio. Cotriguaçu, Fazenda São Nicolau, Matinha (-9.8383, -58.2508), October 2009, FIT, M.S. Gigliotti, EXO-03279 (CEMT, 1ex.); Mato Grosso, Mpio. Cotriguaçu, Fazenda São Nicolau, Prainha (-9.86, -58.215), September 2009, FIT, R.V. Nunes, EXO-03280 (CEMT, 1ex.); Mato Grosso, Fazenda São Nicolau (site 6) (-9.8212, -58.2592), 220 m, 12/3/13, primary forest, FIT, F. Génier (CMNC, 1ex.); Mato Grosso, Mpio. Querencia, Fazenda São Luiz (-12.597, -52.3749), February 2009, FIT, R. Andrade (CEMT, 2ex.); Pará, Altamira – Marabá: km 18 (-3.15, -52.05), May 1984, FIT (CHND, 6ex.); Pará, Barcarena (-1.5, -48.6167), 6/13/91–6/25/91, FIT (CHND, 4ex.); Pará, Carajás (Serra Norte) (-6.0667, -50.2), 9/26/86–10/6/86, FIT (CHND, 5ex.); Pará, Ilha Arapiuns (-2.4, -54.95), 12/30/08–12/31/08, FIT, EXO-03270 (CHND, 1ex.); Pará, Marajó-Breves (-0.8833, -50.5333), 11/18/87–12/5/87, FIT (CHND, 3ex.); Pará, Redenção (-7.7927, -51.9635), 215 m, October, 1999, FIT (CHND, 3ex.); Pará, Serra dos Carajás, Rio Itacaiunas (-5.8733, -50.48), 1/26/84, S. excr. humain (CHND, 2ex.); Pará, Tucuruí (-3.75, -49.667), 7/10/85–7/29/85, FIT (CHND, 3ex.); Pará, Tucuruí (-3.75, -49.667), 6/19/86–7/7/86, FIT (CHND, 2ex.); Pará, Tucuruí (-3.75, -49.667), 6/23/86–7/7/86, FIT, EXO-03266 (CHND, 1ex.); Pará, Tucuruí (-3.75, -49.667), February 1986, FIT (CHND, 6ex.); Pará, Tucuruí (-3.75, -49.667), April 1986, FIT, EXO-03264 (CHND, 1ex.); **French Guiana**: Belvédère de Saül, point de vue (3.6228, -53.2094), 12/20/10, FIT, SEAG (CHND, 2ex.); Régina, Rés. Natur. des Nouragues (4.0378, -52.6725), 11/3/09, FIT, SEAG, EXO-03216 (CHND, 1ex.); Régina, Rés. Natur. des Nouragues (4.0378, -52.6725), 1/28/10, FIT, SEAG (CHND, 7ex.); Régina, Rés. Natur. des Nouragues, Camp Inselberg (4.0833, -52.6833), 7/20/10 and 9/22/10, FIT, SEAG (CHND, 4ex.); Roura (8.4 km SSE) (4.6781, -52.2236), 200 m, 5/29/97–6/10/97, FIT, J. Ashe & R. Brooks, EXO-03211 (CMNC, 1ex.); Saül, 7 km N, 3 km NW Les Eaux Claires, Mt. La Fumée (3.6628, -53.2219), 490 m, 6/1/97–6/8/97, FIT, J. Ashe & R. Brooks, SM0096756 (SEMC, 1ex.); Saül, 7 km N, Les Eaux Claires (3.6628, -53.2219), 220 m, 5/30/97–6/4/97, FIT, J. Ashe & R. Brooks (SEMC, 2ex.); Rés. Forêt des Malgaches, St. Laurent du Maroni (5.5, -54), 10/4/77, Piège + cad. souris, N. Degallier, EXO-03218 (CHND, 1ex.); Maripasoula: Kayodé (3.389, -53.924), December 2005, J. Dalmon (CHND, 3ex.); Maripasoula: Kayodé (3.389, -53.924), October, 2005, J. Dalmon (CHND, 4ex.); Mont tabulaire, Itoupé (3.022, -53.0842), 800 m, 3/17/10, FIT, SEAG (CHND, 38ex.); Mont tabulaire, Itoupé (3.023, -53.0955), 570 m, 3/24/10, FIT, SEAG (CHND, 3ex.); Mont Tabulaire Itoupé (3.0303, -53.1067), 400 m, 3/17/10, FIT, SEAG (CHND, 11ex.); Mont Tabulaire Itoupé (3.0303, -53.1067), 400 m, 3/23/10, FIT, SEAG (CHND, 4ex.); Paracou (5.27, -52.92), November 1996, FIT, P.M. Hammond (NHMUK, 6ex.); **Guyana**: Mazaruni Potaro, Takutu Mountains (6.25, -58.9167), 12/8/83, FIT, montane rainforest near logging area, P.D. Perkins & W.E. Steiner (USNM, 2ex.); Region 8, Iwokrama Field Stn., Iwokrama Forest, 1 km W Kurupukari (4.6719, -58.6844), 60 m, 5/21/01, *Acromyrmex hystrix* refuse pile, R. Brooks, Z. Falin (SEMC, 4ex.); Region 8, Iwokrama Field Stn., Iwokrama Forest, 1 km W Kurupukari (4.6719, -58.6844), 60 m, 5/26/01–5/29/01, FIT, R. Brooks, Z. Falin, SM0565329 (SEMC, 1ex.); Region 8, Kabocalli Field Stn., Iwokrama Forest (4.2844, -58.5097), 60 m, 6/3/01–6/5/01, FIT, R. Brooks, Z. Falin (SEMC, 4ex.); Region 8, Iwokrama Forest, 26 km SW Kurupukari, Iwokrama Mt. (4.3339, -58.7883), 400 m, 5/23/01–5/25/01, FIT, R. Brooks, Z. Falin, SM0565198 (SEMC, 1ex.); Region 8, Iwokrama Forest, 26 km SW Kurupukari, Iwokrama Mt. (4.3339, -58.7883), 600 m, 5/23/01–5/25/01, FIT, R. Brooks, Z. Falin (SEMC, 3ex.); Region 8, Iwokrama Forest, 26 km SW Kurupukari, Iwokrama Mt. (4.3339, -58.7883), 300 m, 5/23/01–5/25/01, FIT, R. Brooks, Z. Falin (SEMC, 4ex.); Region 8, Iwokrama Forest, 26 km SW Kurupukari, Iwokrama Mt. Base camp (4.3381, -58.8106), 100 m, 5/22/01–5/25/01, FIT, R. Brooks, Z. Falin, SM0572702 (SEMC, 1ex.); Region 8, Iwokrama Forest, Turtle Mt. summit (4.7325, -58.7336), 290 m, 5/30/01–6/1/01, FIT, R. Brooks, Z. Falin (SEMC, 2ex.); **Peru**: Madre de Dios, Cocha Cashu Bio. Stn., Manu Nat. Park (-11.8833, -71.4), 360 msm, 3/19/04, En Heces, Larsen & Asenjo, EXO-03256 (MNHN Peru-Lima, 1ex.); Madre de Dios, Pantiacolla Lodge, 2–7 km NW El Mirador Trail, Alto Madre de Dios River (-12.6528, -71.2578), 450–700 m, 10/23/00–10/26/00, FIT, R. Brooks (SEMC, 4ex.); Madre de Dios, Pantiacolla Lodge, 5.5 km NW El Mirador Trail, Alto Madre de Dios River (-12.6528, -71.2578), 500 m, 10/23/00–10/26/00, FIT, R. Brooks (SEMC, 2ex.); Madre de Dios, Pantiacolla Lodge, 8 km NW EL Mirador Trail, Alto Madre de Dios River (-12.6417, -71.2781), 800 m, 10/23/00–10/26/00, FIT, R. Brooks (SEMC, 11ex.); Madre de Dios, Pantiacolla Lodge, Alto Madre de Dios R. (-12.655, -71.2317), 420 m, 11/14/07–11/19/07, FIT, D. Brzoska (SEMC, 57ex.); Madre de Dios, Pantiacolla Lodge, Alto Madre de Dios River (-12.6561, -71.2319), 400 m, 10/23/00–10/26/00, FIT, R. Brooks (SEMC, 10ex.); Madre de Dios, Rio Los Amigos, CICRA (-12.5709, -70.1018), 25/150 m, 11/18/06–11/21/06, FIT, A. Asenjo, EXO-03255 (MUSM Lima, 1ex.); **Suriname**: Brokopondo, Brownsberg Nature Preserve, Witi Creek Trail (4.9486, -55.1814), 340 m, 6/23/99–6/25/99, FIT, Z. Falin, A. Gangadin, H. Hiwat, SM0166993 (SEMC, 1ex.); Brokopondo, Brownsberg Nature Preserve, Witi Creek Trail (4.9486, -55.1814), 80 m, 6/23/99–6/25/99, FIT, Z. Falin, A. Gangadin, H. Hiwat, SM0178761 (SEMC, 1ex.); Commewijne, Akintosoela (5.2714, -54.9208), 40 m, 7/3/99, FIT, Z. Falin, EXO-03206 (CMNC, 1ex.); Commewijne, Akintosoela, 32 km SE Suriname River bridge, road to Redi Doti (5.2714, -54.9208), 40 m, 6/29/99–7/3/99, FIT, Z. Falin, B. DeDijn, A. Gangadin (SEMC, 2ex.); Commewijne, Akintosoela, CELOS camp, rd. to Redi Doli (5.2714, -54.9208), 50 m, 7/3/99, Z. Falin (CMNC, 2ex.); Marowijne, 15 km NE Palumeu ca (3.45, -55.3667), 160 m, 7/7/99, FIT, Z. Falin (CMNC, 2ex.); Marowijne, Palumeu (3.3489, -55.4383), 160 m, 7/8–9/99, FIT, Z. Falin (CMNC, 5ex.); Marowijne, Palumeu, 15 km NE, on Tapanahony River, trail to Poti Hill (3.45, -55.3667), 160 m, 7/6/99–7/7/99, FIT, Z. Falin, D. Konoe (SEMC, 3ex.); Marowijne, Palumeu, 15 km NE, on Tapanahony River, trail to Poti Hill (3.45, -55.3667), 160 m, 7/7/99, monkey dung, Z. Falin (SEMC, 9ex.); Pará, 11 km SE Zanderij Airport (5.3933, -55.1581), 30 m, 6/20/99, FIT, Z. Falin (CMNC, 3ex.); Saramacca, W. SurinameRd., 108 km WSW Zanderij Airport (5.2269, -55.8817), 30 m, 6/10/99, FIT, Z. Falin, EXO-03201 (CMNC, 1ex.); Saramacca, West Suriname Road, 108 km WSW Zanderij Airport (5.2269, -55.8817), 30 m, 6/8/99–6/10/99, FIT, Z. Falin, B. DeDijn, SM0167116 (SEMC, 1ex.); Saramacca, West Suriname Road, 108 km WSW Zanderij Airport (5.2269, -55.8817), 30 m, 6/8/99–6/10/99, FIT, Z. Falin, B. DeDijn (SEMC, 3ex.); Saramacca, West Suriname Road, 139 km WSW Zanderij Airport (5.15, -56.0667), 40–50 m, 6/10/99–6/14/99, FIT, Z. Falin, B. DeDijn, SM0167011 (SEMC, 1ex.); Saramacca, West Suriname Road, 145 km WSW Zanderij Airport (5.1517, -56.1456), 50 m, 6/10/99–6/14/99, FIT, Z. Falin, B. DeDijn (SEMC, 2ex.); Sipaliwini, CI-RAP Surv. Camp 1: on Kutari River (-2.1753, -56.7874), 228 m, 8/19/10–8/24/10, FIT, Larsen & Short, EXO-03207 (SEMC, 24ex.); Surv. Camp 3: Wehepai SE Kwamala (2.3629, -56.6977), 237 m, 9/3/10–9/7/10, FIT, T. Larsen & A.E.Z. Short (SEMC, 1ex.); Sipaliwini, CI-RAP Surv. Camp 1: upper Palumeu River (2.4770°N, 55.6294°W), 275 m, FIT, 3/10/12–3/16/12, A.E.Z. Short (AKTC & SEMC, 7ex.); Sipaliwini, CI-RAP Surv. Camp 4: on lower Kasikasima River (-2.9773, -55.3850), 200 m, 3/20/12–3/25/12, FIT and dung traps, T. Larsen (AKTC & SEMC, 41ex.).

###### Diagnostic description.

Length: 1.38–1.77 mm (avg. 1.60 mm); width: 1.30–1.62 mm (avg. 1.49 mm). Body rounded, convex, dark rufescent, with moderately conspicuous ground punctation; frons narrow, depressed along midline; supraorbital stria complete, detached at sides from frontal; frontal stria interrupted across depression; epistoma depressed at middle, sides weakly elevated; labrum weakly emarginate; each mandible with subacute basal tooth; sides of pronotum rather abruptly narrowed in anterior third; prescutellar impression subtriangular, rounded at base, ~ 1.5 × width of scutellum; pronotal disk with secondary punctures in lateral thirds; median pronotal gland openings small, annulate, rather widely separated, ~ 2/3 behind anterior margin; marginal pronotal stria complete around front and sides; submarginal striae absent; elytron with single, complete epipleural stria; outer subhumeral stria very short, apical, inner absent; dorsal stria one slightly abbreviated from apex, striae two and three usually complete, becoming fragmented apically, striae four and five represented by series of apical punctures, 4^th^ also represented by strong, transverse basal arch; sutural stria present in apical 2/3, also becoming series of punctures toward apex; propygidium uniformly punctate, secondary punctures separated by ~ 1.5 × their diameters; pygidium much more finely and sparsely punctate; prosternal keel very broad, shallowly emarginate at base, lacking striae; prosternal lobe very short, also lacking marginal stria; mesoventrite weakly rounded in front, with fine marginal stria continued by very short, fine postmesocoxal stria; mesometaventral stria strongly crenulate, arched forward to middle of mesoventrite, continued by very short lateral metaventral stria; metaventrite and 1^st^ abdominal ventrite impunctate, but 1^st^ abdominal ventrite with distinct, annulate gland opening mesad metacoxa; protibia slender, outer margin rounded, weakly dentate, with ~ four marginal spines; male with flattened ventral protarsal setae; meso- and metatibiae very slender, with few fine marginal spines toward apex. Male: basal piece 1/3 length of tegmen; tegmen narrow at base, weakly expanded to near apex, apices rounded; tegmen slightly curved in lateral view; medioventral process absent; median lobe ~ 3/4 tegmen length, basal apodemes long, thin.

###### Etymology.

The name *pervagatus* approximately translates to widespread, common, which is very true of this species.

###### Distribution.

This species occurs across tropical South America, found in the Guianas, Ecuador, Peru, Bolivia, and across much of central and northern Brazil.

###### Remarks.

This species is quite consistent in its morphology throughout its broad range. It can be easily recognized by its round (not very elongate) body form, broad prosternal keel lacking striae, short prosternal lobe, laterally flattened pronotal margins, lack of lateral submarginal pronotal stria, and apically serially punctate elytral striae. We restrict the type series to a small number of localities around eastern Ecuador (from which we have obtained DNA sequences) and northern Peru.

While the vast majority of specimens of *P. pervagatus* have been collected by flight interception traps, a few specimens have been collected in leafcutter ant (*Acromyrmex hystrix*) refuse piles, as well as in dung-baited pitfalls.

#### *P. amazoniae* subgroup

This small group of eleven species is well characterized by distinctive genitalic characters. We have only found two external characters that typically co-occur with these, and *P. geijskesi* is an exception to both of them.

Genitalic characters:

- Aedeagus strongly narrowed and bent downward (‘hooked’) apically
- Medioventral process of tegmen absent
- T10 fused and discretely sclerotized across apical 1/2

External characters

- Epistoma with slight pinch in front of antennal bases
- Tibiae often broadened, extremely so in some cases

##### 26. Phelister morbidus sp. nov.

Taxon classificationAnimaliaColeopteraHisteridae

FE3F5B2D-809F-5C8C-8015-E0C2FADF70EC

http://zoobank.org/23D70694-1379-4B68-9018-1FFD33C5AAA2

**Figure 17.**
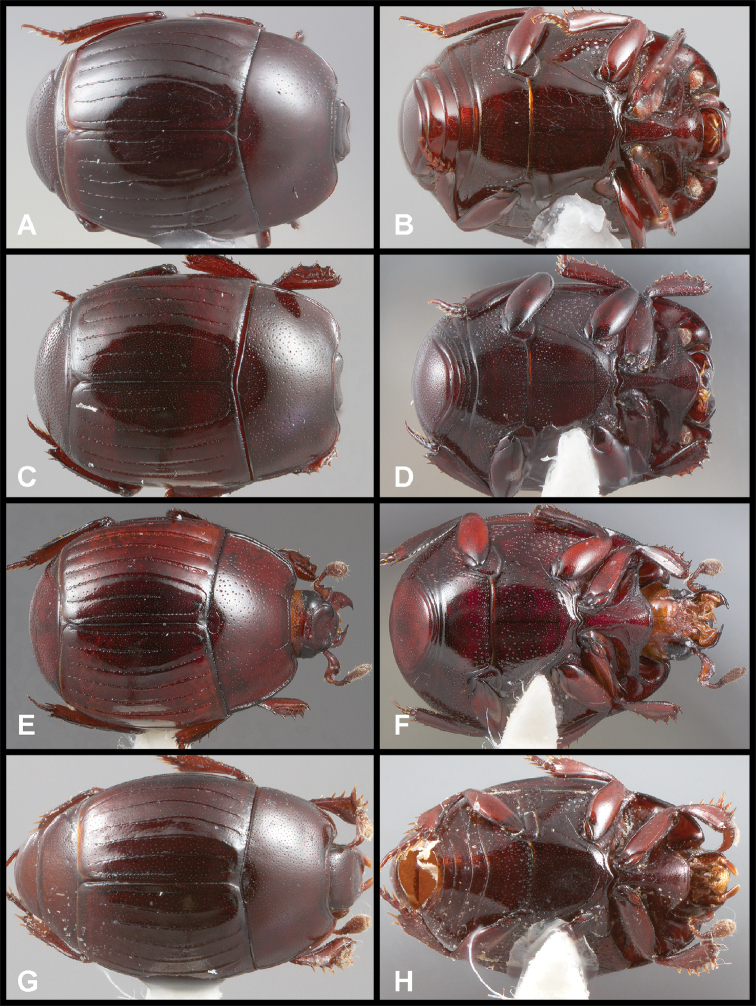
**A, B***Phelister morbidus*: **A** dorsal habitus **B** ventral habitus **C, D***P. annulatus*: **C** dorsal habitus **D** ventral habitus **E, F***P. sphaericus*: **E** dorsal habitus **F** ventral habitus **G, H***P. geijskesi*: **G** dorsal habitus **H** ventral habitus.

**Map 11.**
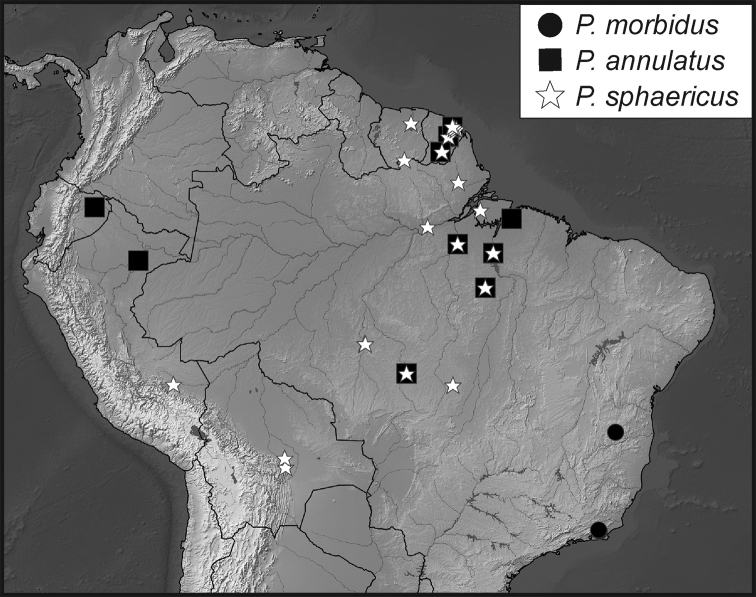
Collecting records for *Phelister morbidus* (circles), *P. annulatus* (squares), and *P. sphaericus* (stars).

**Figure 18.**
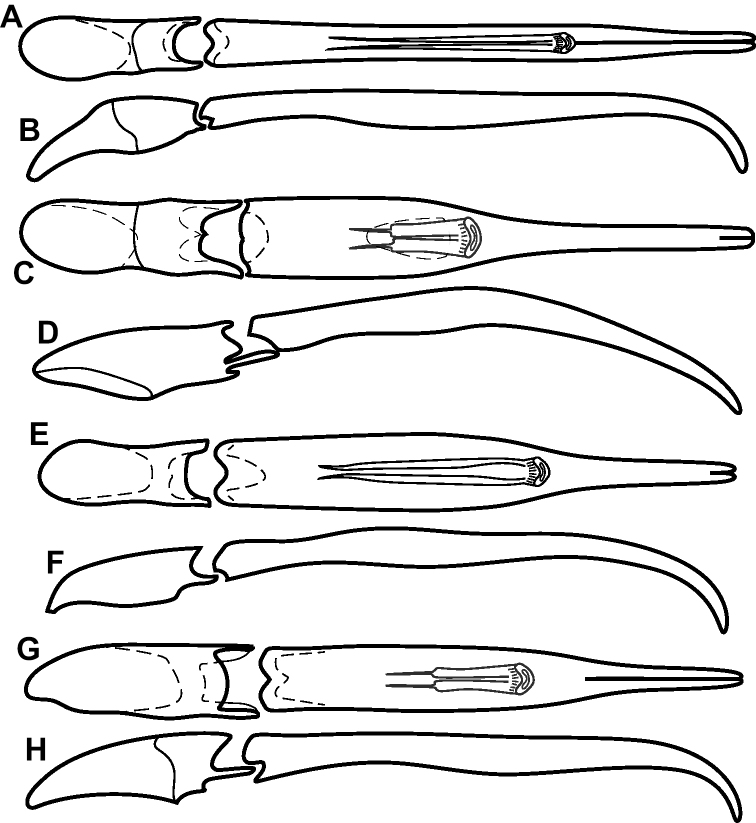
Male genitalia **A, B***Phelister morbidus*: **A** aedeagus, dorsal **B** aedeagus, lateral **C, D***P. annulatus*: **C** aedeagus, dorsal **D** aedeagus, lateral **E, F***P. sphaericus*: **E** aedeagus, dorsal **F** aedeagus, lateral **G, H***P. geijskesi*: **G** aedeagus, dorsal **H** aedeagus, lateral.

Figs 17A, B , 18A, B , Map 11

###### Type material.

***Holotype* male**: “**Brazil**: Minas Gerais, Águas Vermelhas 15°45'S, 41°28'W [-15.75, -41.4667]. Cad. de serpent. Dec 1983” / “Caterino/Tishechkin Exosternini Voucher EXO-00381” (MNHN).

###### Other material.

**Brazil**: Rio de Janeiro, Nova Friburgo, 17 km S (-22.3845, -42.5583), 750 m, 1/23/00, carrion trap, secondary montane Atlantic forest, F. Génier & S. Ide, SM0809245 (SEMC, 1ex.); Rio de Janeiro, Nova Friburgo, Sans Souci (-22.3, -42.6), 11/9/09–11/15/09, FIT, E. Grossi, DZUP 272506 (DZUP, 1ex.); Rio de Janeiro, Macaé de Cima, Nova Friburgo (-22.3816, -42.4819), 1030 m, 10/1/03–10/31/07, FIT (AKTC & CHND, 3ex.).

###### Diagnostic description.

Length: 1.77–2.17 mm (avg. 2.02 mm); width: 1.58–1.81 mm (avg. 1.72 mm). Body elongate oval, convex, very faintly bicolored, rufescent with elytra slightly darker than pronotum or pygidia, ground punctation inconspicuous, very fine and sparse; frons depressed along midline; supraorbital stria present, detached from sides of frontal; frontal stria complete or with central portion detached from sides (type); epistoma with sides angularly subcarinate; labrum shallowly emarginate at apex; mandibles lacking basal teeth; pronotum with semicircular prescutellar impression, slightly broader than scutellum; pronotal disk with numerous secondary punctures in lateral thirds; marginal pronotal stria complete along sides and front; submarginal striae absent; elytron with single complete epipleural stria; outer subhumeral stria present in apical 1/2, inner absent; dorsal striae 1–4 complete, 4^th^ arched to suture, 5^th^ present in apical 1/2, sutural stria present in apical 2/3; all dorsal striae finely impressed; propygidium with secondary punctures rather dense in basal 2/3, sparser apically; pygidium with secondary punctures only in basal 1/3; prosternal keel emarginate at base, rather broad, flat, with striae united along base and weakly convergent, meeting anteriorly; prosternal lobe short, lacking marginal stria; mesoventrite produced at middle, with complete marginal stria continued at side by postmesocoxal stria, which is short, ending freely behind outer edge of coxa; mesometaventral stria angulate at middle, reaching anterior third of mesoventrite, continued by lateral metaventral stria to middle of metacoxa; 1^st^ abdominal ventrite impunctate, with single, incomplete lateral stria; protibia with outer margin weakly dentate, with ~ six marginal spines; outer margins of meso- and metatibiae with few fine marginal spines; all tarsi with ventral setae at least moderately flattened. Male: aedeagus very elongate, narrow; basal piece 1/4 length of tegmen; tegmen narrowed from base to apex, apices strongly hooked; medioventral process absent; median lobe ~ 1/2 tegmen length, thin.

###### Etymology.

With two specimen records coming from carrion traps, this species is named for its ‘morbid’ apparent interest in dead carcasses.

###### Distribution.

This species is only known from a couple localities in east-central Brazil (Rio de Janeiro and Minas Gerais).

###### Remarks.

This species can best be recognized by the combination of its detached central portion of the frontal stria, the relatively broad prosternal keel, and the lack of a stria along the margin of the prosternal lobe. Due to the broad distributional gap between the known localities, we restrict the type series to the single holotype specimen from Minas Gerais (from which we illustrated the male genitalia).

##### 27. Phelister annulatus sp. nov.

Taxon classificationAnimaliaColeopteraHisteridae

11F24859-095E-53EF-AEE2-D1E062EBA5A1

http://zoobank.org/275E370E-A018-47DA-8051-D3DD6A6A051F

Figs 17C, D , 18C, D , Map 11

###### Type material.

***Holotype* male**: “**Brazil**: Pará, Belém, Utinga (IPEAN) 1°27'S, 48°26'W [-1.45, -48.4333] Piège d’interception. x.1986” / “Caterino/Tishechkin Exosternini Voucher EXO-03176 (CEMT). ***Paratypes* (29): Brazil**: Amapá, Serra do Navio (0.98, -52.00), 5/1/91–5/14/91, FIT, EXO-03177 (CHND, 1ex.); Mato Grosso, Sinop (-11.8167, -55.4833), 6/12/85–6/24/85, FIT, EXO-03175 (CHND, 1ex.); Pará, Altamira – Marabá, km 18 (-3.15, -52.05), 11/22/83, Ss. cad. de rat. (CHND, 2ex.); Pará, Carajás (Serra Norte) (-6.0667, -50.2), May 1985, FIT (CHND, 3ex.); Pará, Tucuruí (-3.75, -49.667), 6/19/86–7/7/86, FIT, EXO-03179 (CHND, 1ex.); Pará, Tucuruí (-3.75, -49.667), June 1985, FIT (CHND, 2ex.); **Ecuador**: Orellana, Est. Cientifica Yasuní, mid. Rio Tiputini (-0.675, -76.4), 6/24/99–6/26/99, Dead fish trap, late stage, C. Carlton, LSAM 0045449 (LSAM, 1ex.); **French Guiana**: Régina, Rés. Natur. des Nouragues, Camp Inselberg (4.0833, -52.6833), 11/9/10, FIT, SEAG, EXO-03184 (CHND, 1ex.); Régina, Rés. Natur. des Nouragues (4.0378, -52.6725), 11/3/09, FIT, SEAG, EXO-03183 (CHND, 1ex.); Régina, Rés. Natur. des Nouragues, Camp Inselberg (4.0833, -52.6833), 1/25/11, FIT, SEAG (CHND, 1ex.); Belvédère de Saül, point de vue (3.6228, -53.2094), 11/31/2010, FIT, SEAG (CHND, 2ex.); Montagne des Chevaux (4.7167, -52.4), 9/21/08, FIT, SEAG, EXO-00369 (CHND, 1ex.); Montagne des Chevaux (4.7167, -52.4), 12/16/08–01/18/09, FIT, SEAG (AKTC & CHND, 8ex.); Montagne des Chevaux (4.7167, -52.4), 5/16/09, FIT, SEAG, EXO-03181 (CHND, 1ex.); Montagne des Chevaux (4.7167, -52.4), 7/11/09, FIT, SEAG (CHND, 2ex.); **Peru**: Loreto, Iquitos – Nauta rd., km 58, Rio Itaya (-4.2563, -73.4675), 120 m, 5/5/09–5/9/09, FIT, next to entrance into *Eciton burchelli* statary bivouac in a hollow tree, A.V. Petrov, EXO-00368 (AKTC, 1ex.).

###### Diagnostic description.

Length: 2.40–2.56 mm (avg. 2.44 mm); width: 1.89–2.25 mm (avg. 2.08 mm). Body elongate, sides almost subparallel, dark rufescent to piceous, with conspicuous ground punctation; frons broadly impressed along midline, with sparse small punctures intermingled with ground punctation; supraorbital stria present, detached at sides; frontal stria complete, fine; epistoma raised along lateral and anterior margins; labrum emarginate, apical margin subcarinate; mandibles lacking basal teeth; prescutellar impression semicircular, slightly wider than scutellum; pronotal disk with doubly punctate, with numerous secondary punctures intermingled with ground punctation, denser toward sides; median pronotal gland openings distinctly annulate, just > 1/2 behind anterior margin; marginal pronotal stria complete along sides and front; submarginal pronotal stria complete, deeply impressed, close to margin, barely curving inward at front; elytron with single complete epipleural stria; outer subhumeral stria present at base and apex, briefly interrupted at middle in most individuals; inner subhumeral stria fragmentarily present at middle, rarely nearly complete; dorsal striae 1–4 complete, the 4^th^ arched to the sutural, 5^th^ obsolete in basal fourth, sutural stria complete; all elytral striae weakly crenulate; pygidia densely doubly punctate, with fine and secondary punctures uniformly intermixed; prosternum emarginate at base, striae divergent at base, sinuate to, and meeting at, apex; prosternal lobe short, with complete marginal striae; mesoventrite produced, with complete marginal stria continued at sides by postmesocoxal stria, which ends behind outer corner of mesocoxa; mesometaventral stria angulate at middle, reaching anterior third of mesoventrite, continued at sides by lateral metaventral stria to middle of metacoxa; metaventrite with ground punctation denser toward midline (particularly in males), with secondary punctures interspersed near metacoxae; 1^st^ abdominal ventrite with secondary punctures along anterior margin, lateral stria incomplete along inner margin of metacoxa; protibia weakly expanded, outer margin with ~ five spinose denticles; meso- and metatibiae also weakly expanded, with rather conspicuous marginal spines; ventral setae of all tarsi flattened (those of protarsus, particularly). Male: basal piece 1/3 length of tegmen; tegmen with sides subparallel in basal 1/3, abruptly narrowed to long, downturned apical portion; medioventral process absent; median lobe short, thick.

###### Etymology.

This species name refers to the annulate pronotal punctures (though this character is common to many species in the group).

###### Distribution.

Most specimens of this species have been collected in northeastern Brazil and French Guiana. However, it is also known from Amazonian Ecuador and Peru, as well as Mato Grosso, Brazil.

###### Remarks.

This species can be distinguished by the following combination of characters, pronotal disk largely doubly punctate (secondary punctures are intermingled throughout, though slightly denser toward the sides), which contrasts with the elytra, which exhibit ground punctation only, frontal stria complete, lateral submarginal pronotal stria close to margin, mesometaventral stria very strongly angulate forward, and the outer subhumeral stria interrupted at the middle, but otherwise complete.

##### 28. Phelister sphaericus sp. nov.

Taxon classificationAnimaliaColeopteraHisteridae

641C82E3-73BD-5BE4-BE9C-2B5EF5FA8857

http://zoobank.org/FB5D3517-934C-499A-A163-4CF100A72F16

Figs 17E, F , 18E, F , Map 11

###### Type material.

***Holotype* male**: “**Bolivia**: Santa Cruz, 4–5 km SSE Buena Vista, Hotel Flora y Fauna 17°29'S, 63°33'W [-17.4987, -63.6521] F.I.T.#4 29.iv.-6.v.2004, A.R. Cline” / “Caterino/Tishechkin Exosternini Voucher EXO-00465” (FMNH). ***Paratypes* (53): Bolivia**: Santa Cruz, Amboro National Park, Los Volcanes, c. (-18.1, -63.6), 1000 m, 11/20/04–12/12/04, Carrion trap with rotting small fish, Barclay, M.V.L. & Mendel, H., EXO-02401 (NHMUK, 1ex.); Santa Cruz, 4–5 km SSE Buena Vista, Hotel Flora y Fauna (-17.4987, -63.6521), 440 m, 12/24/03–12/31/03, FIT, S. & J. Peck (AKTC, 2ex.); Santa Cruz, 4–5 km SSE Buena Vista, Hotel Flora y Fauna (-17.4987, -63.6521), 4/25/04–5/6/04, FIT, A.R. Cline (AKTC, 3ex.); Santa Cruz, 5 km SSE Buena Vista, Flora y Fauna Hotel (-17.4987, -63.6521), 12/15/03–12/24/03, FIT, S. & J. Peck (AKTC, FMNH, MSCC, & CMNC, 40ex.); **Brazil**: Mato Grosso, Mpio. Cotriguaçu, Fazenda São Nicolau (-9.815, -58.2858), 12/15/10–12/18/10, FIT, F.Z. Vaz-de-Mello & A.F. Oliveira, EXO-02413 (CEMT, 1ex.); Mato Grosso, Mpio. Cotriguaçu, Fazenda São Nicolau, Mata Norte (-9.8192, -58.26), 12/8/10–12/14/10, FIT, F.Z. Vaz-de-Mello (CEMT, 2ex.); Mato Grosso, Mpio. Cotriguaçu, Fazenda São Nicolau, Matinha (-9.8383, -58.2508), October 2009, FIT, F.Z. Vaz-de-Mello (CEMT, 4ex.); Mato Grosso, Mpio. Cotriguaçu, Fazenda São Nicolau, Matinha (-9.8383, -58.2508), October 2009, FIT, M.S. Gigliotti (CEMT, 21ex.); Mato Grosso, Mpio. Querencia, Fazenda São Luiz (-12.597, -52.3749), February 2009, FIT, R. Andrade (CEMT, 3ex.); Mato Grosso, Sinop (-11.8167, -55.4833), 6/12/85–6/24/85, FIT (CHND, 3ex.);

###### Other material.

**Brazil**: Amapá, Serra do Navio (0.9833, -52), 1/28/90–2/2/90, FIT (CHND, 8ex.); Pará, Altamira – Marabá, km 18 (-3.15, -52.05), May 1985, FIT, EXO-02402 (CHND, 1ex.); Pará, Carajás (Serra Norte) (-6.0667, -50.2), May 1985, FIT, EXO-02403 (CHND, 1ex.); Pará, Marajó-Breves (-0.8833, -50.5333), 11/18/87–12/5/87, FIT, EXO-02404 (CHND, 1ex.); Pará, Monte Alegre (-2, -54.07), 6/17/92–7/3/92, FIT, EXO-02408 (CHND, 1ex.); Pará, Tucuruí (-3.75, -49.667), 12/9/85–12/17/85, FIT (CHND, 5ex.); Pará, Tucuruí (-3.75, -49.667), 5/20/87–6/15/87, FIT (CHND, 2ex.); **French Guiana**: Belvédère de Saül, point de vue (3.6228, -53.2094), 9/2/10, 11/30/10, 12/20/10, 1/17/11, 1/31/11, 2/7/11 and 2/14/11, FIT, SEAG (AKTC & CHND, 14ex.); Régina, Rés. Natur. des Nouragues (4.0378, -52.6725), 11/3/09, FIT, SEAG, EXO-03189 (CHND, 1ex.); Roura, 39.4 km SSE (4.5453, -52.1406), 270 m, 5/29/97–6/10/97, FIT, J. Ashe & R. Brooks, SM0101424 (SEMC, 1ex.); Régina, Rés. Natur. des Nouragues, Camp Inselberg (4.0833, -52.6833), 7/20/09, 9/9/10, 9/30/09, 10/8/09 and 1/25/11, FIT, SEAG (AKTC & CHND, 10ex.); Rés. Trésor (route de Kaw Pk18) (4.6105, -52.279), 225 m, 11/6/09, FIT, SEAG (CHND, 2ex.); Rés. Trésor (route de Kaw Pk18) (4.6105, -52.279), 225 m, 11/12/09, FIT, SEAG, EXO-03191 (CHND, 1ex.); Mont Tabulaire Itoupé (3.0303, -53.1067), 400 m, 3/31/10, FIT, SEAG (CHND, 2ex.); Montagne des Chevaux (4.7167, -52.4), 10/11/09, FIT, SEAG (CHND, 2ex.); Montagne des Chevaux (4.7167, -52.4), 4/14/10, FIT, SEAG (CHND, 4ex.); Montagne des Chevaux (4.7167, -52.4), 5/22/10, FIT, SEAG (CHND, 2ex.); Montagne des Chevaux (4.7167, -52.4), 12/6/09–12/26/08, 1/4/09, 1/18/09 and 2/23/09, FIT, SEAG (AKTC & CHND, 8ex.); Route de Bélizon, p. km 4.5, flight intercept, 8/1/16–8/31/16, J.-L. Guiglaris (AVSC, 1ex.). **Suriname**: Brokopondo, Brownsberg Nature Preserve, Witi Creek Trail (4.9486, -55.1814), 260 m, 6/23/99–6/25/99, FIT, Z. Falin, A. Gangadin, H. Hiwat, SM0165801 (SEMC, 1ex.); Brokopondo, Brownsberg Nature Preserve, Witi Creek Trail (4.9486, -55.1814), 340 m, 6/23/99–6/25/99, FIT, Z. Falin, A. Gangadin, H. Hiwat (SEMC, 4ex.); Sipaliwini, CI-RAP Survey camp 1, upper Palumeu (2.477, -55.6294), 225 m, 3/10/12–3/16/12, FIT, A.E.Z. Short, EXO-03194 (SEMC, 1ex.); **Peru**: Madre de Dios, CICRA Field Stn., ~ 2 km NW cafeteria res. plot. 12.55201°S, 70.10991°W, 295 m, Flight intrcept. 7–9.vi.2011, Chaboo Team (SEMC 1ex.).

###### Diagnostic description.

Length: 1.81–2.88 mm (avg. 2.40 mm); width: 1.89–2.76 mm (avg. 2.25 mm). Body broadly rounded, slightly flattened, rufescent, with elytra faintly bicolored, sides lighter than middle, ground punctation conspicuous throughout; frons rather narrow, deeply depressed along midline; supraorbital stria present, detached at sides; frontal stria complete; epistoma raised along sides and front; labrum shallowly emarginate and subcarinate at apex; right mandible with small basal tooth; pronotum with broadly oval prescutellar impression, ~ 3 × width of scutellum; pronotal disk doubly punctate, with secondary punctures interspersed throughout, denser to sides; median pronotal gland openings distinctly annulate, ~ 2/3 behind anterior margin; lateral marginal pronotal stria complete, interrupted behind eyes, with ends of median portion recurved posterad briefly; lateral submarginal stria complete, deeply impressed, sides of pronotum weakly explanate; elytron with single complete epipleural stria; outer subhumeral stria complete, inner absent; dorsal striae 1–5 and sutural complete, 5^th^ obsolete in basal 1/3; all striae weakly crenulate; pygidia more or less uniformly doubly punctate; prosternum emarginate at base, striae divergent at base, convergent to apex; prosternal lobe short, with complete marginal striae; mesoventrite produced, with complete marginal stria continued at sides by postmesocoxal stria, which ends behind outer corner of mesocoxa; mesometaventral stria angulate at middle, reaching anterior third of mesoventrite, continued at sides by lateral metaventral stria to inner 1/3 of metacoxa; metaventrite with secondary punctures denser towards metacoxae; 1^st^ abdominal ventrite with secondary punctures along anterior margin, lateral stria incomplete along inner margin of metacoxa; protibia weakly expanded, outer margin with ~ five spinose denticles; meso- and metatibiae slender, with fine marginal spines; ventral setae of all tarsi flattened (those of protarsus, particularly). Male: basal piece 1/4 length of tegmen; tegmen with sides subparallel in basal 1/2, abruptly narrowed to ventrally hooked apices; medioventral process absent; median lobe ~ 1/2 tegmen length.

###### Etymology.

This species name, *sphaericus*, refers to its very convex body form.

###### Distribution.

This species has an unusual distribution, with the highest concentration of records coming from northeastern Brazil, Suriname, and French Guiana, but with a number also from central Brazil and Bolivia to the southwest.

###### Remarks.

This species is quite broadly rounded, which alone is distinctive. It is also characterized by its slightly explanate pronotum bearing an elevated marginal bead, doubly punctate pronotal disk, complete outer subhumeral stria, and complete 4^th^ and sutural striae united by a basal arch.

##### 29. Phelister geijskesi

Taxon classificationAnimaliaColeopteraHisteridae

Kanaar, 1997

B685AEA2-8731-5BA5-ADFE-BEDCC0923026

**Map 12.**
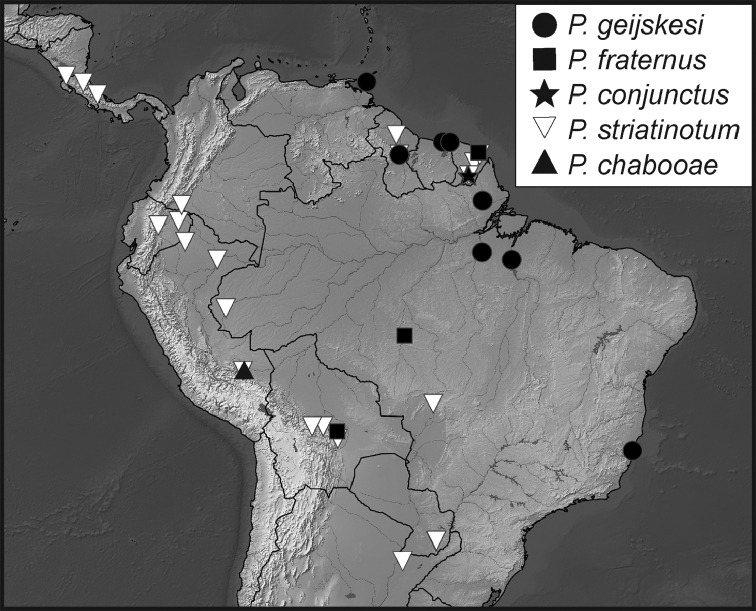
Collecting records for *Phelister geijskesi* (circles), *P. fraternus* (squares), *P. conjunctus* (star), *P. striatinotum* (inverted triangles), and *P. chabooae* (upright triangle).

Figs 17G, H , 18G, H , Map 12

Phelister geijskesi Kanaar, 1997: 280

###### Type material.

***Holotype* male** (not studied): “**Suriname**, Lelydorp, /16-XII-1938, in detritus room of *Atta sexdens* nest. Leg. D. C. Geijskes”; several paratypes with same data as type examined by the authors.

###### Other material.

**Brazil**: Amapá, Serra do Navio (0.9833, -52), 1/28/90–2/2/90, FIT, EXO-03034 (CHND, 1ex.); Amapá, Serra do Navio (0.9833, -52), 5/1/91–5/14/91, FIT (CHND, 3ex.); Espírito Santo, Mun. Linhares, Faz. Lagoa do Macuco (-19.0639, -39.9786), 10 m, 1/27/00, FIT, primary lowland Atlantic forest, sandy soil, Génier & S. Ide, SM0809822 (SEMC, 1ex.); Pará, Altamira – Marabá: km 18 (-3.15, -52.05), May 1984, FIT (CHND, 2ex.); Pará, Tucuruí (-3.75, -49.667), 2/16/89–3/2/89, EXO-03033 (CHND, 1ex.); Pará, Tucuruí (-3.75, -49.667), February 1986, FIT, EXO-03032 (CHND, 1ex.); **Guyana**: Kurupukari (4.6667, -58.6667), September-November 1992, Malaise/FIT, EXO-03035 (NHMUK, 1ex.); **Suriname**: Marowijne Perica, 70 km E Paramaribo on East-West Road (5.6744, -54.6086), 5 m, 5/31/99–6/5/99, FIT, Z. Falin, B. DeDijn, SM0182745 (SEMC, 1ex.); Sipaliwini, CI-RAP Survey camp 1, upper Palumeu (2.477, -55.6294), 225 m, 3/10/10–3/16/12, FIT, A.E.Z. Short (AKTC, 2ex.); **Trinidad**: 11 km SE Arima Arena For. Res. Rainforest (10.5, -61.3), 6/22/93–7/8/93, S. & J. Peck, EXO-03036 (AKTC, 1ex.)

###### Diagnostic description.

Length: 1.73–2.01 mm (avg. 1.82 mm); width: 1.50–1.62 mm (avg. 1.54 mm). Body elongate oval, widest behind middle, rufescent, with moderately conspicuous ground punctation; frons convex, not depressed at middle; frontal stria present at sides, continuous with complete supraorbital, broadly interrupted at front; epistoma convex, not depressed; labrum distinctly emarginate; both mandibles with small basal tooth; pronotum with wide, short prescutellar impression, punctate within; pronotal disk with ground punctation only, lacking secondary lateral punctures; marginal pronotal stria complete along sides and front, submarginal stria absent; median pronotal gland openings annulate, ~ 2/3 back from anterior margin; elytron with single, complete epipleural stria; outer subhumeral stria complete, inner absent; dorsal striae 1–4 complete, 4^th^ arched to suture; 5^th^ slightly abbreviated at base, sutural stria obsolete in basal 1/3; all dorsal stria finely, weakly carinate; prosternal keel deeply emarginate, keel striae separate at base, sinuate, connected at apex; prosternal lobe long, subtruncate, with complete marginal stria; mesoventrite produced, with complete marginal stria continued at side by postmesocoxal stria which terminates before reaching mesepimeron; mesometaventral stria angulate at middle, reaching midpoint of mesoventrite, continued at sides by lateral metaventral stria to near middle of metacoxa; metaventrite depressed at middle in males; 1^st^ abdominal ventrite punctate at middle, lacking lateral striae; protibia laterally rounded, with small teeth but very long lateral spines; male protarsus with expanded ventral setae; meso- and metatibiae rather narrow, with elongate but rather fine marginal spines, tarsi elongate. Male: basal piece 1/3 length of tegmen; tegmen with sides subparallel in basal 1/2, abruptly narrowed to ventrally hooked apices; medioventral process absent; median lobe ~ 1/3 tegmen length.

###### Distribution.

Though described only from Suriname, this species in fact has a wide distribution along the east coast of northern South America, even extending to the island of Trinidad.

###### Remarks.

This species stands out among those treated here in having the frons simply convex, not at all impressed medially. It is also distinctive in having the scutellar impression broadly oval, and punctate within, and in having the elytral striae weakly carinate (the outer edge of the stria is elevated, while the inner edge is flat). In some respects (including its *Atta* association) the species resembles *Pseudister rufulus* (Lewis) (and a few others we consider related to it, such as *Phelister rubens* Marseul), which shares a convex frons, deeply emarginate labrum, and spinose tibiae. However, analyses to date don’t support these as closely related. Instead *P. geijskesi* exhibits numerous features that ally it to the species above, including distinctly annulate pronotal gland openings ~ 2/3 removed from the anterior margin (those of *Pseudister* are non-annulate and close to the margin), depressed male mesoventrite, and anterior narrowing of the prosternal keel, in addition to genitalic features.

##### 30. Phelister fraternus sp. nov.

Taxon classificationAnimaliaColeopteraHisteridae

81590FBE-B796-5F13-A63B-5441E8E806AF

http://zoobank.org/57B6ECF4-84A4-43F4-BF43-7131FD42C58B

**Figure 19.**
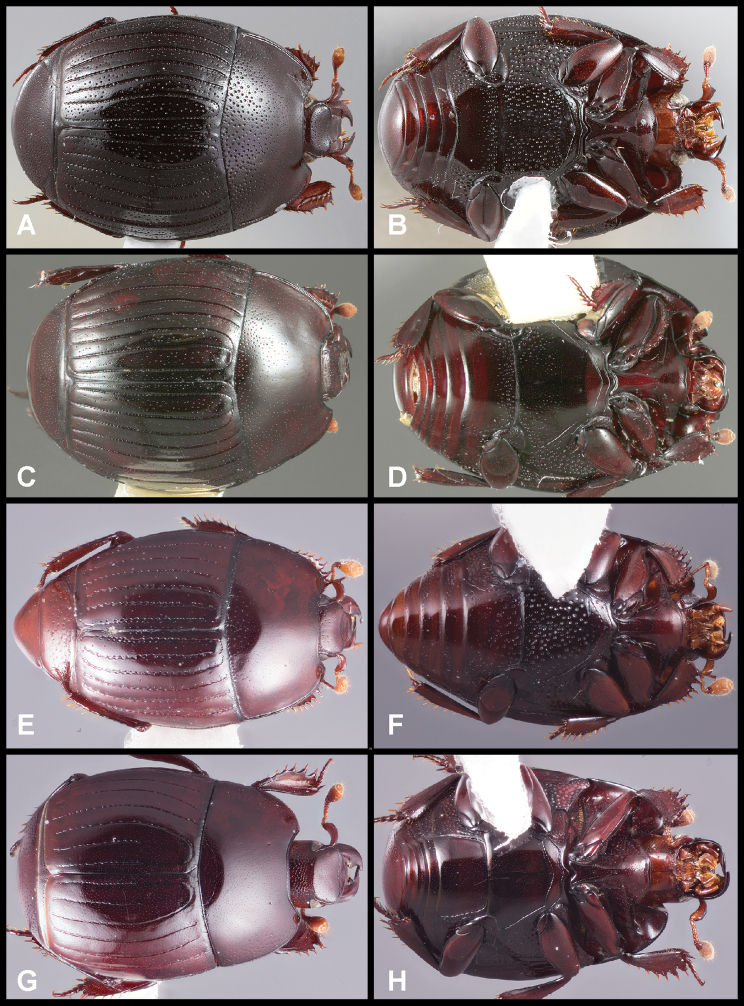
**A, B***Phelister fraternus*: **A** dorsal habitus **B** ventral habitus **C, D***P. conjunctus*: **C** dorsal habitus **D** ventral habitus **E, F***P. chabooae*: **E** dorsal habitus **F** ventral habitus **G, H***P. striatinotum*: **G** dorsal habitus **H** ventral habitus.

**Figure 20.**
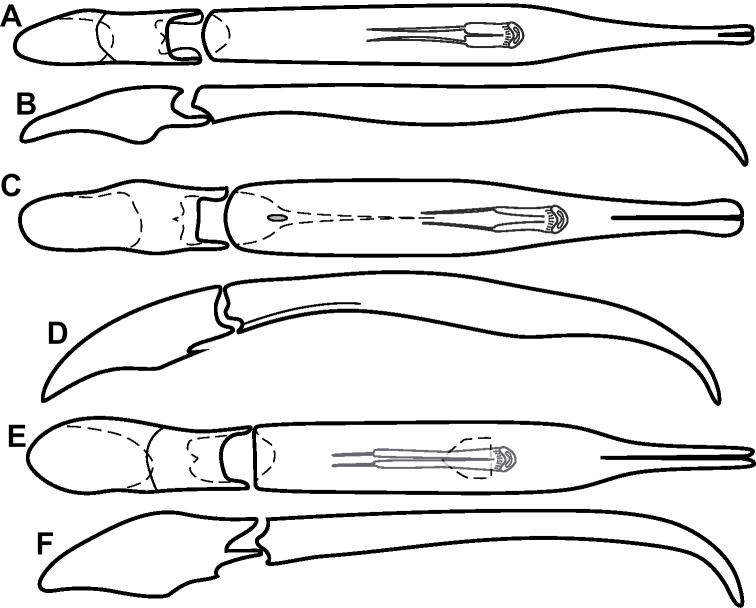
Male genitalia **A, B***Phelister fraternus*: **A** aedeagus, dorsal **B** aedeagus, lateral **C, D***P. conjunctus*: **C** aedeagus, dorsal **D** aedeagus, lateral **E**, **F***P. striatinotum*: **E** aedeagus, dorsal **F** aedeagus, lateral.

Figs 19A, B , 20A, B , Map 12

###### Type material.

***Holotype* male**: “**Guyane Française**: Matoury, Chemin du Lac des Américains, La Désirée 4°50.8'N, 52°19.8'W [4.8467, -52.33] 8.x.2011. SEAG leg.” / “Caterino/Tishechkin Exosternini Voucher EXO-01767 (MNHN).

###### Other material.

**Bolivia**: Santa Cruz, 5 km SSE Buena Vista, Flora y Fauna Hotel (-17.4987, -63.6521), 440 m, 12/15/02–12/24/03, FIT, S. & J. Peck, EXO-00375 (CMNC, 1ex.); **Brazil**: Mato Grosso, Mpio. Cotriguaçu, Fazenda São Nicolau, Matinha (-9.8383, -58.2508), October 2009, FIT, M.S. Gigliotti, EXO-03030 (CEMT, 1ex.).

###### Diagnostic description.

Length: 3.15–3.43 mm (avg. 3.32 mm); width: 2.76–3.11 mm (avg. 2.98 mm). Body large, rounded, strongly convex, castaneous; most surfaces conspicuously punctate, with small ground punctation and significantly larger punctures rather sparsely intermingled; head with frons depressed along midline, weakly produced above antennal bases; supraorbital stria present, disconnected from frontal stria at sides; frontal stria complete, continued finely above epistoma; epistoma laterally weakly carinate, weakly constricted at base, elevated along anterior margin; labrum short, apex weakly emarginate; left mandible with weak basal tooth; pronotum broad, with punctures larger and denser toward prescutellar area; prescutellar impression absent; median pronotal gland openings annulate, located ~ 2/3 from anterior margin; lateral submarginal stria complete, strongly impressed; lateral marginal stria complete and continuous with anterior marginal; elytron with one complete epipleural stria; outer subhumeral striae complete, inner subhumeral slightly abbreviated from base; all other dorsal striae complete, 1^st^-3^rd^ subcarinate, 5^th^ arched to sutural stria; propygidium large, midline length equal to that of pygidium; prosternal keel emarginate at base, with primary striae anteriorly connected, posteriorly divergent; secondary striae present along basal 1/2 of keel; prosternal lobe rounded, marginal stria absent; mesoventrite with strong median projection; marginal mesoventral stria complete, postmesocoxal stria short, ending behind coxa; mesometaventral stria weakly angulate at middle, reaching anterior third of mesoventrite, continued by lateral metaventral stria to inner 1/3 of metacoxa; metaventrite and anterior 1/2 of 1^st^ abdominal ventrite conspicuously punctate; 1^st^ abdominal ventrite with single, incomplete lateral stria along inner edge of metacoxa; protibiae weakly expanded, with inner edge weakly rounded, outer edge weakly 5-dentate, each tooth with small spine; meso- and metatibiae not distinctly expanded, tarsi not compressed. Male: basal piece 1/4 length of tegmen; tegmen with sides subparallel in basal 2/3, abruptly narrowed to ventrally curved apices; medioventral process absent; median lobe ~ 1/4 tegmen length.

###### Etymology.

We name this species *fraternus* as a reference to the ‘brotherhood’ of three similar but non-identical specimens we have available of the species.

###### Distribution.

The entirety of material available for this species is three specimens from three disparate locations (the type locality in French Guiana, plus Santa Cruz, Bolivia and Mato Grosso, Brazil).

###### Remarks.

In having the posterior 1/2 of the pronotum increasingly densely punctate, this species resembles species of *Crenulister*. This shared character may be informative, although they do not resolve together in preliminary phylogenies. This is true of the following two species newly described here, as well, having some characters of *Crenulister*, but resolving apart from it in results of analyses to date.

There is some variation among the specimens studied. The Brazilian example has a more nearly complete inner subhumeral stria, while the one from Bolivia has only a few basal fragments. The Brazilian specimen’s pronotum is also faintly explanate.

##### 31. Phelister conjunctus sp. nov.

Taxon classificationAnimaliaColeopteraHisteridae

5BE2C3CE-FF7E-58E7-A58B-5A5313A78296

http://zoobank.org/3286F567-1258-4D7A-B1A7-AE4E7E54B279

Figs 19C, D , 20C, D , Map 12

###### Type material.

***Holotype* male**: “**Guyane Française**: Mont Tabulaire Itoupé 3°1.82'N, 53°6.40'W [3.0303, -53.1067], 400 m, Piège d’interception No. 3. 31 Mar 2010. SEAG leg.” / “, Caterino/Tishechkin Exosternini Voucher EXO-00378” (MNHN); ***Paratypes* (2): French Guiana**: Belvédère de Saül, point de vue (3.6228, -53.2094), 17.1.2011, FIT, SEAG, EXO-03029 (CHND, 1ex.); Rés. Natur. des Nouragues, Saut Pararé. 4°02'N, 52°41'W. Piège vitre, 20.iv.2010. SEAG leg. (CHND, 1ex.).

###### Diagnostic description.

Length: 4.02–4.65 mm (avg. 4.32 mm); width: 3.90–4.10 mm (avg. 4.03 mm). Body very large, elongate oval, strongly convex, piceous; ground punctation conspicuous, most surfaces with conspicuous but sparse secondary punctures; head with frons weakly depressed along midline, weakly produced above antennal bases; supraorbital stria present, disconnected from frontal stria at sides; frontal stria interrupted at middle; epistoma weakly elevated at sides; labrum with apex emarginate; left mandible with moderate basal tooth; pronotum broad, with secondary punctures more numerous in posterior 1/2; prescutellar area weakly depressed; main median pronotal gland openings weakly annulate, with one pair at anterior margin, also apparently multiplied along a short track reaching 2/3 from anterior margin; lateral submarginal stria complete, strongly impressed; lateral marginal stria complete and continuous with anterior marginal; anterior margin weakly produced behind head; elytron with one complete epipleural stria; outer subhumeral, inner subhumeral and all dorsal striae complete, 4^th^ arched toward base of 5^th^, but free, 5^th^ arched to sutural stria, all striae connected by apical arches along elytral apex; propygidium midline length nearly equal to that of pygidium; prosternal keel emarginate at base, with primary striae narrowly connected in front, posteriorly divergent but connected along basal margin; weak secondary striae present along basal 1/2 of keel; prosternal lobe short, emarginate apically, marginal stria absent; mesoventrite with strong median projection; marginal mesoventral stria complete, slightly disconnected from postmesocoxal stria, which extends just beyond mesocoxa; mesometaventral stria broadly angulate at middle, reaching anterior third of mesoventrite, continued by lateral metaventral stria toward, but not reaching, middle of metacoxa; metaventrite with cluster of secondary punctures near metacoxa; 1^st^ abdominal ventrite conspicuously punctate in anterior 1/2, with incomplete lateral stria along inner edge of metacoxa; protibia weakly expanded, outer edge weakly dentate, each tooth with small spine; meso- and metatibiae not distinctly expanded, tarsi not compressed. Male: basal piece 1/3 length of tegmen; tegmen with sides subparallel in basal 1/2, abruptly narrowed, then weakly expanded to ventrally hooked apices; medioventral process absent; median lobe ~ 1/4 tegmen length.

###### Etymology.

This species name refers to the apically ‘conjunct’ or united elytral striae.

###### Distribution.

This species is only known three close locations in French Guiana.

###### Remarks.

This species appears very closely related to *P. fraternus* (above), and as discussed under that species, has much in common with species we have assigned to *Crenulister*. It lacks the large, spatulate protarsal setae of *Crenulister*, and has quite dissimilar genitalia. Otherwise, compared to those and to *P. fraternus*, it has finer and sparser pronotal punctation, and has its pronotal gland openings apparently multiplied, with two or three openings in a longitudinal series, with larger irregular surrounding annuli. All its elytral striae are complete and most are also connected along the elytral apex.

##### 32. Phelister chabooae sp. nov.

Taxon classificationAnimaliaColeopteraHisteridae

5CA58FC0-2180-5F3C-BCB8-0ADD491719AE

http://zoobank.org/4EC6955C-C49F-41AA-885C-14A4608E2B1F

Fig. 19E, F , Map 12

###### Type material.

***Holotype* female**: “**Peru**: Madre de Dios, CICRA Field Stn., ~ 2 km NW cafeteria res. plot. 12.55236°S, 70.10989°W, 295 m, Flight intrcept. 11–13.vi.2010, Chaboo Team PER-11-FIT-019” / “Caterino/Tishechkin Exosternini Voucher EXO-03628” (SEMC).

###### Diagnostic description.

Length: 2.56 mm; width: 2.13 mm. Body elongate oval, slightly flattened, rufescent; ground punctation fine, with very few, larger secondary punctures; head with supraorbital stria weakly connected to frontal stria at sides; frontal stria complete, slightly recurved at middle; epistoma with sides and apical margin subcarinate; labrum with apex emarginate; left mandible lacking basal tooth, right mandible with very small basal tooth; pronotum broad, with secondary punctures more numerous toward prescutellar area, prescutellar impression small, poorly defined; median pronotal gland openings small, non-annulate, approximately 1/3 from anterior margin; lateral submarginal stria complete, strongly impressed; anterior margin weakly produced behind head; elytron with single, complete epipleural stria; outer subhumeral stria complete, crenulate; inner subhumeral stria absent; dorsal striae 1–5 and sutural stria complete, the 4^th^ arched to base of sutural stria, all striae appearing as series of connected punctures, the punctures alternating sides of the connecting line; propygidium with sparse secondary punctures mostly in basal 1/2; pygidium narrowed to apex, bluntly acute, lacking secondary punctures; prosternal keel deeply emarginate at base, somewhat broad, striae subparallel, connected in front, posteriorly divergent; prosternal lobe deflexed, rounded, with complete marginal stria; mesoventrite with strong median projection; marginal mesoventral stria complete, continued by postmesocoxal stria to mesepimeron; mesometaventral stria angulate at middle, reaching anterior third of mesoventrite, continued by lateral metaventral stria to middle of metacoxa; metaventrite and anterior 1/2 of abdominal ventrite one with dense secondary punctures; abdominal ventrite one with incomplete lateral stria along inner edge of metacoxa; protibia weakly expanded, with outer edge rounded and strongly dentate, with ~ 8 marginal spines; protarsal ventral setae expanded, leaf-like; meso- and metatibiae not distinctly expanded, with numerous long marginal spines; meso- and metatarsi slender, with long ventral spines.

###### Etymology.

We name this species for Dr. Caroline Chaboo, in recognition of her exceptional work on documenting the beetle fauna of Peru.

###### Distribution.

This species is only known from the type locality in Madre de Dios, Peru.

###### Remarks.

This species may represent a connection between this group of *Phelister* and the recently described genus *Crenulister* (Caterino and Tishechkin 2014). We have debated whether it belongs there, lacking a few characters of that genus (and lacking confirmation from male genitalia). It shares some characters, including the broadly rounded and moderately flattened body form, the strongly spinose tibiae, the more conspicuous secondary punctures along the posterior portion of the pronotal disk, the broadly crenulate elytral striae, and the spatulate tarsal setae (not restricted to males). However, it differs from most of them in lacking a pygidial stria, lacking an inner subhumeral elytral stria, and having the middle of the anterior pronotal margin rather distinctly produced over the head. For now, we describe this distinct species in *Phelister*, and recognize that the relationships between these genera requires further examination.

##### 33. Phelister striatinotum

Taxon classificationAnimaliaColeopteraHisteridae

Wenzel & Dybas, 1941

E50EF1D7-52D3-522C-B6C6-7D4438DF28BD

Figs 19G, H , 20E, F , Map 12

Phelister striatinotum Wenzel & Dybas, 1941: 463.
Phelister striatinotus ; Mazur, 2011: 29.

###### Type material.

***Holotype***, of undetermined sex: “Villavicencio, VII:24:38 Colombia” / “col. by H.Dybas” / “Collection R. L. Wenzel” / “Type Phelister striatinotum Wenzel + Dybas” / “Phelister striatinotum Wenzel & Dybas” (FMNH).

###### Other material.

**Argentina**: Corrientes, Rio Riachuelo, Peunte Pexoa at 27.560°S, 58.723°W, 50 m, 12/20/12–12/25/12, K.P. Tomkovich (AVSC, 1ex.); **Bolivia**: Cochabamba, Est. Biol. Valle Sajta, 67.5 km E Villa Tunari (-17.1053, -64.7825), 300 m, 2/9/99–2/13/99, FIT, lowland rain forest, F. Génier, EXO-03490 (CMNC, 1ex.); Cochabamba, 117 km E Cochabamba, at Lagunitas (-17.1061, -65.6825), 1000 m, 2/8/99–2/12/99, FIT, mountain evergreen forest, F. Génier, EXO-03491 (CMNC, 1ex.); Santa Cruz, Amboro National Park, Los Volcanes, c. (-18.1, -63.6), 1000 m, 11/20/04–12/12/04, FIT, Mendel, H. & Barclay, M.V.L., EXO-03492 (NHMUK, 1ex.); Santa Cruz, ~ 5 km SSE Buena Vista, Flora y Fauna Hotel, 17.498°S, 63.652°W, 440 m, flight intercept trap, 14–31.xii.2003, S. & J. Peck (AKTC, 2ex.); **Brazil**: Acre, Cruzeiro do Sul (-7.6333, -72.6), January-February 1988, FIT, EXO-03496 (CHND, 1ex.); Mato Grosso, Mpio. Cuiaba, Fazenda Mutuca (-15.3145, -55.9703), 9/23/08, FIT, F.H. Gava & J.R. Rocha, EXO-03497 (CEMT, 1ex.); **Costa Rica**: Guanacaste, Est. Pitilla, P.N. Guanacaste, 9 km S Sta. Cecilia (10.99, -85.4), 700 m, January 1994, C. Moraga (INBIO, 2ex.); Heredia, Est. Biol. La Selva, 3 km S Pto. Viejo (10.4333, -84.0167), 7/27/76, H.A. Hespenheide, EXO-03495 (INBIO, 1ex.); Heredia, Est. Biol. La Selva, 3 km S Pto. Viejo (10.4333, -84.0167), 6/20/91, Riding *Atta* cut leaf, H.A. Hespenheide, EXO-03494 (INBIO, 1ex.); Limon, Amubri, A.C. Amistad (9.5, -82.9), 70 m, 5/2/94–5/31/94, G. Gallardo, INBIO CRI001 872229 (INBIO, 1ex.); **Ecuador**: Napo, Tena (-1, -78), 2000ft, 2/14/23–2/28/23, FX William, EXO-03493 (USNM, 1ex.); Orellana, Est. Biodiv. Tiputini (-0.6376, -76.1499), 7/30/08, FIT, A. Tishechkin, EXO-00702 (AKTC, 1ex.); Orellana, Est. Cientifica Yasuní, mid. Rio Tiputini (-0.675, -76.4), 7/4/99–7/17/99, FIT, C. Carlton & A. Tishechkin, LSAM 0045446 (LSAM, 1ex.); **French Guiana**: Régina, Rés. Natur. des Nouragues (4.0378, -52.6725), 2/19/10, FIT, SEAG, EXO-00807 (CHND, 1ex.); Roura (8.4 km SSE) (4.6781, -52.2236), 200 m, 5/29/97–6/10/97, FIT, J. Ashe & R. Brooks, EXO-03488 (CMNC, 1ex.); Mont Tabulaire Itoupé (3.022, -53.0842), 800 m, 3/30/10, FIT, SEAG, EXO-03487 (CHND, 1ex.); **Guyana**: Mazaruni-Potaro, Takutu Mountains (6.25, -58.9167), 12/8/83, FIT, in montane rainforest near logging area, P.D. Perkins & W.E. Steiner, EXO-00366 (USNM, 1ex.); Kurupukari (4.6667, -58.6667), September-November 1992, Malaise/FIT, EXO-03489 (NHMUK, 1ex.); **Paraguay**: Cazaapa, San Rafael Reserve, Hermosa, prop. Lopez family (-26.3081, -55.7508), 80 m, 12/1/00–12/3/00, FIT, Z. Falin, EXO-00447 (SEMC, 1ex.); Cazaapa, San Rafael Reserve, Hermosa, prop. Lopez family, bank Rio Tebicuary (-26.2897, -55.7186), 80 m, 12/1/00–12/4/00, FIT, Z. Falin, SM0275458 (SEMC, 1ex.); **Peru**: Loreto, Compamento San Jacinto (-2.3125, -75.8628), 175–215 m, 7/3/93–7/12/93, FIT, R. Leschen, EXO-03499 (CHSM, 1ex.); Loreto, Iquitos (-3.74, -73.27), 90 m, 5/7/92, FIT, J. Danoff-Berg, EXO-03498 (SEMC, 1ex.); Madre de Dios, Rio Alto Madre de Dios, Pantiacolla Lodge (-12.655, -71.2317), 11/14/07–11/19/07, FIT, D. Brzoska, EXO-03500 (SEMC, 1ex.).

###### Diagnostic description.

Length: 1.97–2.60 mm (avg. 2.34 mm); width: 1.65–2.21 mm (avg. 1.97 mm). Body elongate, widest at humeri, slightly flattened, rufescent, with moderately dense and conspicuous ground punctation; frons broad, only weakly depressed at middle; supraorbital stria absent; frontal stria complete, weakly recurved at middle; labrum weakly emarginate at apex; left mandible with broad basal tooth, right mandible with narrower blunt tooth; prescutellar impression very wide, with anterior edge sinuate, ~ 6 × as wide as the scutellum; pronotal disk with slightly larger secondary punctures interspersed at sides; median pronotal gland openings annulate, ~ 1/3 behind anterior margin; marginal pronotal stria complete along sides and front; submarginal stria absent; elytron with single, complete epipleural stria; outer subhumeral stria complete, inner absent; dorsal striae 1–4 and sutural complete, 4^th^ arched to sutural stria, 5^th^ obsolete in basal 1/3; propygidium with sparse, small secondary punctures, pygidium largely lacking secondary punctures; prosternum emarginate at base, striae convergent to apex; prosternal lobe rather long, subtruncate, with deep, complete marginal striae; mesoventrite strongly produced, with complete marginal stria continued at sides by postmesocoxal stria, which ends freely at middle of lateral portion of metaventrite; mesometaventral stria angulate at middle, reaching middle of mesoventrite, continued at sides by lateral metaventral stria to inner 1/3 of metacoxa; 1^st^ abdominal ventrite with one complete and one incomplete lateral striae; protibia rather strongly dentate, with ~ five marginal spines; spines of meso- and metatibiae fine and long, but largely confined to distal 1/2; ventral setae of tarsi simple, not flattened. Male: basal piece 1/3 length of tegmen; tegmen with sides subparallel in basal 2/3, abruptly narrowed to ventrally hooked apices; medioventral process absent; median lobe ~ 1/3 tegmen length.

###### Distribution.

This species is very widely distributed, from Costa Rica in the north to Argentina in the south, with most other records coming from western Amazonia. The species is also known from the Guianas.

###### Remarks.

This is a widespread and rather variable species. The very wide prescutellar impression, with its anterior margin distinctly sinuate, is its most consistent diagnostic feature. This, along with pronotal gland openings that are near the anterior third of the pronotal disk, will identify it unambiguously. Compared to other species treated in this paper, its frons is relatively weakly depressed, and the tooth on the base of the left mandible is quite strong. Its relationships to species immediately above are not very strongly supported, since this species has a completely divided male 10^th^ tergite (it is partly or completely fused in all the preceding species of the *amazoniae* subgroup).

Nearly all specimens have been collected using flight interception traps. However, one specimen from La Selva, Costa Rica was ‘Riding [an] *Atta* Cut Leaf’, suggesting that leafcutter ant nests would be worth exploring for this species.

##### 34. Phelister notandus

Taxon classificationAnimaliaColeopteraHisteridae

Schmidt, 1893

15014E78-58A7-549B-A028-97A67996F84E

Fig. 21A, B

Phelister notandus Schmidt, 1893b: 82.

###### Type material.

***Lectotype***, of undetermined sex, hereby designated: “Brasil” / “coll. J.Schmidt” / “notandus Schm.” / “Type” / “***Lectotype****Phelister notandus* Schmidt, 1893, M.S.Caterino & A.K.Tishechkin des. 2010 (ZMHB).

**Figure 21.**
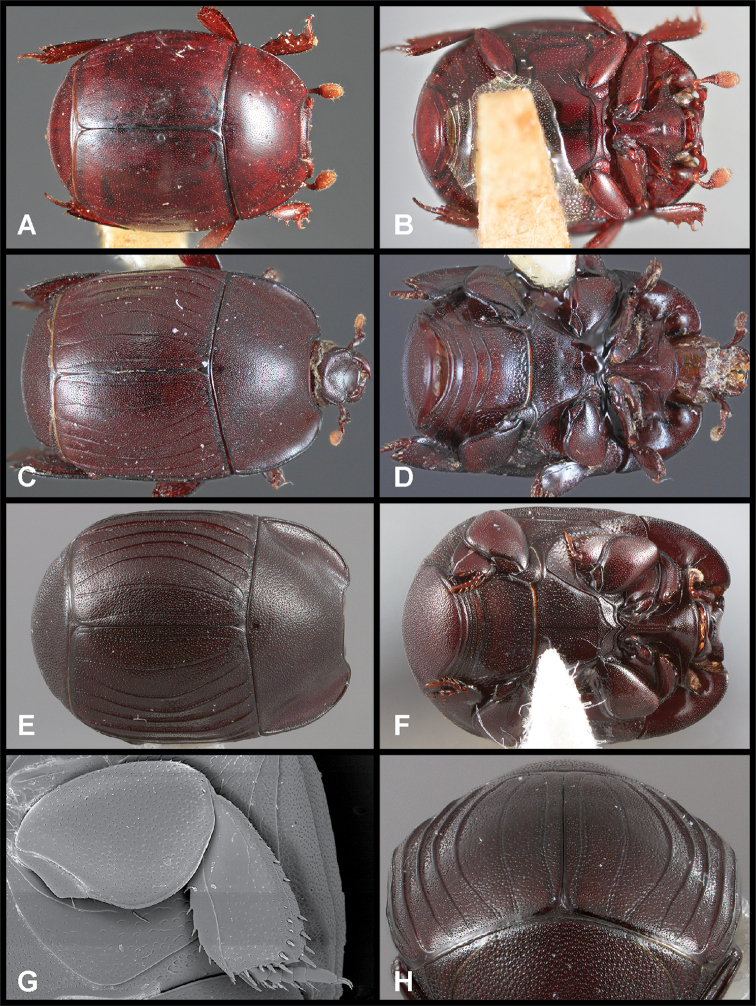
**A, B***Phelister notandus*: **A** dorsal habitus **B** ventral habitus **C, D***Phelister amazoniae*: **C** dorsal habitus **D** ventral habitus **E–H***P. arcuatus*: **E** dorsal habitus **F** ventral habitus **G** mesothoracic leg **H** posterior view showing carinate elytral striae.

###### Diagnostic description.

Length: “3 mm” (Schmidt 1893b). Body rounded, slightly elongate, widest behind humeri, rufescent, with rather dense and conspicuous ground punctation; frons broad, depressed at middle; supraorbital stria absent; frontal stria strongly recurved at middle briefly interrupted; labrum shallowly emarginate at apex; prescutellar impression weakly impressed, narrower than scutellum; pronotal disk with few lateral secondary punctures; median pronotal gland openings annulate, ~ 4/5 behind anterior margin; marginal pronotal stria complete along sides and front; submarginal stria absent; elytral striae very reduced, crowded to side; outer subhumeral stria interrupted but present at base and apex; inner subhumeral present in apical 2/3; striae one and two complete; stria three present at base only; 4^th^, 5^th^ and sutural striae absent except for basal arch; propygidium large, as long along midline as pygidium, with dense ground punctation and only slightly larger secondary punctures interspersed; pygidium largely lacking secondary punctures; prosternal keel emarginate at base, striae convergent to apex; prosternal lobe short, with fine marginal striae; mesoventrite produced, with complete marginal stria continued at sides by postmesocoxal stria, which ends freely at middle of lateral portion of metaventrite; mesometaventral stria sinuate at middle, reaching nearly to mesoventral stria, continued at sides by lateral metaventral stria toward metepipleuron; abdominal ventrites obscured by point; all tibiae slightly broadened; protibia strongly dentate, with ~ five marginal spines; spines of meso- and metatibiae weak, most confined to distal 1/2.

###### Distribution.

This species is only known from the type, with only the unfortunately vague location “Brasil”.

###### Remarks.

Despite not being able to examine male genitalia for this species, its placement in this subgroup seems secure. It is a rather autapomorphic species, yet showing moderate development of some characters that are more extremely expressed in some of the following species, including dense punctation and expanded tibiae. It is hoped that additional material of this species is discovered to allow us to more comprehensively evaluate its relationships, and to pinpoint its geographic range.

##### 35. Phelister amazoniae

Taxon classificationAnimaliaColeopteraHisteridae

(Lewis, 1898) comb. nov.

9B2A14B8-D854-54DC-9274-A635C263B5D4

**Figure 22.**
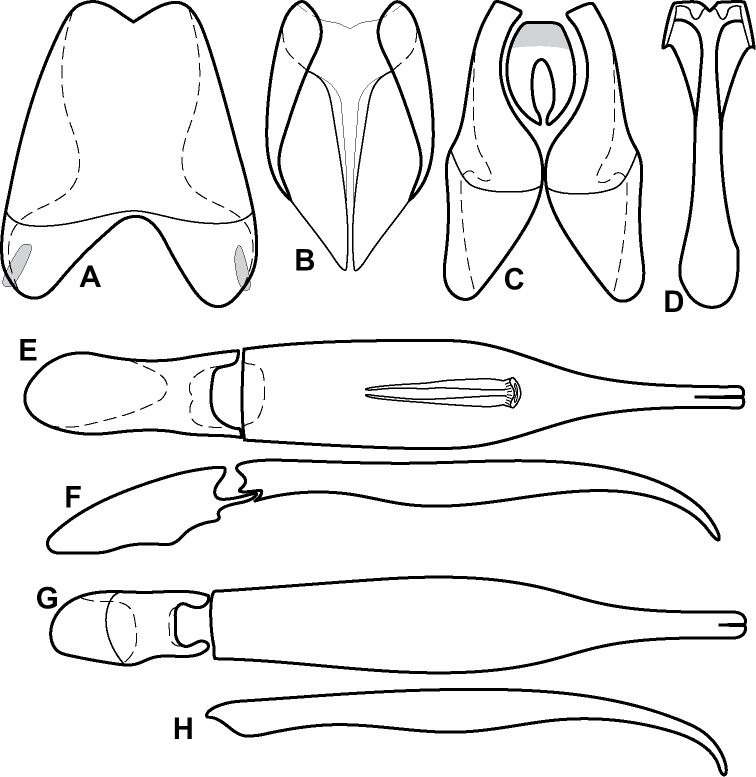
Male genitalia **A–F***Phelister amazoniae*: **A** tergite 8 **B** sternite 8 **C** tergites 9 & 10 **D** sternite 9 **E** aedeagus, dorsal **F** aedeagus, lateral **G, H***P. arcuatus* (outlines only, to contrast with **E** and **F**): **G** aedeagus, dorsal **H** tegmen, lateral.

**Map 13.**
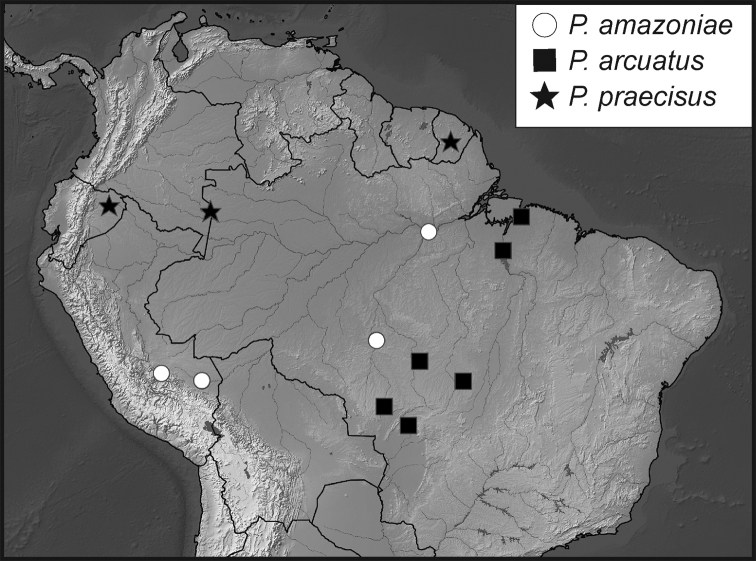
Collecting records for *Phelister amazoniae* (white circles), *P. arcuatus* (black squares), and *P. praecisus* (stars).

Figs 21C, D , 22A–F , Map 13

Discoscelis amazoniae Lewis, 1898: 176.
Reninus amazoniae ; Mazur, 1984: 312; 2011: 129.
Brachylister amazoniae ; Bickhardt, 1917: 234.

###### Type material.

***Lectotype***, sex uncertain, hereby designated: “Santarem” / “H.H. Smith 1898” / “Discoscelis amazoniae Type. Lewis” / “G.Lewis Coll. B.M.1926-369.” / “Type” (NHMUK).

###### Other material.

**Brazil**: Mato Grosso, Mpio. Cotriguaçu, Fazenda São Nicolau, Mata Norte (-9.8192, -58.26), 12/8/10–12/14/10, FIT, F.Z. Vaz-de-Mello (CEMT, 28ex.); Mato Grosso, Mpio. Cotriguaçu, Fazenda São Nicolau, Matinha (-9.8383, -58.2508), October, 2009, FIT, M.S. Gigliotti (CEMT, 5ex.); **Peru**: Cusco, La Convencion, Echarate, CN Timpia (-12.0621, -72.8516) (MUSM, 1ex.); Madre de Dios, Rio Los Amigos, CICRA (-12.5709, -70.1018), 25–150 m, 11/24/06–11/26/06, FIT, A. Asenjo (MUSM Lima, 3ex.); Madre de Dios, Rio Los Amigos, CICRA (-12.5521, -70.1096), 6/9/11–6/11/11, FIT, C. Chaboo (SEMC, 2ex.); Madre de Dios, Rio Los Amigos, CICRA (-12.5526, -70.1101), 6/9/11–6/11/11, FIT, C. Chaboo (SEMC, 2ex.); Madre de Dios, Rio Los Amigos, CICRA (-12.5524, -70.1099), 6/11/11–6/13/11, FIT, C. Chaboo (SEMC, 2ex.); Madre de Dios, Los Amigos Field Station, 12.5624°S, 70.0930°W, 288 m, terra firme forest, pitfall trap, 2–11.i.2007, J. Jacobs (CASC, 5ex.).

###### Diagnostic description.

Length: 2.72–3.11 mm (avg. 2.95 mm); width: 2.40–2.64 mm (avg. 2.56 mm). Body large, elongate, with sides subparallel, strongly convex, dark rufescent to castaneous; most surfaces densely punctate; head with frons depressed along midline, strongly produced above antennal bases, densely punctate, punctures interconnected by fine reticulations; supraorbital stria present, but disconnected from frontal at sides; frontal stria complete along sides, weakly continued above epistoma, may be finely interrupted at middle, also continued anterad by lateral marginal epistomal striae; epistoma constricted at base, swollen along anterior margin, labrum short, apically emarginate; mandibles with incisor edges rather short, without marginal teeth; pronotum broad, densely punctate, most punctures uniform in size, disk explanate along sides; prescutellar impression smaller than scutellum, weakly defined; median pronotal gland openings distinctly annulate, located ~ 2/3 from anterior margin; lateral submarginal stria complete, deeply impressed; lateral marginal stria complete and continuous with anterior marginal; elytral subhumeral striae and all dorsal striae complete, outer striae deeply impressed and carinate, 4^th^ stria arched to sutural stria; punctures of elytral disk distinctly interconnected by fine reticulations; propygidium large, midline length equal to that of pygidium, with two sizes of punctures (small and smaller) intermingled, intervening surface finely microsculptured; pygidium with deep, complete marginal stria, punctation finer and denser than that of pygidium; prosternal keel deeply emarginate at base, narrowed, with median striae complete, subparallel, connected anteriorly; prosternal lobe short, rounded, with complete marginal stria; mesoventrite rather narrow, with strong median projection; marginal mesoventral stria may be weakly interrupted at middle, continued by postmesocoxal stria to outer 1/4 of metaventrite; mesometaventral stria angulate at middle to midpoint of mesoventrite, continued by lateral metaventral stria to middle of metacoxa, then by recurrent stria anterad to middle of metepisternum; 1^st^ abdominal ventrite with broadly depressed, complete lateral stria along inner edge of metacoxa; all femora and tibiae broadly expanded; protibia with rounded inner and outer edges, with four or five marginal spines, spurs present, small; meso- and metafemora with posterior margin strongly produced; meso- and metatibiae broad and flat, with weak marginal spines; tarsi stout, compressed, together little longer than the tibiae are broad. Male: basal piece just > 1/3 length of tegmen; tegmen with sides subparallel to slightly widened in basal 1/2, abruptly narrowed to ventrally hooked apices; medioventral process absent; median lobe simple, ~ 1/3 tegmen length.

###### Remarks.

Although this species was described from Pará state, Brazil, all recent material we have seen is from much further south and west, from Mato Grosso to Amazonian Peru. Nonetheless, the distinctive characters are clearly shared with the type specimen.

This species is very similar and closely related to the following one, but the presence of a pygidial stria in the present species will consistently distinguish them. In most specimens of *P. amazoniae*, the pronotal punctation is simple, comprising consistent-sized punctures, and the elytral punctures are joined by a very fine network of reticulations. Specimens of *P. amazoniae* from Peru (Madre de Dios, La Convencion) tend to have a doubly punctate pronotum, like *P. arcuatus*, as well as non-reticulate elytral punctation, but based largely on their possessing a marginal pygidial stria we retain these here. These two species are also very similar to another new species (*P. dilatatus*, #23 above), which also shares the large convex, generally punctate body, as well as broadly expanded tibiae. However, remarkably, this appears to be convergence, as the male genitalia are quite different. *Phelister dilatatus* is also easily distinguished by the non-explanate pronotum and distinctly toothed (not externally rounded) protibia.

This species has previously been assigned to a genus of Haeteriinae. However, it clearly lacks critical characters of that subfamily (fused labrum, pyramidal antennal scape, sclerotized sides of antennal club, lack of tibial spurs), and is only superficially similar to members of *Reninus* Lewis. Among other characters, its male genitalia clearly ally it to this subgroup of the *Phelister blairi* group.

###### Distribution.

Known from Pará and Mato Grosso states in Brazil, and Madre de Dios and La Convencion, Peru.

##### 36. Phelister arcuatus sp. nov.

Taxon classificationAnimaliaColeopteraHisteridae

9819BBFD-8305-5F63-9F93-185CA7971A84

http://zoobank.org/2A497928-F64A-4C5D-8F05-5C086B778DF4

Figs 21E–H , 22G, H , Map 13

###### Type material.

***Holotype* male**: “Cuyaba [-15.6, -56.1] M. Grossa **Brazil**” / “CNHM Colln. (ex. Colln. C. A. Ballou)” / “Brachylister” (Discoscelis) amazoniae Lewis” / “Caterino/Tishechkin Exosternini Voucher EXO-03000” (FMNH); ***Paratypes* (19): Brazil**: Mato Grosso, Mpio. Claudia (-11.2416, -55.325), 10/17/10–10/27/10, FIT, A.F. Oliveira, EXO-02999 (CEMT, 1ex.); Mato Grosso, Mpio. Querencia, Fazenda São Luiz (-12.597, -52.3749), July 2008, FIT, R. Andrade, (CEMT, 2ex.); Mato Grosso, Mpio. Querencia, Fazenda São Luiz (-12.597, -52.3749), February 2009, FIT, R. Andrade (CEMT, 11ex.); Mato Grosso, Mpio. Tangara da Serra (-14.3175, -57.7317), 640 m, 1/22/09–1/29/09, FIT, R.S. Silva, EXO-02996 (CEMT, 1ex.); Pará, Belém, Utinga (IPEAN) (-1.45, -48.4333), December 1984, FIT (CHND, 2ex.); Pará, Belém, Utinga (IPEAN) (-1.45, -48.4333), October, 1986, FIT, N. Degallier, EXO-00004 (CHND, 1ex.); Pará, Tucuruí (-3.75, -49.667), 12/5/87–12/17/87, FIT, EXO-00825 (CHND, 1ex.).

###### Diagnostic description.

Length: 2.36–2.99 mm (avg. 2.77 mm); width: 2.01–2.80 mm (avg. 2.55 mm). This species is very similar and closely related to the preceding, and is only described here to the extent that they differ. Body smaller; pronotal discal punctures distinctly ‘doubled’ (two sizes of punctures intermingled); elytral striae slightly crowded laterad, with the 4^th^ stria distinctly outwardly arcuate; inner subhumeral and 1^st^ through 4^th^ dorsal striae more distinctly carinate; pygidium lacking marginal stria. Male genitalic morphology generally very similar to the preceding species, but aedeagus narrower and slightly flatter than that of *P. amazoniae*, with the basal wide portion accounting for more of the overall tegmen length, basal piece shorter.

###### Etymology.

This species’ name refers to the strongly outwardly bowed 4^th^ (and other) elytral striae.

###### Distribution.

This species is known from Pará and Mato Grosso states, Brazil.

###### Remarks.

This species is broadly sympatric with the preceding, but the differences are consistent across this range, particularly the lack in the present species of a marginal pygidial stria. Both species are known from males and females, precluding the possibility that their differences represent sexual dimorphism.

#### *P. gregarius* subgroup

This subgroup comprises six species, united by a number of characters. Of them, the divided medioventral process of the aedeagus seems to be a strong synapomorphy.

Genitalic characters:

- Medioventral process of tegmen present but divided
- Accessory sclerites of segment 8 minute or absent
- T8 elongate, with broad ventrolateral apodemes that often meet beneath
- S8 apex broad, flat, lacking setae
- Ventrolateral hooks of T9 small and very near apex
- S9 with rather broad bowl-shaped head, minimal apical emargination
- T10 divided
- Apico-ventral margin of basal piece lacking projection (indeed, usually emarginate)

External characters:

- Protibia with dominant apical spine located basad strong apical emargination
- Male metaventrite may be depressed
- Elytra conspicuously punctate
- Pronotum somewhat elongate

##### 37. Phelister gregarius sp. nov.

Taxon classificationAnimaliaColeopteraHisteridae

E4CEBCC0-FB48-54A5-A0FF-443985FE4F75

http://zoobank.org/DCEC4A58-2D81-4BB8-9231-34EA5321A935

**Map 14.**
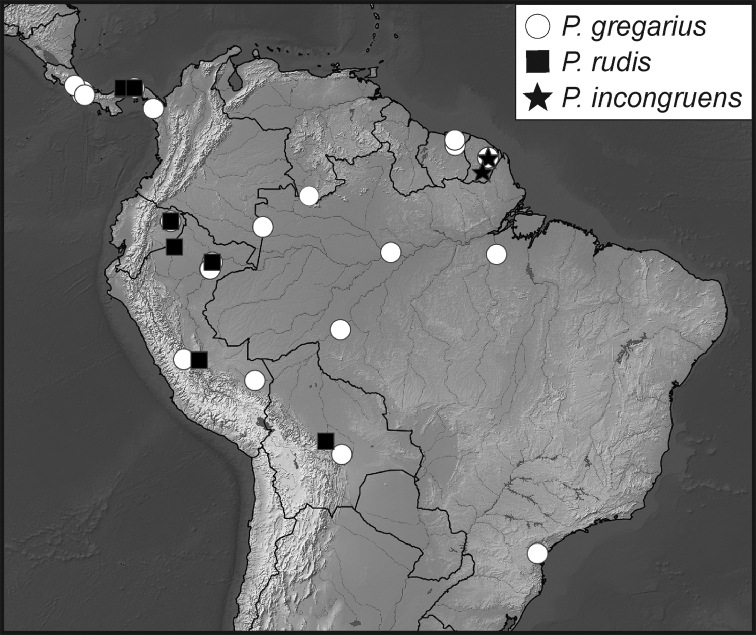
Collecting records for *Phelister gregarius* (circles), *P. rudis* (black squares), and *P. incongruens* (stars).

**Figure 23.**
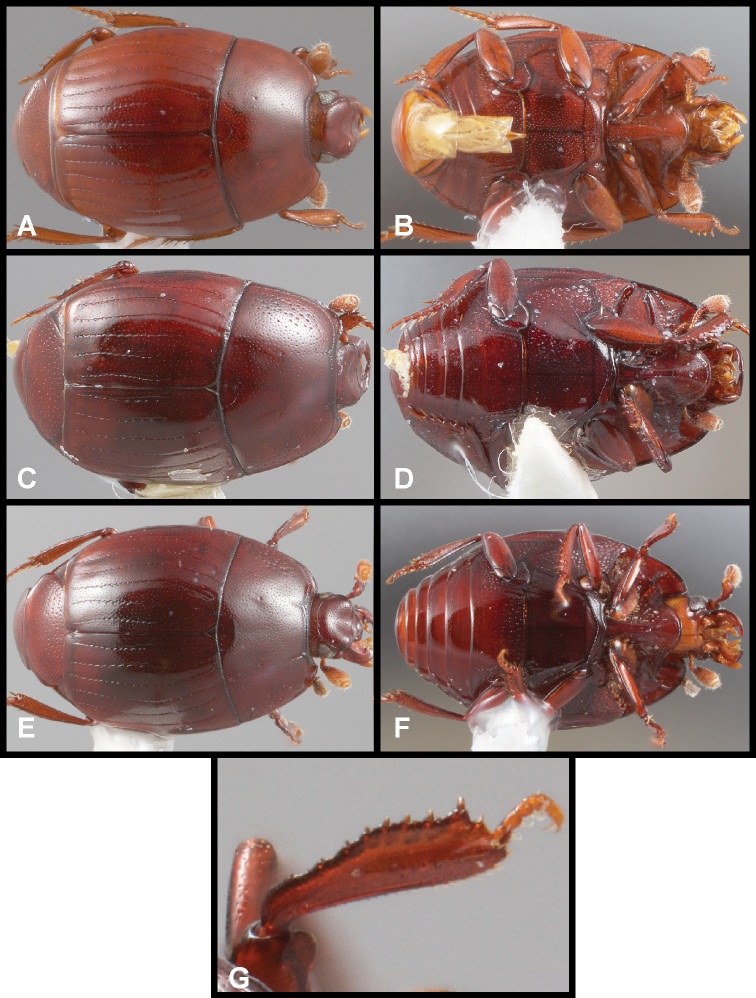
**A, B***Phelister gregarius*: **A** dorsal habitus **B** ventral habitus **C, D***P. praecisus*: **C** dorsal habitus **D** ventral habitus **E–G***P. rudis*: **E** dorsal habitus **F** ventral habitus **G** anterior view of protibia.

**Figure 24.**
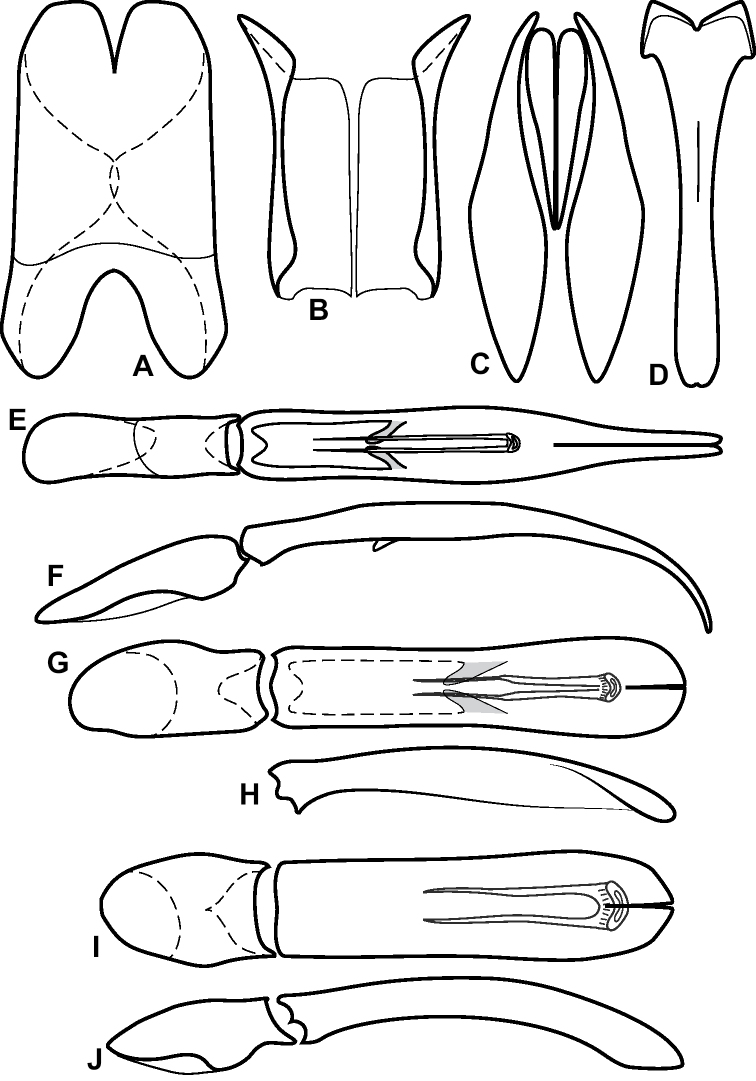
Male genitalia **A–F***Phelister gregarius*: **A** tergite 8 **B** sternite 8 **C** tergites 9 & 10 **D** sternite 9 **E** aedeagus, dorsal **F** aedeagus, lateral **G, H***P. praecisus*: **G** aedeagus, dorsal **H** tegmen, lateral **I, J***P. rudis*: **I** aedeagus, dorsal **J** tegmen, lateral.

Figs 23A, B , 24A–F , Map 14

###### Type material.

***Holotype* male**: “**Peru**: Dept. Loreto, Iquitos – Nauta rd., km 58, Rio Itaya at 4°15.738'S, 73°28.052'W [-4.2563, -73.4675]. 120m” / “Window trap next to entrance into *Eciton burchelli* statary bivouac in a hollow tree 5–9 May 2009. A.V.Petrov” / “Caterino/Tishechkin Exosternini Voucher EXO-00466 (FMNH). ***Paratypes* (46): Peru**: same data as type (22ex.); Loreto, Iquitos (-3.74, -73.27), 90 m, 5/5/92, FIT, J. Danoff-Berg, EXO-03341 (SEMC, 1ex.); Loreto, km 63, rd. Iquitos – Nauta, Rio Itaya (-4.2534, -73.4346), 140 m, 2/10/10–2/14/10, A.V. Petrov, EXO-03340 (AKTC, 1ex.); **Ecuador**: Orellana, Est. Cientifica Yasuní, mid. Rio Tiputini (-0.675, -76.4), 6/28/99–7/5/99, FIT, C. Carlton & A. Tishechkin, LSAM 0045453 (LSAM, 1ex.); Orellana, Est. Cientifica Yasuní (-0.675, -76.4), 7/11/08–7/26/08, FIT, A. Tishechkin (AKTC & LSAM, 5ex.); Orellana, Est. Cientifica Yasuní (-0.675, -76.4), 7/18/99, Berlese of litter and refuse deposit at *Eciton burchelli* bivouac site just after emigration, A. Tishechkin, EXO-00730 (AKTC, 1ex.); Orellana, Est. Cientifica Yasuní (-0.675, -76.4), 7/13/08, Sifting litter at recently abandoned bivouac site of *Eciton burchelli* statary phase colony, A. Tishechkin (AKTC, 3ex.); Orellana, Est. Biodiv. Tiputini (-0.64, -76.15), 7/29/08–8/3/08, FIT, A.K.Tishechkin (AKTC, 3ex.); Orellana, Est. Biodiv. Tiputini (-0.64, -76.15), 8/3/08–8/6/08, FIT, LSAM Team (LSAM, 2ex.); **Colombia**: Vaupes, Est. Biol. Caparu, Rio Apoporis (-1.1, -69.5), 9/27/95–12/1/95, FIT, Black-water terrace forest on sandy soils, B. Gill (AKTC, 3ex.); Vaupes, Parco Nac. Mosiro-Itajura (Caparu), Centro Ambiental (-1.0667, -69.5167), 60 m, 1/20/03–1/30/03, FIT, D. Arias & M. Sharkey (AKTC, 1ex.).

###### Other material.

**Bolivia**: Santa Cruz, Amboro National Park, Los Volcanes (-18.1, -63.6), 1000 m, 11/20/04–12/12/04, FIT, Mendel H. & Barclay, M.V.L. (NHMUK, 2ex.); **Brazil**: Amazonas, Reserva Ducke, AM-010, km 26 (-3, -59.94), 12/2/77, J. Arias, EXO-03349 (USNM, 1ex.); Pará, Altamira – Marabá: km 18 (-3.15, -52.05), May 1984, FIT, EXO-03348 (CHND, 1ex.); Paraná, Piraquara (-25.4961, -48.9817), 9/3/07–9/10/07, P. Grossi, EXO-03347 (DZUP, 1ex.); Rondonia, Rio Jamari (-8.7833, -63.7), 12/1/88–12/12/88, FIT, EXO-03350 (CHND, 1ex.); **Costa Rica**: Puntarenas, Est. Biol. Las Alturas, Coto Brus (8.95, -82.8667), 1550 m, 3/25/03–3/30/03, FIT, A. Cline & A. Tishechkin, EXO-03343 (LSAM, 1ex.); Puntarenas, Est. Biol. Las Cruces (8.7833, -82.9583), 1100 m, 4/10/02, Sifting at recently abandoned bivouac site of *Eciton burchelli* nomadic phase bivouac, A. Tishechkin, EXO-00744 (LSAM, 1ex.); Puntarenas, Est. Pittier, Coto Brus, Senderos Pittier y Pelton (9.06, -82.93), 1670 m, 11/7/99, R. Gonzalez, INB0003153030 (INBIO, 1ex.); Puntarenas, San Vito Las Cruces (8.8, -83), 1200 m, 7/1/82–7/30/82, FIT, B. Gill, EXO-03344 (AKTC, 1ex.); San Jose, Cloudbridge Reserve, 2.4 km ENE Sn Gerardo de Rivas, Ridge trail (9.4678, -83.564), 2050 m, 6/9/04–6/12/04, FIT, J. Ashe, Z. Falin, I. Hinojosa, SM0659028 (SEMC, 1ex.); **French Guiana**: Régina, Rés. Natur. des Nouragues, Camp Inselberg (4.0833, -52.6833), 1/25/11, FIT, SEAG, EXO-03351 (CHND, 1ex.); Belvédère de Saül, point de vue (3.6228, -53.2094), 1/31/11, FIT, SEAG (CHND, 1ex.); **Panama**: Chiriqui, 27.7 km W. Volcan Hartmann’s Finca (8.75, -82.8), 1450 m, 6/14/95–6/17/95, FIT, J. Ashe & R. Brooks, EXO-03352 (SEMC, 1ex.); Chiriqui, 4 km N Sta. Clara Hartmann’s Finca (8.75, -82.8), 1500 m, 6/30/82–7/13/82, FIT, B. Gill (AKTC, 2ex.); Darien, Cana Biological Station, Serrania de Pirre (7.755, -77.685), 1380 m, 6/4/96–6/7/96, FIT, J. Ashe & R. Brooks, SM0050908 (SEMC, 1ex.); Panama, Chepo-Charti Rd. (9.28, -79.1), 400 m, June 1982, FIT, B. Gill, EXO-03353 (CNCI, 1ex.); **Peru**: Junín, Pampa Hermosa Lodge, 22 km N San Ramon (-10.9883, -75.425), 1220 m, 11/14/07–11/27/07, FIT, D. Brzoska, SEMC0869361 (SEMC, 1ex.); Junín, 11 km NE Puerto Ocopa, Los Olivos (-11.05, -74.2587), 1200 m, 3/26/09–3/31/09, FIT, A. Petrov & A. Tishechkin (AKTC, 5ex.); Madre de Dios, Rio Los Amigos CICRA(Puerto) (-12.5709, -70.1018), 230 msm, 8/5/06, Colectados en: basurero de *Eciton* sp., A. Asenjo (MUSM Lima, 3ex.); Cusco, Villa Carmen Fld Stn., 12.89221°S, 71.41946°W, 555 m, 20–30.V.2011, DJ Bennett, FIT (SEMC, 1ex.); **Suriname**: Brokopondo, Ston Eiland Eco Resort nr. Brownsberg (4.9833, -55.1333), 2/10/10–2/13/10, FIT, C. Gillet, P. Skelley, W. Warner, EXO-03345 (FSCA, 1ex.); Pará, 11 km SE Zanderij Airport (5.3933, -55.1581), 30 m, 6/20/99, FIT, Z. Falin, EXO-03346 (CMNC, 1ex.); **Venezuela**: Amazonas, Cerro de la Neblina, base camp (0.8333, -66.1667), 140 m, 1/26/85–1/31/85, FIT, R.A. Faitoute, P.J. & P.M. Spangler & W.E. Steiner (USNM, 1ex.).

###### Diagnostic description.

Length: 1.22–1.54 mm (avg. 1.39 mm); width: 1.14–1.26 mm (avg. 1.20 mm). Body elongate oval, rufescent, smooth, but with conspicuous ground punctation throughout; frons depressed along midline; supraorbital stria present, ends free; frontal stria complete, fine at middle; epistoma with edges raised but not subcarinate; labrum emarginate; mandibles lacking basal teeth; prescutellar impression subtriangular, with basal corners rounded, ~ 2 × as wide as scutellum; median pronotal gland openings ~ 2/3 behind anterior margin, distinctly annulate; pronotal disk with few sparse secondary punctures at sides, most slightly elongate and separated by their lengths or more; marginal pronotal stria complete along lateral and anterior margins, crenulate in front; submarginal stria short, present only in anterior corners, rarely absent; elytron with single, complete epipleural stria; outer subhumeral stria nearly complete, slightly abbreviated at base, inner subhumeral absent; dorsal striae 1–4 complete, 5^th^ present in apical 3/4 and as short basal arch, sutural stria present in apical 2/3; propygidium with dense, coarse secondary punctation; pygidium with ground punctation enlarged and dense; prosternal keel barely emarginate at base, striae converging sinuately to near apex; apex of keel abruptly narrowed at junction with prosternal lobe; prosternal lobe short, with complete marginal stria; mesoventrite weakly produced, with weak, complete marginal stria interrupted laterally by mesocoxa; postmesocoxal stria ending at middle of lateral portion of metaventrite; mesometaventral stria broadly arcuate across middle, reaching middle of mesoventrite, offset laterad at sides from lateral metaventral stria; lateral metaventral stria extending from mesometaventral suture to near middle of metacoxa; metaventrite with few secondary punctures near metacoxa, that of male weakly depressed at middle and with very short scale-like vestiture; 1^st^ abdominal ventrite coarse secondary punctation in anterior 1/2, with fragmented but nearly complete lateral stria along inner margin of metacoxa; protibia rather slender, outer margin weakly dentate, with five or six marginal spines, apical corner with distinct emargination; protarsi of male with flattened ventral setae; meso- and metatibiae slender, with rather long, fine marginal spines. Male: basal piece narrow, elongate, ~ 1/2 length of tegmen; tegmen with sides unevenly subparallel in basal 2/3, abruptly narrowed to ventrally curving apices; medioventral process present, projecting at basal 1/3 of tegmen, divided at middle; median lobe ~ 1/2 tegmen length, very narrow.

###### Etymology.

This species name refers to its apparent predilection for socializing with army ant colonies, from which several records originate.

###### Distribution.

This is a relatively common species, known from many specimens over a broad area from Costa Rica south to Peru, and east to Paraná, Brasil.

###### Remarks.

Despite this species’ wide range, it is relatively consistent in morphology, and readily identifiable, by the rounded triangular shape of its prescutellar impression, relatively sparse lateral secondary punctures on the pronotum, and presence of only a short fragment of the submarginal pronotal stria in the anterior corners. It also appears to be unique in showing a distinct sexual dimorphism, with the males’ metaventrite being medially depressed as well as bearing a field of very short, almost imperceptible, scale-like setae. A few other species show a faint depression in males, but none as distinct as here.

Two specimens from Santa Cruz, Bolivia, and one from Junín, Peru have a complete lateral submarginal pronotal stria, and have pronotal microsculpture (like *P. rudis*), but have sparser dorsal sculpturing and an unbroken anterior marginal pronotal stria, so are ambiguous. We place these here for now, but additional material will be necessary to fully assess patterns of character variation. Specimens from Suriname have the lateral submarginal pronotal stria almost absent, represented by only a very short arc in the anterior corners.

##### 38. Phelister praecisus sp. nov.

Taxon classificationAnimaliaColeopteraHisteridae

E42C9D80-BFC6-5CFB-9A91-FEBC9409A10B

http://zoobank.org/D5AAF7BC-18F8-496B-B5F5-D694F03009A1

Figs 23C, D , 24G, H , Map 13

###### Type material.

***Holotype* male**: “**French Guiana**: Saül, 7 km N, Les Eaux Claires 220 m, 3°39'46"N, 53°13'19"W [3.6628, -53.2219], 30 MAY–4 JUN 1997; J.Ashe, R.Brooks, FG1AB97 ex:flight intercept trap” / “SM0095852 KUNHM-ENT” (SEMC).

###### Other material.

**Colombia**: Vaupes, Parco Nac. Mosiro-Itajura (Caparu) Centro Ambiental (-1.0667, -69.5167), 60 m, 1/20/03–1/30/03, FIT, D. Arias & M. Sharkey, EXO-02966 (MSCC, 1ex.); **Ecuador**: Orellana, Yasuni NP, Via Maxus at Puente Piraña, 0°39.5'S, 76°26'W, 245 m, flight intercept trap, 20–24.vii.2008, A. K. Tishechkin (AKTC, 2ex.); **Peru**: Madre de Dios, Rio Alto Madre de Dios Pantiacolla Lodge 12°39.3'S, 71°13.9'W [-12.6550, -71.2317], Flight intercept 14–19 Nov 2007, D.Brzoska, SEMC0903642 (SEMC, 1ex.).

###### Diagnostic description.

Length: 1.89–2.17 mm (avg. 2.03 mm); width: 1.58–1.93 mm (avg. 1.71 mm). Body elongate oval, dark rufescent, with conspicuous ground punctation throughout; frons depressed along midline; supraorbital stria present, ends free; frontal stria complete, fine at middle; frontal disk with ground punctation but lacking secondary punctures; epistoma broad, with edges raised, subcarinate; labrum weakly emarginate; left mandible with blunt basal tooth, that of right mandible small, subacute; pronotum slightly elongate, sides weakly convergent; prescutellar impression broadly oval, ~ 4 × as wide as scutellum; median pronotal gland openings ~ 2/3 behind anterior margin, distinctly annulate; pronotal disk with numerous secondary punctures at sides; marginal pronotal stria complete along lateral and anterior margins, very close to margin anteriorly; submarginal stria present at sides and front, lateral portion complete (French Guiana) or abbreviated (others), anterior portion detached from lateral, briefly recurved behind eyes; elytron with single, complete epipleural stria; outer subhumeral stria fine, nearly complete, slightly abbreviated at base, inner subhumeral absent; dorsal striae 1–4 complete, 5^th^ present in apical 3/4 and as short basal arch, sutural stria slightly longer than 5^th^, obsolete at base; propygidium with secondary punctures separated by ~ 1.5 × their diameters; pygidium with only few small secondary punctures along base; prosternal keel shallowly emarginate at base, striae united at base and apex, enclosing narrow area; secondary striae present along basal 1/2 of keel; prosternal lobe slightly reflexed, with complete marginal stria; mesoventrite weakly produced, with very fine, complete marginal stria continued laterally by postmesocoxal stria to middle of lateral portion of metaventrite; mesometaventral stria weakly angulate at middle, reaching anterior third of mesoventrite, continued at sides by lateral metaventral stria; lateral metaventral stria extending from mesometaventral suture to near outer 1/3 of metacoxa; metaventrite with few small secondary punctures near metacoxa; 1^st^ abdominal ventrite with complete inner and partial outer lateral striae along inner margin of metacoxa; protibia with outer margin strongly dentate, with five or six marginal spines, apical corner with shallow emargination (mediad apical spine); protarsi of male with flattened ventral setae; meso- and metatibiae with rather long, fine marginal spines. Male: basal piece wide, ~ 1/3 length of tegmen; tegmen with sides subparallel in basal 1/2, then widened to spoon-shaped apex; apical lobes produced laterally over main body of tegmen; medioventral process present, weak, not projecting ventrally, divided at middle; median lobe ~ 1/2 tegmen length, basal apodemes narrowed.

###### Etymology.

This species name, *praecisus*, translates as cut, referring to the interrupted anterior pronotal stria

###### Distribution.

This species is known from a few localities across northern Amazonia, Ecuador and Colombia, as well as French Guiana.

###### Remarks.

This species is difficult to characterize, because it varies considerably in morphology among available material. All series are short and most are all female. We designate as type a singleton male from French Guiana, which differs from most other specimens in its complete lateral submarginal pronotal stria; this is abbreviated posteriorly in most (but not all) others. All available specimens share a very broadly semicircular prescutellar impression, a pattern of dorsal punctation with the pronotal ground punctation slightly denser than that of the elytra (though neither is very dense), and the presence of a number of secondary punctures on the metaventrite anteromediad the hind coxae. The interrupted 5^th^ elytral stria (with a basal arch) is unusual, as is their prosternal keel that is weakly emarginate at base, narrow, with its striae united anteriorly and posteriorly.

##### 39. Phelister rudis sp. nov.

Taxon classificationAnimaliaColeopteraHisteridae

EBC50890-EAC9-5473-8098-9A9CF2184F4B

http://zoobank.org/D5494642-A523-46AE-886B-62B775592D33

Figs 23E–G , 24I, J , Map 14

###### Type material.

***Holotype* male**: “**Panama**: Colón Prov., San Lorenzo Forest, STRI crane site. 9°17'N, 79°58'W [9.2833, -79.9667], FIT-Z-7, 19–20 May 2004, A. Tishechkin. AT-478” / “Phelister sp #16, San Lorenzo Inventory, A.L.Tishechkin det 2010” / “Caterino/Tishechkin Exosternini Voucher EXO-00444” (FMNH). ***Paratypes* (3): Panama**: Colon, 14 km N jct. Escobal & Pina Rds. (9.2, -79.9), 6/2/96–6/11/96, FIT, J. Ashe & R. Brooks, SM0041133 (SEMC, 1ex.); Colon, San Lorenzo Forest, STRI crane site (9.2833, -79.9667), 5/19/04–5/25/04, FIT, A. Tishechkin (AKTC, 1ex.); Panama, Chepo-Charti Rd. (9.28, -79.1), 400 m, June 1982, FIT, B. Gill, EXO-03337 (CNCI, 1ex.).

###### Other material.

**Bolivia**: Cochabamba, Cochabamba, 67.5 km NE Est. Biol. Valle del Sajita Univ. de San Simon (-17.1092, -64.7978), 300 m, 2/7/99–2/9/99, FIT, R. Hanley, SM0161392 (SEMC, 1ex.); **Ecuador**: Orellana, Est. Cientifica Yasuní, mid. Rio Tiputini (-0.675, -76.4), 6/23/99–6/30/99, FIT, C. Carlton & A. Tishechkin, LSAM 0045443 (LSAM, 1ex.); Orellana, Est. Biodiv. Tiputini (-0.64, -76.15), 7/28/08–7/31/08, FIT, A.K.Tishechkin (AKTC, 2ex.); Orellana, Est. Cientifica Yasuní (-0.675, -76.4), 7/11/08–7/26/08, FIT, A. Tishechkin (AKTC & LSAM, 2ex.); Orellana, Est. Cientifica Yasuní, mid. Rio Tiputini (-0.675, -76.4), 07/5–11/1999, FIT, C. Carlton & A. Tishechkin, LSAM 0045451 (LSAM, 1ex.); Orellana, Est. Cientifica Yasuní, mid. Rio Tiputini (-0.675, -76.4), 8/6/99, *Eciton burchelli* colony EC#27, refuse deposit during statary phase, A. Tishechkin, LSAM 0045444 (LSAM, 1ex.); **Peru**: Junín, 11 km NE Puerto Ocopa, Los Olivos (-11.05, -74.2587), 1200 m, 3/23/09–3/24/09, FIT, A. Tishechkin, EXO-00948 (AKTC, 1ex.); Loreto, 1.5 km N Teniente Lopez (-2.5943, -76.1153), 210–240 m, 7/20/93, FIT, R. Leschen, EXO-03338 (SEMC, 1ex.); Loreto, Iquitos (-3.74, -73.27), 90 m, 5/5/92, FIT, J. Danoff-Berg, EXO-03339 (SEMC, 1ex.); Loreto, km 63, rd. Iquitos – Nauta, Rio Itaya (-4.2534, -73.4346), 140 m, 2/05/10–2/06/10, A.V. Petrov (AKTC, 1ex.); Junín, ~ 16 km NW Satipo, Rio Venado (-11.1989, -74.7705), 1500 m, 3/08/10–3/14/10, A.V. Petrov (AKTC, 1ex.); Junín, ~ 16 km NW Satipo, Rio Venado (-11.1989, -74.7705), 3/03/10–3/08/10, A.V. Petrov (AKTC, 2ex.).

###### Diagnostic description.

Length: 1.69–1.89 mm (avg. 1.80 mm); width: 1.42–1.54 mm (avg. 1.48 mm). This species is extremely similar to the preceding two (*P. gregarius* and *P. praecisus*), differing principally in the following characters: frons with microsculpture within frontal depression; prescutellar impression broadly semi-oval; mandibles each with distinct basal tooth; pronotum with marginal stria continuous around sides and front; submarginal pronotal stria present in anterior 1/2 at sides, anterior portion broken and recurved behind eyes; outer subhumeral elytral stria present in apical 1/2 only, rarely with detached basal appendix (especially in Peruvian examples); prosternal keel weakly narrowed anteriorly, with striae complete, united anteriorly only; prosternal lobe short; mesometaventral stria strongly angulate to near marginal mesoventral stria; male metaventrite not depressed; protibia with apical spine prominent, other marginal spines rather fine and slightly separated from it. Male: basal piece broad, short, ~ 1/3 length of tegmen; tegmen with sides subparallel in basal 1/2, then widened slightly to rounded apices, thick and notably curved in lateral view; medioventral process absent; median lobe ~ 1/2 tegmen length, with basal apodemes widely separated.

###### Etymology.

The name *rudis*, or rough, refers to the unusual microsculpture of the frons.

###### Distribution.

This species is known from Panama as well as much of western Amazonia, from Ecuador to Bolivia.

###### Remarks.

We assign specimens from a broad area, ranging from Panama, southward along western South America to Bolivia, to this species. However, we restrict the type series to Panamanian specimens, as there is some variation, and other areas are poorly represented. Most specimens (all those from the type locality) exhibit distinct microsculpture within the frontal depression. This is quite unusual in this group, and within *Phelister* as a whole (being otherwise occasional in some *Operclipygus* spp.). The species is quite similar in other respects to *P. gregarius* and *P. praecisus*. From the former it consistently differs by its broadly oval prescutellar impression (subtriangular in *P. gregarius*) and the broken anterior submarginal pronotal stria. From *P. praecisus* it differs in having a shorter outer subhumeral stria, and prosternal keel striae that are not united at the base. Specimens from Peru tend to have several metaventral punctures, which is otherwise only found in *P. gregarius* (at least among these species), but these males don’t have a depressed metaventrite, as those of *P. gregarius* do.

##### 40. Phelister incongruens sp. nov.

Taxon classificationAnimaliaColeopteraHisteridae

B7A90493-5AFD-5196-B237-1A2A94505336

http://zoobank.org/E0DB2EAA-0086-45BD-BEE5-3CCE7CBD578D

**Figure 25.**
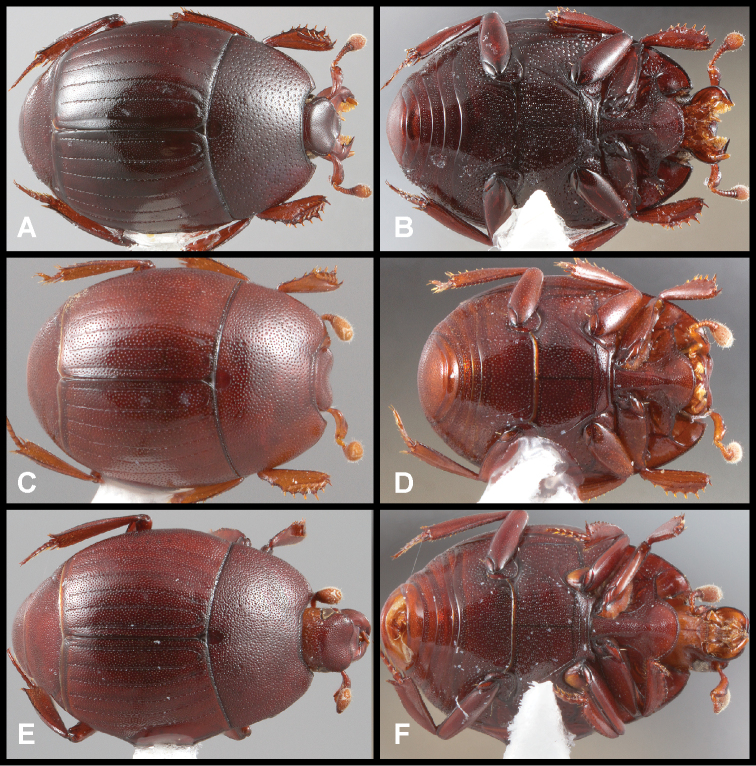
**A, B***Phelister incongruens*: **A** dorsal habitus **B** ventral habitus **C, D***P. congruens*: **C** dorsal habitus **D** ventral habitus **E, F***P. praesignis*: **E** dorsal habitus **F** ventral habitus.

**Figure 26.**
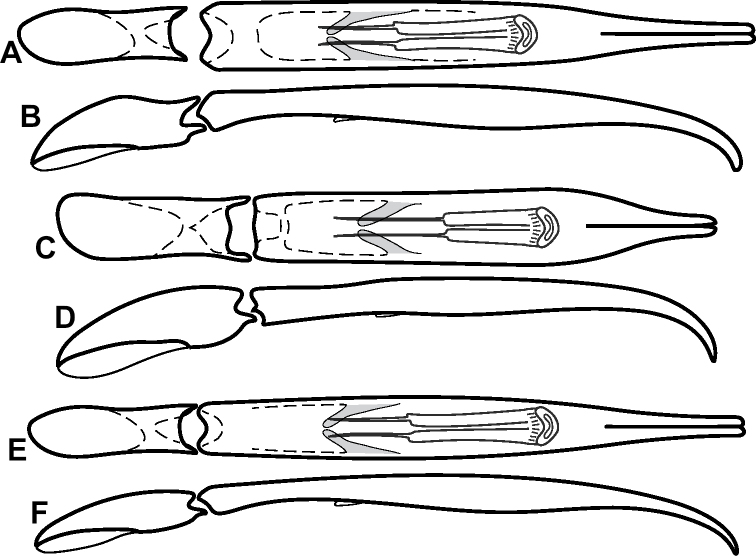
Male genitalia **A, B***Phelister incongruens*: **A** aedeagus, dorsal **B** aedeagus, lateral **C, D***P. congruens*: **C** aedeagus, dorsal **D** aedeagus, lateral **E**, **F***P. praesignis*: **E** aedeagus, dorsal **F** aedeagus, lateral.

Figs 25A, B , 26A, B , Map 14

###### Type material.

***Holotype* male**: “**Guyane Française**: Réserve des Nouragues 4°43'N, -52°24'W [4.0378, -52.6725], piège d’interception 30 Sep 2009, SEAG leg.” / “Caterino/Tishechkin Exosternini Voucher EXO-00455 (MNHN). ***Paratypes* (4): French Guiana**: Régina, Rés. Natur. des Nouragues, Camp Inselberg (4.0833, -52.6833), 1/25/11, FIT, SEAG, EXO-03220 (CHND, 1ex.); Mont Tabulaire Itoupé (3.0303, -53.1067), 400 m, 3/17/10, FIT, SEAG, EXO-03357 (CHND, 1ex.); Mont Tabulaire Itoupé (3.0303, -53.1067), 400 m, 3/23/10, FIT, SEAG, EXO-00373 (CHND, 1ex.); Amazone Nature Lodge, 300 m, 4.55033°N, 52.17031°W flight intercept. 11–23.viii.2017, R.Morris & J.Wappes” (AKTC 1ex.).

###### Diagnostic description.

Length: 1.93–2.29 mm (avg. 2.15 mm); width: 1.62–1.97 mm (avg. 1.85 mm). Body elongate oval, convex, variably rufescent, with very conspicuous ground punctation everywhere, on pronotum uniformly intermingled with secondary punctation; frons weakly depressed; supraorbital stria present, ends free; frontal stria fine, complete; epistoma broad, with edges raised; labrum wide, deeply emarginate; left mandible with short, blunt basal tooth, that of right mandible small, acute; prescutellar impression slightly elongate, only slightly wider than scutellum; median pronotal gland openings obscured by punctation, possibly absent; marginal pronotal stria complete along lateral margin; lateral submarginal stria complete, continuous with marginal stria behind head; elytron with single, complete epipleural stria; outer subhumeral stria fine, present at base and apex, narrowly interrupted at middle, inner subhumeral absent; dorsal striae 1–5 and sutural complete, 5^th^ arched to sutural stria; propygidium with small secondary punctures sparsely intermingled with ground punctation, that of pygidium finer and mainly in basal 1/2; prosternal keel weakly emarginate at base, striae united at base and apex, secondary striae present along basal 1/2 of keel, keel narrowed anteriorly; prosternal lobe short, with complete marginal stria; mesoventrite weakly produced, with complete marginal stria continued laterally by postmesocoxal stria which ends behind posterior corner of mesocoxa; mesometaventral stria arcuate across middle, reaching middle of mesoventrite, continued at sides by lateral metaventral stria to middle of metacoxa; metaventrite of male weakly depressed, with very dense ground punctation, with larger secondary punctures near metacoxa; metaventrite of female flat, with larger secondary punctures; 1^st^ abdominal ventrite with two incomplete lateral striae, the anterior 1/2 of disk with dense secondary punctures; protibia with outer margin weakly dentate but strongly spinose, with five or six marginal spines, the apical most particularly prominent, with a shallow emargination along apical margin; meso- and metatibiae with rather long, fine marginal spines largely restricted to distal halves. Male: basal piece narrow, ~ 1/3 length of tegmen; tegmen with sides subparallel in basal 2/3, abruptly narrowed to hooked apices; medioventral process present, projecting at basal 1/3 of tegmen, divided at middle; median lobe ~ 1/2 tegmen length, abruptly narrowed to bases.

###### Etymology.

The name refers to the very different, incongruous, punctation on the pronotum vs. the elytra.

###### Distribution.

This species is known only from a couple of localities in French Guiana.

###### Remarks.

This species is distinct in the distribution of surface sculpture, with its ground punctation conspicuous everywhere, that on the pronotum uniformly interspersed with coarse secondary punctation (not restricted to sides). Additionally, the venter exhibits distinct macrosculpture all over. The interrupted outer subhumeral stria is an unusual character but is seen in a few other species in this group. It is particularly similar to *P. praesignis* but has the secondary punctation of the pronotum larger yet slightly sparser, and the ground punctation of the pronotum and especially the elytra distinctly finer. It is also more broadly rounded, and has a complete submarginal pronotal stria, absent in *P. praesignis*.

While all (five) specimens of this species are from French Guiana, they fall into two distinct size classes, with the smaller ones (two males, one female) also lighter in color. However, no other differences are apparent, and we assume we have not captured the full range of variation with so few specimens.

##### 41. Phelister congruens sp. nov.

Taxon classificationAnimaliaColeopteraHisteridae

0E9E2825-6577-5D93-AE9C-4F9E61ABF78F

http://zoobank.org/13505EFC-8B7A-43A5-A56D-EF55751B4F19

**Map 15.**
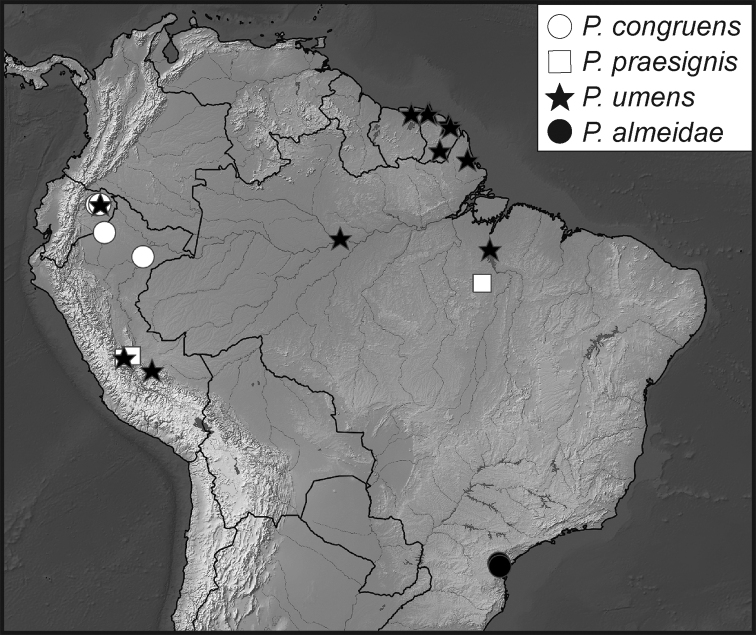
Collecting records for *Phelister congruens* (white circles), *P. praesignis* (square), *P. umens* (stars), and *P. almeidae* (black circles).

Figs 25C, D , 26C, D , Map 15

###### Type material.

***Holotype* male**: “**Peru**: Dept. Loreto, 1.5 km N Teniente Lopez -2°35.66'S, 76°06.92'W [-2.5943, -76.1153], 18 July 1993, 210–240 m, Richard Leschen #119 ex:flight intercept trap” / “SEMC0903645 KUNHM-ENT” (SEMC). ***Paratypes* (13)**: **Colombia**: Vaupes, Parco Nac. Mosiro-Itajura (Caparu), Centro Ambiental (-1.0667, -69.5167), 60 m, 1/20/03–1/30/03, FIT, D. Arias & M. Sharkey, EXO-03437 (AKTC, 1ex.); **Peru**: Loreto, Iquitos – Nauta rd., km 58, Rio Itaya (-4.2563, -73.4675), 120 m, 5/5/09–5/9/09, Window trap next to entrance into *Eciton burchelli* statary bivouac in a hollow tree, A.V. Petrov (AKTC, 2ex.); **Ecuador**: Orellana, Est. Cientifica Yasuní (-0.6744, -76.6472), 215 m, 9/5/99–9/10/99, FIT, primary forest, E. Riley (LSAM, 2ex.); Orellana, Est. Cientifica Yasuní (-0.6744, -76.6472), 7/12/08–7/24/08, FIT, A. Tishechkin (AKTC, 5 ex.); Orellana, Est. Cientifica Yasuní (-0.675, -76.4), 7/12/08–7/19/08, FIT, A. Tishechkin (AKTC & LSAM, 2ex.); Orellana, P.N. Yasuní, Via Maxus at Puente Piraña (-0.6583, -76.4333), 7/14/08–7/20/08, FIT, A. Tishechkin, EXO-03356 (AKTC, 1ex.).

###### Diagnostic description.

Length: 1.38–1.77 mm (avg. 1.56 mm); width: 1.22–1.58 mm (avg. 1.37 mm). Body elongate oval, convex, rufescent, with very conspicuous ground punctation, and no secondary punctation throughout; frons depressed along midline; supraorbital stria present, ends free; frontal stria complete; epistoma broad, with edges raised; labrum emarginate; left mandible with short, blunt basal tooth, that of right mandible small, acute; prescutellar impression semicircular, ~ twice as wide as scutellum; median pronotal gland openings obscured by ground punctation, but apparently present ~ 3/4 behind anterior pronotal margin, within a small elongate impunctate area, not distinctly annulate; marginal pronotal stria complete along lateral and anterior margins; submarginal stria absent; elytron with single, complete epipleural stria; outer subhumeral stria fine, present at base and apex, interrupted at middle, inner subhumeral absent; dorsal striae 1–5 complete, 5^th^ arched to sutural, the sutural stria obsolete in basal one-sixth; propygidium with secondary punctures separated by ~ 1.5 × their diameters; pygidium with only few small secondary punctures along base; prosternal keel subtruncate at base, striae united at base and apex, secondary striae present along basal 1/2 of keel; prosternal keel and meso- & metaventral disks of male bearing very short scale-like setae; prosternal lobe short, with complete marginal stria; mesoventrite not, or only weakly produced, with complete marginal stria continued laterally by postmesocoxal stria to middle of lateral portion of metaventrite; mesometaventral stria arcuate across middle, reaching middle of mesoventrite, continued at sides by lateral metaventral stria; lateral metaventral stria extending from mesometaventral suture to near middle of metacoxa; metaventrite of male not depressed; 1^st^ abdominal ventrite with complete inner displaced mediad, and partial outer lateral striae along inner margin of metacoxa; protibia with outer margin weakly dentate but strongly spinose, with five or six marginal spines, apical corner with shallow emargination (mediad apical spine); protarsi of male with ventral setae not flattened; mesotibia with rather long, fine marginal spines in apical 1/2, those of metatibia very fine to obsolete. Male: basal piece elongate, slightly > 1/3 length of tegmen; tegmen with sides unevenly subparallel in basal 3/4, abruptly narrowed to hooked apices; medioventral process present, projecting at basal 1/3 of tegmen, divided at middle; median lobe ~1/2 tegmen length, basal apodemes abruptly narrowed.

###### Etymology.

This species name is meant to contrast with the above (*P. incongruens*), given the similar, congruous, punctation of its pronotum and elytra.

###### Distribution.

This species is only known from a fairly small area in Amazonian Colombia, Ecuador and northeastern Peru.

###### Remarks.

This species can be readily identified by its consistently dense ground punctation, and lack of interspersed secondary punctures, at least on the pronotum and elytra, as well as the complete lack of submarginal pronotal stria. Its sexual dimorphism is also unique, with the male ventrites not depressed, but bearing very short scale-like setae. There is a surprising degree of size variation among individuals of this species, including among specimens from single localities. However, we can find no other significant characters in which they differ.

##### 42. Phelister praesignis sp. nov.

Taxon classificationAnimaliaColeopteraHisteridae

F26ECA41-70BB-5F75-AE93-79DB65CDC07A

http://zoobank.org/6973F855-0AB3-4C10-8146-0512A22C1DAA

Figs 25E, F , 26E, F , Map 15

###### Type material.

***Holotype* male**: “**Peru**: Junín, 11 km NE Puerto Ocopa, Los Olivos, 11°3.00'S, 74°15.52'W [-11.05, -74.2587],1200 m, Flight intercept. 25–26.iii.2009, A.K.Tishechkin. AT1075” / “Caterino DNA Voucher, Extraction: MSC-2284, Species: Phelister ~ completus, Extraction Date: i.27.2012” / “Caterino/Tishechkin Exosternini Voucher EXO-00940” (FMNH). ***Paratypes* (3): Peru**: Junín, 11 km NE Puerto Ocopa, Los Olivos (-11.05, -74.2587), 1200, 3/27/09–3/28/09, FIT, A. Tishechkin (AKTC, 1ex.); Junín, ~ 16 km NW Satipo, Rio Venado (-11.1989, -74.7705), 1110 m, 2/19/10–2/20/10, A.V. Petrov, EXO-02418 (AKTC, 1ex.); Junín, ~ 15 km NW Satipo, Rio Venado (-11.1979, -74.77), 1100 m, 2/14/13, window trap, A.V. Petrov (AKTC, 1ex.).

###### Other material.

**Brazil**: Pará, Carajás (Serra Norte) (-6.0667, -50.2), October 1986, FIT (CHND, 2ex.).

###### Diagnostic description.

Length: 1.97–2.25 mm (avg. 2.14 mm); width: 1.73–1.97 mm (avg. 1.88 mm). This species is very similar to the preceding (*P. congruens*) in most characters, differing consistently in the following: body larger, more elongate, darker; dorsal punctation denser, particularly pronotum, where slightly larger secondary punctures are intermingled with dense ground punctation; prescutellar impression smaller, more elongate, ~ 1.5 × scutellum size; anterolateral pronotal corners unusually convex, appearing ‘inflated’; outer subhumeral stria weak and short, at most in apical 1/4; prosternal keel not appreciably narrowed anteriorly; male metaventrite weakly impressed and bearing minute setae; meso- and metatibiae slender, edges straight, with only few weak apical spines; male protarsal spines flattened and expanded. Male: basal piece narrow, elongate, slightly > 1/3 length of tegmen; tegmen narrow with sides weakly widened to middle, then abruptly narrowed to weakly hooked apices; medioventral process present, projecting at basal fourth of tegmen, divided at middle; median lobe ~ 1/3 tegmen length, basal apodemes abruptly narrowed

###### Etymology.

The name *praesignis* refers to the ‘outstanding’ appearance of this unusual species.

###### Distribution.

This species is known from two rather widely separated localities, in Amazonian Peru as well as southern Pará (Brazil).

###### Remarks.

This species is quite similar to the preceding two (*P. incongruens* and *P. congruens*), with strong dorsal punctation especially on the pronotum. These also share the distinctive non-spinose metatibia. *Phelister praesignis* is narrower in overall body form than these, and has the secondary pronotal punctation denser, though the individual secondary punctures are not as large as those of *P. incongruens*. In *P. congruens*, the pronotal punctation, while dense, is entirely composed of finer ground punctures. The slightly swollen anterior pronotal corners of *P. praesignis* are also unique.

#### *P. umens* subgroup

This subgroup comprises five species, defined principally by the ‘scalloped’ protibia (a convergence with that of *P. geminus* and *P. serratus*, we believe) and the apically fringed eighth male sternite.

Genitalic characters:

- Aedeagus rather short, basally subparallel, tapered in apical 1/2
- Medioventral process of tegmen present or not
- T8 basal membrane attachment line coincident with apex of basal emargination
- Accessory sclerites absent
- S8 with broad setal fringe
- S9 gradually expanded in apical 1/2
- T10 divided

External characters:

- Scalloped protibia
- Lateral metaventral stria parallel to postmesocoxal stria on side of metaventrite
- Prosternal keel striae weak to obsolete
- Simple, abbreviated 4 th elytral stria, with no basal arch

##### 43. Phelister umens sp. nov.

Taxon classificationAnimaliaColeopteraHisteridae

ED34CB81-B9B7-5CD6-8FEE-EB34F59C443F

http://zoobank.org/36031FCA-64CE-49F5-B1EA-BE68F8E7E0D1

**Figure 27.**
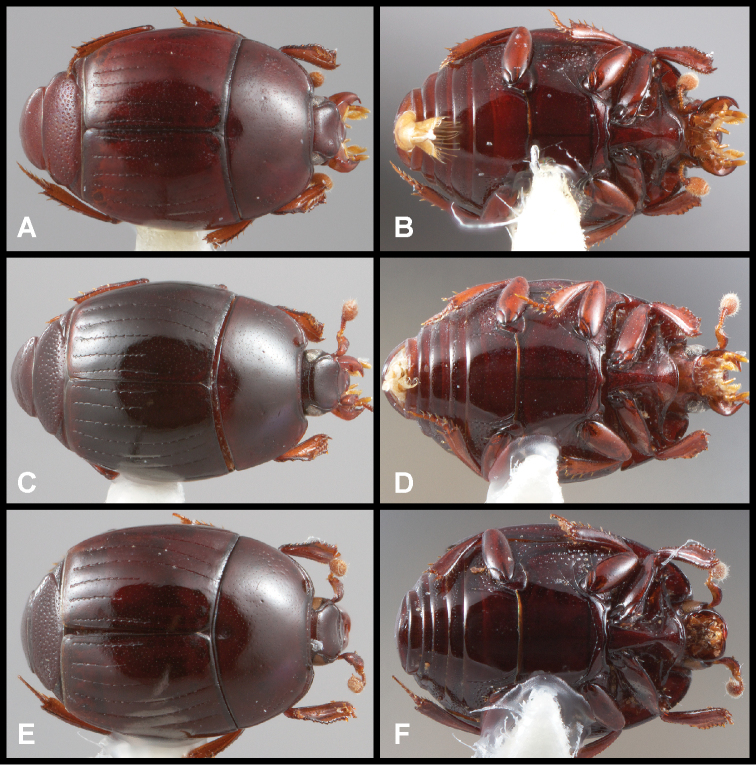
**A, B***Phelister umens*: **A** dorsal habitus **B** ventral habitus **C, D***P. almeidae*: **C** dorsal habitus **D** ventral habitus **E, F***P. chicomendesi*: **E** dorsal habitus **F** ventral habitus.

**Figure 28.**
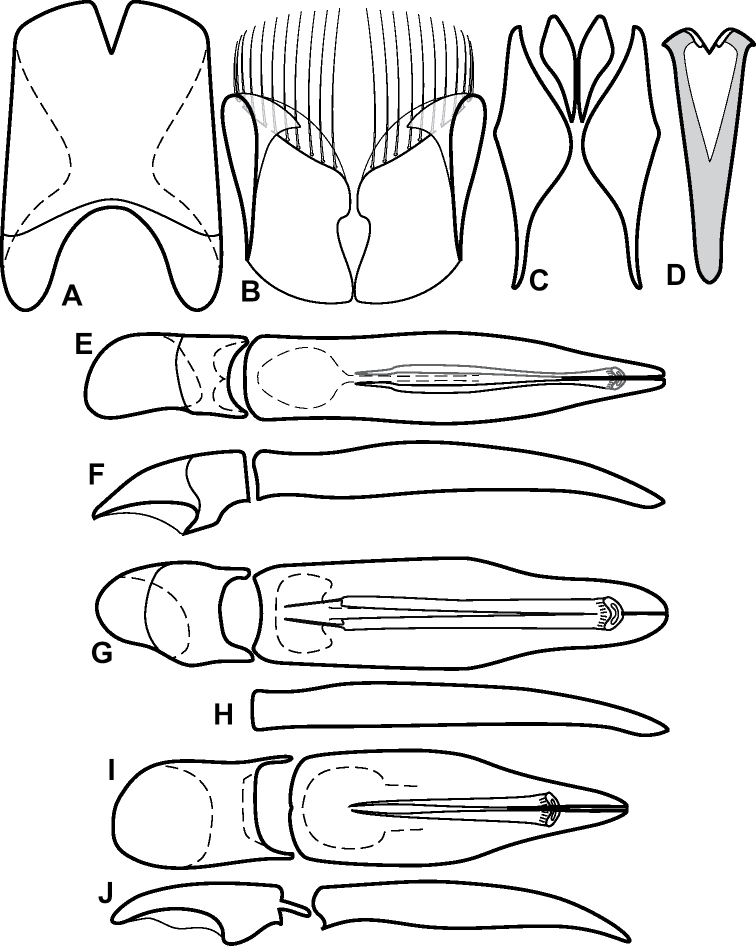
Male genitalia **A–F***Phelister umens*: **A** tergite 8 **B** sternite 8 **C** tergites 9 & 10 **D** sternite 9 **E** aedeagus, dorsal **F** aedeagus, lateral **G, H***P. almeidae*: **G** aedeagus, dorsal **H** tegmen, lateral **I, J***P. chicomendesi*: **I** aedeagus, dorsal **J** tegmen, lateral.

Figs 27A, B , 28A–F , Map 15

###### Type material.

***Holotype* male: “Brazil**: Am[azonas]. Reserva Ducke, 26 km NE, Manaus [-3, -59.94] Barbosa M.G.V.” / “Plot B, FIT1, Feb. 1995” / “Caterino/Tishechkin Exosternini Voucher EXO-00386 (NHMUK). ***Paratypes* (6): Brazil**: Amazonas, Reserva Ducke, 26 km NE, Manaus (-3, -59.94), February 1995, FIT, Barbosa M.G.V., EXO-00386 (NHMUK, 1ex.); Amazonas, Reserva Ducke, 26 km NE, Manaus (-3, -59.94), December 1995, Leaf litter, Barbosa M.G.V., EXO-03286 (NHMUK, 1ex.); Amazonas, Manaus (-3, -59.94), August 1935, G.V. Vrendenburg, EXO-03287 (NHMUK, 1ex.); Amazonas, Presidente Figueiredo, 2.017°S, 59.717°W, 17.ix.2009, FIT, FWT Leivas (DZUP, 2ex.); Amazonas, Fazenda Esteio [-2.38, -59.88], Rd. ZF3, KM23, 5.VII.1985, human dung bait, Bert Klein (FSCA, 1ex.).

###### Other material.

**Brazil**: Amapá, Rio Calçoene lg. do Tigre (2.4, -51.2), 8/6/61–8/8/61, J. & B. Bechyne, EXO-03288 (CHND, 1ex.); Pará, Tucuruí (-3.75, -49.667), September 1984, FIT, EXO-03289 (CHND, 1ex.); **Colombia**: Vaupes, Parco Nac. Mosiro-Itajura (Caparu), Centro Ambiental (-1.0667, -69.5167), 60 m, 1/20/03–1/30/03, FIT, D. Arias & M. Sharkey (AKTC, 1ex.); **Ecuador**: Orellana, Est. Biodiv. Tiputini (-0.64, -76.15), 7/30/08, FIT, A.K.Tishechkin (AKTC, 1ex.); Orellana, Est. Cientifica Yasuní (-0.675, -76.4), 7/17/08–7/19/08, FIT, A. Tishechkin (AKTC, 1ex.); Orellana, Est. Cientifica Yasuní, Rio Tiputini (-0.675, -76.4), 7/26/99–8/4/99, FIT, A. Tishechkin, LSAM 0045448 (LSAM, 1ex.); **French Guiana**: Acarouany (5.6, -53.8), 11/6/75, Piège + excr. humain, EXO-03293 (CHND, 1ex.); Acarouany, Rte. de St. Laurent (5.6, -53.8), 12/27/75, EXO-03294 (CHND, 1ex.); Crique Anguille (4.83, -52.5), 11/1/79, Excr. humain, EXO-03296 (CHND, 1ex.); Mont Tabulaire Itoupé (3.0303, -53.1067), 400 m, 3/17/10, FIT, SEAG, EXO-03297 (CHND, 1ex.); piste Acarouany – St. Laurent du Maroni (5.55, -53.9333), 12/27/75, Piège + cad. oiseau (CHND, 2ex.); Route Nac. 2, P.K. 47 (4.6333, -52.3667), 7/11/78, Piège + cad. de serpent, EXO-03295 (CHND, 1ex.); **Peru**: Junín, ~ 16 km NW Satipo, Rio Venado (-11.1989, -74.7705), 1150 m, 3/08/10–3/14/10, A.V. Petrov (AKTC, 1ex.); Junín, ~ 15 km NW Satipo, Rio Venado at 11°11'40"S, 74°46'8"W, 1195 m, window trap, 27.i.2019, A. V. Petrov (AKTC, 1ex.); Junín, ~ 15 km NW Satipo, nr. Rio Venado at 11°11.1–8'S, 74°46.0–2'W, 1100–1400 m, 4/20/19–5/18/19, A. V. Sokolov (AVSC, 1ex.); Cusco, La Convencion, Echarate, CN Timpia (-12.0621, -72.8516), 454 m, 22.x.2009, M.Alvarado, E.Rázuri (MUSM, 1ex.). **Suriname**: Pará, nr. Overbridge River Resort (5.53, -55.0583), 2/10/10–2/18/10, FIT, P. Skelley, C. Gillett (FSCA, 3ex.); Pará, nr. Overbridge at 5.5195, -55.0695, 2/10/10–2/14/10, FIT, W.B. Warner (AKTC & WBWC, 11ex.); Brokopondo, Ston Eiland Eco Resort nr. Brownsberg (4.9833, -55.1333), 2/10/10–2/13/10, FIT, C. Gillet, P. Skelley, W. Warner, EXO-03334 (FSCA, 1ex.); Sipaliwini, CI-RAP Surv. Camp 1: on Kutari River (-2.1753, -56.7874), 228 m, 8/19/10–8/24/10, FIT, T. Larsen & A.E.Z. Short (SEMC, 1ex.); Sipaliwini, CI-RAP Surv. Camp 1: upper Palumeu River (2.4770°N, 55.6294°W), 275 m, FIT, 3/10/12–3/16/12, A.E.Z. Short (AKTC & SEMC, 6ex.); Sipaliwini, CI-RAP Surv. Camp 4: on lower Kasikasima River (2.9773, -55.3850), 200 m, 3/20/12–3/25/12, FIT, T. Larsen (SEMC, 1ex.).

###### Diagnostic description.

Length: 1.58–1.77 mm (avg. 1.69 mm); width: 1.34–1.54 mm (avg. 1.47 mm). Body elongate oval, weakly depressed, rufescent, with fairly conspicuous ground punctation; frons weakly depressed in middle, supraorbital stria abbreviated at sides, frontal stria complete, sinuate through depression; epistoma depressed at middle, sides raised; labrum emarginate at apex, each mandible with very small basal tooth; prescutellar impression oval, ~ 1.5 × as wide as scutellum; pronotal disk with few to ~ 20 larger secondary punctures near sides; marginal pronotal stria complete along sides and front; lateral submarginal stria usually abbreviated from base, obsolete in basal 1/4, continuing around anterior angle, ending freely near anterior marginal stria; median pronotal gland openings annulate, ~ 2/3 behind anterior pronotal margin; elytron with single, complete epipleural stria; outer and inner subhumeral striae absent; dorsal stria one abbreviated apically, present in basal 1/2 to 2/3, stria two nearly complete, may be abbreviated from apex, stria three present in apical 1/3 and represented by very fine scratch-like stria in basal 1/3, striae four and five both present in apical 1/3, sutural stria in apical 1/2 to 2/3; propygidium with dense secondary punctures, sparser posterad; pygidial secondary punctures much smaller and sparser, finer toward apex; prosternal keel emarginate at base, striae weakly impressed, may be interrupted by conspicuous gland openings between coxae, free anteriorly; prosternal keel-lobe junction narrowed, lobe rounded, with complete marginal stria; mesoventrite wide and short, produced anteriorly at middle, with fine marginal stria continued at side by short postmesocoxal stria which ends behind mesocoxa; meso-metaventral stria angulate at middle, reaching middle of mesoventrite; lateral metaventral stria short, diverging, ending freely behind mesocoxa; metaventrite impunctate; 1^st^ abdominal ventrite with lateral stria interrupted at gland opening mesad posterior corner of metacoxa; protibia with outer margin sinuate, ‘scalloped’, with marginal spines tiny, inconspicuous, set within close-spaced marginal incisions; mesotibia with outer edge rounded, with rather robust marginal spines; metatibia more slender, with finer marginal spines. Male: S8 with dense, long setal fringe; aedeagus with basal piece ~ 1/3 length of tegmen; tegmen with sides unevenly subparallel in basal 2/3, evenly narrowed to subacute apices, largely straight in lateral view; medioventral process absent; median lobe ~ 3/4 tegmen length, basal apodemes abruptly narrowed.

###### Etymology.

This species name, *umens*, means wet or humid, referring to this species’ Amazonian rainforest habitat.

###### Distribution.

Records of this species extend from Amazonian Ecuador east along the Amazon River to Pará, Brazil, as well as to the northeast in Suriname and French Guiana.

###### Remarks.

The members of this small species group are all quite similar, having more or less ‘scalloped’ protibiae, weak to obsolete prosternal keel striae, the 1^st^ elytral stria tending to be obsolete posteriorly, and the lateral pronotal margin somewhat compressed, or bent down outside the submarginal stria. Among these, *P. umens* is distinguished in external characters by having the lateral submarginal pronotal stria slightly abbreviated posteriorly (in others it may be either more complete, or substantially abbreviated), having the subhumeral striae absent, and in having quite dense propygidial punctation.

##### 44. Phelister almeidae sp. nov.

Taxon classificationAnimaliaColeopteraHisteridae

4DCF1BD0-685F-537A-9717-B67E7EA0F2B9

http://zoobank.org/E493CD5E-6141-435D-84D1-0D713BD6CC37

Figs 27C, D , 28G, H , Map 15

###### Type material.

***Holotype* male**: “**Brazil**: Paraná, Piraquara, Mananciais da Serra, 1000 m, 25°29.77'S, 48°58.90'W [-25.4961, -48.9817], intercep. de võo. Jan 2007 P. Grossi & D. Parizotto” / “Caterino/Tishechkin Exosternini Voucher EXO-00547” (DZUP). ***Paratypes* (17): Brazil**: Paraná, Campina Grande do Sul, Estrada da Mandacaia – 1800 (-25.3, -49.04), 12/26/08, FIT, F.W.T. Leivas (DZUP, CESP, 4ex.); Paraná, Campina Grande do Sul, Estrada da Mandacaia – 1800 (-25.3, -49.04), 1/31/09, FIT, F.W.T. Leivas (DZUP, 3ex.); Paraná, Mpio. Curitiba, nr. Campina Grande do Sul (-25.2965, -49.0381), 12/7/11–12/10/11, FIT, F.W.T. Leivas (MSCC, 2ex.); Paraná, Piraquara (-25.4961, -48.9817), 09/3/07–09/10/07, P. Grossi (DZUP, 2ex.); Paraná, Piraquara, Mananciais da Serra (-25.4961, -48.9817), 1000 m, 10/17/07–10/31/07, FIT, P. Grossi & D. Parizotto (CHND, 2ex.); Paraná, Piraquara, Mananciais da Serra (-25.4961, -48.9817), 1000 m, 11/1/07–11/8/07, FIT, P. Grossi & D. Parizotto, EXO-03299 (CHND, 1ex.); Paraná, Piraquara, Mananciais da Serra (-25.4961, -48.9817), 1000 m, 11/8/07–11/23/07, FIT, P. Grossi & D. Parizotto, EXO-03300 (CHND, 1ex.); Paraná, Piraquara, Mananciais da Serra (-25.4961, -48.9817), 1000 m, January 2007, FIT, P. Grossi & D. Parizotto (CHND, 2ex.).

###### Other material.

**Brazil**: Paraná [sic] Tucuruí, Sept. 1984, FIT (CHND 1ex.). This specimen is labelled as written. However, Tucuruí is in Pará state. Whether this specimen is in fact from this disjunct locality seems unlikely, and we suspect it is from some other locality in Paraná.

###### Diagnostic description.

Length: 1.54–1.81 mm (avg. 1.68 mm); width: 1.26–1.50 mm (avg. 1.37 mm). Body elongate oval, weakly depressed, dark rufescent, with fairly conspicuous ground punctation; frons broad, distinctly widened anteriorly, weakly depressed in middle, supraorbital stria complete, meeting frontal stria at sides, frontal stria complete, sinuate through depression; epistoma depressed at middle, sides raised; labrum sinuate, not emarginate, at apex, each mandible with very small basal tooth; prescutellar impression oval, barely larger than scutellum; pronotal disk with few secondary punctures near sides; marginal pronotal stria complete along sides and front, occasionally joining end of submarginal behind eye, thus interrupted; lateral submarginal stria obsolete in basal 1/2, continuing around anterior angle, ending freely near anterior marginal stria or joining it; median pronotal gland openings annulate, slightly > 1/2 pronotal length behind anterior margin; elytron with single, complete epipleural stria; outer subhumeral stria present in apical 1/2, inner absent; dorsal stria one weakened to obsolete in apical 1/2, striae two and three complete, striae four and five both present in apical 1/2, sutural stria present in apical 2/3; propygidium with secondary punctures separated by about their diameters at base, sparser posterad; pygidial secondary punctures smaller and sparser; prosternal keel shallowly emarginate at base, striae separated at base or weakly joined along basal margin, united anteriorly; prosternal keel-lobe junction slightly narrowed, lobe short, rounded, with complete marginal stria; mesoventrite wide and short, produced anteriorly at middle, with fine marginal stria continued at side by short postmesocoxal stria which ends behind mesocoxa; meso-metaventral stria angulate at middle, reaching middle of mesoventrite; lateral metaventral stria diverging, ending freely at middle of lateral portion of metaventrite; metaventrite impunctate; 1^st^ abdominal ventrite with lateral stria complete or interrupted at gland opening mesad posterior corner of metacoxa; protibia with outer margin sinuate, ‘scalloped’, mainly in apical 1/2; meso- and metatibiae with fine marginal spines mainly in apical halves. Male: S8 with setal fringe; aedeagus with basal piece ~ 1/3 length of tegmen; tegmen with sides subparallel in basal 2/3, abruptly narrowed to bluntly rounded apices, largely straight in lateral view; medioventral process absent; median lobe ~ 5/6 tegmen length, basal apodemes thick over most of their lengths abruptly narrowed at extreme bases.

###### Etymology.

We name this species in honor of Dr. Lúcia Massutti de Almeida, of the Universidade Federal do Paraná, in recognition of her mentorship of several outstanding histeridologists, and in gratitude for her hospitality to the authors during a 2011 visit.

###### Distribution.

This species is only known from a few localities, all within Paraná (Brazil).

###### Remarks.

This species is very similar to the preceding, but differs in a number of respects, having the prescutellar impression elongate oval and only slightly larger than the scutellum, the pronotal gland openings more anterior, a more strongly abbreviated lateral submarginal pronotal stria, an outer subhumeral present apically, and having the 3^rd^ dorsal elytral stria complete. Ventrally the prosternal keel striae are also more distinctly impressed in this species.

##### 45. Phelister chicomendesi sp. nov.

Taxon classificationAnimaliaColeopteraHisteridae

C1BD9C5E-DBA4-5C04-9F0A-B46F4548447E

http://zoobank.org/442A0CF6-258B-49A5-AD5D-9B4DF7D155D1

**Map 16.**
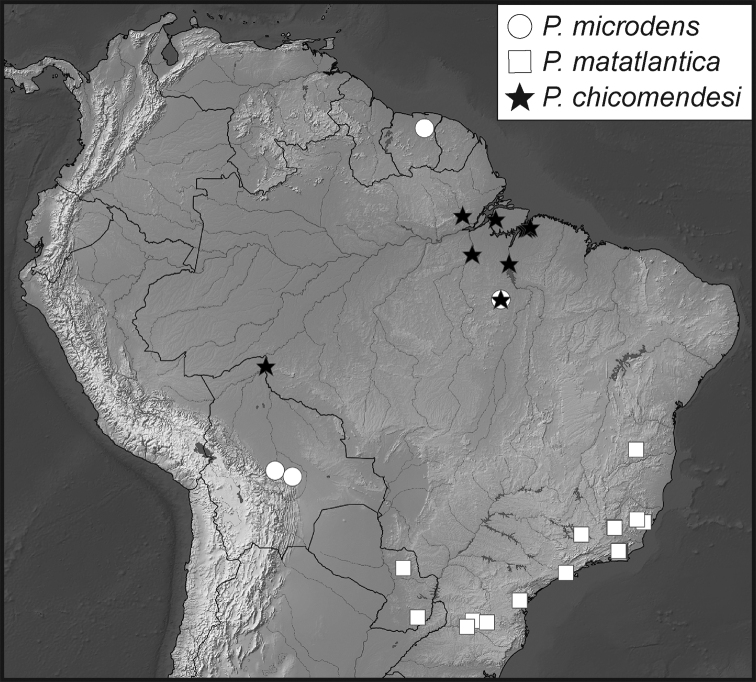
Collecting records for *Phelister chicomendesi* (stars), *P. microdens* (white circles), and *P. matatlantica* (squares).

Figs 27E, F , 28I, J , Map 16

###### Type material.

***Holotype* male**: “**Brazil**: Pará, Carajás (Serra Norte) 6°04'S, 50°12'W [-6.0667, -50.2], Piège d’interception. Iii.1989” / “Caterino/Tishechkin Exosternini Voucher EXO-00388” (CEMT). ***Paratypes* (34): Brazil**: Marajó-Breves (-0.8833, -50.5333), 11/18/87–12/5/87, FIT, EXO-02414 (CHND, 1ex.); Pará, Altamira – Marabá: km 18 (-3.15, -52.05), May 1986, FIT (CHND, 4ex.); Pará, Altamira – Marabá: km 18 (-3.15, -52.05), May 1985, FIT, EXO-03310 (CHND, 1ex.); Pará, Barcarena (-1.5, -48.6167), 6/13/91–6/25/91, FIT (CHND, 6ex.); Pará, Belém, Utinga (IPEAN) (-1.45, -48.4333), November 1984, FIT (CHND, 4ex.); Pará, Belém, Utinga (IPEAN) (-1.45, -48.4333), October, 1985, FIT, EXO-03308 (CHND, 1ex.); Pará, Carajás, Serra Norte (-6.0667, -50.2), 1/22/84–1/27/84, Ss. Cad. de Sarigue (CHND, 2ex.); Pará, Marajó-Breves (-0.8833, -50.5333), 11/18/87–12/5/87, FIT (CHND, 4ex.); Pará, Mcpio Jari, Pacanari (-0.65, -52.6667), 220 m, 3/21/11–4/2/11, Baited pitfalls, C.J. Marsh, EXO-03313 (CHND, 1ex.); Pará, Tucuruí (-3.75, -49.667), 6/19/86–7/7/86, FIT, EXO-03303 (CHND, 1ex.); Pará, Tucuruí (-3.75, -49.667), 6/19/86–7/7/86, FIT, EXO-03305 (CHND, 1ex.); Pará, Tucuruí (-3.75, -49.667), February 1986, FIT, EXO-03304 (CHND, 1ex.); Pará, Tucuruí (-3.75, -49.667), June 1985, FIT (CHND, 3ex.); Pará, Marituba (-1.4, -48.3), 6/10/61, EXO-03315 (CESP, 1ex.).

###### Other material.

**Bolivia**: Pando, nr. Villa Bella (-10.3667, -65.3667), 120 m, 2/25/96, pitfall human dung, primary forest, F. Guerra, EXO-03314 (USNM, 1ex.); **Suriname**: Sipaliwini, upper Palumeu River, CI-RAP Survey Camp 1, 2.4770°N, 55.6294°W, 275 m, flight intercept trap, 10–16.iii.2012, A. E. Z. Short (SEMC, 1ex.).

###### Diagnostic description.

Length: 1.62–1.73 mm (avg. 1.67 mm); width: 1.38–1.42 mm (avg. 1.41 mm). This species is very similar to the preceding, differing principally in the following characters: lateral submarginal pronotal stria obsolete from apical 2/3 or more, present only around anterior angle; outer subhumeral stria present in apical 1/3 to 1/2; 3^rd^ dorsal stria nearly or fully complete (not abbreviated or scratch-like at base); propygidial punctures slightly smaller, but still dense; pygidial punctures more numerous; prosternal keel striae very fine, often nearly or fully obsolete. Male: S8 with dense, long setal fringe; aedeagus with basal piece broad almost 1/2 length of tegmen; tegmen short, with sides unevenly subparallel in basal 1/2, evenly narrowed to subacute apices, largely straight in lateral view, slightly ventrally curved at apex; medioventral process absent; median lobe ~ 2/3 tegmen length, basal apodemes evenly narrowed.

###### Etymology.

We name this species to honor Francisco Alves (‘Chico’) Mendes Filho, a Brazilian labor and environmental activist who was assassinated in 1988.

###### Distribution.

Most records of this species come from Pará, Brazil, but it is also known form northeastern Bolivia.

###### Remarks.

Among the species in this group, the very short lateral submarginal pronotal stria, the apically present outer subhumeral stria, the complete 3^rd^ dorsal stria, and the very weak to obsolete prosternal keel striae will distinguish *P. chicomendesi*. One very disjunct specimen from Bolivia is similar in all these respects, although its protibiae are more quadrate and have more and denser marginal scalloping.

##### 46. Phelister microdens sp. nov.

Taxon classificationAnimaliaColeopteraHisteridae

4D86CA46-701B-53C7-8826-52E9F01A5693

http://zoobank.org/D377C817-A6B3-41BA-8F76-75C14044274A

**Figure 29.**
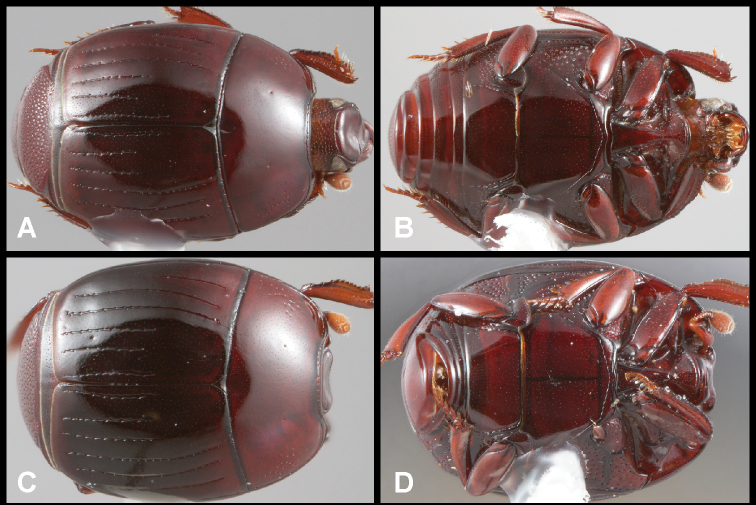
**A, B***Phelister microdens*: **A** dorsal habitus **B** ventral habitus **C, D***P. matatlantica*: **C** dorsal habitus **D** ventral habitus.

**Figure 30.**
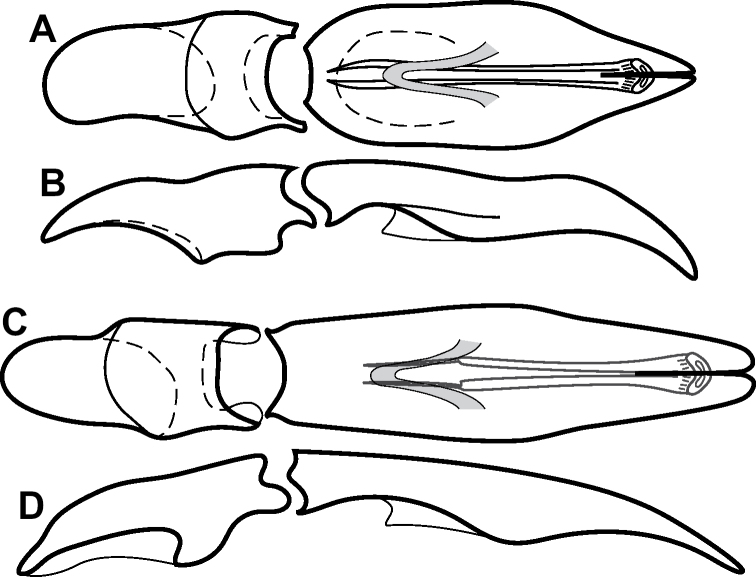
Male genitalia **A, B***Phelister microdens*: **A** aedeagus, dorsal **B** aedeagus, lateral **C, D***P. matatlantica*: **C** aedeagus, dorsal **D** aedeagus, lateral.

Figs 29A, B , 30A, B , Map 16

###### Type material.

***Holotype* male**: “**Bolivia**: Cochabamba, 67.5 km E Villa Tunari, Est. Biol. Valle Sajta, Univ. San Simon 300 m, 17°06'19"S, 64°46'57"W [-17.1053, -64.7825] 9–13.II.1999, F. Génier, lowland rain for., ex. carr. tp. 3, 99–075” / “Caterino/Tishechkin Exosternini Voucher EXO-00387” (CMNC). ***Paratypes* (9): Bolivia**: Cochabamba, Est. Biol. Valle Sajta, 67.5 km E Villa Tunari (-17.1053, -64.7825), 300 m, 2/9/99–2/13/99, carrion trap, lowland rain for., F. Génier, EXO-00387 (CMNC, 1ex.); Santa Cruz, 3.7 km SSE Buena Vista, Flora y Fauna Hotel (-17.4987, -63.6521), 400–440 m, 11/3/03–11/9/03, FIT, R. Leschen, EXO-03332 (AKTC, 1ex.); Santa Cruz, 5 km SSE Buena Vista, Flora y Fauna Hotel (-17.4987, -63.6521), 440 m, 12/15/03–12/24/03, FIT, S. & J. Peck, EXO-02416 (CMNC, 1ex.); Santa Cruz, 5 km SSE Buena Vista, Flora y Fauna Hotel (-17.4987, -63.6521), December 2003, FIT, S & J. Peck (AKTC, 2ex.); Santa Cruz, ~ 5 km SSE Buena Vista, Flora y Fauna Hotel, -17.498 -63.652, 12/14/03–12/24/03, FIT, S. & J. Peck (AKTC, 4ex.).

###### Other material.

**Brazil**: Pará, Carajás, Serra Norte (-6.0667, -50.2), January 1984, Piège + excr. humain, EXO-03333 (CHND, 1ex.).

###### Diagnostic description.

Length: 1.50–1.81 mm (avg. 1.65 mm); width: 1.26–1.62 mm (avg. 1.46 mm). This species is externally almost indistinguishable from the preceding, differing principally in the following characters: median pronotal gland openings near the pronotal midpoint (further anterad than *P. chicomendesi*); lateral submarginal pronotal striae slightly longer, present in most of anterior 1/2; elytral striae more distinctly ‘striatopunctate’, the interconnected punctures larger and more prominent; 4^th^ and 5^th^ dorsal striae slightly longer, the 4^th^ present in apical 1/2, the 5^th^ nearly as long; prosternal keel with striae weak but present, united anteriorly. Male: S8 with apical setal fringe; aedeagus short, basal piece slightly > 1/2 length of tegmen; tegmen with sides unevenly subparallel in basal 2/3, narrowed to subacute apices, apex slightly hooked in lateral view; medioventral process very strong, produced at basal fourth; median lobe ~ 3/4 tegmen length, basal apodemes narrow throughout.

###### Etymology.

The name *microdens* refers to the unusual, finely dentate form of the protibia (shared with a few others treated in this paper).

###### Distribution.

This species is known from few specimens that nonetheless cover a broad swath of tropical South America, from Bolivia to Pará, Brazil.

###### Remarks.

This species is very similar to the two above but may be distinguished by the more anterior location of the pronotal gland openings, the weak but present prosternal keel striae, and the strongly punctate elytral striae.

##### 47. Phelister matatlantica sp. nov.

Taxon classificationAnimaliaColeopteraHisteridae

B76E0F6B-0A74-5ED5-9A6F-18EB70B0ABBF

http://zoobank.org/D22D3203-C4EE-4544-A8C1-6979D38A0DA6

Figs 29C, D , 30C, D , Map 16

###### Type material.

***Holotype* male**: “**Brazil**: São Paulo, 50 km SE Mogi das Cruzes, Serra do Mar, Est. Biol. Boracéia, 800–900m [-23.7, -46], 28–30.IV.1997, F. Génier & S. Ide, ex. feces trap, cloud forest” / “Caterino/Tishechkin Exosternini Voucher EXO-00384” (CMNC). ***Paratypes* (58): Brazil**: Espírito Santo, Pico Pedra Azul (-20.4333, -40.9833), 1500 m, January 1969, F. Vaz-de-Mello, EXO-03326 (CHND, 1ex.); Espírito Santo, Venda Nova do Imigrante (-20.2667, -41.4167), December 2000, FIT, F.Z. Vaz-de-Mello, EXO-03327 (AKTC, 1ex.); Minas Gerais, Águas Vermelhas (-15.75, -41.4667), December 1997, FIT, A.M. Bello & F.Z. Vaz-de-Mello (CEMT, 2ex.); Minas Gerais, Águas Vermelhas (-15.75, -41.4667), December 1983, Cad. de serpent (CHND, 2ex.); Minas Gerais, Aguas Vermelhas (-15.75, -41.4667), December 1997, F. Vaz-de-Mello (CHND, 20ex.); Minas Gerais, Lavras (-21.2333, -45), September 2001, FIT, Dry forest, F.Z. Vaz-de-Mello, EXO-03324 (AKTC, 1ex.); Minas Gerais, Viçosa (-20.75, -42.8833), December 1998, FIT, F.Z. Vaz-de-Mello, EXO-03325 (AKTC, 1ex.); Minas Gerais, Viçosa, Mata do Paraiso (-20.805, -42.8556), 750 m, 2/3/00, dg. tps., prim. mesophilic semideciduous Atlantic for., F. Génier (CMNC, 4ex.); Minas Gerais, Parque Estadual do Itacolomi, Trilha do Forno at 20.4290°S, 43.5075°W, 1350 m, flight intercept trap, 8–10.ii.2014, A. K. Tishechkin (AKTC, 4ex.); Paraná, Piraquara Mananciais da Serra (-25.4934, -48.9786), 12/9/11–12/11/11, FIT, F.W.T. Leivas, M.S. Caterino & A.K. Tishechkin, EXO-00929 (MSCC, 1ex.); Rio de Janeiro, 17 km E Nova Friburgo (-22.3844, -42.5583), 750 m, 1/23/00, carrion trap, secondary mount. Atlantic for., F. Génier & S. Ide, EXO-03317 (CMNC, 1ex.); Rio de Janeiro, 17 km E Nova Friburgo (-22.3844, -42.5583), 750 m, 1/29/00, carrion trap, secondary mount. Atlantic for., F. Génier & S. Ide (CMNC, 2ex.); Rio de Janeiro, N. Friburgo, Sitio Bacco (-22.3, -42.5), 4760ft, 3/26/91–4/9/91, FIT, K.P. Bland (NHMUK, 2ex.); Rio de Janeiro, Nova Friburgo (-22.2667, -42.5333), 10/26/09–10/31/09, FIT (CHND, 2ex.); Rio de Janeiro, Nova Friburgo, 17 km S (-22.3845, -42.5583), 750 m, 1/23/00, carrion trap, secondary montane Atlantic forest, F. Génier & S. Ide, SM0808780 (SEMC, 1ex.); Rio de Janeiro, Nova Friburgo, Macaé de Cima (-22.2667, -42.5333), 1500 m, February 2000, F. Vaz-de-Mello (CHND, 2ex.); Rio de Janeiro, Macaé de Cima, Nova Friburgo (-22.3816, -42.4819), 1030 m, 10/1/03–10/31/07, FIT (AKTC & CHND, 3ex.); Rio de Janeiro, Nova Friburgo, Sans Souci (-22.3, -42.6), 11/9/09–11/15/09, FIT, E. Grossi (DZUP, CESP, 3ex.); Santa Catarina, Chapecozinho (-26.8, -52), January 1958, litter primeral forest, F. Plaumann, FMNH-INS 0000 069 245 (FMNH, 1ex.); Santa Catarina, Nova Teutonia (-27.1833, -52.3833), 10/7/52, bei *Acromyrmex*, F. Plaumann, FMNH-INS 0000 069 241 (FMNH, 1ex.); Santa Catarina, Nova Teutonia (-27.1833, -52.3833), 5/10/60, Berlese: floor litter, F. Plaumann, FMNH-INS 0000 069 236 (FMNH, 1ex.); Santa Catarina, Rio das Antas (-26.9, -51.1), January 1953, Camargo, EXO-03328 (FMNH, 1ex.); São Paulo, Est. Biol. Boracéia, 50 km SE Mogi das Cruzes, Serra do Mar (-23.7, -46), 800–900 m, 4/28/97–5/30/97, feces trap, cloud forest, F. Génier & S. Ide (CMNC, 2ex.); **Paraguay**: Concepcion, Estancia Cororo (-23.4, -56.5), 11/26/99–11/30/99, Tampa con intestino de vacuno, C. Aguilar, EXO-03330 (CHND, 1ex.); Itapua, Itapua Poty (-26.5833, -55.5667), 2/2/91–2/28/91, C. Aguilar, EXO-03329 (CHND, 1ex.).

###### Diagnostic description.

Length: 2.05–2.25 mm (avg. 2.13 mm); width: 1.85–2.01 mm (avg. 1.95 mm). Body rather round, elongate oval, weakly depressed, rufescent, often faintly bicolored, with the central portion of the elytra darkened; ground punctation rather fine and sparse; frons and epistoma depressed in middle, supraorbital stria complete, meeting frontal at sides, frontal stria complete, well impressed, weakly sinuate through depression; epistoma with sides raised; labrum weakly emarginate; each mandible with very small basal tooth; prescutellar impression oval, barely larger than scutellum; pronotal disk with few secondary punctures near sides; marginal pronotal stria complete along sides and front, strongly crenulate in front; lateral submarginal stria complete; median pronotal gland openings annulate, slightly > 1/2 pronotal length behind anterior margin; elytron with single, complete epipleural stria; outer subhumeral stria present in apical one-third, inner absent; dorsal stria one weakened to obsolete in apical 1/2, stria two and three complete, striae four and five both present in apical 1/2, sutural stria in apical 2/3; propygidium with secondary punctures dense almost throughout, sparse only along posterior margin; pygidial secondary punctures smaller and sparser; prosternal keel shallowly emarginate at base, striae well-impressed joined along basal margin, slightly sinuate, united anteriorly; prosternal keel-lobe junction slightly narrowed, lobe short, rounded, with complete marginal stria; mesoventrite wide and short, produced anteriorly at middle, with fine marginal stria often interrupted at middle, continued at side by short postmesocoxal stria which ends behind mesocoxa; meso-metaventral stria bluntly angulate at middle, reaching anterior third of mesoventrite; lateral metaventral stria directed posterolaterally, nearly reaching middle of metacoxa; metaventrite impunctate; 1^st^ abdominal ventrite with complete inner and abbreviated outer lateral striae; protibia with outer margin sinuate, ‘scalloped’, more weakly so in basal 1/2; meso- and metatibiae with fine marginal spines those of mesotibia more numerous and robust. Male: S8 with apical setal fringe; aedeagus with basal piece nearly 1/2 length of tegmen; tegmen with sides slightly widened to middle, evenly narrowed to bluntly rounded apices, apex slightly curved ventrally in lateral view; medioventral process very strong, produced at basal fourth; median lobe ~ 3/4 tegmen length, basal apodemes abruptly narrowed in basal 1/3.

###### Etymology.

The species name is a contraction of Mata Atlantica, the Brazilian Atlantic Forest globally recognized hotspot on which this species largely depends.

###### Distribution.

This species is known mainly from coastal (Atlantic forest) areas of southeastern Brazil. However, it also extends inland through Santa Catarina and into Paraguay.

###### Remarks.

While similar in most characters to other species in this group, this species is considerably larger than the others. They are also frequently faintly bicolored (redder anterad), have the lateral submarginal stria complete, and have the prosternal keel striae well impressed, united anteriorly.

#### *P. curvipes* subgroup

This small subgroup of just two species share a number of seemingly informative external characters, yet their male genitalia have few distinctive features in common. The monophyly of the subgroup, thus, remains to be more firmly established.

External characters:

- Protibia curved
- Mandibles strong, with very large basal tooth, left basal tooth bifid
- Median pronotal gland openings non-annulate
- Prescutellar impression relatively small
- Prosternal lobe short
- Median portion of frontal stria present, separate from lateral portions

##### 48. Phelister curvipes sp. nov.

Taxon classificationAnimaliaColeopteraHisteridae

B14560C2-230F-54AD-A01A-C6E82CC1DF1C

http://zoobank.org/A46F4BD1-6CF4-41BD-A4BA-2B8279059C90

**Map 17.**
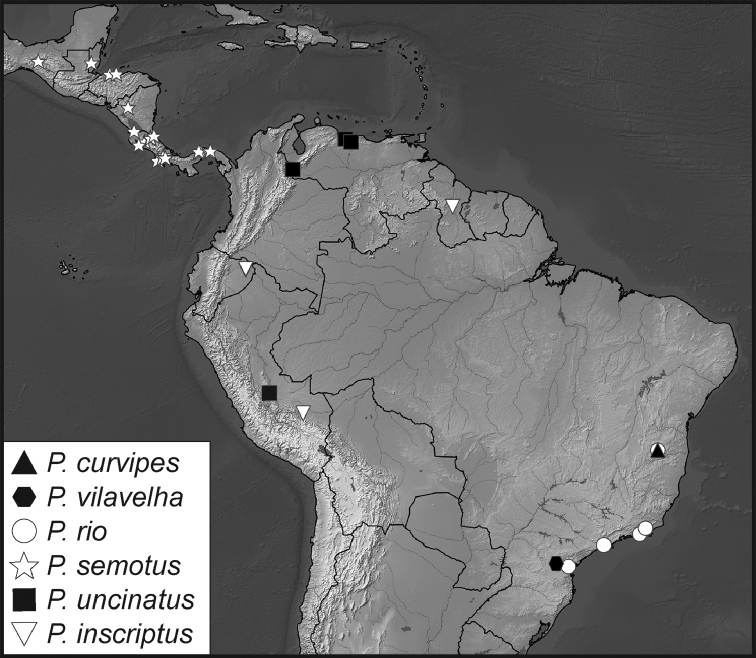
Collecting records for *Phelister curvipes* (upright black triangle), *P. vilavelha* (black hexagon), *P. rio* (white circle), *P. semotus* (stars), *P. uncinatus* (black squares), and *P. inscriptus* (inverted white triangles).

**Figure 31.**
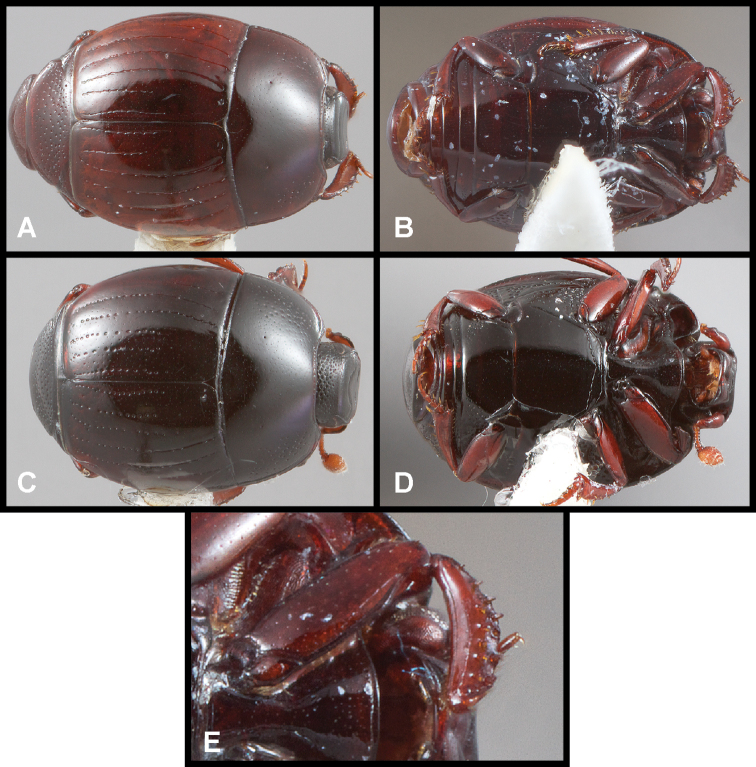
**A, B***Phelister curvipes*: **A** dorsal habitus **B** ventral habitus **C–E***P. vilavelha*: **C** dorsal habitus **D** ventral habitus **E** prothoracic leg, showing curved protibia.

**Figure 32.**
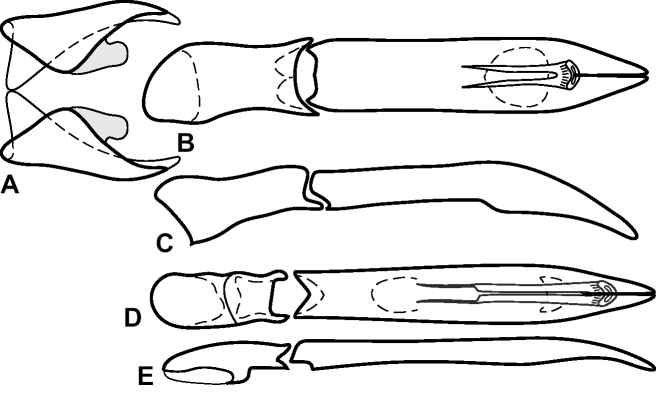
Male genitalia **A–C***Phelister curvipes*: **A** eight tergite, dorsal (T8) **B** aedeagus, dorsal **C** aedeagus, lateral **D, E***P. vilavelha*: **D** aedeagus, dorsal **E** aedeagus, lateral.

Figs 31A, B , 32A–C , Map 17

###### Type material.

***Holotype* male**: “**Brazil**: Minas Gerais, Águas Vermelhas 15°45'S, 41°28'W [-15.75, -41.4667]. Dec 1997, F.Vaz-de-Mello” / “Caterino/Tishechkin Exosternini Voucher EXO-00088” (CEMT).

###### Diagnostic description.

Length: 1.58 mm; width: 1.50 mm. Body elongate oval, convex, faintly bicolored, body dark rufescent with elytra lighter, ground punctation fine and sparse; frons and epistoma weakly depressed; supraorbital stria complete; anterior part of frontal stria comprising two isolated arcs interrupted at middle and detached from sides; labrum weakly emarginate; left mandible with strong basal tooth, right mandible not examined; prescutellar impression an elongate semi-ovals, slightly larger than scutellum; pronotal disk with ~ ten larger lateral secondary punctures; marginal stria complete along sides and front, weakly crenulate anteriorly; lateral submarginal stria complete, close to lateral margin, reaching around anterior corner, nearly meeting anterior marginal stria behind eye; pronotal gland openings 3/4 behind anterior margin, not annulate; epipleuron with single, complete stria; outer subhumeral stria in apical 1/2, inner absent; 1^st^ dorsal stria abbreviated from apex, striae 2–4 complete, 5^th^ in apical 1/2 and with very short, isolated basal arch, sutural stria in apical 2/3; propygidium with small but rather dense secondary punctures in basal 1/3, becoming sparser to apex; pygidial secondary punctures, smaller, obsolete beyond basal 1/2; prosternal keel wide, weakly emarginate at base, prosternal striae united along basal margin, converging to middle then diverging anteriorly, free; prosternal lobe short, rounded, with complete marginal stria; mesoventrite weakly produced, marginal straight across front, well, impressed, not quite meeting base of short postmesocoxal stria at side; mesometaventral stria bluntly angulate at middle, crenulate, continued at sides by lateral metaventral stria nearly to inner 1/3 of metacoxa; metaventrite impunctate; 1^st^ abdominal ventrite impunctate, with single, complete lateral stria; protibia inwardly arcuate along inner margin, outer margin rounded, weakly dentate, with six marginal spines; meso- and metatibiae slender, with fine marginal spines. Male: T8 with secondary lobes along inner apical edges; aedeagus with basal piece nearly 1/2 length of tegmen; tegmen with sides subparallel in basal 3/4, evenly narrowed to subacute apices, tegmen abruptly expanded ventrad at midpoint in lateral view; medioventral process absent; median lobe ~ 1/4 tegmen length, basal apodemes evenly narrowed to tips.

###### Etymology.

We name this species for the distinctive curved protibia (which it shares with the following species).

###### Distribution.

This species is only known from Minas Gerais in eastern Brazil.

###### Remarks.

This species appears closely related only to the following, and they are very similar in a few distinctive external characteristics, listed above. In addition to the surprisingly substantial genitalic differences detailed in the description, this species is typically vaguely bicolored, whereas the following species is uniformly dark. They also differ in the form of the elytral striae, where in *P. curvipes* all the dorsal striae are contiguous, in *P. vilavelha* the inner elytral striae are formed by series of disconnected punctures.

##### 49. Phelister vilavelha sp. nov.

Taxon classificationAnimaliaColeopteraHisteridae

7602654E-3B08-52CA-A9A5-65B8E8D47368

http://zoobank.org/6E44A76D-5A10-4984-B47C-7ED9A9357B2A

Figs 31C–E , 32D–E , Map 17

###### Type material.

***Holotype* male**: “**Brazil**: Paraná, Ponta Grossa, Vila Velha [-25.25, -50.01] 14–16VIII/2009, Grossi & Parizotto (leg.)” / “fezes” / “Caterino/Tishechkin Exosternini Voucher EXO-00751” (DZUP). ***Paratypes* (2)**: same data as type (CESP, CERPE).

###### Diagnostic description.

Length: 1.69–1.81 mm (avg. 1.76 mm); width: 1.54–1.65 mm (avg. 1.62 mm). This species is extremely similar to the preceding (*P. curvipes*) in external morphology (though not in male genitalia), differing principally in the following characters: body not bicolored, more less uniformly piceous frontal ground punctation slightly more conspicuous; right mandible with strong basal tooth (possibly not a difference from the preceding, in which it could not be observed); median pronotal gland openings 2/3 behind anterior pronotal margin; lateral secondary pronotal punctures more numerous and covering most of the outer 1/4 of the pronotal disk; 1^st^ dorsal elytral stria obsolete in the apical 2/3; 2^nd^ through sutural elytral striae distinctly comprising series of disconnected punctures; 4^th^ stria present only in apical 2/3, 5^th^ stria with no basal arch; secondary pygidial punctures sparse but extending nearly to apex; prosternal keel striae obsolete in anterior 1/2; protibia more strongly curved; Male: S8 without apical processes; aedeagus with basal piece ~ 1/3 length of tegmen; tegmen slightly widened to near apex, then evenly narrowed to subacute apices, apex slightly curved ventrally in lateral view; medioventral process absent; median lobe ~ 2/3 tegmen length, basal apodemes abruptly narrowed in basal 1/3.

###### Etymology.

This species is named for the Parque Estadual Vila Velha, the only known locality for this species.

###### Distribution.

This species is only known from the type locality in Paraná, Brazil.

###### Remarks.

The differences between this species and the very similar preceding one are adequately detailed in the preceding remarks section and the description above.

#### *P. rio* subgroup

This subgroup of four rather small, broadly rounded species shares non-annulate median pronotal gland openings with the preceding group.

External characters:

- Median pronotal gland openings non-annulate
- Dorsum largely impunctate
- Prescutellar impression small
- 1 st dorsal elytral stria weak to abbreviated apically
- Elytral striae 3–5 interrupted or abbreviated from base
- Antennal club rather small, round
- Frons weakly impressed, with complete frontal stria
- Mesometaventral stria broadly arched forward
- Lateral metaventral stria divergent laterad
- Male genitalia characters:
- Most with medioventral process of the tegmen

##### 50. Phelister rio sp. nov.

Taxon classificationAnimaliaColeopteraHisteridae

12D6BC52-84CA-5931-9E6B-21330600E105

http://zoobank.org/9EBBF7A8-0C62-450B-8C03-9F8BBF16C48A

**Figure 33.**
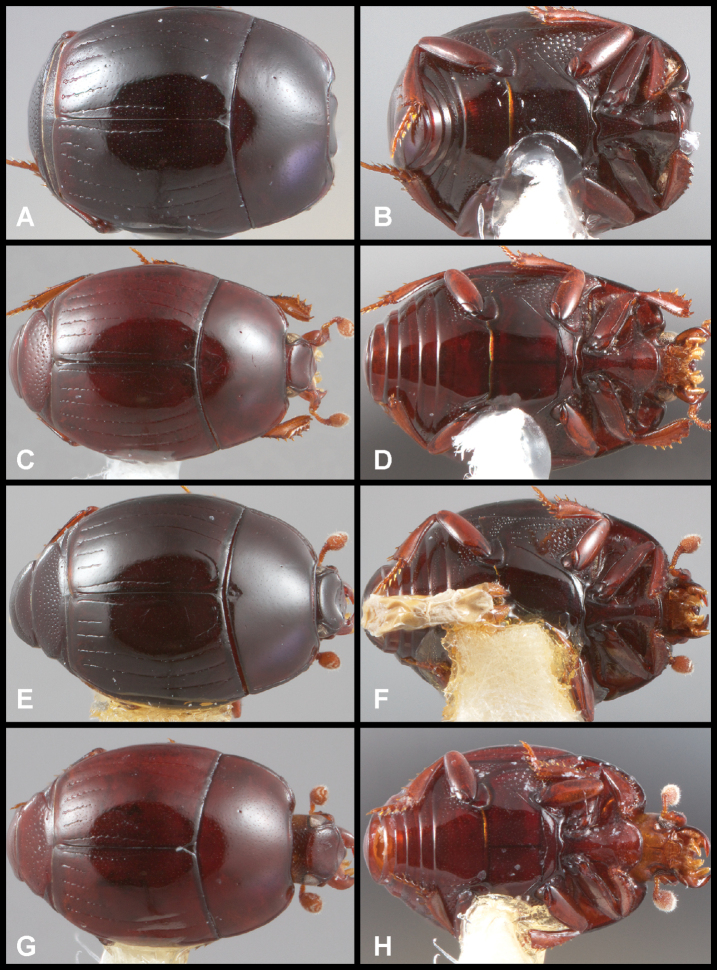
**A, B***Phelister rio*: **A** dorsal habitus **B** ventral habitus **C, D***P. semotus*: **C** dorsal habitus **D** ventral habitus **E, F***P. uncinatus*: **E** dorsal habitus **F** ventral habitus **G, H***P. inscriptus*: **G** dorsal habitus **H** ventral habitus.

**Figure 34.**
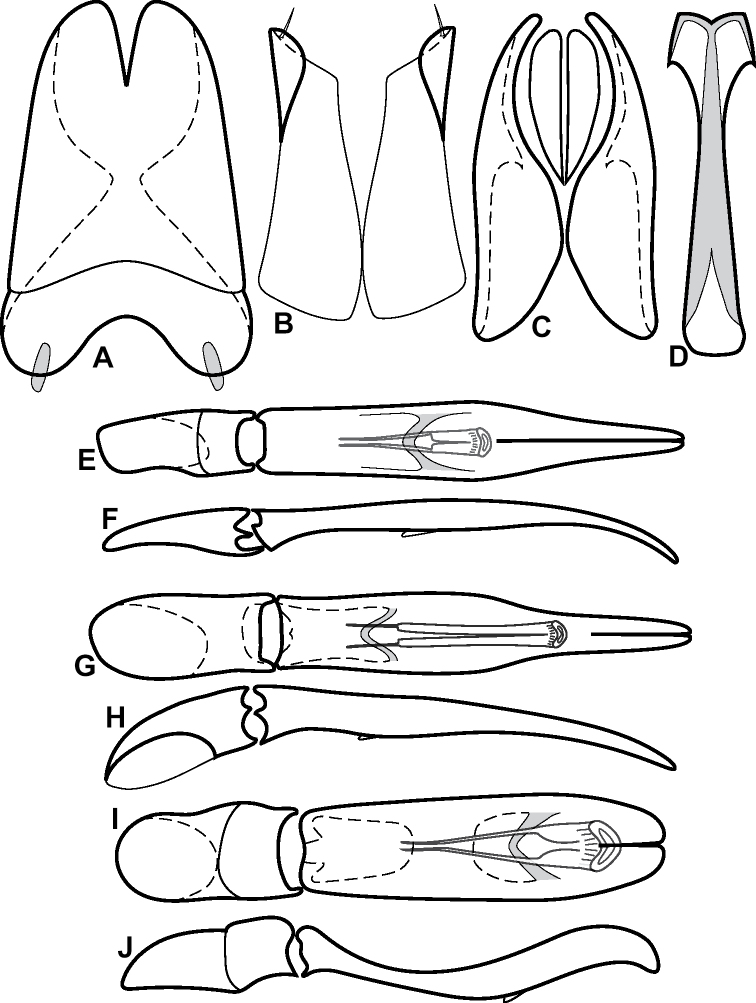
Male genitalia **A–F***Phelister rio*: **A** tergite 8 **B** sternite 8 **C** tergites 9 & 10 **D** sternite 9 **E** aedeagus, dorsal **F** aedeagus, lateral **G, H***P. semotus*: **G** aedeagus, dorsal **H** tegmen, lateral **I, J***P. uncinatus*: **I** aedeagus, dorsal **J** tegmen, lateral.

Figs 33A, B , 34A–F , Map 17

###### Type material.

***Holotype* male**: “**Brazil**: Rio De Janeiro, 17 km E Nova Friburgo 22°23'04"S, 42°33'30"W [-22.3844, -42.5583], 750 m, 29.I.2000, F. Génier & S. Ide, secondary mountain Atlantic for. ex. f.i.t., day 4–9, FG2000-58” / “Caterino/Tishechkin Exosternini Voucher EXO-00358” (CMNC). ***Paratypes* (9): Brazil**: Paraná, Mananciais da Serra (-25.4961, -48.9817), 1000 m, 11/8/07–11/23/07, FIT, P. Grossi & D. Parizotto (CHND, 3ex.); Paraná, Piraquara (-25.4961, -48.9817), 11/3/07–11/10/07, P. Grossi, EXO-03547 (DZUP, 1ex.); Paraná, Piraquara, Mananciais da Serra (-25.4961, -48.9817), 1000 m, 10/17/07–10/31/07, FIT, P. Grossi & D. Parizotto, EXO-03546 (CHND, 1ex.); Rio de Janeiro, Guanabara (-22.8, -43), October, 1963, M. Alvarenga, EXO-03545 (USNM, 1ex.); BRAZIL: Rio de Janeiro, Macaé de Cima, Nova Friburgo (-22.3816, -42.4819), 1030 m, 10/1/03–10/31/07, FIT (CHND, 1ex.); Rio de Janeiro, N. Friburgo, Sitio Bacco (-22.3, -42.5), 4760ft, 3/26/91–4/9/91, FIT, K.P. Bland (NHMUK, 2ex.); São Paulo, Est. Biol. Boracéia, 50 km SE Mogi das Cruzes, Serra do Mar (-23.7, -46), 800–900 m, 4/28/97–4/30/97, feces trap, cloud forest, F. Génier & S. Ide, EXO-03544 (CMNC, 1ex.).

###### Other material.

**Brazil**: Minas Gerais, Águas Vermelhas (-15.75, -41.4667), December 1997, FIT, A.M. Bello & F.Z. Vaz-de-Mello, EXO-03243 (CEMT, 1ex.); Minas Gerais, Águas Vermelhas (-15.75, -41.4667), December 1997, F. Vaz-de-Mello, EXO-00367 (CHND, 1ex.); Minas Gerais, Parque Estadual do Itacolomi, Trilha do Forno at 20.4290°S, 43.5075°W, 1350 m, flight intercept trap, 9–11.ii.2014, A. K. Tishechkin (AKTC, 2ex.); Minas Gerais, Parque Estadual do Rio Doce, Centro de Pesquisas, 19.7637°S, 42.6303°W, 305 m, sifting dry creek bed litter, A. K. Tishechkin (AKTC, 1ex.).

###### Diagnostic description.

Length: 1.42–1.89 mm (avg. 1.67 mm); width: 1.22–1.77 mm (avg. 1.51 mm). Body small, rounded, convex, rufescent to faintly bicolored, the elytra darker than the pronotum, with inconspicuous ground punctation; frons broad, weakly depressed, with complete, rounded frontal stria; supraorbital stria present in middle third; epistoma weakly depressed; labrum emarginate at apex; right mandible with very small basal tooth, left lacking tooth; prescutellar impression very wide, rounded, ~ 5 × as wide and 1.5 × as long as scutellum; disk lacking secondary punctures at sides; median pronotal gland openings small, slightly elongate, non-annulate, ~ 2/3 behind anterior margin; marginal pronotal stria complete around sides and front; lateral submarginal stria complete along lateral margin, barely turned inward at front, well impressed, the marginal bead distinctly convex; elytron with single, complete, crenulate epipleural stria; outer subhumeral stria short, apical, inner absent; dorsal striae very finely impressed, stria one usually slightly abbreviated from apex, striae two and three complete, 4^th^ represented by apical and sometimes basal fragments (no basal arch), 5^th^ present in apical 1/5 only, sutural stria present in apical 3/4; propygidium with secondary punctures concentrated in anterior 1/2, pygidium with only fine, sparse secondary punctures; prosternal keel narrow, emarginate at base, with complete striae separated at base, united anteriorly; prosternal lobe rounded, with complete marginal stria; mesoventrite produced at middle, with fine marginal stria continued at sides by short postmesocoxal; mesometaventral stria strongly arched forward, nearly to marginal mesoventral stria, at sides continued by strongly divergent lateral metaventral stria, which nearly reaches middle of metepipleuron; metaventrite impunctate; 1^st^ abdominal ventrite impunctate, with single, complete lateral stria; protibia quite narrow at base, widened to apex but slender, weakly dentate, with ~ six small marginal spines; male protarsal setae unmodified; meso- and metatibiae slender, with few marginal spines mainly in apical halves. Male: accessory sclerites present, S8 elongate, halves approximate, apex narrowed, somewhat truncate, T10 divided; aedeagus with basal piece ~ one third tegmen length; tegmen slightly widened to midpoint, then tapered to narrowly rounded apices; medioventral process medial, weak; median lobe ~ 1/2 tegmen length, proximal apodemes long, thin.

###### Etymology.

This species is named as an informal reference to the type locality, Rio de Janeiro.

###### Distribution.

This species has been recorded from a handful of localities along coastal southeastern Brazil.

###### Remarks.

The four species within this fairly distinctive group are mostly characterized by minor external differences (despite considerable variation in aedeagal form). *Phelister rio* is distinguished by its wide prescutellar impression in combination with a short, apical outer subhumeral stria, complete 3^rd^ dorsal stria, and lack of basal arch of the 4^th^ or 5^th^ striae. The male genitalia are quite similar to those of *Operclipygus*, a similarity that we presently hypothesize to be a convergence.

Specimens from Minas Gerais are somewhat variable in key characters, some individuals having a smaller, oval prescutellar impression, and more coarsely punctate and posteriorly inturned elytral striae. Others, including a male, however, correspond well to the above diagnosis, and male genitalia match as well.

##### 51. Phelister semotus sp. nov.

Taxon classificationAnimaliaColeopteraHisteridae

5307720D-EBA4-5543-A960-D33B544D9950

http://zoobank.org/5BE27098-01A0-49E5-8894-D51E1B3A3466

Figs 2F , 33C, D , 34G, H , Map 17

###### Type material.

***Holotype* male: “Costa Rica**, Prov. Puntarenas, Golfito, Pque Nal Corcovado, Est Agujas, Cerro Rincón, 745m 13 FEB 2000 A. Azofeifa, L-S 275500 522000 [8.53, -83.41], #56525” / “INB0003078905 INBIOCRI COSTA RICA” (INBIO). ***Paratypes* (145): Belize**: Cayo, Las Cuevas Res. Sta. (16.73, -88.98), May 1996, EXO-03536 (NHMUK, 1ex.); **Costa Rica**: Alajuela, Sector San Ramon (10.1, -84.5), 620 m, 3/12/94–3/13/94, D. Garcia, INBIO CRI001 749708 (INBIO, 1ex.); Guanacaste, Est. Pitilla, 9 km S. de Santa Cecilia (10.99, -85.4), 700 m, May 1995, P. Rios, C. Moraga, INBIO CRI002 427108 (INBIO, 1ex.); Heredia, Est. Biol. La Selva, 3.2 km SE Puerto Viejo (10.4, -84), 100 m, 1/28/92–3/17/92, FIT, W. Bell (SEMC, 12ex.); Heredia, Est. Biol. La Selva (10.4333, -84.0167), 6/19–29/98, FIT, C. Carlton & A. Tishechkin (LSAM, 14ex.); Heredia, Est. Biol. La Selva (10.4333, -84.0167), 50–150 m, 3/5/01–3/8/01, FIT, E. Riley, EXO-03525 (TAMU, 1ex.); Heredia, Est. Magsasay, P.N. Braulio Carrillo (10.4, -84.05), 200 m, January 1991, M. Barrelier, INBIO CRI000 064294 (INBIO, 1ex.); Limon, Sector Cerro Cocori, Fca. de E. Rojas (10.6, -83.72), 150 m, May 1993 (INBIO, 2ex.); Limon, Sector Cerro Cocori, Fca. de E. Rojas (10.6, -83.72), 150 m, January 1993, INBIO CRI001 404993 (INBIO, 1ex.); Puntarenas, Est. Biol. Las Alturas, Coto Brus (8.95, -82.8667), 1550 m, 3/25/02–3/30/02, FIT, A. Cline & A. Tishechkin, EXO-03505 (LSAM, 1ex.); Puntarenas, Est. Biol. Las Alturas, Coto Brus (8.95, -82.8667), 1600 m, 3/30/02–4/7/02, FIT, A. Tishechkin (LSAM, 8ex.); Puntarenas, Est. Biol. Las Cruces, Coto Brus (8.7833, -82.95), 1100 m, 3/22/02–4/1/02, FIT, A. Tishechkin (LSAM, 10ex.); Puntarenas, Est. Biol. Las Cruces, Coto Brus (8.7833, -82.95), 1000 m, 4/1/02–4/8/02, FIT, A. Cline & A. Tishechkin (LSAM, 3ex.); Puntarenas, Est. Biol. Las Cruces, Coto Brus (8.7833, -82.95), 100 m, 4/8/02–4/10/02, FIT, A. Tishechkin, EXO-03517 (AKTC, 1ex.); Puntarenas, Est. Biol. Las Cruces (8.7857, -82.9697), 1330 m, 5/28/04–5/31/04, FIT, J. Ashe, Z. Falin, I. Hinojosa, (SEMC, 6ex.); Puntarenas, Est. Quebrada Bonita, R.B. Carara (9.83, -85), 50 m, March 1994, R. Guzman, INBIO CRI001 752753 (INBIO, 1ex.); Puntarenas, Est. Quebrada Bonita, R.B. Carara (9.83, -85), 100 m, February 1995, R. Guzman, INBIO CRI002 194055 (INBIO, 1ex.); Puntarenas, Pque Nal Corcovado, Est Agujas, Golfito, Cerro Rincon (8.53, -83.41), 745 m, 2/11/00, A. Azofeifa, INB0003120274 (INBIO, 1ex.); Puntarenas, Pque Nal Corcovado, Est Agujas, Golfito, Cerro Rincon (8.53, -83.41), 745 m, 2/12/00, A. Azofeifa, (INBIO, 2ex.); Puntarenas, Pque Nal Corcovado, Est Agujas, Golfito, Cerro Rincon (8.53, -83.41), 745 m, 2/13/00, A. Azofeifa, (INBIO, 3ex.); Puntarenas, San Vito, Las Cruces (8.8, -83), 1200 m, 7/1/82–7/30/82, FIT, B. Gill (AKTC, 3ex.); **Honduras**: Atlantida, Lancetilla Bot. Grd., Tela (15.7667, -87.45), 10 m, 6/23/94, FIT, J. Ashe & R. Brooks, EXO-03537 (SEMC, 1ex.); Atlantida, 15km. W La Ceiba (15.8, -86.9), 6/15/96–6/19/96, FIT, tropical rainforest, R. Lehman (TAMU, 21ex.); Atlantida, 15km. W La Ceiba (15.8, -86.9), 175 m, 7/9/96–7/30/96, Malaise trap, tropical rainforest, R. Lehman (TAMU, 29ex.); **Nicaragua**: Costa Caribe Norte, P.N. Cerro Saslaya (13.77005 -84.98072), 310 m, 5/7/11, mature wet forest, sifted leaf litter, LLAMA collectors (CSCA, 1ex.); Matagalpa, 6 km N Matagalpa, Selva Negra (12.9998, -85.9088), 1250 m, 5/18/02–5/22/02, FIT, forest, S. Peck, EXO-03535 (CMNC, 1ex.); Matagalpa, 6 km N Matagalpa, Selva Negra Hotel, Bavaria Trail (12.9998, -85.9088), 1240 m, 5/18/02–5/21/02, FIT, R. Brooks, Z. Falin, S. Chatzimanolis, SM0558829 (SEMC, 1ex.); **Panama**: Canal Zone, Canal Zonel: Is. Barro Colorado (9.2, -79.8), 4/2/75–4/6/75, Pseudobombax flower fall Snyder-Molino 2, A. Bolton, EXO-03534 (FMNH, 1ex.); Chiriqui, Hartmann’s Finca, 4 km N Sta. Clara (8.75, -82.8), 1500 m, 6/30/82–7/13/82, FIT, B. Gill (AKTC, 3ex.); Colon, P.N. San Lorenzo, Achiote (9.2, -79.9833), 250 m, 1/10/08–1/25/08, FIT, A. Mercado, EXO-03532 (AKTC, 1ex.); Colon, San Lorenzo Forest, STRI crane site (9.2833, -79.9667), 5/11/04–5/25/04, FIT, A. Tishechkin (AKTC, 7ex.); Panama, Chepo-Charti Rd. (9.28, -79.1), 400 m, June 1982, FIT, B. Gill (CNCI, 6ex.); Panama, Nusagandi, Ina Igar (trail) (9.33, -79), 5/18/93–5/21/93, FIT, primary forest, E. Riley, EXO-03526 (TAMU, 1ex.).

###### Other material.

**Mexico**: Chiapas, Ocozocuatla, Laguna Belgica, Sendero Montana (16.8778, -93.454), 1080 m, 7/8/03, oak forest litter, R. Anderson, SM0476858 (SEMC, 1ex.).

###### Diagnostic description.

Length: 1.42–1.73 mm (avg. 1.58 mm); width: 1.14–1.42 mm (avg. 1.30 mm). This species is very similar to the preceding, differing principally in the following characters: supraorbital stria nearly complete; prescutellar impression small, elongate oval, may be acuminate anteriorly, little larger than scutellum; pronotal disk rarely with few secondary punctures at sides; elytral striation rather variable, outer subhumeral stria generally absent, though apical and occasionally basal fragments may be visible, inner absent, stria one usually strongly abbreviated from apex, striae two and three complete, 4^th^ represented by apical and sometimes basal fragments, sometimes complete, 5^th^ present in apical 1/5 and usually as short basal arch, sutural stria present in apical 2/3; mesometaventral stria arched forward to basal 1/2 of mesoventrite, at sides continued by divergent lateral metaventral stria, which ends in middle of lateral portion of metaventrite; mesotibia with rather robust marginal spines; metatibiae slender, with few marginal spines mainly in apical 1/2. Male: aedeagus narrowing abruptly behind midpoint; medioventral process situated ~ 1/4 from base of tegmen; median lobe thin, slightly > 1/2 tegmen length.

###### Etymology.

This species name, *semotus*, refers to its being far or isolated, geographically, from most of the other species in this subgroup.

###### Distribution.

This is one of only a few species in the larger *P. blairi* group that occurs in Central America, ranging from Chiapas, Mexico to Panama.

###### Remarks.

This species has a small oval prescutellar impression, a complete 3^rd^ stria, and a narrow arch at the base of the 5^th^ elytral stria, but lacks an outer subhumeral stria. Otherwise it is very similar to others in this group. One individual (female, excluded from type series) from Chiapas has a strong basal elytral arch from the suture to the base of the 4^th^ stria, but significant differences beyond this aren’t evident.

##### 52. Phelister uncinatus sp. nov.

Taxon classificationAnimaliaColeopteraHisteridae

B02F8118-6C1E-55DD-B89B-71A1BB2283E7

http://zoobank.org/3D3C8ED0-4B8E-4C54-8B4B-B3E2CE36016D

Figs 2D , 33E, F , 34I, J , Map 17

###### Type material.

***Holotype* male: “Venezuela**: Aragua, Rancho Grande Biol.Stn. 10°21'N, 67°41'W [10.3500, -67.6833], 1140 m, 6–9 Mar 1995, Robert W. Brooks#070, ex: flight intercept trap” / “SEMC0903640 KUNHM-ENT” (SEMC); ***Paratypes* (6)**: same data as type (SEMC, 1ex.); same locality but 25–28 Feb 1995 (1ex.); same locality but 1–8 Mar 1995 (1ex.); **Venezuela**: Aragua, Tiara, 50 km SW Caracas (10.1, -67.2), 2000 m, 2/22/71–2/25/71, forest carrion, S. Peck, EXO-02420 (CHSM, 1ex.); Tachira, 20 km NE. San Cristobal (7.8, -72.1), 4000ft, 5/18/74–5/22/74, bait trap, W. Suter (FMNH, 2ex.).

###### Other material.

**Peru**: Junín, 11 km NE Puerto Ocopa, Los Olivos (-11.05, -74.2587), 1200 m, 3/25/09–3/31/09, FIT, A. Tishechkin (AKTC, CHND and MSCC, 12ex.).

###### Diagnostic description.

Length: 1.58–1.73 mm (avg. 1.64 mm); width: 1.18–1.54 mm (avg. 1.37 mm). This species is very similar to the preceding two, differing principally in the following characters: median pronotal gland openings only ~ 1/3 behind anterior pronotal margin; prescutellar impression small, a weakly transverse oval only slightly wider than scutellum; outer subhumeral elytral stria present in apical 1/2; dorsal stria one well impressed in basal 1/3, present as a fine, scratch-like stria nearly to apex; dorsal stria two complete, 3^rd^ stria interrupted, present at base and apex, 4^th^ stria present as short apical fragment and strong basal arch, 5^th^ present in apical 1/4, sutural stria in apical 3/4; propygidial secondary punctures dense in basal 2/3; pygidial punctures uniformly small and sparse; mesometaventral stria strongly arched forward onto mesoventrite, occasionally disrupting marginal mesoventral stria; lateral metaventral stria abbreviated posteriorly, but extending toward outer-third of metacoxa (not toward metepisternum). Male with distinctive undulate aedeagus, tegmen shallowly ‘S’-shaped in lateral view (Fig. 34I); T9 with ventrolateral apodemes small and subapical; S9 with distinct large apical flange on either side of narrow emargination; T10 divided; median lobe of aedeagus with gonopore very large, its upper edge extended and apically acute; proximal apodemes thin, convergent; ventromedial process present near apical one-third, thin and broadly u-shaped.

###### Etymology.

This species name, *uncinatus*, refers to the curved or hooked basal elytral strial fragment.

###### Distribution.

This species is known from a few localities in Venezuela. The specimens from Peru differ slightly in aedeagus characteristics and elytral striation but may simply represent geographic variation.

###### Remarks.

This species is very similar to the preceding, but has its pronotal gland openings much further forward, only ~ 1/3 behind anterior pronotal margin, and has a strong, isolated basal arch between the 4^th^ and sutural striae.

##### 53. Phelister inscriptus sp. nov.

Taxon classificationAnimaliaColeopteraHisteridae

3B0FFB1A-F078-51D3-A903-F69925F5DEDB

http://zoobank.org/52110817-9198-4F7D-93A9-0C12B9F285DC

**Figure 35.**
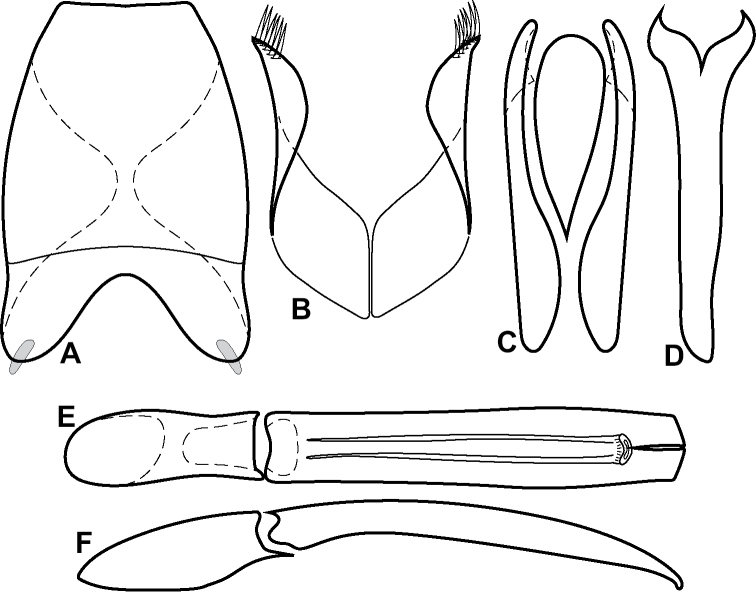
Male genitalia, *Phelister inscriptus***A** tergite 8 **B** sternite 8 **C** tergites 9 & 10 **D** sternite 9 **E** aedeagus, dorsal **F** aedeagus, lateral.

Figs 33G, H , 35A–F , Map 17

###### Type material.

***Holotype* male**: “**Peru**: Madre de Dios, Rio Alto Madre de Dios Pantiacolla Lodge 12°39.3'S, 71°13.9'W [-12.6550, -71.2317], Flight intercept 14–19 Nov 2007, D.Brzoska” / “SEMC0903642 KUNHM-ENT” (SEMC); ***Paratypes* (8)**: same data as type (3ex.); **Peru**: Cusco, Villa Carmen Fld Stn. ~ 1.7 km west cafeteria, 12.89250°S, 71.41917°W, 555 m, 24–26.V.2011, DJ Bennett & E. Razuri, FIT (SEMC, 2ex.); **Ecuador**: Napo, Yuturi Lodge, Rio Napo (-0.5483, -76.0383), 270 m, 3/20/99–3/21/99, FIT, R. Brooks, D. Brzoska, SM0156707 (SEMC, 1ex.); Orellana, Est. Biodiv. Tiputini (-0.6333, -76.0383), 220 m, 9/5/00–9/25/00, D. Inward & K. Jackson, EXO-03335 (NHMUK, 1ex.); Napo, Misahualli Lodge, 1.03138°S, 77.66334°W, vii.6–9.2010, C.M.Riley, FIT (TAMU, 1ex.).

###### Other material.

**French Guiana**: Belvédère de Saül, point de vue (3.6228, -53.2094), 9/2/10, FIT, SEAG (CHND, 1ex.); Régina, Rés. Natur. des Nouragues, Camp Inselberg (4.0833, -52.6833), 9/22/10, FIT, SEAG (CHND, 1ex.); **Guyana**: Region 8, Iwokrama Field Stn., Iwokrama Forest, 1 km W Kurupukari (4.6719, -58.6844), 60 m, 5/30/01–6/2/01, FIT, R. Brooks, Z. Falin, SM0569609 (SEMC, 1ex.).

###### Diagnostic description.

Length: 1.42–1.69 mm (avg. 1.55 mm); width: 1.18–1.50 mm (avg. 1.35 mm). This species is very similar to the preceding three, differing principally in the following characters: median pronotal gland openings 2/3 behind anterior pronotal margin; prescutellar impression semicircular, ~ 2 × as wide as scutellum; anterior margin of pronotum very weakly produced at middle; outer subhumeral elytral stria present in apical 1/3; dorsal stria one nearly complete, scratch-like in apical 1/5 or less, dorsal stria two complete, 3^rd^ stria abbreviated from base, present as serial punctures in apical one-third, but also traceable in basal 1/3 as a fine, scratch-like line, 4^th^ and 5^th^ striae very short, apical, as series of punctures, no basal arches present, sutural stria present in apical 3/4; propygidial secondary punctures dense in basal 2/3; pygidial punctures uniformly small and sparse; mesometaventral stria bluntly angulate at middle, only reaching basal 1/3 of mesoventrite; lateral metaventral stria divergent, extending toward but not reaching metepisternum; 1^st^ abdominal ventrite with small gland opening in extreme corner mediad metacoxa; protibia with outer and apical margins almost evenly, narrowly rounded, outer margin rather densely spinose, bearing ~ ten closely set spines; ventral protarsal setae expanded and flattened, those of male more broadly than females. Male: accessory sclerites present, S8 short, but with long, setose lateral processes; T10 entire; aedeagus with basal piece slightly > 1/3 tegmen length; tegmen weakly widened beyond midpoint, then narrowing slightly to subtruncate apices; medioventral process absent; median lobe long, proximal apodemes thin, undifferentiated.

###### Etymology.

The name of this species refers to the finely ‘inscribed’ basal portion of the 3^rd^ elytral stria.

###### Distribution.

This species is known from three widely separated localities, in Amazonian Ecuador and Peru to Guyana.

###### Remarks.

The primary distinguishing character of this species, compared to the others in this group, is the basally abbreviated 3^rd^ dorsal stria, which is replaced with a very fine, scratch-like basal fragment. The protibial shape, with the rather evenly rounded, densely spinose margins, is also unusual, and the mesometaventral stria in this species is not as strongly arched forward as others in this group. A single female specimen from Guyana has the same tibia and other major characters but differs in having more conspicuous ground punctation, a larger prescutellar impression, and a more complete 3^rd^ dorsal stria. But lacking a male to assess aedeagus shape, we consider it to be an aberrant member of the present species for now.

#### *Incertae sedis*

A few species treated below are either not obviously closely related to any of the subgroups delimited above, or they are not represented by adequate material (males, in one case) to verify critical characters for placement.

##### 54. Phelister incertus sp. nov.

Taxon classificationAnimaliaColeopteraHisteridae

CD750E11-F5F2-51A1-A045-EFF769ED0C1A

http://zoobank.org/724B8D61-F44A-471E-AA83-F10B8A1293AC

**Figure 36.**
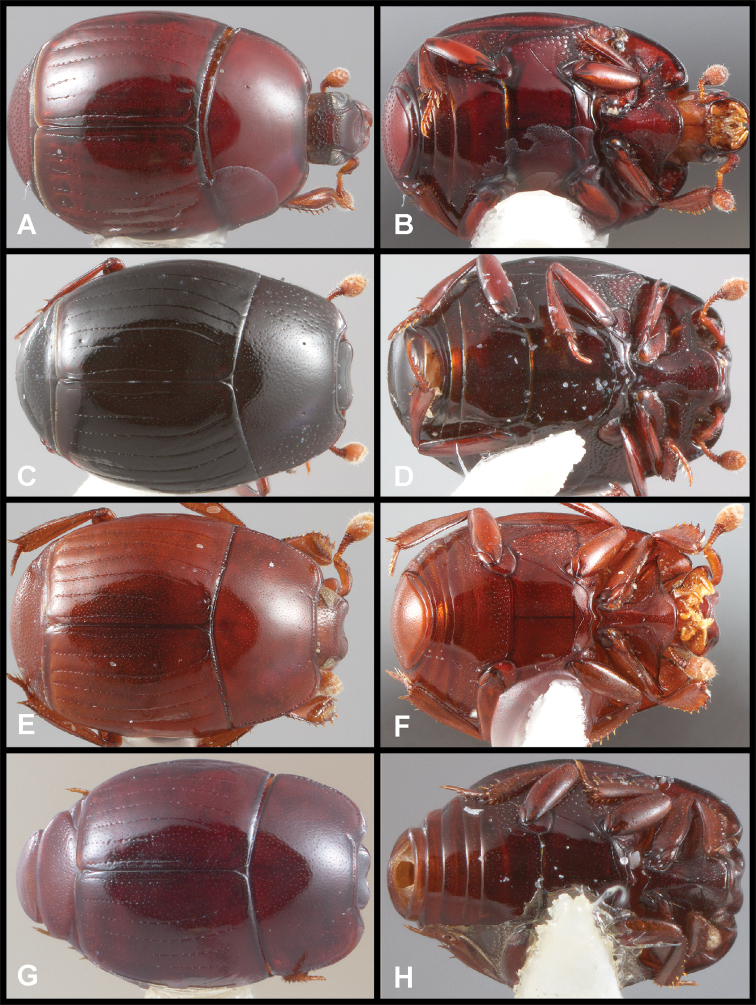
**A, B***Phelister incertus*: **A** dorsal habitus **B** ventral habitus **C, D***P. okeefei*: **C** dorsal habitus **D** ventral habitus **E, F***P. blairoides*: **E** dorsal habitus **F** ventral habitus **G, H***P. pirana*: **G** dorsal habitus **H** ventral habitus.

**Figure 37.**
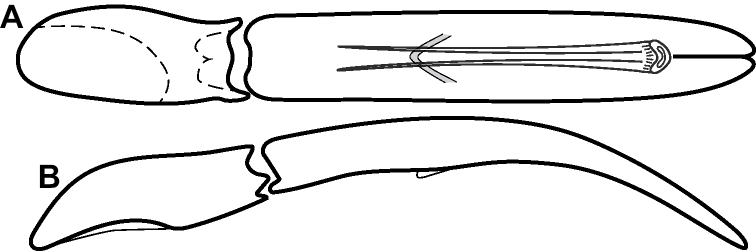
Male genitalia, *Phelister incertus***A** aedeagus, dorsal **B** aedeagus, lateral.

**Map 18.**
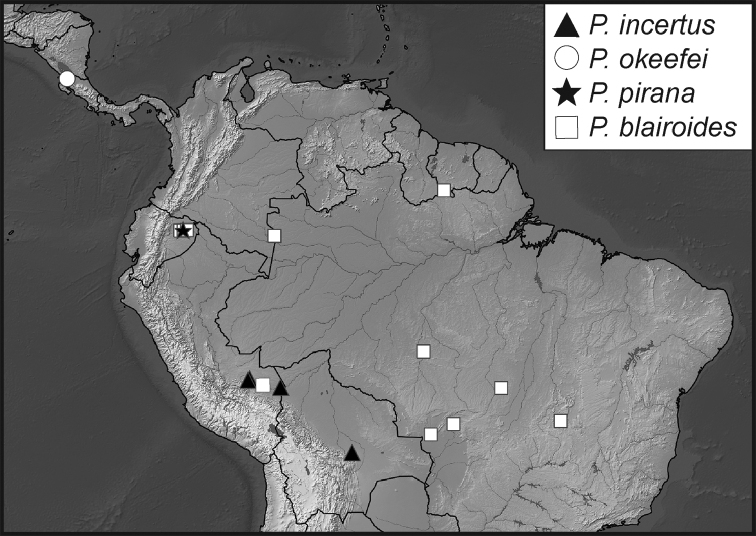
Collecting records for *Phelister incertus* (black triangles), *P. okeefei* (white circle), *P. blairoides* (white squares), and *P. pirana* (star).

Figs 36A, B , 37A, B , Map 18

###### Type material.

***Holotype* male**: “**Bolivia**: Santa Cruz Dep., 3.7 km SSE Buena Vista, Flora y Fauna Hotel 17°29.9'S, 63°33.2'W [-17.4987, -63.6521], 400–440 m, F.I.T. 3–9 Nov 2003, R.Leschen # 052” / “Caterino/Tishechkin Exosternini Voucher EXO-00361” (SEMC); ***Paratypes* (35): Bolivia**: Santa Cruz, ~ 5 km SSE Buena Vista, Flora y Fauna Hotel (-17.4987, -63.6521), 440 m, 12/14/03–12/24/03, FIT, S & J. Peck (AKTC, 1ex.); **Peru**: Madre de Dios, Cocha Cashu Bio. Stn., Manu Nat. Park (-11.8958, -71.4067), 350 m, 10/17/00–10/19/00, FIT, R. Brooks, EXO-03548 (CMNC, 1ex.); Madre de Dios, Cocha Salvador, Manu National Park (-12.0036, -71.5267), 310 m, 10/20/00–10/21/00, FIT, R. Brooks, SM0264371 (SEMC, 1ex.); Madre de Dios, Tambopata, Res. Cuzco Amazonico, 15 km NE Pto. Maldonado (-12.55, -69.05), 200 m, 6/13/89–7/16/89, FIT, R. Leschen (SEMC, 32ex.).

###### Diagnostic description.

Length: 1.42–1.54 mm (avg. 1.51 mm); width: 0.91–1.34 mm (avg. 1.24 mm). Body elongate oval, convex, outline somewhat interrupted at pronotal-elytral junction, with pronotum narrower, rufescent, ground punctation very fine and inconspicuous; frons rather narrow and elongate, along with epistoma weakly depressed; supraorbital stria nearly complete, slightly abbreviated at sides; frontal stria carinate, outwardly rounded, complete; epistoma with sides and front subcarinate, almost appearing striate around edges of depression; labrum weakly emarginate; antennal club short, round, with elongate setose patch on dorsum; prescutellar impression small, elongate oval, narrower than, and about as long as scutellum; pronotal disk with few or no lateral secondary pronotal punctures; marginal pronotal stria complete along sides and front; lateral submarginal pronotal stria complete, arched around anterior corner; pronotal gland openings 3/4 behind anterior margin, widely separated, non-annulate; elytral epipleuron with single, complete stria; outer subhumeral stria in apical 1/3, inner absent; 1^st^ dorsal stria slightly abbreviated from apex, 2^nd^ complete, 3^rd^ complete or interrupted at middle; striae four and five very short, apical, 4^th^ often with short basal fragment, 5^th^ with basal arch connected to base of sutural, sutural stria complete or very narrowly interrupted near base; pro- and pygidium evenly, sparsely covered with conspicuous secondary punctures; prosternal keel weakly emarginate at base, striae weakly divergent anteriorly and posteriorly, free anteriorly; mesoventral process weak, with fine, complete marginal stria; postmesocoxal stria rather long, ending behind outer corner of mesocoxa; mesometaventral stria broadly arched, reaching basal 1/3 of metaventrite, not at all crenulate; lateral metaventral striae divergent toward posterior corner of mesepisternum; protibia with outer margin slightly rounded, weakly dentate, with ~ seven long marginal spines; meso- and metatibiae slender, mesotibia with fairly long marginal spines over most of its length, those of metatibia confined to apical one-third. Male: T10 entire; S8 with few, inconspicuous apical setae; accessory sclerites absent; aedeagus with basal piece slightly > 1/3 tegmen length; tegmen mostly parallel-sided, weakly narrowed to rounded apices; medioventral process present, weak; median lobe 3/4 as long as tegmen, with proximal apodemes undifferentiated.

###### Etymology.

This species name refers to uncertainty with regard to its systematic placement.

###### Distribution.

This species is known from several localities in southeastern Peru and Bolivia.

###### Remarks.

While this species has non-annulate pronotal gland openings, like the preceding two subgroups (*P. curvipes* and *P. rio* subgroups), genitalic characters such as the complete T10 and medioventral aedeagal process make it an awkward fit in either. These characters, in combination with small body size (~ 1 mm), lack of secondary lateral pronotal punctures, and complete, basally hooked sutural stria will distinguish it from other species treated here as well as other *Phelister* spp.

##### 55. Phelister okeefei sp. nov.

Taxon classificationAnimaliaColeopteraHisteridae

9ADDB566-B523-546B-96EE-822C757B7250

http://zoobank.org/175867AE-F265-4248-86A6-70D97B7F21CC

**Figure 38.**
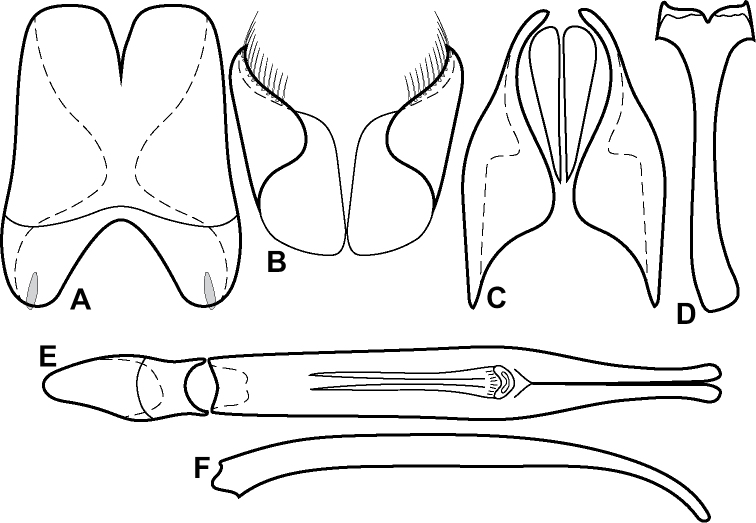
Male genitalia, *Phelister okeefei***A** tergite 8 **B** sternite 8 **C** tergites 9 & 10 **D** sternite 9 **E** aedeagus, dorsal **F** aedeagus, lateral.

Figs 36C, D , 38A–F , Map 18

###### Type material.

***Holotype* male**: “**Costa Rica**: Guanacaste, Guayabal [10.8, -85.2], vii.23.1996, S. O’Keefe” / “Caterino/Tishechkin Exosternini Voucher EXO-00546” (FMNH, 1ex.).

###### Diagnostic description.

Length: 1.85 mm; width: 1.58 mm. Body elongate oval, piceous, pronotum rather narrow, with conspicuous ground punctation; frons and epistoma weakly depressed, frontal stria complete, shallowly recurved at middle; labrum weakly emarginate, apical margin not at all carinate; both mandibles with basal tooth on incisor edge; prescutellar impression forming a small rounded triangle, ~ 2 × the length and width of scutellum; pronotal disk with numerous lateral secondary punctures in outer fourths; marginal pronotal stria complete along sides and front, not crenulate anteriorly; lateral pronotal margins slightly compressed, edges turned down, flattened; lateral submarginal pronotal stria absent; median pronotal gland openings 1/3 behind anterior margin, annulate; epipleuron with single, complete stria; outer subhumeral stria present in apical 1/3, inner absent; dorsal striae 1–4 complete, 4^th^ slightly sinuate at base, 5^th^ stria present in apical 1/3 plus a short detached basal arch, sutural stria present in apical 2/3; propygidium and pygidium each with small, sparse secondary punctures confined to basal halves; base of prosternal keel deeply emarginate, striae divergent, free at base and apex; prosternal lobe rounded, with complete marginal stria; mesoventral process strong, marginal stria complete; postmesocoxal stria short, ending behind mesocoxa; mesometaventral stria broadly arched forward to basal 1/3 of mesoventrite; lateral metaventral stria extended toward middle of metacoxa; metaventrite impunctate; 1^st^ abdominal ventrite impunctate, with single, complete lateral stria; protibia somewhat broad, parallel-sided in apical 2/3, weakly dentate, with ~ six robust marginal spines; meso- and especially metatibiae slender, with few marginal spines. Male T8 with accessory sclerites present; S8 with conspicuous apical fringe; T10 divided; aedeagus thin, basal piece ~ 1/4 tegmen length; tegmen widened slightly to just beyond midpoint, abruptly narrowed, apices narrowly rounded, weakly divergent, curved ventrad; medioventral process absent; median lobe ~ 1/2 tegmen length.

###### Etymology.

We take pleasure in naming this species for the collector of the only known specimen of this species, scydmaenine staphylinid specialist, Dr. Sean O’Keefe (Morehead State University, USA).

###### Distribution.

This species is only known from Guanacaste, Costa Rica.

###### Remarks.

This species is a bit of an outlier among those treated in this paper. It has a relatively large subtriangular prescutellar impression, and annulate pronotal gland openings ~ 1/3 behind the anterior margin. But its frons and epistoma are only very weakly impressed, its labrum is only weakly emarginate, and the left mandible (and maybe the barely visible right one) has a strong basal tooth. Together, these characters will distinguish it from any other Exosternini in Central America.

##### 56. Phelister blairoides sp. nov.

Taxon classificationAnimaliaColeopteraHisteridae

3E28A3D4-DF1C-5C04-9FA0-2D2A38263934

http://zoobank.org/97B442DB-5E46-4D48-BB07-D27202204D43

**Figure 39.**
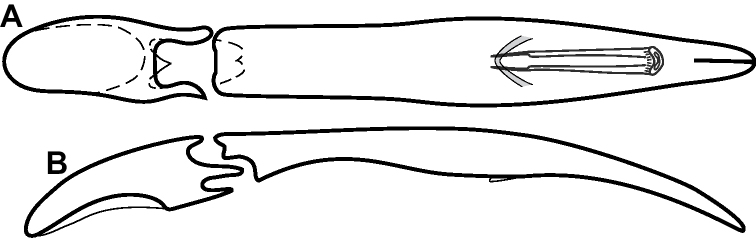
Male genitalia *Phelister blairoides***A** aedeagus, dorsal **B** aedeagus, lateral.

Figs 36E, F , 39A, B , Map 18

###### Type material.

***Holotype* male**: “**Ecuador**: Napo Prov., Estación Cientifica Yasuní 00°40'28"S, 76°50'50"W [-0.6744, -76.6472], IX-5-10-1999, 215 m, Coll. E. G. Riley” / “flight intercept trap, primary forest” / “Caterino/Tishechkin Exosternini Voucher EXO-00356” (TAMU); ***Paratypes* (39)**: same data as type (TAMU, 11ex.); **Ecuador**: Orellana, Est. Biodiv. Tiputini (-0.6376, -76.1499), 6/4/11–6/9/11, FIT, M. Caterino & A. Tishechkin, EXO-00705 (MSCC, 1ex.); Orellana, Est. Cientifica Yasuní, mid. Rio Tiputini (-0.675, -76.4), 7/26/99–7/30/99, FIT, A. Tishechkin, LSAM 0045163 (LSAM, 1ex.); Orellana, Est. Biodiv. Tiputini (-0.64, -76.15), 7/28/08–7/31/08, FIT, A.K.Tishechkin (AKTC, 2ex.); Orellana, Est. Biodiv. Tiputini (-0.64, -76.15), 8/3/08–8/6/08, FIT, LSAM Team (LSAM, 3ex.); Orellana, Est. Cientifica Yasuní (-0.675, -76.4), 7/11/08–7/24/08, FIT, A. Tishechkin (AKTC, CHND & LSAM, 12ex.); **Colombia**: Vaupes, Est. Biol. Caparu, Rio Apoporis (-1.1, -69.5), 9/27/95–12/1/95, FIT, Black-water terrace forest on sandy soils, B. Gill, EXO-03436 (AKTC, 1ex.); Vaupes, Perco. Nac. Mosiro-Itajura (Caparu), Centro Ambiental (-1.0667, -69.5167), 60 m, 1/20/03–1/30/03, FIT, D. Arias & M. Sharkey, EXO-03437 (8ex.).

###### Other material.

**Brazil**: Distrito Federal, Res. Ecol. do IBGE, Brasília, Armad. Janela, área não queimada (-15.0917, -47.8833), 1/20/98, EXO-03430 (CHND, 1ex.); Distrito Federal, Res. Ecol. do IBGE, Brasília, Armad. janela, área queimada (-15.0917, -47.8833), 1/6/97, EXO-03429 (CHND, 1ex.); Distrito Federal, Res. Ecol. do IBGE, Brasília, Armad. janela, área queimada (-15.0917, -47.8833), 1/20/98, EXO-03431 (CHND, 1ex.); Mato Grosso, Cáceres (-16.1, -57.7), 11/17/84, Buzzi, Mieike, Elias, Casagrande, DZUP 272612 (DZUP, 1ex.); Mato Grosso, Mpio. Cotriguaçu, Fazenda São Nicolau, Mata Norte (-9.8192, -58.26), 12/8/10–12/14/10, FIT, F.Z. Vaz-de-Mello (CEMT, 5ex.); Mato Grosso, Mpio. Cotriguaçu, Fazenda São Nicolau, Matinha (-9.8383, -58.2508), October 2009, FIT, F.Z. Vaz-de-Mello, EXO-03434 (CEMT, 1ex.); Mato Grosso, Mpio. Cuiaba Fazenda Mutuca (-15.3145, -55.9703), 3/13/09, FIT, J.R. Rocha, EXO-03433 (CEMT, 1ex.); Mato Grosso, Mpio. Cuiaba Fazenda Mutuca (-15.3145, -55.9703), 12/6/11–12/20/11, FIT, cerrado, M. Caterino & A. Tishechkin, EXO-00862 (MSCC, 1ex.); Mato Grosso, Mpio. Querencia, Fazenda São Luiz (-12.597, -52.3749), February 2009, FIT, R. Andrade, EXO-03432 (CEMT, 1ex.); **French Guiana**: Belvédère de Saül, point de vue (3.6228, -53.2094), 12/20/10, FIT, SEAG (CHND, 1ex.); Régina, Rés. Natur. des Nouragues, Camp Inselberg (4.0833, -52.6833), 9/30/10, FIT, SEAG (CHND, 1ex.); **Peru**: Madre de Dios, CICRA Field Stn., ~ 2 km NW cafeteria res. plot. 12.55236°S, 70.10989°W, 295 m, Flight intrcept. 7–13.vi.2011, Chaboo Team (SEMC 4ex.); **Suriname**: Sipaliwini, CI-RAP Surv. Camp 3: Wehepai SE Kwamala (2.3629, -56.6977), 237 m, 9/3/10–9/7/10, FIT, Larsen & Short, EXO-03438 (SEMC, 1ex.); Sipaliwini, CI-RAP Surv. Camp 4: on lower Kasikasima River (-2.9773, -55.3850), 200 m, 3/20/12–3/25/12, FIT, T. Larsen (AKTC & SEMC, 4ex.).

###### Diagnostic description.

Length: 1.81–2.36 mm (avg. 2.11 mm); width: 1.58–1.97 mm (avg. 1.81 mm). Body elongate, only weakly oval, rufescent, with ground punctation moderately conspicuous; frons and epistoma somewhat narrow, deeply depressed along midline, conspicuously punctate; supraorbital stria present, narrowly separated from sides of frontal; frontal stria narrowly interrupted at middle; epistoma raised at sides; labrum weakly emarginate; each mandible with only very small basal tooth; antennal club elongate with conspicuous setose patch on dorsal surface; prescutellar impression of pronotum narrowly oval, about equal in width to scutellum; pronotal disk with numerous small secondary punctures in lateral thirds, sparser toward anterior corner; median pronotal gland openings distinctly annulate, ~ 1/5 behind anterior margin; lateral marginal pronotal stria complete, broken with inner ends recurved briefly behind eyes; submarginal pronotal stria complete along lateral margin, weakly impressed, crenulate, rounding anterior corner, nearly meeting broken ends of anterior marginal stria; elytron with single, complete epipleural stria; outer subhumeral stria present in basal and apical halves, interrupted at middle, inner absent; dorsal striae 1–4 complete, 4^th^ arched to sutural, 5^th^ present in apical 1/2, sutural stria usually complete; propygidium with uniformly dense, small secondary punctures; those of pygidium sparser and much smaller, barely larger than ground punctures; prosternal keel emarginate at base, striae separate or weakly united along base, enclosing a triangle ~ 3/4 length of keel, with secondary striae along basal 1/2; prosternal lobe rounded, with complete marginal stria; mesoventrite produced, with complete marginal stria continued at sides by postmesocoxal stria, ending freely beyond posterior corner of mesocoxa; mesometaventral stria sinuate, angulate at middle, reaching middle of mesoventrite, continued at sides by lateral metaventral stria to inner 1/3 of metacoxa; middle portion of metaventrite impunctate; 1^st^ abdominal ventrite with secondary punctures along anterior third, with nearly complete lateral stria along inner margin of metacoxa; protibia broad, outer margin with fine, sparse marginal spines. Male: T8 with accessory sclerites present, T10 divided, S8 with a couple apical setae, no fringe; aedeagus with basal piece ~ 1/3 tegmen length; tegmen sides parallel in basal 1/3, slightly widened to midpoint, then tapered to narrowly rounded apex; median lobe short, apical.

###### Etymology.

The name of this species signifies its strong but perhaps convergent similarity to the species *P. blairi* .

###### Distribution.

This widespread species is known from Amazonian Ecuador, Colombia, central Brazil, and southern Suriname.

###### Remarks.

This species is very similar to *P. blairi* in a number of external characters, although genitalic morphology suggest the two are not closely related. Both have the pronotal gland openings quite far forward, at most 1/4 behind the pronotal margin, and typically have the anterior marginal pronotal stria broken and/or recurved in front of these. *Phelister blairoides* can be distinguished by its complete (not obsolete basally) lateral submarginal pronotal stria, and its outer subhumeral elytral stria being present basally as well as apically (though interrupted at middle), whereas *P. blairi* has only the apical portion of the outer subhumeral stria present. It is also considerably larger in overall body size than *P. blairi*. The most salient differences in male genitalia include the fully divided 10^th^ tergite in *P. blairoides* (halves fused basally in *P. blairi*), and the more distal position of the medioventral process in *P. blairoides*.

##### 57. Phelister pirana sp. nov.

Taxon classificationAnimaliaColeopteraHisteridae

5E30D20B-10DB-5F40-88BA-541E7623266A

http://zoobank.org/C6E2CBB4-440A-454A-90D9-7DE85603212E

Fig. 36G, H , Map 18

###### Type material.

***Holotype* (genitalia missing)**: “**Ecuador**: Depto. Orellana, P.N. Yasuní, Via Maxus at Puente Piraña 0°39.5'S, 76°26'W [-0.6583, -76.4333], Flight intercept FIT3a-1. 14–20.vii.2008 A.K.Tishechkin. AT872” / “Caterino/Tishechkin Exosternini Voucher EXO-01299” (CSCA).

###### Diagnostic description.

Length: 1.42 mm; width: 1.18 mm. Body small, elongate oval, slightly flattened, rufescent, with ground punctation quite conspicuous; frons and epistoma somewhat narrow, depressed along midline, conspicuously punctate; frontal stria complete across depression; epistoma raised at sides; labrum emarginate and subcarinate apically; mandibles lacking conspicuous basal teeth; prescutellar impression of pronotum absent, prescutellar region vaguely depressed, but not delimited by stria; pronotal disk with only a few small secondary punctures intermingled with conspicuous ground punctation very close to lateral margins; median pronotal gland openings small, annulate, only ~ 1/8 pronotal length behind anterior margin; marginal pronotal stria complete laterally, broken with inner ends recurved briefly behind eyes; submarginal pronotal stria present along pronotal sides, curved inward around anterior corner, not meeting anterior portion of marginal stria; elytron with single, complete epipleural stria, epipleuron weakly subcarinate above, mainly in posterior 1/2; outer subhumeral stria more or less complete, slightly abbreviated anteriorly, sinuate over lateral elytral gland opening, inner subhumeral stria absent; dorsal striae 1–4 complete, all tending to become fragmented posteriorly, 4^th^ arched to sutural, 5^th^ stria present in apical 1/3, sutural stria present in apical 2/3; propygidium with very sparse secondary punctures intermingled with ground punctation; pygidium with ground punctation only, disk appearing slightly depressed along lateral margins; prosternal keel deeply emarginate at base, strongly narrowed (‘pinched’) anteriorly, striae obsolete; prosternal lobe short, rounded, with complete marginal stria; mesoventrite produced, with complete marginal stria not reaching postmesocoxal stria, which ends freely behind outer corner or mesocoxa; mesometaventral stria angulate at middle, reaching middle of mesoventrite, continued posterad by lateral metaventral stria to middle of metacoxa; middle portion of metaventrite impunctate; 1^st^ abdominal ventrite with few small secondary punctures along anterior margin, with incomplete lateral stria along inner margin of metacoxa; protibia with outer margin weakly dentate, with five or six marginal spines; meso- and metatibiae slightly broadened, mesotibia with rather robust marginal spines, those of metatibia fine and restricted to apical 1/2. Male not known.

###### Etymology.

This species name refers to the name of the bridge, Puente Piraña, near the type locality.

###### Distribution.

This species is known only from the type locality in Orellana, Ecuador.

###### Remarks.

This species is readily distinguished by its obsolete prosternal striae and pinched prosternal keel/lobe junction, its weakly margined elytra, and median pronotal gland openings very far forward. Its relationships may lie within the *blairi* subgroup, based on the position of the pronotal gland openings. However, it is not otherwise very similar to others there, and male genitalia would help assess its placement.

## Supplementary Material

XML Treatment for Phelister blairi

XML Treatment for Phelister erwini

XML Treatment for Phelister fimbriatus

XML Treatment for Phelister stellans

XML Treatment for Phelister sparsus

XML Treatment for Phelister pretiosus

XML Treatment for Phelister trigonisternus

XML Treatment for Phelister globosus

XML Treatment for Phelister serratus

XML Treatment for Phelister geminus

XML Treatment for Phelister parana

XML Treatment for Phelister asperatus

XML Treatment for Phelister uniformis

XML Treatment for Phelister miscellus

XML Treatment for Phelister inbio

XML Treatment for Phelister sculpturatus

XML Treatment for Phelister tunki

XML Treatment for Phelister praedatoris

XML Treatment for Phelister ifficus

XML Treatment for Phelister genieri

XML Treatment for Phelister marginatus

XML Treatment for Phelister vazdemelloi

XML Treatment for Phelister dilatatus

XML Treatment for Phelister spectabilis

XML Treatment for Phelister pervagatus

XML Treatment for Phelister morbidus

XML Treatment for Phelister annulatus

XML Treatment for Phelister sphaericus

XML Treatment for Phelister geijskesi

XML Treatment for Phelister fraternus

XML Treatment for Phelister conjunctus

XML Treatment for Phelister chabooae

XML Treatment for Phelister striatinotum

XML Treatment for Phelister notandus

XML Treatment for Phelister amazoniae

XML Treatment for Phelister arcuatus

XML Treatment for Phelister gregarius

XML Treatment for Phelister praecisus

XML Treatment for Phelister rudis

XML Treatment for Phelister incongruens

XML Treatment for Phelister congruens

XML Treatment for Phelister praesignis

XML Treatment for Phelister umens

XML Treatment for Phelister almeidae

XML Treatment for Phelister chicomendesi

XML Treatment for Phelister microdens

XML Treatment for Phelister matatlantica

XML Treatment for Phelister curvipes

XML Treatment for Phelister vilavelha

XML Treatment for Phelister rio

XML Treatment for Phelister semotus

XML Treatment for Phelister uncinatus

XML Treatment for Phelister inscriptus

XML Treatment for Phelister incertus

XML Treatment for Phelister okeefei

XML Treatment for Phelister blairoides

XML Treatment for Phelister pirana

## Supplementary materials

Supplementary material 1 Table S1. Taxa selected for the phylogenetic reanalysis performed in this paper, along with what data partitions were available and included Data type taxa data https://binary.pensoft.net/file/484987This dataset is made available under the Open Database License (http://opendatacommons.org/licenses/odbl/1.0/). The Open Database License (ODbL) is a license agreement intended to allow users to freely share, modify, and use this Dataset while maintaining this same freedom for others, provided that the original source and author(s) are credited.Michael S. Caterino, Alexey K. Tishechkin

Supplementary material 2 Morphological and molecular phylogenetic data (NEXUS) for analysis of *Phelister blairi* group Data type phylogenetic https://binary.pensoft.net/file/484988This dataset is made available under the Open Database License (http://opendatacommons.org/licenses/odbl/1.0/). The Open Database License (ODbL) is a license agreement intended to allow users to freely share, modify, and use this Dataset while maintaining this same freedom for others, provided that the original source and author(s) are credited.Michael S. Caterino, Alexey K. Tishechkin

## References

[B1] Bickhardt H (1917) Histeridae. In: Wytsman P (Ed.) Genera Insectorum, 166a, b. Martinus Nijhoff, The Hague, 302 pp.

[B2] Caterino MS Tishechkin AK Dégallier N (2012) A revision of the genus *Mecistostethus* Marseul (Coleoptera: Histeridae). ZooKeys 213: 63–78. 10.3897/zookeys.213.3552 PMC3426875 22933855

[B3] Caterino MS Tishechkin AK (2013a) A systematic revision of the genus *Operclipygus* Marseul. ZooKeys. 271: 1–401. 10.3897/zookeys.271.4062 PMC3652427 23717185

[B4] Caterino MS Tishechkin AK (2013b) A systematic revision of the genus *Baconia* Lewis. ZooKeys 343: 1–297. 10.3897/zookeys.343.5744 PMC3817432 24194656

[B5] Caterino MS Tishechkin AK (2014) New genera and species of Neotropical Exosternini. ZooKeys 381: 11–78. 10.3897/zookeys.381.6772 PMC3950425 24624014

[B6] Caterino MS Tishechkin AK (2015) Phylogeny and generic limits in New World Exosternini (Coleoptera: Histeridae: Histerinae). Systematic Entomology 40: 109–142. 10.1111/syen.12095

[B7] Caterino MS Tishechkin AK (2016) A systematic revision of *Megalocraerus* Lewis. ZooKeys 557: 59–78. 10.3897/zookeys.557.7087 PMC4740836 26877699

[B8] Caterino MS Tishechkin AK (2019) A revision of the *Phelister haemorrhous* species group (Histeridae: Exosternini). ZooKeys 854: 41–88. 10.3897/zookeys.854.35133 31231158 PMC6579788

[B9] Caterino MS Tishechkin AK Proudfoot GA (2013) A new genus and species of North American Exosternini associated with cavity nesting owls, and a reassignment of *Phelister simoni* Lewis (Histeridae: Histerinae: Exosternini). Coleopterists Bulletin 67(4): 557–565. 10.1649/0010-065X-67.4.557

[B10] Helava JVT Howden HF Ritchie AJ (1985) A review of the New World genera of the myrmecophilous and termitophilous subfamily Hetaeriinae (Coleoptera: Histeridae). Sociobiology 10(2): 127–382.

[B11] Hinton HE (1935) New American Histeridae. Stylops 4: 57–65. 10.1111/j.1365-3113.1935.tb00556.x

[B12] Kanaar P (1997) New attaphilous Histeridae from Suriname, with notes on other species. Zoologische Mededelingen 71: 277–286.

[B13] Lewis G (1898) On new species of Histeridae and notices of others. Annals and Magazine of Natural History 7: 156–181. 10.1080/00222939808678030

[B14] Marseul SA (1889) Les descriptions de nouvelles especies d’Histerides du genre *Phelister* (2^e^ partie). Bulletin de la Société entomologique de France 1889: cxxxviii–cxl.

[B15] Mazur S (1984) A World Catalogue of Histeridae (Coleoptera). Polskie Pismo Entomologiczne 54(3/4): 1–376.

[B16] Mazur S (2011) A concise catalogue of the Histeridae (Coleoptera). Warsaw University of Life Sciences – SGGW Press, Warsaw, 332 pp.

[B17] Ôhara M (1994) A revision of the superfamily Histeroidea of Japan (Coleoptera). Insecta Matsumurana, New Series 51: 1–283.

[B18] Reichensperger A (1939) Beitrage zur kenntnis der myrmecophilenfauna Costa Ricas und Brasiliens VII, nebst beschreibung der konigin von Eciton (Acamatus) pilosum. Zoologische Jahrbücher. Abteilung für Systematik, Geographie und Biologie der Tiere 73: 261–300.

[B19] Schmidt J (1893a) Neue Histeriden. Entomologische Nachrichten 19: 5–16. 10.1002/mmnd.48018930117

[B20] Schmidt J (1893b) Zwölf neue *Phelister*. Entomologische Nachrichten 19: 81–91.

[B21] Swofford DL (2002) PAUP*. Phylogenetic Analysis Using Parsimony (*and Other Methods). Sinauer & Assoc., Sunderland.

[B22] Wenzel RL Dybas HS (1941) New and little known neotropical Histeridae (Coleoptera). Fieldiana, Zoology 22(7): 433–472. 10.5962/bhl.title.3828

