# Supplementary material for: Recognition and revision of the Phelister blairi group (Histeridae, Histerinae, Exosternini)
Source: Zookeys. 2020 Dec 9;1001:1–154. doi: 10.3897/zookeys.1001.58447 (PMC7744391; doi:10.3897/zookeys.1001.58447)
Supplement: Supplementary material 1 — Table S1. Taxa selected for the phylogenetic reanalysis performed in this paper, along with what data partitions were available and included [file zookeys-1001-001-s001.docx]

**Supplemental Table 1.** Taxa selected for the phylogenetic reanalysis performed in this paper, along with what data partitions were available and included. Members of the *P. blairi* group are listed first, with subgroup also indicated. The subsequent taxa represent other Neotropical Exosternini included to assess monophyly of the *P. blairi* group with respect to various other Neotropical lineages. The taxa explicitly listed as ‘Outgroups’ represent extralimital Exosterini and other tribes of Histerinae, as well as the putatively closely related Haeteriinae. Taxa listed within single quotes (‘) represent informal morphospecies designations and, in a few cases, tentative generic assignments.

| **Taxon** | **morphology** | **COI** | **18S** | **28S** |
| --- | --- | --- | --- | --- |
| *Phelister conjunctus (blairi - amazoniae)* | **•** |  |  |  |
| *Phelister amazoniae (blairi – amazoniae)* | **•** |  |  |  |
| *Phelister annulatus (blairi – amazoniae)* | **•** |  |  |  |
| *Phelister arcuatus (blairi – amazoniae)* | **•** |  |  |  |
| *Phelister chabooae (blairi – amazoniae)* | **•** |  |  |  |
| *Phelister fraternus (blairi – amazoniae)* | **•** |  |  |  |
| *Phelister geijskesi (blairi – amazoniae)* | **•** |  |  |  |
| *Phelister morbidus (blairi – amazoniae)* | **•** |  |  |  |
| *Phelister notandus (blairi – amazoniae)* | **•** |  |  |  |
| *Phelister sphaericus (blairi – amazoniae)* | **•** |  |  |  |
| *Phelister striatinotum (blairi – amazoniae)* | **•** |  |  |  |
| *Phelister asperatus (blairi – blairi)* | **•** |  |  |  |
| *Phelister blairi (blairi – blairi)* | **•** |  |  |  |
| *Phelister dilatatus (blairi – blairi)* | **•** |  |  |  |
| *Phelister erwini (blairi – blairi)* | **•** | **•** | **•** |  |
| *Phelister fimbriatus (blairi – blairi)* | **•** | **•** | **•** |  |
| *Phelister geminus (blairi – blairi)* | **•** |  | **•** |  |
| *Phelister genieri (blairi – blairi)* | **•** |  |  |  |
| *Phelister globosus (blairi – blairi)* | **•** |  |  |  |
| *Phelister ifficus (blairi – blairi)* | **•** |  |  |  |
| *Phelister inbio (blairi – blairi)* | **•** |  |  |  |
| *Phelister marginatus (blairi – blairi)* | **•** |  |  |  |
| *Phelister miscellus (blairi – blairi)* | **•** | **•** | **•** |  |
| *Phelister parana (blairi – blairi)* | **•** | **•** |  |  |
| *Phelister pervagatus (blairi – blairi)* | **•** | **•** | **•** |  |
| *Phelister praedatoris (blairi – blairi)* | **•** |  |  |  |
| *Phelister pretiosus (blairi – blairi)* | **•** |  |  |  |
| *Phelister sculpturatus (blairi – blairi)* | **•** |  |  |  |
| *Phelister serratus (blairi – blairi)* | **•** | **•** | **•** |  |
| *Phelister sparsus (blairi – blairi)* | **•** |  |  |  |
| *Phelister spectabilis (blairi – blairi)* | **•** |  |  |  |
| *Phelister stellans (blairi – blairi)* | **•** | **•** | **•** |  |
| *Phelister trigonisternus (blairi – blairi)* | **•** |  |  |  |
| *Phelister tunki (blairi – blairi)* | **•** |  |  |  |
| *Phelister uniformis (blairi – blairi)* | **•** |  |  |  |
| *Phelister vazdemelloi (blairi – blairi)* | **•** |  |  |  |
| *Phelister curvipes (blairi – curvipes)* | **•** |  |  |  |
| *Phelister vilavelha (blairi – curvipes)* | **•** |  |  |  |
| *Phelister congruens (blairi – gregarius)* | **•** |  |  |  |
| *Phelister gregarius (blairi – gregarius)* | **•** |  | **•** |  |
| *Phelister incongruens (blairi – gregarius)* | **•** |  |  |  |
| *Phelister praecisus (blairi – gregarius)* | **•** |  |  |  |
| *Phelister praesignus (blairi – gregarius)* | **•** | **•** | **•** | **•** |
| *Phelister rudis (blairi – gregarius)* | **•** | **•** |  |  |
| *Phelister incertus (blairi – rio)* | **•** |  |  |  |
| *Phelister inscriptus (blairi – rio)* | **•** |  |  |  |
| *Phelister rio (blairi – rio)* | **•** |  |  |  |
| *Phelister semotus (blairi – rio)* | **•** | **•** |  |  |
| *Phelister uncinatus (blairi – rio)* | **•** |  |  |  |
| *Phelister almeidae (blairi – umens)* | **•** | **•** | **•** |  |
| *Phelister chicomendesi (blairi – umens)* | **•** |  |  |  |
| *Phelister matatlantica (blairi – umens)* | **•** | **•** | **•** |  |
| *Phelister microdens (blairi – umens)* | **•** |  |  |  |
| *Phelister umens (blairi – umens)* | **•** |  |  |  |
| *Phelister blairoides (blairi –* inc. sed.*)* | **•** | **•** | **•** |  |
| *Phelister okeefei (blairi –* inc. sed*.)* | **•** |  |  |  |
| *Phelister pirana (blairi –* inc. sed.*)* | **•** |  |  |  |
| *Baconia guartela* | **•** | **•** | **•** | **•** |
| *Baconia riouka* | **•** |  |  |  |
| *Baconia rufescens* | **•** | **•** | **•** | **•** |
| *Baconia salobrus* | **•** | **•** | **•** | **•** |
| *Chapischema doppelganger* | **•** |  |  |  |
| *Conchita propygidiale* | **•** | **•** | **•** | **•** |
| *Conocassis invaginata* | **•** |  |  |  |
| *Conocassis minor* | **•** | **•** | **•** | **•** |
| *Crenulister explanatus* | **•** |  |  |  |
| *Crenulister grossus* | **•** |  |  |  |
| *Crenulister paucitans* | **•** |  |  |  |
| *Crenulister seriatus* | **•** |  |  |  |
| *Crenulister simplex* | **•** |  |  |  |
| *Enkyosoma rockwelli* | **•** |  |  |  |
| *Hypobletus subridens* | **•** | **•** | **•** | **•** |
| *Hypobletus* ‘sp10’ | **•** | **•** | **•** | **•** |
| *Kaszabister carinatus* | **•** |  |  |  |
| *Kaszabister rubellus* | **•** |  |  |  |
| *Lacrimorpha balbina* | **•** |  |  |  |
| *Lacrimorpha glabra* | **•** |  |  |  |
| *Mecistostethus flechtmanni* | **•** | **•** | **•** |  |
| *Mecistostethus seagorum* | **•** |  |  |  |
| *Megalocraerus rubricatus* | **•** |  |  |  |
| *Nunbergia exosternoides* | **•** |  |  |  |
| *Operclipygus crenatus* | **•** | **•** | **•** |  |
| *Operclipygus dubitabilis* | **•** |  |  |  |
| *Operclipygus florifaunensis* | **•** | **•** | **•** | **•** |
| *Operclipygus longidens* | **•** | **•** |  |  |
| *Operclipygus marginellus* | **•** |  |  |  |
| *Operclipygus mirabilis* | **•** |  |  |  |
| *Operclipygus schlingeri* | **•** |  |  |  |
| *Operclipygus striatellus* | **•** | **•** | **•** | **•** |
| *Operclipygus subterraneus* | **•** | **•** | **•** |  |
| *Operclipygus teapensis* | **•** |  |  |  |
| *Operclipygus variabilis* | **•** | **•** | **•** |  |
| *Phelister* ‘’Bahia1b’ | **•** |  |  |  |
| *Phelister ‘*aduncus_B’ | **•** |  |  |  |
| *Phelister* ‘AKT_SanLorenzo_sp21’ | **•** |  |  |  |
| *Phelister* ‘AKT_Yasuni_sp17’ | **•** |  |  |  |
| *Phelister* ‘AKT_Yasuni_sp18’ | **•** | **•** | **•** |  |
| *Phelister* ‘AKT_Yasuni_sp32’ | **•** |  |  |  |
| *Phelister* ‘AKT_Yasuni_sp6’ | **•** |  |  |  |
| *Phelister* ‘AKT_YasuniGen3sp3’ | **•** | **•** | **•** |  |
| *Phelister* ‘Bigwilliamsi’ | **•** |  |  |  |
| *Phelister* ‘Bolivia6’ | **•** |  |  |  |
| *Phelister* ‘Chamela1’ | **•** |  |  |  |
| *Phelister* ‘CMN2004.3’ | **•** |  |  |  |
| *Phelister* ‘CMN2004.6’ | **•** |  |  |  |
| *Phelister* ‘CMN2004.9’ | **•** |  |  |  |
| *Phelister* ‘Columbia1’ | **•** |  |  |  |
| *Phelister* ‘FMNH2010.5’ | **•** |  |  |  |
| *Phelister* ‘InbioD’ | **•** |  |  |  |
| *Phelister* ‘Jujuy’ | **•** |  |  |  |
| *Phelister* ‘KUNHM2010.11’ | **•** | **•** | **•** |  |
| *Phelister* ‘KUNHM2010.12’ | **•** | **•** | **•** |  |
| *Phelister* ‘KUNHM2010.13’ | **•** | **•** | **•** | **•** |
| *Phelister* ‘KUNHM2010.14’ | **•** |  |  |  |
| *Phelister* ‘KUNHM2010.15’ | **•** |  |  |  |
| *Phelister* ‘KUNHM2010.16’ | **•** |  |  |  |
| *Phelister* ‘KUNHM2010.18’ | **•** |  |  |  |
| *Phelister* ‘KUNHM2010.19’ | **•** | **•** | **•** |  |
| *Phelister* ‘KUNHM2010.20’ | **•** |  |  |  |
| *Phelister* ‘KUNHM2010.21’ | **•** | **•** | **•** | **•** |
| *Phelister* ‘KUNHM2010.30’ | **•** |  |  |  |
| *Phelister* ‘KUNHM2010.6’ | **•** | **•** | **•** |  |
| *Phelister* ‘KUNHM2010.7’ | **•** |  |  |  |
| *Phelister* ‘LaSalle1’ | **•** | **•** | **•** | **•** |
| *Phelister* ‘LasCruces’ | **•** |  |  |  |
| *Phelister* ‘MazursBalzanii’ | **•** |  |  |  |
| *Phelister* ‘ND23b’ | **•** | **•** | **•** |  |
| *Phelister* ‘ND56a’ | **•** |  |  |  |
| *Phelister* ‘nr.luculentus’ | **•** |  |  |  |
| *Phelister* ‘nr.nanus’ | **•** |  |  |  |
| *Phelister* ‘rufigroup5 | **•** |  |  |  |
| *Phelister* ‘TAMU2010.1’ | **•** |  |  |  |
| *Phelister* ‘willBraz1’ | **•** |  |  |  |
| *Phelister* ‘willBraz2’ | **•** |  |  |  |
| *Phelister* ‘willBraz3’ | **•** |  |  |  |
| *Phelister* ‘willFrG1’ | **•** |  |  |  |
| *Phelister aduncus* | **•** |  |  |  |
| *Phelister affinis* | **•** |  |  |  |
| *Phelister* *balzanii* | **•** |  |  |  |
| *Phelister* *bipulvinatus* | **•** |  |  |  |
| *Phelister* *bistriatus* | **•** | **•** | **•** |  |
| *Phelister* *bolivianus* | **•** |  |  |  |
| *Phelister* *bovinus* | **•** |  |  |  |
| *Phelister brevis* | **•** | **•** | **•** | **•** |
| *Phelister brevistrius* | **•** | **•** | **•** | **•** |
| *Phelister carinifrons* | **•** |  |  |  |
| *Phelister colombiae* | **•** |  |  |  |
| *Phelister completus* | **•** |  |  |  |
| *Phelister condor* | **•** |  |  |  |
| *Phelister daugar* | **•** |  |  |  |
| *Phelister degallieri* | **•** |  |  |  |
| *Phelister desbordesi* | **•** |  |  |  |
| *Phelister egincola* | **•** | **•** | **•** | **•** |
| *Phelister flectohumerale* | **•** |  |  |  |
| *Phelister fractistrius* | **•** | **•** | **•** |  |
| *Phelister fulvulus* | **•** |  |  |  |
| *Phelister globiformis* | **•** |  |  |  |
| *Phelister gracilis* | **•** |  |  |  |
| *Phelister haemorrhous* | **•** | **•** | **•** |  |
| *Phelister interrogans* | **•** |  |  |  |
| *Phelister interruptus* | **•** |  |  |  |
| *Phelister latus* | **•** |  |  |  |
| *Phelister luculentus* | **•** | **•** | **•** | **•** |
| *Phelister mobilensis* | **•** |  |  |  |
| *Phelister nanus* | **•** |  |  |  |
| *Phelister nidicola* | **•** |  |  |  |
| *Phelister panamensis* | **•** | **•** | **•** |  |
| *Phelister petro* | **•** | **•** | **•** | **•** |
| *Phelister praecox* | **•** |  |  |  |
| *Phelister pulvis* | **•** |  |  |  |
| *Phelister purgamenticolus* | **•** |  |  |  |
| *Phelister pusio* | **•** | **•** | **•** | **•** |
| *Phelister pygmaeus* | **•** |  |  |  |
| *Phelister rectisternus* | **•** |  |  |  |
| *Phelister rouzeti* | **•** |  |  |  |
| *Phelister rubens* | **•** | **•** | **•** | **•** |
| *Phelister ruptistrius* | **•** | **•** |  | **•** |
| *Phelister sanguinipennis* | **•** | **•** | **•** |  |
| *Phelister* ‘SEAG2019_1’ | **•** |  |  |  |
| *Phelister subrotundus* | **•** | **•** | **•** | **•** |
| *Phelister testudo* | **•** | **•** | **•** |  |
| *Phelister tremolerasi* | **•** |  |  |  |
| *Phelister tristriatus* | **•** |  |  |  |
| *Phelister vernus* | **•** | **•** | **•** | **•** |
| *Phelister vibius* | **•** | **•** | **•** | **•** |
| *Phelister weberi* | **•** |  |  |  |
| *Phelister* *williamsi* | **•** |  |  |  |
| *Pluricosta onthophilodes* | **•** |  |  |  |
| *Pseudister rufulus* | **•** |  |  |  |
| *Pseudister suturalis* | **•** |  |  |  |
| *Pyxister devorator* | **•** |  |  |  |
| *Pyxister labralis* | **•** |  |  |  |
| *Scaptorus pyramus* | **•** |  |  |  |
| *Strigister simoni* | **•** |  |  |  |
| *Yarmister* ‘sp1’ | **•** |  |  |  |
| *Yarmister emersoni* | **•** |  |  |  |
| ‘enig_excavate_mesotibiae’ | **•** |  |  |  |
| ‘enig_Florayfauna’ | **•** |  |  |  |
| ‘enig_SanNicolau’ | **•** |  |  |  |
| ‘nrConchita’ | **•** |  |  |  |
| ‘nrConchita2’ | **•** |  |  |  |
| ‘Pseud_1’ | **•** |  |  |  |
| ‘Pseud_5’ | **•** |  |  |  |
| ‘Pseud_chancani’ | **•** | **•** | **•** | **•** |
| ‘Pseud_frontalhorn’ | **•** |  |  |  |
| ‘Pseud_INB2’ | **•** |  |  |  |
| ‘Pseud_JesusI.’ | **•** |  |  |  |
| ‘Pseud_M3’ | **•** |  |  |  |
| ‘Pseud_M4’ | **•** |  |  |  |
| ‘Pseud_M5’ | **•** |  |  |  |
| ‘Pseud_MG’ | **•** |  |  |  |
| ‘Pseud_plicaeTrigona’ | **•** |  |  |  |
| ‘Pseud_pronotalplicae’ | **•** | **•** | **•** | **•** |
| ‘Pseud_USNM1’ | **•** | **•** | **•** | **•** |
| ‘sang_CMNC1’ | **•** |  |  |  |
| ‘sang_fracti2’ | **•** |  |  |  |
| ‘sang_InbioJ’ | **•** |  |  |  |
| ‘sang_MatoGrosso’ | **•** |  |  |  |
| ‘sang_ND9’ | **•** | **•** | **•** |  |
| ‘sang_nr.pauli_bicolor’ | **•** | **•** | **•** |  |
| ‘sang_nr.pauli_bicolor2’ | **•** |  |  |  |
| ‘sang_nr.wideaedeagus’ | **•** |  |  |  |
| ‘VenezuelaCavePseudister’ | **•** |  |  |  |
| Outgroup-*Anaglymma congonis* | **•** | **•** | **•** | **•** |
| Outgroup-*Coelocraera cohici* | **•** | **•** | **•** | **•** |
| Outgroup-*Exosternus* sp. | **•** |  |  |  |
| Outgroup-*Paratropus* ‘sp2’ | **•** | **•** | **•** |  |
| Outgroup-*Platybletes stirpium* | **•** | **•** |  | **•** |
| Outgroup-*Platysomatinus diversicollis* | **•** | **•** | **•** | **•** |
| Outgroup-*Spathochus coyei* | **•** |  |  |  |
| Outgroup-*Hister unicolor* (Histerini) | **•** | **•** | **•** | **•** |
| Outgroup-*Hololepta vicina* (Hololeptini) | **•** | **•** | **•** |  |
| Outgroup-*Omalodes grossus* (Omalodini) | **•** | **•** | **•** | **•** |
| Outgroup-*Platysoma punctigerum* (Platysomatini) | **•** | **•** | **•** | **•** |
| Outgroup-*Haeterius morsus* (Haeteriinae) | **•** | **•** | **•** | **•** |
